# Global, regional, and national mortality trends in youth aged 15–24 years between 1990 and 2019: a systematic analysis

**DOI:** 10.1016/S2214-109X(21)00023-1

**Published:** 2021-03-01

**Authors:** Bruno Masquelier, Lucia Hug, David Sharrow, Danzhen You, Colin Mathers, Patrick Gerland, Leontine Alkema, UN Inter-agency Group for Child Mortality Estimation

**Affiliations:** a Catholic University of Louvain, Louvain-la-Neuve, Belgium; b Division of Data, Analytics, Planning and Monitoring, UNICEF, New York, NY, USA; c Technical Advisory Group of the UN Inter-agency Group for Child Mortality Estimation, UN, New York, NY, USA; d Population Division, UN, New York, NY, USA; e University of Massachusetts Amherst, Amherst, MA, USA

## Abstract

**Background:**

The global health community is devoting considerable attention to adolescents and young people, but risk of death in this population is poorly measured. We aimed to reconstruct global, regional, and national mortality trends for youths aged 15–24 years between 1990 and 2019.

**Methods:**

In this systematic analysis, we used all publicly available data on mortality in the age group 15–24 years for 195 countries, as compiled by the UN Inter-agency Group for Child Mortality Estimation. We used nationally representative vital registration data, estimated the completeness of death registration, and extracted mortality rates from surveys with sibling histories, household deaths reported in censuses, and sample registration systems. We used a Bayesian B-spline bias-reduction model to generate trends in _10_q_15_, the probability that an adolescent aged 15 years would die before reaching age 25 years. This model treats observations of the _10_q_15_ probability as the product of the actual risk of death and an error multiplier that varies depending on the data source. The main outcome that we assessed was the levels of and trends in youth mortality and the global and regional mortality rates from 1990 to 2019.

**Findings:**

Globally, the probability of an individual dying between age 15 years and 24 years was 11·2 deaths (90% uncertainty interval [UI] 10·7–12·5) per 1000 youths aged 15 in 2019, which is about 2·5 times less than infant mortality (28·2 deaths [27·2–30·0] by age 1 year per 1000 live births) but is higher than the risk of dying from age 1 to 5 (9·7 deaths [9·1–11·1] per 1000 children aged 1 year). The probability of dying between age 15 years and 24 years declined by 1·4% per year (90% UI 1·1–1·8) between 1990 and 2019, from 17·1 deaths (16·5–18·9) per 1000 in 1990; by contrast with this total decrease of 34% (27–41), under-5 mortality declined by 59% (56–61) in this period. The annual number of deaths declined from 1·7 million (90% UI 1·7–1·9) in 1990 to 1·4 million (1·3–1·5) in 2019. In sub-Saharan Africa, the number of deaths increased by 20·8% from 1990 to 2019. Although 18·3% of the population aged 15–24 years were living in sub-Saharan Africa in 2019, the region accounted for 37·9% (90% UI 34·8–41·9) of all worldwide deaths in youth.

**Interpretation:**

It is urgent to accelerate progress in reducing youth mortality. Efforts are particularly needed in sub-Saharan Africa, where the burden of mortality is increasingly concentrated. In the future, a growing number of countries will see youth mortality exceeding under-5 mortality if current trends continue.

**Funding:**

UN Children's Fund, Bill & Melinda Gates Foundation, United States Agency for International Development.

## Introduction

Remarkable progress in improving child survival has been achieved over the past three decades. The number of deaths between birth and age 15 years fell by 57% from 1990, when it was 14·1 million (90% uncertainty interval [UI] 14·0–14·4), to 2019, when it was 6·1 million (5·9–6·5).^1^ Increased chances of surviving in the first 15 years of life have resulted in a growth in the number of adolescents aged 15–19 years and young people aged 20–24 years, a growth amplified by the demographic transition taking place globally. In 2020, there were 1·2 billion people aged 15–24 years worldwide, an age group referred to as “youth” by the UN.^2^ This period of life is increasingly seen as key to securing gains in health and wellbeing made in childhood and early adolescence,^3^, ^4^ as emphasised by the *Lancet* Commission on adolescents and wellbeing.^5^ Several global trends are contributing to better health in youth, including extensions to the length of education, improved health and nutrition in young children, and a delayed entry into marriage and parenthood. However, threats are also posed to their health by climate change and environmental degradation, mass migration and conflicts, and unemployment and exposure to initiatives promoting unhealthy lifestyles.^5^ These factors might explain why mortality rates among youth aged 15–24 years appear to decline less rapidly than those among young children. Additionally, the relative burden of injuries and non-communicable diseases grow as children transition to adolescence and early adulthood, causing shifts in causes of death. In their analysis of vital registration data from 50 countries in 2011, Viner and colleagues^6^ compared mortality rates in various age groups (1–4 years, 5–9 years, 10–14 years, 15–19 years, and 20–24 years). They showed that those aged 15–24 years recorded the slowest decline in mortality over the period 1955 to 2004, which was about half that of children aged 1–4 years. This assessment, however, was restricted to countries with comprehensive systems of death registration.

**Research in context**

**Evidence before this study**

We searched PubMed with the terms “youth mortality”, “mortality”, and “adolescent” for papers published between Jan 1, 1970, and July 8, 2020, in English or French. The published literature is based either on countries with accurate vital statistics or modelled estimates from the Global Burden of Disease Study, WHO, or the UN Population Division. No comprehensive study has been done to reconstruct mortality trends in the age group 15–24 years from mortality measurements specifically for this age range. Previous attempts to estimate youth mortality in countries without high-quality vital registration used model age patterns of mortality to infer youth mortality from child mortality (age 0–5 years), adult mortality (age 15–60 years), or both.

**Added value of this study**

We assembled a large database using different sources: vital statistics (with adjustments for the incompleteness of death registration), sibling survival histories, information collected in censuses on recent household deaths, and national sources, such as sample registration systems. Using a Bayesian penalised B-spline regression model, we reconstructed trends in the probability of dying between the 15th and 25th birthdays. To our knowledge, this is the first study on youth mortality that uses the same methods as those used to monitor trends in child mortality for the Sustainable Development Goals, avoids the use of covariates, and directly exploits mortality measurements related to youth. We show that most deaths are concentrated in countries where vital statistics are incomplete, highlighting the importance of using age-specific survey and census estimates.

**Implications of all the available evidence**

The decline in youth mortality has been modest since 1990, and much slower than that of young children. The annual rate of reduction has been only 1·4% since 1990, with no significant acceleration between 1990–2000 and 2000–2019. As the youth population has grown, the number of deaths has declined only marginally, from 1·7 million to 1·4 million between 1990 and 2019. Over the same period, the number of deaths of children under 5 years more than halved, from 12·5 million to 5·2 million. Youth mortality accounts for a growing share of premature deaths, and age patterns of mortality are gradually changing; in all regions except sub-Saharan Africa, the risk of dying between ages 15 years and 25 years globally now surpasses the risk of death in early childhood (between ages 1 year and 5 years). Strong political commitment and investments have helped to save millions of lives in neonates and young children during the past decades. More than ever, similar efforts are needed to reduce preventable deaths in youth.

Few low-income and middle-income countries have comprehensive vital registration systems that are able to generate reliable and timely mortality rates, and existing estimates of youth mortality in these countries are based on modelling. Trends in mortality for youth aged 15–24 years are generally inferred from life tables that are extrapolated using the under-5 mortality rate (_5_q_0_) and the risk of dying between the age 15 and 60 years (_45_q_15_). This strategy has been used in the Global Burden of Disease Study^7^ and the World Population Prospects (WPP) report.^2^ There is some variation in the way in which the probabilities _5_q_0_ and _45_q_15_ are estimated and combined with model life tables (summarising expected age patterns of mortality in countries with high-quality data), but in both cases, mortality rates in youth are not directly measured; they are inferred from a combination of _5_q_0_ and _45_q_15_. Information contained in surveys or censuses on age patterns of mortality within the bracket of 15–59 years is lost because the age-specific mortality rates are summarised in the summary index _45_q_15_. Youth mortality could be evolving at rates that are imperfectly reflected by the combination of the probabilities _5_q_0_ and _45_q_15_. For example, the development of the HIV/AIDS epidemic will differentially affect youth aged 15–24 years and adults aged 25–59 years, partly because adolescents and young people have lower HIV testing coverage and lower adherence to antiretroviral treatment than adults older than 25 years.^8^ Summary mortality indices, such as _45_q_15_, will also conceal the excess mortality that is regularly observed during the transition to adulthood. This so-called young adult mortality hump (typically from age 15 to 34 years) is mostly attributed to external mortality, HIV/AIDS, and maternal mortality.^9^ It is therefore essential to measure age-specific mortality directly.

In this study, we aimed to determine global, regional, and national trends in mortality for youth aged 15–24 years between 1990 and 2019. Our indicator, denoted _10_q_15_, represented the probability that an adolescent aged 15 years would die before reaching age 25 years if they were exposed to the age-specific mortality rates of that year.

## Methods

### Overview

In this systematic analysis, we compiled all publicly available nationally representative data on mortality in the age group 15–24 years (_10_q_15_) for 195 countries. We used a Bayesian B-spline bias-reduction model^10^ to obtain country-specific trends and short-term projections to 2019 in _10_q_15_. These estimates were adopted by the UN Inter-agency Group for Child Mortality Estimation (UN IGME), and our approach is similar to that taken by the UN IGME to estimate mortality in children aged less than 5 years^1^ and those aged 5–14 years.^11^ Taken together, these estimates provide a basis for consistently monitoring mortality trends in the first 25 years of life.

### Database construction

The UN IGME assesses the quality of surveys, censuses, and vital statistics to generate mortality estimates that are internationally comparable using statistical models. An empirical database is updated annually and available in the public domain with final estimates. For this study, we searched the same data sources as in our previous studies^1^, ^11^ but extracted the information on mortality among those aged 15–24 years. We present a summary of these data series in what follows and provide the full list in the appendix (pp 28–42).

Nationally representative vital registration data are provided by Member States to WHO. Mortality data from vital registration systems were available for 5989 country-years in total for the period 1990–2019, for 138 different countries. We assumed that death registration was complete in 39 countries included in the Human Mortality Database, which has strict data quality requirements. For the other countries, we used demographic methods comparing the age distribution of deaths between two censuses with the age distribution of the population enumerated in these censuses, to select country-years for which vital registration data are included and to compute time-dependent adjustment factors in case of incomplete registration.^12^ We also used estimates from sample vital registration systems in India and Bangladesh, the Chinese national mortality surveillance system,^13^ and the Rapid Mortality Surveillance in South Africa.^14^

To measure deaths of those aged 15–59 years in countries without vital registration data, sibling survival histories are collected in large-scale household surveys. The respondents provide an exhaustive list of all their brothers and sisters, with information on their gender, survival status, current age for surviving siblings or age at death, and the timing of death for the deceased. Sibling histories were drawn from 148 Demographic and Health Surveys done in 52 countries. The database also includes estimates from 42 WHO World Health Surveys, nine Multiple Indicator Cluster Surveys, and other country-specific surveys.

However, sibling histories can be plagued by reporting errors.^15^ Siblings—living or deceased—can be omitted, and the ages at the survey or death can be misreported. The time of death can also be affected by heaping or systematic misstatement. To account for the possibility of bias, we modelled recall errors as a function of the retrospective period, by contrasting the sibling-based estimates with vital registration data for overlapping periods.

Before fitting the Bayesian B-spline bias-reduction model, we excluded some surveys based on the plausibility of age patterns of mortality. We considered two indices of mortality: _10_q_15_ (youth mortality from sibling histories) and _5_q_0_ (under-5 mortality from birth histories). We compared the _10_q_15_ to _5_q_0_ relationship observed in each survey with an expected pattern obtained from (1) the Human Mortality Database, representing the historical experience of countries with a long statistical tradition, (2) datasets from Health and Demographic Surveillance Systems, and (3) data from (sample) registration systems in Mauritius and India. From this reference database, we used a log-quadratic regression to predict, for a given level of _5_q_0_, what the expected level of _10_q_15_ would be (appendix p 11). We excluded from the model fitting all surveys depicting a _10_q_15_ probability that was below the lower bound of the 95% prediction intervals. This quality requirement led to the exclusion of nine (6%) of 148 Demographic and Health Surveys, three (33%) of nine Multiple Indicator Cluster Surveys, and 13 (31%) of 42 World Health Surveys.

Censuses often include questions on household deaths in the past 12 months, which can be used to calculate mortality estimates for the population aged 15–24 years. The calculation of the probability _10_q_15_ was derived from a standard period abridged life table. The _10_q_15_ to _5_q_0_ relationship observed in the data was checked against the predictions of the log-quadratic regression, and estimates falling outside of the 95% predictions intervals were discarded. In most countries, we did not adjust for the incompleteness of death reporting in surveys and censuses because the Bayesian B-spline bias-reduction model has a data model to correct for recall errors. In China, a previous study^16^ provided census mortality estimates adjusted for the incompleteness of death reporting from 1982 to 2000. We used these estimates and evaluated the completeness of death reporting in the 2010 census.

### Data analysis

To estimate _10_q_15_, we used the following approach. Sampling and reporting errors affect survey and census data, and in many countries, the most recent data are several years old. A statistical model is needed to account for such biases and to make short-term extrapolations. We used the Bayesian B-spline bias-reduction model,^10^ treating observations of the _10_q_15_ probability as the product of the actual risk of death and a so-called error multiplier that will vary depending on the data source. Temporal trends in the logarithm of _10_q_15_ were modelled using B-splines, exchanging information across countries on the degree to which the logarithm of _10_q_15_ might deviate from a linear trend.

The Bayesian B-spline bias-reduction model includes a data model to correct for recall and selection biases in the data series. These biases vary depending on the source of the data. Mean biases within surveys are modelled as a linear function of the retrospective period of observation. Biases are estimated on the basis of consistency between each observation and other observations relating to the same periods, using a hierarchical model. The model takes into account differences in sampling and non-sampling variance between observations.

We ran an out-of-sample validation exercise to assess the model performance in estimating mortality for the age group of 15–24 years. A training dataset was constructed by eliminating all data collected in 2013 or later, so that about 20% of the data was omitted. This dataset was used to generate new estimates that would be obtained if more recent data were not available. The estimated values were compared with observed values from 2013 onwards and with mortality trends obtained from the full database. Validation measures indicate that the model works well for youth mortality (appendix pp 21–24). When predicting left-out observations after 2012, median errors are given by –0·6, median absolute errors are given by 2·2, and coverage of 90% prediction intervals is nominal.

Data from sample surveys, censuses, and vital registration systems might not accurately capture abrupt changes in mortality rates due to conflicts, natural disasters, or epidemics. Adjustments were introduced in 61 countries to account for crisis-related deaths. Excess deaths due to conflicts were estimated based on the Uppsala Conflict Data Programme dataset, the Armed Conflict Location and Event Data Project, and other country-specific data sources (appendix pp 18–20). Deaths due to natural disasters were obtained from the International Disaster Database. From crisis-related deaths, we estimated excess mortality in terms of the _10_q_15_ probability. We excluded input data referring to the years affected by the crises, fitted the model to the remaining data, and added the excess mortality to the smoothed trend.

Concerning countries with insufficient data, in 37 countries, accounting for 4·9% of the population aged 15–24 years in 2019, the input database had fewer than four data points or the period covered by the observations was less than 10 years. For these countries, the Bayesian B-spline bias-reduction model is not applicable because it does not use covariates to fill data gaps. The probability _10_q_15_ was modelled for these countries on the basis of the relationship between mortality in the age groups 0–4 years and 15–24 years, as observed in the 158 countries for which the Bayesian B-spline bias-reduction model was used. A mixed-effects linear model was used to regress log(_10_q_15_) against log(under-5 mortality rate), with random intercepts and random slopes for each region. The annual probability _10_q_15_ from 1990 to 2019 was predicted on the basis of country-year specific under-5 mortality rate estimates and coefficients of this regression.

To estimate the number of deaths among youth aged 15–24 years, the probability _10_q_15_ had to be split into two segments, referring to the age groups 15–19 years (_5_q_15_) and 20–24 years (_5_q_20_). To do so, a database of mortality measurement for adolescents aged 15–19 years was constructed from the data sources already mentioned. A penalised spline regression model was used to estimate country-specific trends in the logit transformation of *r*(log(*r*/(1–*r*))), where *r* is the ratio of the probability _5_q_15_ to the median model estimate of _10_q_15_. Using the probabilities _10_q_15_ and _5_q_15_, we derived the probability _5_q_20_. Age-specific probabilities were converted into central death rates and multiplied by population estimates from the WPP.^2^ The numbers of deaths are presented with 90% UIs obtained from the posterior samples of mortality rates. Uncertainty in population counts is not taken into account, because it is not available from the WPP. Mortality rates and numbers of deaths were calculated at the country level and were aggregated to obtain regional and global estimates.

A joint UNICEF and WHO country consultation was undertaken in 2020 to allow the ministries of health, national statistics offices, or other responsible government agencies to review the data inputs, the methodology, and the draft estimates, and to provide additional data. The database was updated for 63 countries after the consultation.

### Role of the funding source

The funders of the study had no role in the study design, data analysis, data interpretation, or writing of the report.

## Results

**Figure 1:**
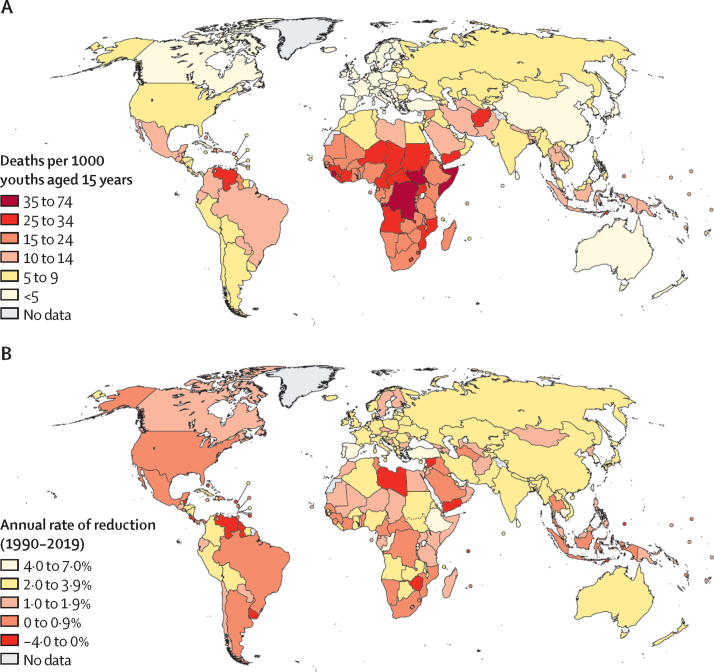
Probability of dying among youths aged 15–24 years in 2019 (A) and annual rate of reduction in that probability between 1990 and 2019 (B)

Globally, the probability of dying between the 15th and 25th birthday (_10_q_15_) was 11·2 deaths (90% UI 10·7–12·5) per 1000 youths aged 15 years in 2019 (table 1). The risk of dying between the 15th and 20th birthday (_5_q_15_) was 4·9 deaths (4·7–5·2) per 1000 youths aged 15 years in 2019, which increased in those aged 20–24 years (_5_q_20_) to 6·3 deaths (5·8–7·5) per 1000 youths aged 20 years. In west and central Africa in 2019, the probability _10_q_15_ was 24·6 deaths (21·8–30·4) per 1000 in 2019, which was twice the risk in Latin America and the Caribbean (12·3 [11·7–13·1]), four times the risk in East Asia and the Pacific (5·9 [4·8–7·8]), and eight times the risk in western Europe (3·0 [3·0–3·1]). Countries bearing the greatest burden were concentrated in sub-Saharan Africa: 34 (85%) of the 40 countries with the highest mortality rates were located in this region (figure 1).

**Table 1:** Global and regional probabilities of dying among youth aged 15–19 years, 20–24 years, and 15–24 years for 1990–2019

		**Probability _5_q_15_**			**Probability _5_q_20_**			**Probability _10_q_15_**			**Annual rate of reduction in _10_q_15_, 1990–2019**
		1990	2000	2019	1990	2000	2019	1990	2000	2019	
Sub-Saharan Africa		18·6 (17·6–21·1)	15·6 (15·2–16·9)	10·2 (9·5–11·5)	25·0 (22·3–32·6)	21·9 (20·8–24·2)	13·2 (11·8–17·0)	43·2 (41·5–51·2)	37·2 (36·7–39·8)	23·3 (22·1–27·3)	2·1% (1·6–2·7)
	Eastern and southern Africa	20·0 (19·1–23·5)	16·9 (16·6–18·7)	9·3 (8·6–10·7)	28·5 (25·4–34·6)	25·3 (24·4–28·6)	12·9 (11·3–17·1)	47·9 (46·5–54·8)	41·7 (41·6–45·8)	22·1 (20·7–26·7)	2·7% (2·1–3·1)
	West and central Africa	17·1 (14·6–20·1)	14·1 (12·9–15·6)	11·1 (9·7–13·2)	21·0 (15·7–34·3)	18·1 (15·6–21·0)	13·6 (10·4–19·1)	37·7 (33·4–51·3)	32·0 (30·0–34·6)	24·6 (21·8–30·4)	1·5% (0·6–2·6)
Middle East and north Africa		7·0 (6·3–7·8)	4·6 (4·4–4·9)	4·5 (4·2–4·8)	7·6 (5·7–13·8)	5·5 (4·8–6·3)	5·5 (4·8–6·7)	14·5 (12·8–20·8)	10·0 (9·4–10·9)	9·9 (9·2–11·3)	1·3% (0·7–2·5)
South Asia		10·5 (9·8–11·3)	8·0 (7·7–8·4)	4·7 (4·1–5·4)	13·9 (12·0–16·5)	11·1 (10·2–12·1)	6·1 (4·4–8·7)	24·3 (22·5–26·7)	19·0 (18·2–20·0)	10·8 (8·8–13·6)	2·8% (2·0–3·6)
East Asia and Pacific		5·4 (4·9–6·1)	4·1 (3·8–4·4)	2·4 (2·1–2·8)	4·7 (3·4–6·6)	4·5 (3·8–5·4)	3·5 (2·5–5·2)	10·2 (9·0–11·9)	8·6 (8·0–9·5)	5·9 (4·8–7·8)	1·9% (0·8–2·8)
Latin America and Caribbean		6·0 (5·9–6·2)	5·4 (5·3–5·5)	5·0 (4·8–5·2)	8·8 (8·3–9·3)	7·9 (7·7–8·1)	7·3 (6·8–8·1)	14·7 (14·3–15·3)	13·2 (13·0–13·4)	12·3 (11·7–13·1)	0·6% (0·4–0·8)
North America		4·3 (4·2–4·4)	3·3 (3·3–3·4)	2·6 (2·4–2·7)	5·3 (5·1–5·6)	4·5 (4·4–4·7)	4·9 (4·2–5·7)	9·6 (9·4–9·8)	7·8 (7·7–8·0)	7·5 (6·7–8·3)	0·9% (0·5–1·2)
Europe and central Asia		4·7 (3·8–5·4)	4·0 (3·9–4·2)	1·9 (1·8–1·9)	6·0 (4·9–13·2)	6·3 (5·9–6·8)	2·7 (2·6–2·8)	10·7 (8·9–17·8)	10·3 (9·9–10·8)	4·5 (4·4–4·7)	3·0% (2·3–4·7)
	Western Europe	3·0 (3·0–3·0)	2·4 (2·3–2·4)	1·2 (1·2–1·2)	4·2 (4·1–4·2)	3·3 (3·3–3·4)	1·8 (1·8–1·9)	7·1 (7·1–7·2)	5·7 (5·7–5·7)	3·0 (3·0–3·1)	2·9% (2·8–3·0)
	Eastern Europe and central Asia	6·5 (4·6–7·9)	5·4 (5·1–5·7)	2·5 (2·5–2·6)	8·2 (5·8–24·0)	9·1 (8·3–10·2)	3·5 (3·4–3·8)	14·6 (10·8–30·2)	14·5 (13·7–15·6)	6·0 (5·9–6·3)	3·0% (2·0–5·5)
World		8·0 (7·7–8·3)	6·7 (6·6–6·9)	4·9 (4·7–5·2)	9·2 (8·6–11·0)	8·8 (8·4–9·2)	6·3 (5·8–7·5)	17·1 (16·5–18·9)	15·4 (15·1–15·9)	11·2 (10·7–12·5)	1·4% (1·1–1·8)

Probability _5_q_15_ is the probability of young people alive at age 15 years dying before reaching age 20 years, probability _5_q_20_ is the probability of young people alive at age 20 years dying before reaching age 25 years, and probability _10_q_15_ is the probability of young people aged 15 years dying before reaching age 25 years, all expressed per 1000 youths in 1990, 2000, and 2019, with 90% uncertainty intervals. Definitions of regional classifications are in the appendix (p 164).

**Figure 2:**
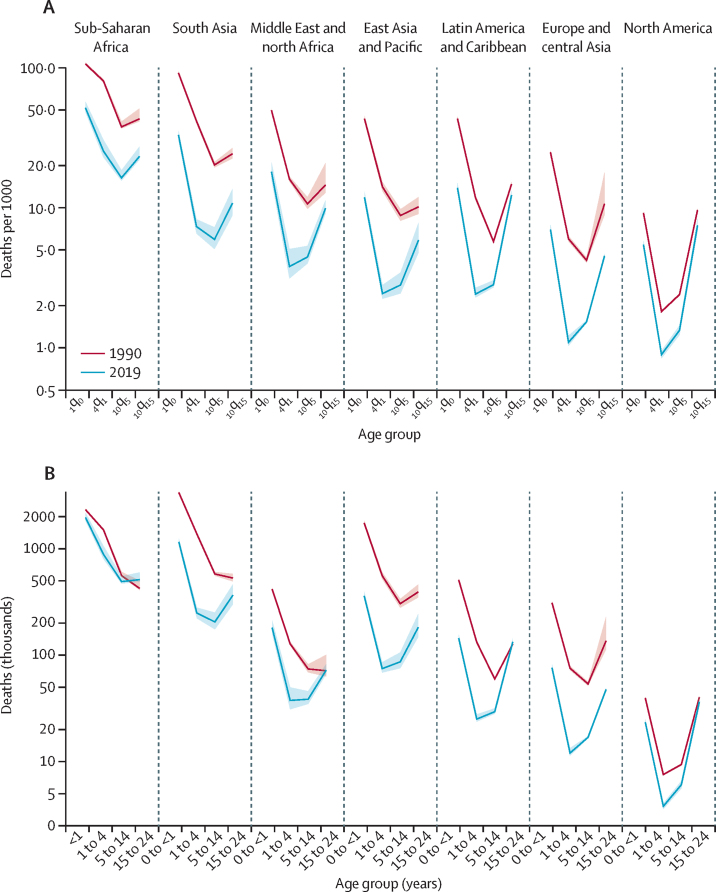
Regional trends in mortality for infants aged 0–1 years, children aged 1–4 years, older children and young adolescents aged 5–14 years, and youth aged 15–24 years in 1990 and 2019 (A), and absolute numbers of deaths by age group (B) Shaded areas represent 90% uncertainty intervals. Probability _1_q_0_ is the probability of infants alive at birth dying before reaching age 1 year, probability _1_q_4_ is the probability of children alive at age 1 year dying before reaching age 5 years, probability _10_q_5_ is the probability of young people alive at age 5 years dying before reaching age 15 years, and probability _10_q_15_ is the probability of youths aged 15 years dying before reaching age 25 years, all expressed per 1000 live births, children, or young people (depending on the age group considered). Definitions of regional classifications are in the appendix (p 164).

The world has made slow progress in preventing youth mortality. The probability _10_q_15_ declined by 1·4% per year (90% UI 1·1–1·8) between 1990 and 2019 (table 1), whereas the annual rate of reduction in under-5 mortality was more than two times higher (3·1% [2·8–3·3]).^1^ The annual rate of reduction in youth mortality increased between the period 1990–2000 (1·0% [0·6–2·0]) and the period 2000–2019 (1·7 [1·1–2·0]), but this increase was not significant (table 1). By contrast, there was a significant acceleration in under-5 mortality rate from 2·0% (1·9–2·2) in 1990–2000 to 3·7% (3·3–3·9) in 2000–2019.^1^ The decline in _10_q_15_ was rapid in Europe and central Asia (annual rate of reduction 3·0% [2·3–4·7]) and south Asia (2·8% [2·0–3·6]), but it was particularly slow in Latin America and the Caribbean (0·6% [0·4–0·8]) and North America (0·9% [0·5–1·2%]; table 1; figure 2). In 62 countries, there was no significant mortality decline, because the 90% UIs around the annual rate of reduction included 0. A significant mortality increase was observed in eight countries during the period, including Venezuela, Yemen, and Syria, due to crises (figure 1).

Although the probability of dying in the age group 15–24 years declined by 1·4% (90% UI 1·1 to 1·8) in 1990–2019, the population at risk increased globally by 0·6% each year over the same period.^2^ Hence, the global number of deaths among youth aged 15–24 years changed only slightly from 1·7 million (90% UI 1·7 to 1·9) in 1990 to 1·4 million (1·3 to 1·5) in 2019 (table 2; figure 2). Between 1990 and 2019, the number of deaths in youths in sub-Saharan Africa increased by 20·8% (90% UI 4·8 to 40·0), from 424 000 to 513 000 (table 2), because the mortality decline was outpaced by population growth; the population aged 15–24 years increased by 126% during this period in the region.^2^ The annual number of deaths in youth aged 15–24 years did not change significantly in eastern and southern Africa (1·4% [–9·8% to 20·1%]) during 1990–2019, but west and central Africa registered a large increase (49·2% [10·9 to 88·2]). There was no significant change in the number of deaths in North America, Latin America and the Caribbean, and the Middle East and north Africa. By contrast, the number of deaths declined by 31·0% (12·9 to 44·9) in south Asia, by 53·4% (36·5 to 64·5) in East Asia and the Pacific, and by 65·1% (58·0 to 79·1) in Europe and central Asia. Deaths are therefore increasingly concentrated in sub-Saharan Africa. This region hosted 18·3% of the population aged 15–24 years in 2019 but accounted for 37·9% (34·8 to 41·9) of deaths in this age group, increasing from 24·6% (22·4 to 27·3) in 1990.^2^ Because 27·1% (22·7 to 31·5%) of deaths occurred in south Asia, these two regions combined account for about two-thirds of the global number of deaths in 2019.

**Table 2:** Global and regional numbers of deaths among youth aged 15–19 years, 20–24 years, and 15–24 years in 1990–2019, with 90% uncertainty intervals

		**Number of deaths among youths aged 15–19 years (thousands)**			**Number of deaths among youths aged 20–24 years (thousands)**			**Number of deaths among youths aged 15–24 years (thousands)**			**Share of global deaths in youths aged 15–24 years, 2019**	**Relative change in number of deaths in youths aged 15–24 years, 1990–2019**
		1990	2000	2019	1990	2000	2019	1990	2000	2019		
Sub-Saharan Africa		201 (191 to 228)	229 (223 to 248)	243 (227 to 275)	223 (198 to 291)	269 (255 to 297)	270 (239 to 347)	424 (410 to 500)	497 (492 to 531)	513 (489 to 598)	37·9% (34·8 to 41·9)	20·8% (4·8 to 40·0)
	Eastern and southern Africa	116 (111 to 136)	132 (130 to 147)	115 (107 to 134)	136 (121 to 166)	165 (159 to 187)	140 (122 to 185)	252 (246 to 288)	297 (297 to 326)	255 (240 to 308)	18·9% (16·8 to 21·6)	1·4% (−9·8 to 20·1)
	West and central Africa	85 (73 to 101)	96 (88 to 107)	127 (111 to 151)	87 (65 to 142)	104 (89 to 120)	130 (99 to 184)	172 (154 to 230)	200 (189 to 215)	257 (230 to 315)	19·0% (16·6 to 22·3)	49·2% (10·9 to 88·2)
Middle East and north Africa		37 (33 to 41)	33 (32 to 36)	32 (30 to 35)	34 (26 to 63)	34 (30 to 39)	40 (35 to 49)	71 (63 to 101)	67 (63 to 73)	72 (67 to 82)	5·4% (4·7 to 6·0)	1·3% (−27·2 to 20·0)
South Asia		244 (228 to 261)	234 (225 to 245)	162 (142 to 186)	287 (247 to 339)	283 (259 to 308)	204 (146 to 289)	530 (493 to 581)	517 (496 to 541)	366 (301 to 462)	27·1% (22·7 to 31·5)	−31·0% (−44·9 to −12·9)
East Asia and Pacific		211 (190 to 236)	142 (133 to 154)	73 (63 to 86)	182 (132 to 252)	149 (127 to 179)	110 (80 to 163)	392 (349 to 461)	291 (272 to 321)	183 (149 to 243)	13·5% (10·9 to 17·0)	−53·4% (−64·5 to −36·5)
Latin America and Caribbean		55 (53 to 56)	56 (55 to 57)	54 (52 to 56)	72 (69 to 77)	75 (73 to 77)	79 (74 to 88)	127 (124 to 131)	131 (129 to 133)	133 (127 to 142)	9·9% (8·8 to 10·6)	4·8% (−0·9 to 12·5)
North America		17 (17 to 17)	15 (14 to 15)	12 (11 to 13)	23 (22 to 24)	19 (18 to 20)	24 (21 to 28)	40 (39 to 41)	34 (33 to 34)	36 (33 to 41)	2·7% (2·3 to 3·0)	−9·4% (−18·6 to 1·0)
Europe and central Asia		60 (48 to 69)	52 (50 to 53)	19 (19 to 19)	76 (63 to 169)	79 (74 to 86)	29 (28 to 30)	136 (114 to 229)	131 (126 to 138)	48 (47 to 49)	3·5% (3·2 to 3·7)	−65·1% (−79·1 to −58·0)
	Western Europe	19 (19 to 19)	14 (14 to 14)	6 (6 to 6)	29 (29 to 30)	20 (20 to 21)	10 (10 to 10)	48 (48 to 49)	34 (34 to 35)	16 (16 to 17)	1·2% (1·1 to 1·3)	−66·5% (−67·2 to −65·6)
	Eastern Europe and central Asia	41 (29 to 49)	38 (36 to 40)	13 (12 to 13)	47 (34 to 140)	58 (53 to 65)	19 (18 to 20)	88 (65 to 180)	96 (91 to 103)	31 (30 to 33)	2·3% (2·1 to 2·5)	−64·3% (−82·5 to −51·6)
World		824 (793 to 864)	761 (746 to 784)	595 (568 to 637)	898 (837 to 1080)	907 (870 to 955)	756 (696 to 894)	1722 (1668 to 1905)	1668 (1637 to 1717)	1351 (1288 to 1506)	100%	−21·6% (−29·4 to −13·0)

For 103 countries, we estimated that the completeness of death registration was equal to or higher than 80% after 2010. These countries accounted for only 20·3% (90% UI 18·2–21·5) of deaths in 2019. 25·9% (21·3–30·0) of deaths also occurred in China, Bangladesh, and India, where sample registration systems are in place. For the remaining 53·9% (50·7–58·6) of deaths, surveys and censuses were the primary sources of information.

Because the decline in youth mortality is out of sync with that of under-5 mortality, long-established age patterns of mortality are gradually being reversed. Globally, in 1990, the probability _10_q_15_ was about half the probability of dying between age 1 year and 5 years (17·1 deaths [90% UI 16·5–18·9] per 1000 youths *vs* 30·4 deaths [29·9–31·0] per 1000 children; table 1).^1^ Three decades later, an adolescent aged 15 years had a similar risk of dying before reaching age 25 years as a child aged 1 year had of dying before reaching age 5 years (11·2 deaths [10·7–12·5] per 1000 youths *vs* 9·7 deaths [9·1–11·1] per 1000 children in 2019),^1^ but it was about 2·5 times less than infant mortality (28·2 deaths [27·2–30·0] by age 1 year per 1000 livebirths). Regional trends in _1_q_0_, _4_q_1_, _10_q_5_, and _10_q_15_ show distinct age patterns (figure 2). In all regions except sub-Saharan Africa, mortality rates in youths (_10_q_15_) are now higher than child mortality rates (_4_q_1_). In Latin America and the Caribbean, the probability _10_q_15_ is close to that of infant mortality (_1_q_0_), and in North America it exceeds infant mortality.

## Discussion

Mortality remains excessively high in adolescents and young people aged 15–24 years, resulting in about 1·4 million deaths in 2019 globally. Beyond the cost in human lives, a huge emotional burden is associated with these deaths.^17^, ^18^ There are also enormous financial costs to families and society in general, particularly because young people have attained high levels of education and are gradually entering the labour force.

The global number of deaths in youth is substantially less than that in children younger than 5 years (5·2 million in 2019),^1^ and it is measured across a larger population (1·2 billion youths aged 15–24 years *vs* 0·7 billion younger than 5 years). However, the ratio between youth and young child mortality is changing. Youth mortality reduced by only 34% from 1990 to 2019, compared with a 59% decline in under-5 mortality. The annual rate of reduction in youth mortality showed an insignificant increase between the periods 1990–2000 and 2000–2019, whereas under-5 mortality rate showed a significant acceleration across these periods. In the future, a growing number of countries will see youth mortality exceeding under-5 mortality if current trends continue. Globally, the risk of death between age 15 years and age 25 years is already higher than the risk of death between the first and the fifth birthday.

This shift in age patterns of mortality needs to be interpreted with reference to changes in the relative contribution of underlying causes of death. During the epidemiological transition, the burden of infectious diseases declines faster than the burden associated with other causes of death. Because deaths in young children are predominantly caused by infectious diseases and neonatal conditions, progress in reducing mortality is more rapid in young children than in older children and youth. WHO and the Maternal Child Epidemiology Estimation Group regularly estimate causes of death in children younger than 5 years. They plan to extend the perspective to children and adolescents younger than 20 years, using the estimates presented in this study as mortality envelopes. The Global Burden of Disease (GBD) Study also provides detailed information on causes of death in youth and highlights the contribution of injuries to numbers of deaths. The leading causes of death in the group 15–24 years in 2019 were self-harm and interpersonal violence, transport injuries, unintentional injuries, respiratory infections and tuberculosis, neoplasms, cardiovascular diseases, and enteric infections.^19^ A large fraction of these deaths remains preventable. As highlighted in the Global Accelerated Action for the Health of Adolescents framework,^20^ many interventions have proven effective in improving adolescent health outcomes and ultimately lowering mortality risks. These interventions include comprehensive sex education, routine vaccinations, prevention of suicides, and the promotion of healthy behaviours. These interventions should be scaled up to accelerate the mortality reduction and keep pace with progress in child survival.

Our study was limited by the focus on mortality rates for both sexes combined. To generate sex-specific estimates, additional statistical modelling would be required to account for standard errors in the sex ratios, gender differentials in death registration completeness, and data quality issues. There is also room for improvement in the estimates for both sexes combined. Alternative modelling approaches could be explored, such as imposing an age structure and jointly modelling mortality rates by age intervals from birth to age 25 years. Methodological developments are needed to make better use of the information contained in vital statistics that are only partially complete. Data systems also need to be further developed in countries with incomplete death registration. Censuses should systematically include a module on household deaths in the past 12 months, and survey programmes should repeatedly include sibling survival histories. Finally, our study was restricted to the period 1990–2019; future iterations of this work will need to factor in the effect of the COVID-19 pandemic on youth mortality. Although the available evidence indicates that adolescents and young people account for a small percentage of COVID-related deaths,^21^ excess mortality could be caused by reductions in access to essential health services and disruptions in immunisation campaigns,^22^ the effects of quarantine and social distancing measures on mental health,^23^ and the looming economic crisis.

Despite the remaining data gaps and limitations, this study shows that there is a sufficient evidence base to estimate youth mortality from empirical measurements in most countries. Because of differences in underlying data and modelling approaches, our estimates contrast with those of the WPP^2^ and GBD 2019.^7^ The difference between the GBD 2019 estimate of the probability _10_q_15_ and our estimate for 2019 was more than 20% in 95 countries.^7^ The difference was more than 20% in 74 countries when comparing our study with the WPP.^2^ Globally, our estimates fall between those two other series: the number of deaths among youths aged 15–24 years in the GBD 2019 survey is 12% lower than the estimate from this study, and the WPP estimate is 13% higher than ours. The most striking discrepancies are in sub-Saharan Africa (appendix pp 161–163). In west Africa, the GBD 2019 estimates of deaths among youth aged 15–24 years are almost three times lower than those of the WPP, and in central Africa, they are two times lower than ours. Given the wide range spanned by these different estimates, a concerted effort to reconcile estimates is necessary to enable robust monitoring of health targets. Youth mortality is not a specific indicator of the 2030 Agenda for Sustainable Development, but accelerating the reduction in youth mortality will be required to meet many of the targets in Sustainable Development Goal 3 (eg, premature mortality from non-communicable diseases and deaths from road traffic accidents). Mortality in adolescents aged 10–19 years has also been retained as a key indicator of the Global Strategy for Women's, Children's and Adolescents' Health (2016–2030).

Robust monitoring of youth mortality remains challenging because the burden is concentrated in countries with deficient civil registration and vital statistics systems. In 2019, only 20·3% of deaths among youth occurred in countries where death registration was at least 80% complete. 25·9% of deaths occurred in China, Bangladesh, and India, where sample registration systems are in place, and for the remaining 53·9% of deaths, surveys and censuses were the primary sources of information. The global health community should recognise the urgent need to ensure that all deaths in youth are counted in functioning civil registration systems.

## Data sharing

All input data from surveys, censuses, and vital registration are publicly available in the public domain with final estimates, where links are provided to additional explanatory notes. The Bayesian B-spline bias-reduction model code is available upon request to the corresponding author.
