# Supplementary material for: Global, regional, and national mortality trends in youth aged 15–24 years between 1990 and 2019: a systematic analysis
Source: Lancet Glob Health. 2021 Mar 1;9(4):e409–17. doi: 10.1016/S2214-109X(21)00023-1 (PMC7966666; doi:10.1016/S2214-109X(21)00023-1)
Supplement: Supplementary appendix [file mmc1.pdf]

THE LANCET
Global Health

Supplementary appendix

This appendix formed part of the original submission and has been peer reviewed. We post it as supplied by the authors.

Supplement to: Masquelier B, Hug L, Sharrow D, et al. Global, regional, and national mortality trends in youth aged 15–24 years between 1990 and 2019: a systematic analysis. *Lancet Glob Health* 2021; published online March 1. http://dx.doi.org/10.1016/S2214-109X(21)00023-1.

Appendix

Global, regional, and national mortality trends in youth (15-24 years) from 1990 to 2019: an analysis of empirical data

Contents

1 Database **2**
1.1 Vital registration data 2
1.1.1 Data availability 2
1.1.2 Estimation of completeness 2
1.1.3 Calculation of mortality rates 8
1.2 Sibling survival histories 9
1.2.1 Calculation of mortality rates 9
1.2.2 Data exclusion and estimation of recall bias 10
1.3 Recent household deaths in censuses and surveys 13

2 Estimation model **13**
2.1 Statistical model 13
2.2 Bayesian penalized splines regression 13
2.3 Data model 14
2.4 Extrapolations 16
2.5 Computation 16
2.6 Adjustments for crisis-related mortality 18
2.7 Model validation 21
2.8 Deriving estimates for countries with insufficient data 25
2.9 Calculation of the annual rate of reduction 27

3 List of data series **27**

4 Mortality levels and numbers of deaths in 1990 and 2019 **43**

5 Country-specific trends in the probability $_{10}q_{15}$ **63**

6 Comparison with estimates from model life tables, the World Population Prospects 2019 and the Global Burden of Disease 2019 **161**

7 UNICEF regional classification **164**

8 United Nations Inter-Agency Group for Child Mortality Estimation **165**

1 Database

Data sources for the mortality rates were nationwide vital registration and sample registration systems, nationally representative sibling histories from surveys, and recent household deaths reported in censuses. Mortality rates were recalculated from the survey microdata when available or based on published tabulations.

1.1 Vital registration data

1.1.1 Data availability

Vital registration data were extracted from the WHO mortality database[1]. For 138 countries, death registration data were available for at least one year since 1990 [2]. Mortality rates for the period before 1989 were not included in the statistical model as our focus is on trends in the period 1990-2019.

1.1.2 Estimation of completeness

We assumed that death registration was complete in 38 countries included in the Human Mortality Database (HMD). For the other countries, we evaluated the completeness based on "death distribution methods" (DDMs). These methods compare the age distribution of deaths in the VR with the age distribution of the population enumerated in censuses to estimate the fraction of deaths that are reported. Each method has a standard version that applies to one census only but assumes a stable population. The methods have subsequently been revised for use in non-stable populations [1]. Three methods are applicable when two censuses are available, and deaths have been recorded in the intercensal period: the generalized growth balance method (GGB) [2], the synthetic extinct generation method (SEG) [3], and a combination of the two, called the hybrid method (or GGB>SEG). We employed here the GGB>SEG approach, which has been shown to perform better than the GGB and SEG methods used separately [4].

All the DDMs make strong assumptions, that under-reporting of deaths is constant by age (over a certain age limit), that age reporting is accurate, and that the population is closed to migration (unless net migration rates are available by age and sex). To reduce the sensitivity of estimates to violations of these assumptions, demographers usually select an age interval to calculate completeness, eliminating the youngest and oldest age groups to avoid introducing errors due to small numbers of deaths, age misreporting or net migration. Based on simulations, Murray et al. (2010) recommend using the age group 40-70 for the GGB, 55-80 for the SEG, 50-70 for the GGB>SEG [5]. Measuring completeness from adults over the age of 40 (or 50 or 55) reduces potential biases introduced by migration, which is often more frequent in young adults. However, estimates based on older ages are

[1] https://www.who.int/healthinfo/mortality_data/en/

[2] The WHO Mortality Database also contains some estimates from recent household deaths in censuses or intercensal surveys, but these were not treated as vital registration data and are not considered in this count.

more likely to be affected by age reporting errors. In another study, Hill and colleagues (2009) suggested using the age groups 5+ to 65+ for fitting purposes [4]. The selection of age groups remains an area requiring further research. Depending on the frequency of age errors and the extent of migration, various age groups should probably be selected for different methods and/or applications. In this study, the age range was selected based on visual inspection of the diagnostic plots. For example, in the case of the GGB method, it is usually recommended to plot $b(x+)$, the partial birth rate, against $d(x+)$, the partial death rate, for each age x. A regression line is fitted on these points, and the analyst examines the fit and residuals to select the points that provide the best fit [1]. The DDM package of R [6] replicates this manual approach by retaining the points that minimize the square of the average squared residual. We inspected all diagnostic plots, and in most cases, retained the age range that had been selected by the DDM package. In a few countries with substantial migration, or when completeness estimates were highly sensitive to the selection of age groups, we preferred to opt for older age groups, often selecting the age range 50-70 as recommended by Murray et al. (2010).

Completeness was estimated for each country for periods between pairs of recent censuses for which an age distribution of the population was available in the Demographic Yearbook[3]. When the estimated completeness was less than 80%, mortality rates derived from vital registration data were excluded from the model fit. When completeness was greater than or equal to 95%, the registration was considered complete, and mortality rates were not adjusted upwards. If completeness was between 80 and 95%, we multiplied the inverse of the completeness rate by the number of deaths to obtain adjusted estimates.

We did not introduce any smoothing of completeness levels, and changed the level from one intercensal period to the next. Mortality trends are smoothed in a second step using the B3 model (see below). After the last census, it is not possible to estimate completeness with death distribution methods, so completeness levels were kept constant until 2019.

Table S1 below presents the intercensal periods considered, the completeness estimates obtained with the GGB>SEG method and the age groups considered for the fit. In 103 countries, vital registration data could be used for at least one year after 2010. This number is slightly larger than that for under-five mortality (94 countries). The completeness of death registration could be lower among children, due to common under-reporting of neonatal deaths [7]. Countries with at least one usable vital registration data point after 2010 represented 29% of the global population of adolescents and young people, but only 20% of deaths in this age group.

Our estimates of completeness are compared in Table S1 with those of two other sources:

- First, as part of the *Global Burden of Disease* Study, the Institute for Health Metrics and Evaluation (IHME) regularly estimates the completeness of death registration also based on DDMs. The GBD 2017 study combined estimates derived from the GGB, SEG and GGB>SEG methods in a statistical model predicting completeness

[3] https://unstats.un.org/unsd/demographic-social/products/dyb/dybcensusdata.cshtml

in adults from that of the registration of under-five deaths[4]. Estimates were smoothed in time, as well as in space, and some information on completeness was also borrowed from neighbouring countries [8]. Completeness of the registration of under-five deaths was calculated from the ratio of the observed mortality rate from the vital registration to the final GBD estimate of child mortality. This method introduces a dependence on child mortality estimates and completeness of registration in children that we wish to avoid here. To our knowledge, the interrelationships between the completeness of child and adult registration have not been well studied. In some countries, under-registration of child deaths could occur, for example, because of difficulties in registering neonatal deaths, whereas registration of adults could be more systematic. For example, formerly Soviet Republics used a definition of live birth that excluded a substantial proportion of early neonatal deaths [9], and this will affect the completeness of child registration, but not that of deaths among older children and adults. We also wish to avoid borrowing information from neighbouring countries, as situations can vary widely within regions. For instance, according to GBD 2017 estimates, the completeness of the registration of adult deaths in Honduras is only 28%, while registration is considered complete in Guatemala and Nicaragua[5].

- The second set of completeness estimates comes from the United Nations Statistics Division (UNSD). The UNSD provides a recent estimate for most countries[6], but no trend over time and a variety of sources are involved (including workshops with national representatives, reports from National Statistical Offices and estimates from the World Health Organization).

In most countries, our estimates based solely on the GGB>SEG method are consistent the GBD estimates (derived from more involved modelling) (Figure S1). The mean absolute percentage error (MAPE) between our estimates and the GBD 2017 estimates is 5.4% and the concordance correlation coefficient is 0.79 (0.71-0.84) [10]. Comparison with UNSD estimates is more difficult because the UNSD values refer only to the most recent estimate available for each country, and for many countries, coverage is estimated in a range rather than a precise value. Several cases deserve further attention as estimates based on death distribution methods are quite higher than those provided by UNSD (e.g. Colombia, Guyana, Jordan, Trinidad and Tobago, Tunisia).

---

[4]At the time we analyzed the completeness of death registration, the GBD 2019 Study was not available. More recent completeness estimates from the GBD are now available at https://vizhub.healthdata.org/mortality/completeness.

[5]GBD estimates of completeness were retrieved from https://vizhub.healthdata.org/mortality/completeness, accessed July 2020, 25

[6]https://unstats.un.org/unsd/demographic-social/crvs/,accessedJuly2020,25

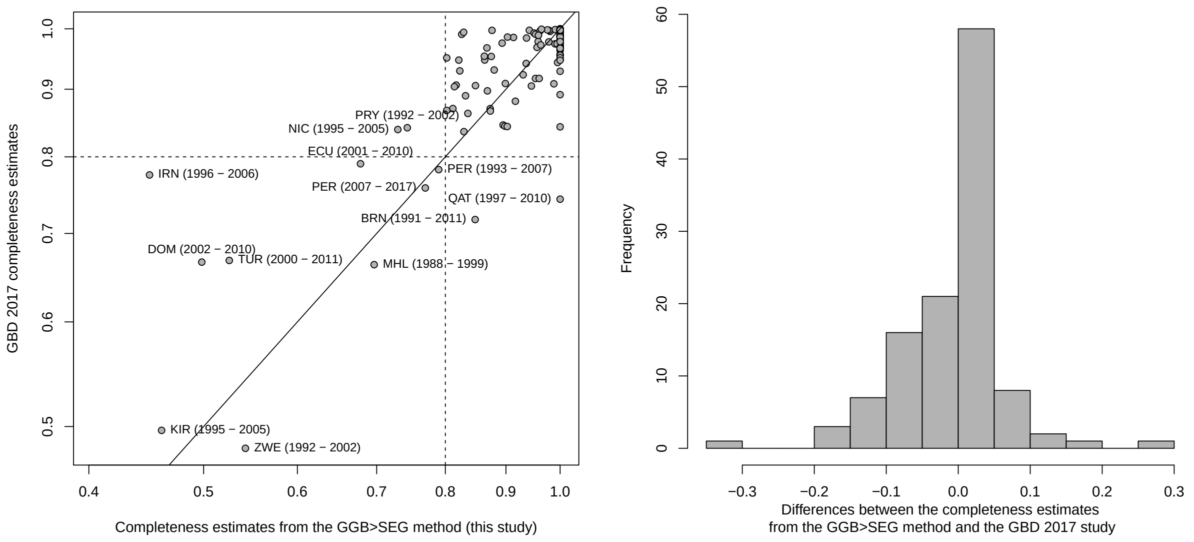

Figure S1 – *Concordance between the GBD 2017 estimates of completeness and estimates obtained from the GGB>SEG method in this study*

Country	First census	Second census	Age group	GGB>SEG	GBD 2017	UNSD
Albania	1989	2001	35-70	1.00	0.96	75-89% (2016)
Albania	2001	2011	40-75	1.00	0.89	75-89% (2016)
Algeria	1998	2008	5-75	1.00	0.97	less than 90% (2001)
Antigua and Barbuda	2001	2011	15-75	1.00	0.95	90% or more (2000)
Argentina	1991	2001	15-50	1.00	1.00	90% or more (2008)
Argentina	2001	2010	15-55	0.98	1.00	90% or more (2008)
Armenia	1989	2001	40-75	1.00	0.96	0.971 (2011)
Armenia	2001	2011	40-75	1.00	1.00	0.971 (2011)
Azerbaijan	1989	1999	50-70	0.82	0.91	90% or more (2001)
Azerbaijan	1999	2009	20-55	0.90	0.85	90% or more (2001)
Bahamas	1990	2000	5-65	0.81	0.90	90% or more (2003)
Bahamas	2000	2010	50-70	0.85	0.91	90% or more (2003)
Bahrain	1991	2001	10-65	0.81	0.87	90% or more (2015)
Bahrain	2001	2010	35-75	0.88	0.93	90% or more (2015)
Barbados	2000	2010	40-75	0.98	0.98	100% (2007)
Belize	1991	2000	25-60	0.94	0.94	100% (2008)
Belize	2000	2010	25-60	1.00	0.97	100% (2008)
Brazil	1991	2000	20-65	0.96	0.98	90-99% (2017)
Brazil	2000	2010	20-60	0.94	1.00	90-99% (2017)
Brunei Darussalam	1991	2011	5-65	0.85	0.72	100% (2017)
Cabo Verde	1990	2000	5-65	1.00	0.97	75% or more (2010-2015)
Cabo Verde	2000	2010	5-65	1.00	0.97	75% or more (2010-2015)
Colombia	1993	2005	5-65	0.99	1.00	0.729 (2000)
Cook Islands	1996	2006	20-55	1.00		82.0% (2012)
Costa Rica	1984	2000	10-65	0.95	0.99	0.97 (2016)
Costa Rica	2000	2011	10-65	1.00	0.99	0.97 (2016)
Cuba	1981	2002	30-70	0.99	0.97	100% (2017)

Country	First census	Second census	Age group	GGB>SEG	GBD 2017	UNSD
Cuba	2002	2012	25-60	1.00	0.98	100% (2017)
Cyprus	1992	2001	15-55	0.95	0.92	90% or more (2014)
Cyprus	2001	2011	40-75	0.90	0.84	90% or more (2014)
Dominica	1991	2001	25-60	1.00	1.00	90% or more (2000)
Dominica	2001	2011	40-75	1.00	1.00	90% or more (2000)
Dominican Republic	2002	2010	15-50	0.50	0.67	50-74% (2016)
Ecuador	1990	2001	30-65	1.00	1.00	70-79% (2017)
Ecuador	2001	2010	15-50	0.68	0.79	70-79% (2017)
Egypt	1996	2006	20-55	1.00	1.00	0.96 (2016)
Egypt	2006	2017	20-65	1.00	1.00	0.96 (2016)
El Salvador	1992	2007	35-75	0.96	0.92	80-89% (2017)
Fiji	2007	2017	20-55	1.00	0.97	100% (2010)
Georgia	1989	2002	5-55	0.87	0.90	90% or more (2000)
Georgia	2002	2014	20-65	1.00	0.98	90% or more (2000)
Grenada	1981	2001	30-65	0.83	0.99	100% (2009)
Grenada	2001	2011	35-65	0.98	1.00	100% (2009)
Guatemala	1981	2018	30-65	1.00	0.99	0.9 (2006)
Guyana	1991	2002	20-70	1.00	0.97	0.73 (2008)
Guyana	2002	2012	35-70	1.00	0.96	0.73 (2008)
Iran (Islamic Republic of)	1996	2006	15-50	0.45	0.78	0.92 (2015)
Iran (Islamic Republic of)	2006	2011	30-65	0.83	0.84	0.92 (2015)
Jamaica	1991	2001	45-70	0.86	0.95	75-89% (2008)
Jamaica	2001	2011	35-70	0.99	0.97	75-89% (2008)
Jordan	2004	2015	25-65	0.83	0.89	0.65 (2017)
Kazakhstan	1999	2009	40-75	1.00	0.95	90% or more (2001)
Kiribati	1995	2005	15-50	0.46	0.50	76.0% (2002)
Kuwait	1995	2005	35-60	1.00	1.00	90% or more (2001)
Kyrgyzstan	1989	1999	5-75	0.95	0.91	90-99% (2017)
Kyrgyzstan	1999	2009	5-75	1.00	0.94	90-99% (2017)
Malaysia	1991	2000	50-70	1.00	0.99	90-99% (2017)
Malaysia	2000	2010	15-55	1.00	1.00	90-99% (2017)
Maldives	1985	1995	25-60	1.00	0.97	100% (2014)
Maldives	1995	2006	20-65	0.95	0.99	100% (2014)
Malta	1995	2005	15-50	0.96	0.97	90% or more (2016)
Marshall Islands	1988	1999	15-50	0.70	0.66	90% or more (2001)
Mauritius	1990	2000	30-65	1.00	0.98	90% or more (2013)
Mauritius	2000	2011	40-75	1.00	0.99	90% or more (2013)
Mexico	1990	2000	30-65	1.00	0.99	90-99% (2017)
Mexico	2000	2010	30-70	1.00	0.99	90-99% (2017)
Mongolia	1989	2000	20-65	0.80	0.87	90-99% (2017)
Mongolia	2000	2010	35-70	0.90	0.91	90-99% (2017)
Montenegro	1991	2003	25-60	1.00	0.95	90% or more (2006)
Montenegro	2003	2011	15-60	1.00	0.95	90% or more (2006)
Morocco	1994	2004	15-50	0.40		0.6236 (2007)
Nicaragua	1995	2005	50-70	0.73	0.84	0.699 (2009)
North Macedonia	1991	2002	5-60	0.96	0.97	90% or more (2016)
Panama	1990	2000	15-55	0.87	0.97	0.937 (2014)
Panama	2000	2010	25-60	0.89	0.98	0.937 (2014)
Paraguay	1992	2002	15-50	0.74	0.84	0.65 (2013)
Peru	1993	2007	5-75	0.79	0.78	50-74% (2015)
Peru	2007	2017	5-65	0.77	0.76	50-74% (2015)

Country	First census	Second census	Age group	GGB>SEG	GBD 2017	UNSD
Philippines	1990	2000	5-70	1.00	0.93	0.876 (2015)
Philippines	2000	2015	15-60	0.99	0.91	0.876 (2015)
Qatar	1997	2010	50-65	1.00	0.74	1 (2017)
Republic of Korea	1985	1995	15-50	0.83	0.99	100% (2017)
Republic of Korea	1995	2005	15-50	1.00	0.98	100% (2017)
Republic of Korea	2005	2015	15-50	0.82	0.95	100% (2017)
Republic of Moldova	1989	2004	15-55	1.00	0.99	90% or more (2010)
Republic of Moldova	2004	2014	40-75	1.00	0.99	90% or more (2010)
Romania	1992	2002	30-65	1.00	0.99	90% or more (2016)
Romania	2002	2011	40-75	1.00	0.99	90% or more (2016)
Saint Kitts and Nevis	1991	2001	35-70	1.00		0.82 (2009)
Saint Lucia	1991	2001	35-70	1.00	0.99	0.92 (2010)
Saint Vincent and the Grenadines	1991	2001	40-75	1.00	1.00	90% or more (2014)
Saint Vincent and the Grenadines	2001	2012	35-70	1.00	1.00	90% or more (2014)
Serbia	1991	2002	30-65	1.00	1.00	90% or more (2016)
Serbia	2002	2011	25-70	1.00	1.00	90% or more (2016)
Seychelles	1994	2002	15-60	0.96	1.00	90% or more (2012)
Seychelles	2002	2010	15-55	0.98	1.00	90% or more (2012)
Singapore	1990	2000	25-70	0.80	0.95	90% or more (2012)
Singapore	2000	2010	35-70	0.87	0.95	90% or more (2012)
South Africa	1991	2001	20-60	0.92	0.88	75-89% (2008)
South Africa	2001	2011	20-55	0.93	0.92	75-89% (2008)
Sri Lanka	1981	2001	30-75	1.00	0.99	90% or more (2009)
Sri Lanka	2001	2012	15-55	0.94	0.98	90% or more (2009)
Suriname	1980	2004	15-65	0.87	0.87	90% or more (2016)
Suriname	2004	2012	20-55	0.90	0.84	90% or more (2016)
Tajikistan	1989	2000	30-70	0.82	0.93	75-89% (2007)
Tajikistan	2000	2010	5-70	1.00	0.84	75-89% (2007)
Thailand	1990	2000	50-75	0.84	0.86	85.0% (2012)
Thailand	2000	2010	50-70	0.86	0.95	85.0% (2012)
Tonga	1996	2006	50-70	1.00	0.97	70.0% (2002)
Tonga	2006	2016	50-70	1.00	0.98	70.0% (2002)
Trinidad and Tobago	1990	2000	35-70	1.00	1.00	0.86 (2007)
Trinidad and Tobago	2000	2011	30-65	0.96	1.00	0.86 (2007)
Tunisia	1994	2004	50-70	0.87	0.87	64.0% (2000)
Turkey	2000	2011	25-70	0.53	0.67	90% or more (2016)
Turkmenistan	1989	1995	50-70	0.91	0.99	
Uruguay	1996	2004	35-70	1.00	1.00	90% or more (2015)
Uruguay	2004	2011	40-75	1.00	1.00	90% or more (2015)
Uzbekistan	1979	1989	50-70	0.88	1.00	90% or more (2016)
Venezuela (Bolivarian Republic of)	1990	2001	15-50	0.96	0.99	90% or more (2016)
Venezuela (Bolivarian Republic of)	2001	2011	20-60	0.90	0.99	90% or more (2016)
Zimbabwe	1992	2002	15-50	0.54	0.48	

Table S1 – *Completeness of death registration from various sources for intercensal periods*
Note: GGB>SEG refers to our estimates for the population aged 5 and above, GBD to the completeness among adults in the GBD 2017, UNSD to the completeness for deaths of all ages. The GGB>SEG estimates refer to the intercensal period, GBD to the mid-point of the period, and reference years for UNSD are provided in the Table.

All estimates of completeness derived from death distribution methods remain ques-

tionable and should be compared with other studies, e.g. from capture-recapture studies [11]. Murray et al. (2010) demonstrate that uncertainty intervals around completeness estimates from DDM are quite large, roughly about 25% [5]. This is in part because death distribution methods are based on assumptions that are often poorly respected in practice. In some cases, we have preferred not to adjust upwards the deaths reported to vital statistics, either because of large migrations flows that could invalidate the method, or because published studies had investigated the completeness of death registration. These exceptions are as follows:

- Brazil: our estimate of completeness based on the GGB>SEG method was 0.96 for the period 1991-2000 and 0.94 for the period 2000-2010. Both the SEG and GGB methods taken separately are higher than 95% for this second period. Li and Gerland (2019) [12] use various death distribution methods and also obtain completeness estimates above 95% after 2000. Hence we did not adjust the vital registration estimates upwards for Brazil.

- Republic of Korea: estimates of completeness obtained from DDMs applied to the WHO Mortality Database are irregular and inconsistent with GBD estimates that place the completeness above 95% after 1990. Based on the study by Hill and colleagues (2005) [13], we assumed that death registration was complete after 1990.

- Syria: It was not possible to use DDMs to estimate completeness as the VR data currently available only cover the years 1983, 1984 and 1998-2010, and the censuses were conducted in 1994 and 2004. VR estimates are currently used without adjustment after 1998.

- Turkey: We cannot estimate completeness after 2010 with DDMs as the last census was conducted in 2011. Özdemir and colleagues (2015) [14] provide estimates for the period 2009-2013 based on population data from TURKSTAT Address Based Population Registration System (ABPRS). They estimate that in the period 2009-2013, death registration was higher than 95% for both sexes. Prior to that, death registration was lower than 80%. In this study, we used the VR data only from 2010.

- Singapore, Bahrain, Sri Lanka: we assumed that the vital registration was complete in these three countries, even though death distribution methods suggest otherwise, most likely due to the perturbing role played by international migrations. In Sri Lanka, the civil war could have introduced disruptions in both the numerators and denominators, such as changes in coverage of the population enumerated in the censuses.

1.1.3 Calculation of mortality rates

The calculation of the probability ${}_{10}q_{15}$ was derived from a standard period abridged life table. The inputs were the number of deaths for age groups 15-19 (noted ${}_5D_{15}$) and for the age group 20-24 (${}_5D_{20}$), as well as the mid-year population for the same age groups (${}_5P_{15}$ and ${}_5P_{20}$).

- The death rate for age group 15-19, ${}_5M_{15}$ was obtained by dividing ${}_5D_{15}$ by ${}_5P_{15}$.
- The probability ${}_5q_{15}$, which is the risk of dying between age 15 and age 20, was obtained as ${}_5q_{15} = (5 \times {}_5M_{15})/[1 + (5 - {}_5a_{15}) \times {}_5M_{15}]$, where ${}_5a_{15}$ is the average number of years lived by adolescents who died in the age group 15-19 - set at 2.5 for all countries for this study.
- The same calculation was applied for ${}_5q_{20}$.
- The probability ${}_{10}q_{15}$ was computed as ${}_{10}q_{15} = 1000 \times (1 - (1 - {}_5q_{15}) \times (1 - {}_5q_{20}))$.

The numbers of deaths and mid-year populations refer to both sexes. Mortality rates were recalculated by pooling deaths and population from successive years together such that the coefficient of variation (the ratio of the standard deviation to the mean) is less than 20%. The stochastic standard errors of the mortality rates were calculated using a Poisson approximation, based on the number of children turning 15 in each year, estimated from the 2019 World Population Prospects [15].

1.2 Sibling survival histories

1.2.1 Calculation of mortality rates

Mortality estimates in adolescents and young people aged 15-24 can be derived from sibling survival histories (SSH). In SSH, women aged 15-49 years are asked to list all their siblings born to the same mother by birth order and to report on their gender, survival status, current age when alive, or age at death and number of years since death when deceased. SSH have been included in about 150 Demographic and Health Surveys (DHS)[7], about 10 Multiple Indicator Cluster Surveys (MICS)[8] and about 40 World Health Surveys[9].

In this study, SSH were used to estimate the probability of dying in youth (${}_{10}q_{15}$) for a period of 0-12 years prior to each survey. This period was divided in intervals of various length (6, 4, 3, 2, 1 years) depending on the coefficient of the variation of the estimates. The original data sets, which have one row for each woman aged 15-49, were reshaped into files containing one row for each sibling. Based on the dates of birth and death (imputed by DHS), each life course was split into different spells; a new spell was created at every birthday and every time the number of completed years preceding the survey changed. These spells were then aggregated to form a new dataset in which each row corresponds to a person-period (a unique combination of a given age group, sex, and time preceding the survey). For each person-period, the exposure time and the number of deaths were summed and weighted by the sample weights. Standard errors were obtained using a Jackknife variance estimation method [16]. Age-specific mortality rates were converted

[7] https://dhsprogram.com/

[8] https://mics.unicef.org/ - Some MICS surveys also include a summary sibling history with information on the number of adult sisters and the number of these sisters who have died, without information on ages at death.

[9] https://apps.who.int/healthinfo/systems/surveydata/index.php/catalog/whs/about

into probabilities ${}_5q_{15}$ and ${}_5q_{20}$ and these probabilities were chained together to form the probability ${}_{10}q_{15}$.

There is a debate in the literature about the presence of selection bias in sibling survival histories because larger sibships are over-represented in the sample, some sibships with no surviving members are not apparent in the data and the respondent is alive and reports on a group which she belongs to [17]. It has been demonstrated, however, that these selection biases cancel each other out when adult mortality is not associated with the number of adult siblings [18, 19]. We assumed here that this was the case and used the standard DHS approach to obtain mortality rates (excluding the respondent when computing person-years of exposure).

1.2.2 Data exclusion and estimation of recall bias

Sibling survival data have been evaluated in validation studies [20, 21] and compared to census or model-based estimates [22, 23]. Other studies have examined the consistency of mortality rates across surveys [24, 25] and the plausibility of reported sibship sizes against past fertility estimates [26, 27]. This literature suggests that sibling histories could lead to underestimates of adult mortality due to under-reporting of deaths, especially for reference periods located more than 10 to 15 years before the survey. Under-reporting of deaths could be amplified or attenuated by systematic misstatement of ages and the timing of deaths. The magnitude of these biases likely varies by age but few studies have assessed biases in the 15-24 age group specifically. In a validation study in a Health and Demographic Surveillance System (HDSS) in Senegal, Helleringer and colleagues (2014) observed that sibling histories led to an underestimation of mortality only from the age of 50 onwards [20]. In younger age groups, sibling survival estimates were consistent with mortality levels in the HDSS. Biases affecting sibling data may, therefore, be limited when estimating youth mortality but additional validation studies are needed to reach firmer conclusions. To account for the possibility of bias, we proceeded in two steps. First, we excluded surveys that presented implausible age patterns of mortality and second, we modelled reporting bias in the surveys that were retained.

First, we examined age patterns of mortality contained in surveys against a standard based on high-quality mortality data. This standard was built from the following sources:

- The 5x5 life tables from the Human Mortality Database [28], representing the historical experience of 41 countries or areas with long statistical tradition of accurate vital registration;
- Life tables recalculated for periods of 2 or 3 years from publicly-available datasets from 48 Health and Demographic Surveillance Systems (HDSS)[10];

[10] Life tables from the following HDSS were retained: Chakaria and Matlab (ICDDR,B Area) in Bangladesh; Nanoro, Nouna, Ouagadougou and Oubritenga in Burkina Faso; Taabo in Côte d'Ivoire; Arba Minch, Butajira, Dabat, Gilgel Gibe, Harar Urban, Kersa, Kilite Awlaelo in Ethiopia; Farafenni in The Gambia; Dodowa, Kintampo and Navrongo in Ghana; Bandim in Guinea-Bissau; Vadu in India; Kilifi, Kisumu/KEMRI-CDC, Kombewa, Mbita and Nairobi in Kenya; Karonga in Malawi; Chokwe and

- Life tables based on vital registration data from Mauritius and sample vital registration data from India (obtained from the UN Population Division).

The relationship between ${}_5q_0$ and ${}_{10}q_{15}$ in these various life tables is presented in Figure S2 below, using a log-log scale, alongside the relationship captured in historical model life tables (here the North and South models of Coale and Demeny life tables[11]).

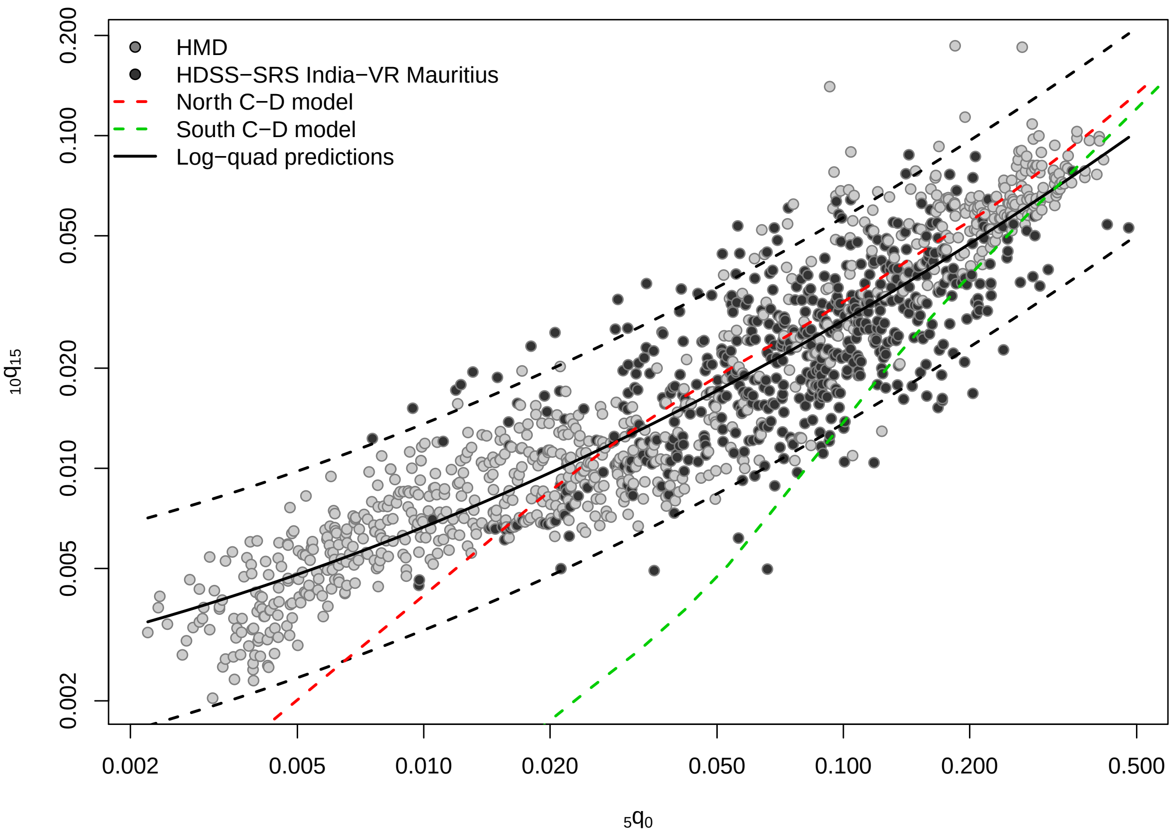

Figure S2 – *Relationship between ${}_5q_0$ and ${}_{10}q_{15}$ in the life tables from HMD, HDSS, SRS in India and VR in Mauritius.*
Note: the dotted lines in color refer to the ${}_{10}q_{15}$-to-${}_5q_0$ relationship in Coale-Demeny model life tables (revised by the United Nations to achieve life expectancies up to 100 years for population projections).

Manhica in Mozambique; Nahuche in Nigeria; Bandafassi, Mlomp and Niakhar in Senegal; Africa Centre, Agincourt and Dikgale in South Africa; Ifakara Rural, Magu and Rufiji in Tanzania; Iganga/Mayuge and Rakai in Uganda; Dar es Salaam, Hai, Magu and Morogoro in Tanzania; Chililab and Nam FilaBavi in Viet Nam; Gwembe in Zambia; Manicaland in Zimbabwe.

[11]United Nations Population Division, Extended model life tables, available at https://www.un.org/en/development/desa/population/publications/mortality/model-life-tables.asp, accessed June 04, 2020

From this database containing about 1300 data points, we used a log-quadratic regression model to predict, for a given level of under-five mortality, what would be the expected level of mortality between the ages of 15 and 25:

$$y_i = \big(\beta_0 + \beta_1 x_{i1} + \beta_2 x_{i2}\big) + \epsilon_i \tag{1}$$

where y_i is value of $log({}_{10}q_{15})$ for observation i, x_{i1} is the value of $log({}_5q_0)$ for observation i and x_{i2} equals $\big(log({}_5q_0)\big)^2$ for observation i. The independent errors terms ϵ_i are normally distributed. Prediction intervals around $log({}_{10}q_{15})$ for a new estimate of $log({}_5q_0)$ were be obtained as

$$\hat{y}_h \pm t_{(\alpha/2,n-p)} \times \sqrt{MSE + (\text{se}(\hat{y}_h))^2} \tag{2}$$

where $\hat{y}_h$ is the fitted value when the predictor values are $\mathbf{X}_h = (1, X_{h,1}, X_{h,2})^{\mathrm{T}}$, $t_{(\alpha/2,n-p)}$ is the t-multiplier and $\sqrt{MSE + (\text{se}(\hat{y}_h))^2}$ is the standard error of the prediction.

Turning to sample surveys, we extracted the probability ${}_5q_0$ from birth histories for the period 0-12 years prior to data collection and the probability ${}_{10}q_{15}$ from sibling histories for the same period. We used Eq.2 to obtain the 95% intervals around the predicted value of $log({}_{10}q_{15})$ for the observed value of $log({}_5q_0)$. We checked if these prediction intervals contained the observed value of $log({}_{10}q_{15})$. When they did not, the mortality pattern encompassed in survey estimates was deemed implausible, and all survey data points were excluded so that they did not inform the model fitting. This procedure assumes that birth histories are unbiased and that our standard adequately reflect the ${}_{10}q_{15}$-to-${}_5q_0$ relationship in all countries for which we have sample surveys.

This quality requirement led to the exclusion of 9 DHS surveys (out of 148)[12], 13 WHS surveys (out of 42)[13] and 3 MICS (out of 9)[14]. Judging from the proportions of surveys excluded from the model fit by type of series, sibling histories collected in the WHS and MICS surveys seem of lower quality than in DHS. Hence, biases in levels and trends were estimated from the B3 specifically for sibling histories from DHS on the one hand, and those from WHS and MICS on the other.

Finally, we modelled the bias in the remaining surveys, both in terms of the level and the trend, as a function of the retrospective period. This bias was estimated by contrasting the sibling-based estimates with vital registration data for overlapping time periods (see section 2.3 below).

---

[12]The following DHS were excluded: Bangladesh 2001, Indonesia 1997, Mali 1995-1996, Nigeria 1999, Morocco 1992 and 2003-2004, Peru 2009, 2010 and 2012.

[13]We excluded the following WHS: Bosnia & Herzegovina, Chad, China, Comoros, Congo, Estonia, Malaysia, Mauritania, Pakistan, Senegal, Slovakia, Ukraine, Uruguay. The implausible trends in mortality in estimates extracted from the WHS surveys conducted in Philippines and Mexico, as well as the very wide uncertainty intervals around estimates in Georgia and Russian Federation led us to exclude these four surveys.

[14]The following MICS were excluded: Bhutan 2010, Lao PDR 2012, Mauritania 2011.

1.3 Recent household deaths in censuses and surveys

Censuses often include questions on household deaths in the last 12 months, which can be used to calculate mortality estimates for the population aged 15-24. The calculation of the probability ${}_{10}q_{15}$ was derived from a standard period abridged life table. As for sibling histories, we checked the ${}_{10}q_{15}$-to-${}_{5}q_{0}$ relationship observed in the data against the predictions of the log-quadratic model above, and discarded estimates that fell outside of the 95% predictions intervals. This assessment led to the exclusion of about 29% of observations from censuses.

In most cases, we did not adjust for incompleteness of death reporting in surveys and censuses. However, in China, we used published estimates from census data from 1982 to 2000, adjusted for incompleteness of death reporting with the General Growth Balance method by Banister and Hill (2004) [29]. To be consistent with this study, we also evaluated the completeness of death reporting in the 2010 census with the General Growth Balance method [30].

2 Estimation model

2.1 Statistical model

To obtain estimates of the probability ${}_{10}q_{15}$ for all country-years based on all available data, we applied to the 15-24 age group the model used by the UN IGME to monitor trends in under-five mortality. This is a Bayesian penalized B-splines bias-reduction (B3) model. All details on B3 are available in Alkema and New (2014) [31]. We repeat below the main features of this model to highlight a few changes implemented for the 15-24 age group.

We let u_i denote the observed probability ${}_{10}q_{15}$ for the observation i in country $c[i]$ and year $t[i]$: $u_i = U_{c[i]}(t[i]) \cdot \varepsilon_i$, where $U_c(t)$ denotes the true probability ${}_{10}q_{15}$ and $\varepsilon_i > 0$ is the error multiplier. On the natural log-scale, this corresponds to

$$y_i = f_{c[i]}(t[i]) + \delta_i, \tag{3}$$

where $y_i = \log(u_i)$, $f_c(t) = \log(U_c(t))$, and $\delta_i = \log(\varepsilon_i)$.

2.2 Bayesian penalized splines regression

The regression spline model for $f_c(t)$ from Eq.(3) is given by:

$$f_c(t) \quad = \quad \sum_{k=1}^{K_c} B_{c,k}(t)\alpha_{c,k}, \tag{4}$$

where $\alpha_{c,k}$ refers to splines coefficient k in country c and $B_{c,k}(t)$ the k-th spline, evaluated in year t, given by a third order B-spline [32, 33]. Equally spaced knots were used such that the resulting splines are non-zero for a total of $4 \cdot I$ years, where I refers to the in-between

knots interval length. We chose $I = 2.5$ years such that each spline is non-zero for 10 years. In each country, the knots were placed such that the largest two splines $B_{c,K_c-2}(t)$ and $B_{c,K_c-1}(t)$ in the most recent observation year $t = t_{n_c}$ have equal height while $B_{c,K_c}(t)$ is close to zero.

When fitting the splines model from Eq.(4) to the observations, second-order differences in adjacent spline coefficients ($\Delta^2\alpha_k = \alpha_k - 2\alpha_{k-1} + \alpha_{k-2}$) are penalized to guarantee smoothness of the resulting ${}_{10}q_{15}$ trajectory and to project forward the recently observed rate of change past the most recent data. Let $\boldsymbol{t}_c = (t_{c,1}, \ldots, t_{c,n_c})'$ refer to the vector of country-specific observation years with spline model estimate $f_c(\boldsymbol{t}_c) = (f(t_{c,1}), \ldots, f(t_{c,n_c}))'$.

The splines model $f_c(\boldsymbol{t}_c) = \boldsymbol{B}_c(\boldsymbol{t}_c)\boldsymbol{\alpha}_c$, with $\boldsymbol{B}_c(\boldsymbol{t}_c) = (B_{c,1}(\boldsymbol{t}_c), \ldots, B_{c,K_c}(\boldsymbol{t}_c))$, $\boldsymbol{\alpha}_c = (\alpha_{c,1}, \ldots, \alpha_{c,K_c})'$, can be written as follows [34, 33]:

$$\begin{aligned} f_c(\boldsymbol{t}_c) &= \boldsymbol{B}_c(\boldsymbol{t}_c)\boldsymbol{G}_{K_c}\boldsymbol{b}_c + \boldsymbol{Z}_c(\boldsymbol{t}_c)\boldsymbol{e}_c, \\ \boldsymbol{G}_{K_c} &= (\boldsymbol{1}_{K_c}, \boldsymbol{g}_{K_c})', \text{ where } \boldsymbol{g}_{K_c} = (1 - K_c/2, \ldots, K_c - K_c/2)', \\ \boldsymbol{Z}_c(\boldsymbol{t}_c) &= \boldsymbol{B}_c(\boldsymbol{t}_c)\boldsymbol{D}'_{K_c}(\boldsymbol{D}_{K_c}\boldsymbol{D}'_{K_c})^{-1}, \end{aligned} \tag{5}$$

where in difference matrix $\boldsymbol{D}_{K_c}$, $D_{K_c,i,i} = D_{K_c,i,i+2} = 1$, $D_{K_c,i,i+1} = -2$ and $D_{K_c,i,j} = 0$ otherwise.

The first part in Eq.(5), $\boldsymbol{B}_c(\boldsymbol{t}_c)\boldsymbol{G}_{K_c}\boldsymbol{b}_c$, describes the linear trend during the observation period, and the second part $\boldsymbol{Z}_c(\boldsymbol{t}_c)\boldsymbol{e}_c$ describes the fluctuations around the main trend. The unknown parameters are given by $\boldsymbol{b}_c = (b_{c,0}, b_{c,1})'$, and $\boldsymbol{e}_c = \boldsymbol{D}_{K_c}\boldsymbol{\alpha}_c$, where $\boldsymbol{e}_c = (e_{c,1}, \ldots, e_{c,Q_c})'$, with $Q_c = K_c - 2$ and $e_{c,q} = \Delta^2\alpha_{c,q+2}$ for $q = 1, \ldots, Q_c$. Second-order differences are penalized by imposing

$$e_{c,q} \sim N(0, \sigma_c^2), \text{ for } q = 1, \ldots, Q_c, \tag{6}$$

where variance σ_c^2 determines the extent of smoothing; a smaller variance corresponds to smoother trajectories. In the limit when σ_c decreases to zero, a linear fit for $\log({}_{10}q_{15})$ is obtained.

The model is fit in the Bayesian framework. No information on levels or trends during the observation period is exchanged across countries when estimating the spline coefficients. Information exchanged across countries only concerns the variability of the (second order) difference in the spline coefficients through a multilevel model. The variance of $e_{c,q} = \alpha_{c,q+2}$ - $2\alpha_{c,q+1} + \alpha_{c,q}$ is estimated hierarchically:

$$\log(\sigma_c) \sim N(\chi, \varphi_\sigma^2). \tag{7}$$

Vague prior distributions are used for the $\boldsymbol{b}_c$'s and the hyper parameters for the hierarchical model for the σ_c's [31].

2.3 Data model

The error distribution for observations from complete VR or SVR is given by

$$\delta_i \sim N(0, \tau_i^2/u_i^2),$$

where τ_i/u_i is the stochastic standard error. These errors are calculated using a Poisson approximation (using the numbers of adolescents turning 15 from the World Population Prospects 2019) and set to a minimum of 2.5%. If the Poisson sampling standard error cannot be calculated (e.g. for Sample Registration Systems), it is set to 10%.

For non-VR data, δ_i, the error term on the log-scale, is specified as follows:

$$\delta_i \;=\; E_i + S_i \cdot X_i,$$

where E_i is the mean bias, S_i the scale parameter and X_i determines the distribution for observation i.

For all non-VR data series with repeated observations, mean biases were modeled as a linear function of the retrospective period of the observation in the survey:

$$E_i \;=\; \beta_{0,s[i]} + \beta_{1,s[i]} \cdot \pi_i, \tag{8}$$

where $\beta_{0,s[i]}+\beta_{1,s[i]}\cdot\pi_i$ represents the bias in level and trend as a function of the retrospective period π_i for observation i in data series $s[i]$. The retrospective period π_i was centered at 5 years for this study. The bias in the level of the series, $\beta_{0,s}$ is estimated with a multilevel model:

$$\beta_{0,s} \;\sim\; N(\mu_{0,d[s]}, \gamma^2_{0,d[s]}), \tag{9}$$

where $d[s]$ refers to the source type of series s, based on data source (the source types with multiple observations per series are given by DHS Direct and Others Direct (MICS and WHS surveys)), and $\mu_{0,d}$ and $\gamma^2_{0,d}$ represent source type-specific mean bias and between-series variance respectively. A similar approach is used to estimate the slope $\beta_{1,s}$:

$$\beta_{1,s} \;\sim\; N(\mu_{1,d[s]}, \gamma^2_{1,d[s]}), \tag{10}$$

where $\mu_{1,d}$ and $\gamma^2_{1,d}$ represent the mean slope and the between-series variance for source type d. For single observations constructed from reported household deaths, and single observations obtained from reported life tables, we assume that $E_i = \mu_{0,d[s[i]]}$.

Scale parameter S_i is modeled as a combination of sampling variance τ_i^2/u_i^2 (based on sampling variance τ_i^2 for ${}_{10}q_{15}$) and non-sampling variance $\omega^2_{d'[s[i]]}$:

$$S_i^2 \;=\; \omega^2_{d'[s[i]]} + \tau_i^2/u_i^2, \tag{11}$$

where source type $d'[s]$ for series s refers to a further breakdown of source types to distinguish between DHS, Other (MICS and WHS) and recent household deaths. Where the sampling variance for non-VR data is not reported, we assume a sampling standard error of 10%.

Finally, the distribution for δ_i is given by:

$$X_i \;\sim\; \begin{cases} N(0,1) & \text{for Standard and Other DHS direct,} \\ t_\nu & \text{otherwise, with } \nu \sim U(2,30). \end{cases} \tag{12}$$

A t-distribution with ν degrees of freedom is used for observations that are not obtained from Standard or Other DHS, in accordance with the model used by UN IGME for under-five mortality.

All model parameters in Eq.(8)–(12) were assigned vague prior distributions. In the UN IGME model for under-five mortality, an informative prior distribution was used for the mean bias $\mu_{0,d}$ for the DHS Direct series, but here we used vague prior distributions for all parameters.

Figure S3 presents mean biases and 90% prediction intervals for "new" data points by type of data source. This visualization shows that for a "true" level of the probability $_{10}q_{15}$ of 15 deaths before age 25 per 1000 adolescents at age 15, the mean bias will vary across data sources and with the length of the retrospective period (in the survey estimates). The prediction intervals based on uncertainty in the bias parameters only (dark colors) are large, indicating that there is substantial variability in biases across data series of the same source type. Mean biases tend to be negative in sample surveys and slightly positive for data on recent household deaths (represented in pink). The median of the predicted $_{10}q_{15}$ for a retrospective period of 2 years (our first estimate from DHS) is 14.9 per thousand (12.4-17.3). For a retrospective period of 10 years, it declines to 12.4 (10.3-14.6), corresponding to under-estimating the true level by about 17%. This is consistent with previous studies on sibling histories indicating that the quality of death reporting declines as the reference periods stretch back in the past [24, 25, 22].

2.4 Extrapolations

For extrapolations, we implemented a logarithmic pooling procedure to combine country-specific posterior predictive distributions for changes in spline coefficients with a global posterior predictive distribution. This procedure was applied to modify the posterior predictive distributions for $\alpha_{c,k}$ for $k = K_c, K_c + 1, \ldots, P_c$, where P_c refers to the last spline in the projection period of interest. While α_{c,K_c} was among the spline coefficients that were included in the observation period up to year t_{n_c}, it was included in the set of "projected" coefficients to be pooled because its estimate is based mainly on an extrapolation of past changes. The logarithmic pooling weight κ, which determines the extent of pooling, was set at 0.8 for this study, after examination of country plots for various weights and also based on validation outputs (see below). Further details on the logarithmic pooling procedure are provided in [31].

2.5 Computation

A Markov Chain Monte Carlo (MCMC) algorithm was employed to sample from the posterior distribution of the parameters in the global and country-specific models with the use of the software JAGS. For the global run, 10 parallel chains with different starting points were run with a total of 75,000 iterations in each chain. Of these, the first 25,000 iterations in each chain were discarded as burn-in and every 20th iteration after was retained. The resulting chains contained 2,500 samples each. For the country-specific runs, we ran 10

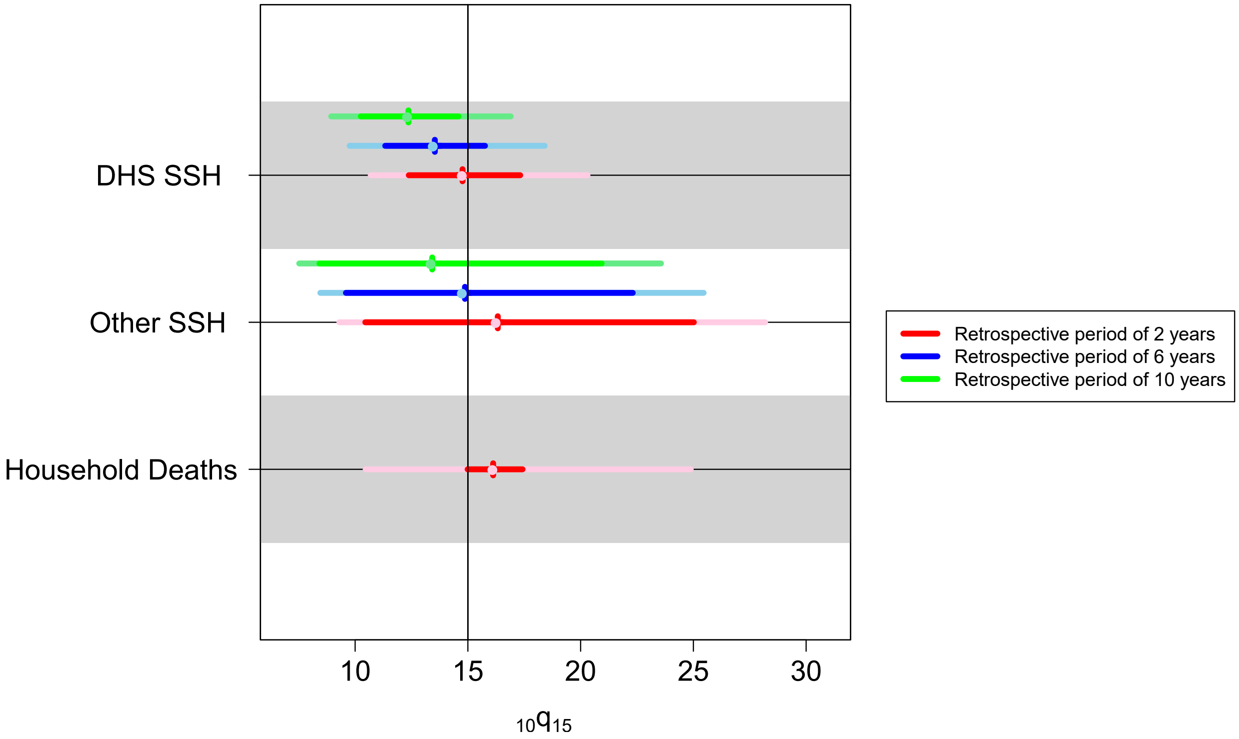

Figure S3 – *90% prediction intervals for new data points by source type and length of the retrospective period.*
Note: For a "true" probability $_{10}q_{15}$ *of 15 deaths per 1,000 adolescents aged 15 (represented by the vertical black line), the predicted mean observed* $_{10}q_{15}$ *is represented by the colored vertical lines. The difference between the mean* $_{10}q_{15}$ *and 15 represents the mean bias. The dark horizontal lines represent the 90% prediction intervals for an observation based on uncertainty in the bias parameters only (excluding sampling and non-sampling variability), while the 90% prediction intervals excluding the sampling variability are in light colors.*

chains with a total of 62,500 iterations in each chain. Of these, the first 25,000 iterations in each chain were discarded as burn-in and every 30th iteration after was retained. The resulting chains contained 1,250 samples each.

A computationally cheaper model was implemented to allow for updates of country-specific estimates with additional data without the need to re-run the global model. Non-country-specific parameters were fixed at the posterior medians from the global model run. Both models resulted in very similar estimates.

In some countries, there was insufficient smoothing, resulting in unrealistic short-term changes for a subset of country-years without VR data. For these countries, instead of using country-specific smoothing determined by variance parameter σ_c^2 from Eq.(7), we set $\sigma_c = \exp(\hat{\chi})$, where $\hat{\chi}$ refers to the posterior median of μ, which is referred to as the global smoothing level. This subset includes 1) countries with both VR and non-VR data, or both adjusted and non-adjusted VR data, 2) countries that have VR data with gaps more than 5 years in the data, as well as 3) small countries with less than 15,000 adolescents aged 15 in 2019 (based on the World Population Prospects 2019).

2.6 Adjustments for crisis-related mortality

Adjustments were made to account for abrupt increases in mortality due to conflicts and/or disasters, which would otherwise not be present in the smoothed mortality curves obtained from the statistical model. Vital registration systems, survey programs and censuses might also miss some crisis-related deaths, due to disruptions in the notification of deaths, interruptions in data collection or because some households have been dissolved as a result of mass displacement.

WHO has been estimating conflict deaths for their Global Health Estimates (GHE) and has relied primarily on databases maintained by the Uppsala Conflict Data Program (UCDP) together with information from a number of country-specific data sources [35, 36]. Since 2014, the Armed Conflict Location and Event Data Project (ACLED) has been collecting and making available detailed disaggregated information on conflicts and political violence in over 100 countries in real-time [37, 38]. As of early 2020, ACLED included conflict deaths for 92 countries. ACLED includes all African countries from 1997 onwards. However, ACLED data has only become reasonably complete for several other regions from 2016 onwards and does not yet include countries in the Americas.

For updated estimates of conflict deaths for the period 1990-2019, ACLED data for conflict deaths to the end of 2019 were assessed for consistency with conflict deaths estimated using previous GHE methods. The latter were updated using the latest available data from UDCP for Battle-Related Deaths (version 19.1-1989-2018), Non-State Conflict Dataset (version 19.1-1989-2018), and One-sided Violence Dataset (version 19.1-1989-2018) [35]. Estimates for countries using other specific data sources [36] were also updated.

ACLED-based estimates are generally very consistent with the updated GHE estimates and were used to update years beyond 2006 for most regions. Additionally, for some high-conflict countries such as Afghanistan, Syria, Iraq and Yemen where GHE estimates have in the past been based on country-specific data sources with somewhat ad-hoc methods, ACLED offers a single reasonably consistent alternative and was used to revise and update the conflict death time series.

Age-sex distributions for conflict deaths were applied to estimates of total conflict deaths by country-year based on available distributions of conflict deaths by age and sex for specific conflicts [36].

Estimated deaths for major natural disasters up to and including 2019 were obtained from the EM-DAT/CRED International Disaster Database [39]. GHE age-sex distributions were used. These are based on a number of studies of earthquake deaths and tsunami deaths [36].

Direct and indirect deaths caused by the Ebola epidemic in 2014-2016 in Guinea, Liberia and Sierra Leone were added based on WHO Global Health Estimates.

For inclusion of crisis deaths, the splines regression model is fitted to "extreme event-free" observations (obtained as the observed values of the probability ${}_{10}q_{15}$ minus the ex-

treme events). The estimated additional mortality for the crisis event is then added. Uncertainty intervals for ${}_{10}q_{15}$ for the crises-years are based on the uncertainty in crisis-free ${}_{10}q_{15}$. The following criteria were used to identify crises:

1. The crisis was isolated to a few years,
2. Crisis deaths among adolescents and young adults aged 15-24 were >10 % of non-crisis deaths in this age group,
3. The crisis ${}_{10}q_{15}$ was greater than 0.2 per 1,000,
4. The number of crisis deaths among youth was greater than 10.

In total, adjustments for crises were made in 61 countries, listed below.

Country	Year(s) with adjustment
Albania	1997
Algeria	1994 1995 1997 1998 2003
Angola	1990 1993 1994
Armenia	1992 - 1994
Azerbaijan	1992 - 1994
Bahrain	2011
Bangladesh	1991
Bhutan	2000
Bosnia & Herzegovina	1992 - 1995
Burundi	1993 - 1998 2000 2002
Central African Republic	2013 2014 2017 2018
Chad	1990 2006
China	2008
Comoros	1997
Congo	1997 - 1999
Congo DR	1996 1997
Croatia	1991 - 1995
Djibouti	1992
El Salvador	1990
Ethiopia	1990 1999 2000
Federated States of Micronesia	2002
Georgia	1991 - 1993 2008
Guinea-Bissau	1998 1999
Haiti	2004 2010
Honduras	1998
Indonesia	2004
Iran	1990 2003
Iraq	1991 2003 - 2019

Country	Year(s) with adjustment
Israel	2002
Japan	1995 2011
Jordan	2005
Kuwait	1990 1991
Lebanon	1990 1996 2006 2008 2011 - 2017
Liberia	1990 1993 - 1996 2001 2003 2014
Libya	2011 2012 2014 - 2019
Macedonia	2001
Maldives	2004
Moldova	1992
Myanmar	2008
Nepal	2002 2004 2015
Nicaragua	1998
Papua New Guinea	1998
Peru	1990 1991
Philippines	1991
Rwanda	1993 - 1995 1997 2001 2009
Samoa	2009
Saudi Arabia	2017 - 2019
Serbia	1991 - 1993 1995
Sierra Leone	1995 1998 1999 2014
Somalia	1990 - 1992
South Sudan	2012 - 2017
Sri Lanka	1990 - 1992 2004 2008 2009
State of Palestine	2001 - 2004 2006 2009 2014
Sudan	1990 - 1993 1997 1998 2000 2001 2003 2004
Syria	2011 - 2020
Tajikistan	1992 1993 1996 1998
Turkey	1994 1997 1999 2016
Ukraine	2014 2015
Vanuatu	1997
Venezuela	1999
Yemen	1991 1992 1994 1995 1998 1999 2012 2014 - 2020

When applied to under-five mortality, the B3 model also includes adjustments for biases introduced by HIV/AIDS, because survey data come from mothers. Among these mothers, some are HIV-positive and without treatment, they face excess mortality and risk of transmitting the virus to their children. Birth histories, therefore, underestimate under-five mortality in settings where treatment is not widely available, or for earlier periods before treatment became widespread. No adjustment is made here for mortality between 15 and 25 years of age, as the data are derived from information provided on sibling survival by adult respondents.

2.7 Model validation

To assess the performance of the B3 model for ${}_{10}q_{15}$, we used out-of-sample validation. A training set was constructed by removing all data collected in or after 2013 (i.e. about 20% of observations were left out). All retrospective observations from a survey carried out in or after 2013 were left out of the training set, even if they referred to periods before 2013. We present below two sets of validation results: based on left-out observations (Tables S3 and S4), and based on the comparison between estimates obtained from the training and full data set (Table S5). Results are presented for estimates without crises in high mortality countries, defined as countries with a probability ${}_{10}q_{15}$ above 12 per 1000 youths aged 15 in 1990. We compare validation measures for ${}_{10}q_{15}$ with those obtained by Alkema and New (2014) for U5MR[15] and those obtained when applying B3 to mortality in children aged 5-14[16]. Results are presented for two values of the pooling weight κ; 0.5 to compare across studies and 0.8 as it is the pooling weight retained for ${}_{10}q_{15}$.

Table S3 presents the percentage of observations falling below and above the 90% uncertainty intervals based on the training set[17]. If the model is well-calibrated, around 5% of observations should fall below and above the 90% uncertainty intervals based on the training set. This is what we observed for the most recent period. There seems to be some asymmetry in coverage in the period before 2013, but this is based only on 26 high-mortality countries only. 11% of observations fall below the lower bound obtained from the training set, corresponding to about 3 countries out of 26. If the model is well calibrated, the chance of having at least 3 countries outside the interval is 13.8% (if we take a random sample of size 26 with a probability of 0.05 of being outside). Hence this percentage should not be interpreted as a sign of poor performance.

Results reported in Table S4 refer to the mean and median relative error and absolute relative error. Errors are defined as $\epsilon_i = u_i - \tilde{u}_i$, where $\tilde{u}_i$ is the posterior median of the predictive distribution for a left-out observation u_i based on the training set. The median or mean relative error (MRE) and the median or mean absolute relative error (MARE) are larger than those of under-five mortality for the period before 2012, but relatively similar for the most recent period.

Table S5 presents the results based on a comparison between estimates based on the training and full data set. The error in the estimate based on the training sample is defined as $\epsilon_{c,t} = \widehat{U}_c(t) - \widetilde{U}_c(t)$, where $\widetilde{U}_c(t)$ refers to the posterior median estimate based on the training sample, and $\widehat{U}_c(t)$ refers to the ${}_{10}q_{15}$ estimate obtained from the full data set for country c in year t. Relative error is defined as $\epsilon_{c,t}/\widehat{U}_c(t) \cdot 100$. Again, the validation measures are the mean and median relative error and absolute relative error, and the

[15]Their results refer to high mortality countries with U5MR above 40 per thousand in 1990, without HIV adjustments.

[16]These results refer to high mortality countries with ${}_{10}q_5$ above 8 per thousand in 1990.

[17]These percentages were calculated for 100 sets of left-out observations, where each set consisted of a random sample of one left-out observation per country. The results presented in Table S3 are the median and standard deviation of these percentages based on the outcomes in the 100 sets.

Table S3 – *Validation results based on left-out observations: Median and SD of percentage of observations below and above 90% predictions intervals based on the training set, from 100 sets of left-out observations, in countries with data in both the training and test set and left-out observations in the period of interest.*

$_{10}q_{15}$ (this study)

	% Below	% Above	Nb countries
$\kappa = 0.5$			
Year $\leq$ 2012	11.5 (4.3)	3.8 (2.6)	26
Year $>$ 2012	5.0 (2.1)	5.0 (1.4)	60
$\kappa = 0.8$			
Year $\leq$ 2012	15.4 (4.2)	3.8 (2.6)	26
Year $>$ 2012	5.0 (1.9)	5.0 (1.8)	60

$_{5}q_{0}$ (Sc: Alkema & New 2014 - $\kappa = 0.5$)

	% Below	% Above	Nb countries
Year $\leq$ 2005	7.0 (2.4)	7.0 (1.8)	71
Year $>$ 2005	6.2 (1.5)	1.5 (1.0)	65

$_{10}q_{5}$ (Sc: Masquelier et al. 2018 - $\kappa = 0.5$)

	% Below	% Above	Nb countries
Year $\leq$ 2007	2 (2.1)	2 (1.3)	49
Year $>$ 2007	5.8 (1.2)	0 (0.7)	52

Table S4 – *Validation results based on left-out observations: median or mean error (ME), median or mean relative error (MRE), median or mean absolute relative error (MARE) based on the training set.*

$_{10}q_{15}$ (this study)

**Median**	ME	MAE	MRE	MARE
$\kappa = 0.5$				
Year $\leq$ 2012	-1.2 (1.1)	5.1 (1.2)	-5.5 (5.7)	22.2 (4.1)
Year $>$ 2012	-0.6 (0.2)	2.5 (0.2)	-5.5 (2.1)	16.2 (1.8)
$\kappa = 0.8$				
Year $\leq$ 2012	-1.2 (1.2)	4.8 (1.2)	-5.2 (6.3)	20.9 (4.7)
Year $>$ 2012	-0.6 (0.2)	2.2 (0.2)	-4.6 (2.2)	15.3 (1.7)
**Mean**	ME	MAE	MRE	MARE
$\kappa = 0.5$				
Year $\leq$ 2012	-2.6 (1.1)	6.8 (0.8)	-20.0 (5.1)	33.3 (4.4)
Year $>$ 2012	-1.7 (0.4)	4.8 (0.3)	-14.4 (2.4)	30.1 (1.9)
$\kappa = 0.8$				
Year $\leq$ 2012	-2.7 (1.3)	6.9 (0.9)	-20.2 (5.4)	34.4 (4.3)
Year $>$ 2012	-1.6 (0.3)	4.4 (0.4)	-12.7 (2.1)	26.6 (1.7)

$_5q_0$ (Sc: Alkema & New 2014) - $\kappa = 0.5$

**Median**	ME	MAE	MRE	MARE
Year $\leq$ 2005	-1.7 (1.3)	10.7 (1.3)	-1.9 (1.3)	12.9 (1.4)
Year $>$ 2005	-3.7 (0.1)	7.6 (1.2)	-10.7 (1.1)	17.5 (1.5)
**Mean**	ME	MAE	MRE	MARE
Year$\leq$ 2005	-1.9 (1.4)	15.8 (1.1)	-3.8 (1.6)	17.6 (1.3)
Year $>$ 2005	-6.0 (0.5)	14.2 (0.5)	-14.8 (1.2)	25.8 (1.1)

$_{10}q_5$ (Sc: Masquelier et al., 2018) - $\kappa = 0.5$

**Median**	ME	MAE	MRE	MARE
Year $\leq$ 2007	-0.4 (0.5)	4.4 (0.5)	-5.0 (3.6)	33.3 (3.9)
Year $>$ 2007	0 (0.4)	3.5 (0.6)	0.9 (3.2)	31.4 (2.9)
**Mean**	ME	MAE	MRE	MARE
Year $\leq$ 2007	1 (0.5)	5.6 (0.5)	-23.2 (6.6)	47.8 (6)
Year $>$ 2007	-0.4 (0.1)	2.4 (0.1)	-32.1 (1.5)	50.1 (1.5)

percentage of estimates obtained from the full dataset that are falling below or above the 90% uncertainty intervals obtained from the training set only. Overall, these results indicate that the model performance is not substantially different for ${}_{10}q_{15}$ than when applied to the other age groups.

Table S5 – *Validation measures based on the comparison between estimates based on the training and full data set.*

${}_{10}q_{15}$ (this study)

	Mean			Median				
	MRE	MARE	S	MRE	MARE	S	% below	% above
$\kappa = 0.5$								
2007	-3.4	6.4	0.4	-0.3	1.6	0.4	3.3	0.0
2012	-6.8	14.9	0.9	-0.8	8.1	0.8	4.9	4.9
$\kappa = 0.8$								
2007	-3.4	6.3	0.4	-0.3	1.6	0.4	3.3	0.0
2012	-5.9	13.7	0.8	-0.8	7.9	0.8	4.9	4.9

${}_{5}q_{0}$ (Sc: Alkema and New 2014) - $\kappa = 0.5$

	Mean			Median				
	MRE	MARE	S	MRE	MARE	S	% below	% above
2000	-2.4	4.4	1.2	-4.1	9	1.6	3.8	5.1
2005	-6.1	8.1	1.2	-8.9	14.7	1.6	7.7	1.3

${}_{10}q_{5}$ (Sc: Masquelier et al., 2018) - $\kappa = 0.5$

	Mean			Median				
	MRE	MARE	S	MRE	MARE	S	% below	% above
2000	-4.1	11.5	0.6	-1.1	6.0	0.4	1.9	5.6
2007	-14.7	23.0	1.0	-6.6	14.4	0.9	5.6	3.7

2.8 Deriving estimates for countries with insufficient data

In 37 countries with insufficient data sources, we estimated youth mortality based on the relationship observed between U5MR and the probability ${}_{10}q_{15}$ in countries for which the B3 model was used. Our approach is similar to that used to exclude certain surveys on the basis of the plausibility of mortality age patterns (section 1.2.2), except that we can use the results of the B3 model rather than the standard constructed from vital registration and HDSS data. The B3 estimates offer more insights into the diversity of regional patterns than the standard mortality age pattern described above. We removed the quadratic term and fit a varying-slope and varying-intercept model to predict $\log({}_{10}q_{15})$ from $\log({}_{5}q_{0})$, allowing the slope of the $\log({}_{5}q_{0})$ variable to vary by region [40]:

$$log({}_{10}q_{15})_i = N(\alpha_{j[i]} + \beta_{j[i]} log({}_{5}q_{0})_i, \sigma_y^2) \text{ for } i = 1, \ldots, n \text{ country-year estimates.} \tag{13}$$

$$\begin{pmatrix} \alpha_j \\ \beta_j \end{pmatrix} = N\left(\begin{pmatrix} \mu_\alpha \\ \mu_\beta \end{pmatrix}, \begin{pmatrix} \sigma_\alpha^2 & \rho\sigma_\alpha\sigma_\beta \\ \rho\sigma_\alpha\sigma_\beta & \sigma_\beta^2 \end{pmatrix} \right) \text{ for } j = 1, \ldots, J \text{ UNICEF regions.}$$

The fixed effect parameter estimates are presented in Table S6 and Figure S4 displays the model predictions for different regions (using the UNICEF classification), for the range of U5MR estimates observed in each region in the period 1990-2019. We combined the median value of U5MR estimates from UN IGME for each country-year between 1990 and 2019 with the fixed and random effects of this model to obtain a point estimate $\log({}_{10}q_{15})$. We did not account for uncertainty quantified by σ_y^2 or the uncertainty around the U5MR estimates to make these predictions.

For a given level of child mortality, there are significant regional differences in the expected levels of the ${}_{10}q_{15}$ probability. For example, for a level of 50 per thousand, the predicted ${}_{10}q_{15}$ probability will be 11 per thousand in Eastern Europe and Central Asia, compared to 16 in East Asia and the Pacific, and up to 24 in Eastern and Southern Africa. The estimates for data-poor countries are to be considered with caution. They will be revised in future iterations as the database develops, by making greater use of non-standard surveys for these countries (upon data availability). This modelling covers a very small share of the world population aged 15-24 since these 37 countries represent only 4.9% of the population in this age group[18].

[18] In Eswatini, we excluded all data series and derived ${}_{10}q_{15}$ from ${}_{5}q_{0}$ as our database did not include any data point after 2007 and the period after the scale-up of ART treatment was not well represented. Estimates from the 2012 Intercensal survey and the 2017 census will be used when they are publicly available.

Table S6 – *Fixed effect parameter estimates from the varying slope and varying intercept model*

	log10q15
logU5MR	0.473***
	(0.030)
Constant	−2.774***
	(0.189)
N	4,770
Log Likelihood	−1,576.103
AIC	3,164.206
BIC	3,203.027

*p < .05; **p < .01; ***p < .001

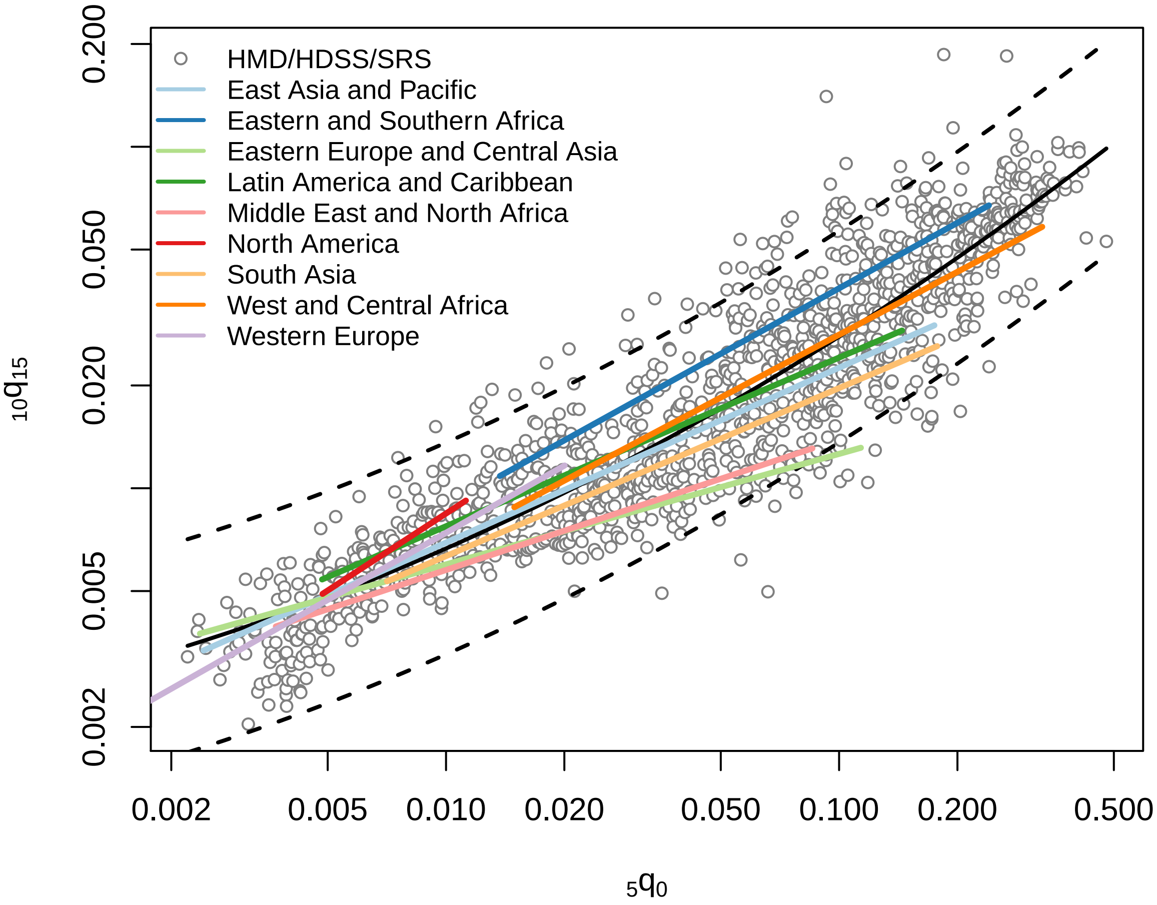

Figure S4 – *Relationship between* $_5q_0$ *and* $_{10}q_{15}$ *in the life tables from HMD, HDSS, SRS in India and VR in Mauritius, and predictions from the varying slope and varying intercept model.*

2.9 Calculation of the annual rate of reduction

The annual rate of reduction (in %) between two years, y_1 and y_2 ($y_1 < y_2$), is computed as:

$$\texttt{ARR} = 100 * \frac{log\left(\frac{{}_{10}q_{15}(y_2)}{{}_{10}q_{15}(y_1)}\right)}{y_2 - y_1}.$$

3 List of data series

Table S7 lists all countries included in the analysis, their region, the different data series included in the mortality database and indicates the type of data collection. Some data series were included in the database but excluded from the statistical model. The 'Inclusion' column identifies these series with a 0. Excluded series refer to country-years with vital registration data until 1989 (as these vital statistics were not used for modelling trends from 1990), incomplete vital registration data or surveys and censuses with implausible age patterns. This column is also set to '0' for countries for which trends in ${}_{10}q_{15}$ were modelled based on trends in U5MR (because of insufficient data series).

Country	Region	Series	Years	Inclusion	Type
Afghanistan	South Asia	Census	1979	1	Household Deaths
		Afghanistan Mortality Survey (excluding South Zone)	2010	1	Household Deaths
		Afghanistan Mortality Survey (excluding South Zone)	2010	1	Sibling survival histories
		Demographic and Health Survey	2015	1	Sibling survival histories
Albania	Eastern Europe and Central Asia	Census	2011	1	Household Deaths
		WHO VR (recalculated - unadjusted)	1980-2012	0	Vital Registration
		WHO VR (recalculated - unadjusted)	1989-2020	1	Vital Registration
Algeria	Middle East and North Africa	Demographic Survey	1970	1	Household Deaths
		WHO VR (recalculated - unadjusted)	1980-1986	0	Vital Registration
		WHO VR (recalculated - unadjusted)	1986-2020	1	Vital Registration
Andorra	Western Europe	WHO VR (recalculated - unadjusted)	1994-2019	0	Vital Registration
Angola	Eastern and Southern Africa	Census	2014	0	Household Deaths
		Inquerito de Indicadores Multiplos e de Saude	2016	0	Sibling survival histories
Antigua and Barbuda	Latin America and Caribbean	WHO VR (recalculated - unadjusted)	1961-1996	0	Vital Registration
		WHO VR (recalculated - unadjusted)	1996-2018	1	Vital Registration
Argentina	Latin America and Caribbean	WHO VR (recalculated - unadjusted)	1966-1989	0	Vital Registration
		WHO VR (recalculated - unadjusted)	1989-2019	1	Vital Registration
Armenia	Eastern Europe and Central Asia	WHO VR (recalculated - unadjusted)	1981-1989	0	Vital Registration
		WHO VR (recalculated - unadjusted)	1989-2019	1	Vital Registration
Australia	East Asia and Pacific	WHO VR (recalculated - unadjusted)	1950-1989	0	Vital Registration
		WHO VR (recalculated - unadjusted)	1989-2019	1	Vital Registration
Austria	Western Europe	WHO VR (recalculated - unadjusted)	1950-1989	0	Vital Registration
		WHO VR (recalculated - unadjusted)	1989-2018	1	Vital Registration
Azerbaijan	Eastern Europe and Central Asia	WHO VR (recalculated - unadjusted)	1981-2018	0	Vital Registration
		WHO VR (recalculated - adjusted for incompleteness)	1989-2018	1	Vital Registration
Bahamas	Latin America and Caribbean	WHO VR (recalculated - unadjusted)	1969-2016	0	Vital Registration
		WHO VR (recalculated - adjusted for incompleteness)	1990-2016	1	Vital Registration
Bahrain	Middle East and North Africa	WHO VR (recalculated - unadjusted)	1980-1989	0	Vital Registration
		WHO VR (recalculated - unadjusted)	1989-2015	1	Vital Registration
Bangladesh	South Asia	Maternal Health Services and Maternal Mortality Survey	2001	0	Sibling survival histories
		World Health Survey	2003	1	Sibling survival histories
		Census	2011	1	Household Deaths
		SVR (from published reports)	1981-2016	1	Sample Vital Registration
Barbados	Latin America and Caribbean	WHO VR (recalculated - unadjusted)	1955-1989	0	Vital Registration
		WHO VR (recalculated - unadjusted)	1989-2014	1	Vital Registration
Belarus	Eastern Europe and Central Asia	WHO VR (recalculated - unadjusted)	1959-1989	0	Vital Registration
		WHO VR (recalculated - unadjusted)	1989-2019	1	Vital Registration
Belgium	Western Europe	WHO VR (recalculated - unadjusted)	1950-1989	0	Vital Registration
		WHO VR (recalculated - unadjusted)	1989-2019	1	Vital Registration
Belize	Latin America and Caribbean	WHO VR (recalculated - unadjusted)	1964-2000	0	Vital Registration
		WHO VR (recalculated - unadjusted)	2000-2019	1	Vital Registration
		WHO VR (recalculated - adjusted for incompleteness)	1991-2000	1	Vital Registration
		WHO VR (recalculated - adjusted for incompleteness)	2000-2019	0	Vital Registration
Benin	West and Central Africa	Demographic and Health Survey	1996	1	Sibling survival histories
		Demographic and Health Survey	2006	1	Sibling survival histories
		Multiple Indicator Cluster Survey	2014	1	Sibling survival histories

Country	Region	Series	Years	Inclusion	Type
		Demographic and Health Survey	2018	1	Sibling survival histories
Bhutan	South Asia	National Health Survey	1994	1	Household Deaths
		National Health Survey	2000	1	Household Deaths
		Census	2005	1	Household Deaths
		Multiple Indicator Cluster Survey	2010	0	Sibling survival histories
		National Health Survey	2012	1	Household Deaths
Bolivia (Plurinational State of)	Latin America and Caribbean	National Demographic Survey	1980	1	Household Deaths
		Census	1992	1	Household Deaths
		Demographic and Health Survey	1993-1994	1	Sibling survival histories
		Census	2001	1	Household Deaths
		Demographic and Health Survey	2003	1	Sibling survival histories
		Demographic and Health Survey	2008	1	Sibling survival histories
		Census	2012	0	Household Deaths
Bosnia and Herzegovina	Eastern Europe and Central Asia	World Health Survey	2003	0	Sibling survival histories
		WHO VR (recalculated - unadjusted)	1985-1989	0	Vital Registration
		WHO VR (recalculated - unadjusted)	1989-2017	1	Vital Registration
Botswana	Eastern and Southern Africa	Census	1981	1	Household Deaths
		Census	1991	1	Household Deaths
		Demographic Survey	1998	1	Household Deaths
		Census	2001	1	Household Deaths
		Demographic Survey	2006	1	Household Deaths
		Census	2011	1	Household Deaths
Brazil	Latin America and Caribbean	Demographic and Health Survey	1996	1	Sibling survival histories
		Census	2010	1	Household Deaths
		WHO VR (recalculated - unadjusted)	1979-1989	0	Vital Registration
		WHO VR (recalculated - unadjusted)	1989-2018	1	Vital Registration
Brunei Darussalam	East Asia and Pacific	WHO VR (recalculated - unadjusted)	1982-2020	0	Vital Registration
		WHO VR (recalculated - adjusted for incompleteness)	1991-2020	1	Vital Registration
Bulgaria	Eastern Europe and Central Asia	WHO VR (recalculated - unadjusted)	1950-1989	0	Vital Registration
		WHO VR (recalculated - unadjusted)	1989-2020	1	Vital Registration
Burkina Faso	West and Central Africa	Census	1985	1	Household Deaths
		Census	1996	1	Household Deaths
		Demographic and Health Survey	1998-1999	1	Sibling survival histories
		Demographic and Health Survey	2003	1	Sibling survival histories
		World Health Survey	2003	1	Sibling survival histories
		Census	2006	1	Household Deaths
		Demographic and Health Survey	2010	1	Sibling survival histories
Burundi	Eastern and Southern Africa	Population Survey	1965	1	Household Deaths
		Demographic Survey	1970	1	Household Deaths
		Demographic Survey	2002	1	Sibling survival histories
		Census	2008	1	Household Deaths
		Demographic and Health Survey	2010	1	Sibling survival histories
		Demographic and Health Survey	2017	1	Sibling survival histories
Cabo Verde	West and Central Africa	Census	2000	1	Household Deaths
		Census	2010	1	Household Deaths
		WHO VR (recalculated - unadjusted)	1980-1986	0	Vital Registration

Country	Region	Series	Years	Inclusion	Type
		WHO VR (recalculated - unadjusted)	1990-2014	1	Vital Registration
Cambodia	East Asia and Pacific	Demographic and Health Survey	2000	1	Sibling survival histories
		Demographic and Health Survey	2005	1	Sibling survival histories
		Demographic and Health Survey	2010	1	Sibling survival histories
		Demographic and Health Survey	2014	1	Sibling survival histories
Cameroon	West and Central Africa	Census	1976	1	Household Deaths
		Census	1987	1	Household Deaths
		Demographic and Health Survey	1998	1	Sibling survival histories
		Demographic and Health Survey	2004	1	Sibling survival histories
		Census	2005	1	Household Deaths
		Demographic and Health Survey	2011	1	Sibling survival histories
		Demographic and Health Survey	2018	1	Sibling survival histories
Canada	North America	WHO VR (recalculated - unadjusted)	1950-1989	0	Vital Registration
		WHO VR (recalculated - unadjusted)	1989-2019	1	Vital Registration
Central African Republic	West and Central Africa	Survey	1959-1960	1	Household Deaths
		Census	1988	1	Household Deaths
		Demographic and Health Survey	1994-1995	1	Sibling survival histories
		Census	2003	1	Household Deaths
Chad	West and Central Africa	Demographic and Health Survey	1996-1997	1	Sibling survival histories
		World Health Survey	2003	0	Sibling survival histories
		Demographic and Health Survey	2004	1	Sibling survival histories
		Census	2009	1	Household Deaths
		Demographic and Health Survey	2014-2015	1	Sibling survival histories
Chile	Latin America and Caribbean	WHO VR (recalculated - unadjusted)	1955-1989	0	Vital Registration
		WHO VR (recalculated - unadjusted)	1989-2016	1	Vital Registration
China	East Asia and Pacific	Adjusted Census Deaths	(1964-1982)	1	Household Deaths
		Adjusted Census Deaths	(1982-1990)	1	Household Deaths
		Adjusted Census Deaths	(1990-2000)	1	Household Deaths
		Adjusted Census Deaths	(1999-2000)	1	Household Deaths
		Adjusted Census Deaths	(2000-2010)	1	Household Deaths
		Population 1% Sample Survey	2005	1	Household Deaths
		China 1987 One-per-Hundred National Population Survey	1987	1	Household Deaths
		China 1995 One-percent Sample Survey	1995	1	Household Deaths
		World Health Survey	2003	0	Sibling survival histories
		China 2007 Annual Population Change Survey	2007	1	Household Deaths
		China 2008 Annual Population Change Survey	2008	1	Household Deaths
		China 2009 Annual Population Change Survey	2009	0	Household Deaths
		China CDC Surveillance surveys	2013	0	Sample Vital Registration
		China CDC Surveillance surveys	2013	1	Sample Vital Registration
		Population Sample Survey	2015	0	Household Deaths
Colombia	Latin America and Caribbean	Census	2005	0	Household Deaths
		Demographic and Health Survey	2015	1	Sibling survival histories
		WHO VR (recalculated - unadjusted)	1955-1993	0	Vital Registration
		WHO VR (recalculated - unadjusted)	1993-2020	1	Vital Registration
Comoros	Eastern and Southern Africa	Census	1958	1	Household Deaths
		Census	1980	1	Household Deaths
		Census	2003	1	Household Deaths

Country	Region	Series	Years	Inclusion	Type
		World Health Survey	2003	0	Sibling survival histories
		Demographic and Health Survey	2012	1	Sibling survival histories
Congo	West and Central Africa	Census	1984	0	Household Deaths
		World Health Survey	2003	0	Sibling survival histories
		Demographic and Health Survey	2005	1	Sibling survival histories
		Demographic and Health Survey	2011-2012	1	Sibling survival histories
		Multiple Indicator Cluster Survey	2015	1	Sibling survival histories
Cook Islands	East Asia and Pacific	WHO VR (recalculated - unadjusted)	1980-1987	0	Vital Registration
		WHO VR (recalculated - unadjusted)	1987-2010	1	Vital Registration
Costa Rica	Latin America and Caribbean	WHO VR (recalculated - unadjusted)	1956-1989	0	Vital Registration
		WHO VR (recalculated - unadjusted)	1989-2020	1	Vital Registration
Côte d'Ivoire	West and Central Africa	Demographic and Health Survey	1994	1	Sibling survival histories
		World Health Survey	2003	1	Sibling survival histories
		AIDS Indicator Survey	2005	1	Sibling survival histories
		Demographic and Health Survey	2011-2012	1	Sibling survival histories
Croatia	Eastern Europe and Central Asia	Census	2011	1	Household Deaths
		WHO VR (recalculated - unadjusted)	1982-1989	0	Vital Registration
		WHO VR (recalculated - unadjusted)	1989-2019	1	Vital Registration
Cuba	Latin America and Caribbean	WHO VR (recalculated - unadjusted)	1964-1989	0	Vital Registration
		WHO VR (recalculated - unadjusted)	1989-2020	1	Vital Registration
Cyprus	Western Europe	WHO VR (recalculated - unadjusted)	1980-2018	0	Vital Registration
		WHO VR (recalculated - unadjusted)	1989-2001	1	Vital Registration
		WHO VR (recalculated - adjusted for incompleteness)	2001-2018	1	Vital Registration
Czechia	Western Europe	WHO VR (recalculated - unadjusted)	1950-1989	0	Vital Registration
		WHO VR (recalculated - unadjusted)	1989-2020	1	Vital Registration
Democratic People's Republic of Korea	East Asia and Pacific	Census	1993	0	Household Deaths
		Census	2008	0	Household Deaths
Democratic Republic of the Congo	West and Central Africa	SPS	1955-1957	1	Household Deaths
		Demographic and Health Survey	2007	1	Sibling survival histories
		Demographic and Health Survey	2013-2014	1	Sibling survival histories
Denmark	Western Europe	WHO VR (recalculated - unadjusted)	1951-1989	0	Vital Registration
		WHO VR (recalculated - unadjusted)	1989-2020	1	Vital Registration
Dominica	Latin America and Caribbean	WHO VR (recalculated - unadjusted)	1961-1985	0	Vital Registration
		WHO VR (recalculated - unadjusted)	1985-2016	1	Vital Registration
Dominican Republic	Latin America and Caribbean	Demographic and Health Survey	2002	1	Sibling survival histories
		World Health Survey	2003	1	Sibling survival histories
		Demographic and Health Survey	2007	1	Sibling survival histories
		Census	2010	1	Household Deaths
		Demographic and Health Survey	2013	1	Household Deaths
		WHO VR (recalculated - unadjusted)	1958-2020	0	Vital Registration
Ecuador	Latin America and Caribbean	World Health Survey	2003	1	Sibling survival histories
		National Health and Nutrition Survey	2012	1	Household Deaths
		WHO VR (recalculated - unadjusted)	1968-2018	0	Vital Registration
		WHO VR (recalculated - unadjusted)	1990-2002	1	Vital Registration
Egypt	Middle East and North Africa	WHO VR (recalculated - unadjusted)	1955-1989	0	Vital Registration
		WHO VR (recalculated - unadjusted)	1989-2020	1	Vital Registration
El Salvador	Latin America and Caribbean	Census	1992	1	Household Deaths

Country	Region	Series	Years	Inclusion	Type
		Census	2007	0	Household Deaths
		WHO VR (recalculated - unadjusted)	1958-1989	0	Vital Registration
		WHO VR (recalculated - unadjusted)	1989-2017	1	Vital Registration
Equatorial Guinea	West and Central Africa	Census	1994	0	Household Deaths
Estonia	Western Europe	Census	2011-2012	1	Household Deaths
		WHO VR (recalculated - unadjusted)	1959-1989	0	Vital Registration
		WHO VR (recalculated - unadjusted)	1989-2020	1	Vital Registration
Eswatini	Eastern and Southern Africa	Census	1997	0	Household Deaths
		World Health Survey	2003	0	Sibling survival histories
		Demographic and Health Survey	2006-2007	0	Sibling survival histories
		Census	2007	0	Household Deaths
Ethiopia	Eastern and Southern Africa	Demographic and Health Survey	2000	1	Sibling survival histories
		World Health Survey	2003	1	Sibling survival histories
		Demographic and Health Survey	2005	1	Sibling survival histories
		Census	2007	1	Household Deaths
		Demographic and Health Survey	2011	1	Sibling survival histories
		Demographic and Health Survey	2016	1	Sibling survival histories
Fiji	East Asia and Pacific	WHO VR (recalculated - unadjusted)	1978-2000	0	Vital Registration
		WHO VR (recalculated - unadjusted)	2000-2018	1	Vital Registration
Finland	Western Europe	WHO VR (recalculated - unadjusted)	1950-1989	0	Vital Registration
		WHO VR (recalculated - unadjusted)	1989-2020	1	Vital Registration
France	Western Europe	WHO VR (recalculated - unadjusted)	1950-1989	0	Vital Registration
		WHO VR (recalculated - unadjusted)	1989-2019	1	Vital Registration
Gabon	West and Central Africa	Demographic and Health Survey	2000	1	Sibling survival histories
		Demographic and Health Survey	2012	1	Sibling survival histories
Gambia	West and Central Africa	Census	2013	0	Household Deaths
		Demographic and Health Survey	2013	0	Sibling survival histories
Georgia	Eastern Europe and Central Asia	World Health Survey	2003	0	Sibling survival histories
		WHO VR (recalculated - unadjusted)	1981-2002	0	Vital Registration
		WHO VR (recalculated - unadjusted)	2002-2019	1	Vital Registration
		WHO VR (recalculated - adjusted for incompleteness)	1989-2002	1	Vital Registration
		WHO VR (recalculated - adjusted for incompleteness)	2002-2019	0	Vital Registration
Germany	Western Europe	WHO VR (recalculated - unadjusted)	1969-1989	0	Vital Registration
		WHO VR (recalculated - unadjusted)	1989-2019	1	Vital Registration
Ghana	West and Central Africa	World Health Survey	2003	1	Sibling survival histories
		Maternal Health Survey	2007	1	Sibling survival histories
		Census	2010	1	Household Deaths
		Demographic and Health Survey	2017	1	Household deaths
Greece	Western Europe	WHO VR (recalculated - unadjusted)	1956-1989	0	Vital Registration
		WHO VR (recalculated - unadjusted)	1989-2019	1	Vital Registration
Grenada	Latin America and Caribbean	WHO VR (recalculated - unadjusted)	1985-2001	0	Vital Registration
		WHO VR (recalculated - unadjusted)	2001-2019	1	Vital Registration
		WHO VR (recalculated - adjusted for incompleteness)	1989-2001	1	Vital Registration
		WHO VR (recalculated - adjusted for incompleteness)	2001-2019	0	Vital Registration
Guatemala	Latin America and Caribbean	Demographic and Health Survey	1995	1	Sibling survival histories
		WHO VR (recalculated - unadjusted)	1958-1989	0	Vital Registration

Country	Region	Series	Years	Inclusion	Type
		WHO VR (recalculated - unadjusted)	1989-2018	1	Vital Registration
Guinea	West and Central Africa	Census	1983	0	Household Deaths
		Census	1996	1	Household Deaths
		Demographic and Health Survey	1999	1	Sibling survival histories
		Demographic and Health Survey	2005	1	Sibling survival histories
		Demographic and Health Survey	2012	1	Sibling survival histories
		Census	2014	1	Household Deaths
Guinea-Bissau	West and Central Africa	Census	2009	0	Household Deaths
		Multiple Indicator Cluster Survey	2014	0	Sibling survival histories
Guyana	Latin America and Caribbean	Census	2012	0	Household Deaths
		WHO VR (recalculated - unadjusted)	1977-1989	0	Vital Registration
		WHO VR (recalculated - unadjusted)	1989-2015	1	Vital Registration
Haiti	Latin America and Caribbean	Census	1971	0	Household Deaths
		Demographic and Health Survey	2000	1	Sibling survival histories
		Demographic and Health Survey	2005-2006	1	Sibling survival histories
		Demographic and Health Survey	2017	1	Sibling survival histories
		WHO VR (recalculated - unadjusted)	1980-2005	0	Vital Registration
Honduras	Latin America and Caribbean	National Demographic Survey	1983	0	Household Deaths
		Census	2001	0	Household Deaths
		Census	2013	0	Household Deaths
		WHO VR (recalculated - unadjusted)	1966-2014	0	Vital Registration
Hungary	Western Europe	WHO VR (recalculated - unadjusted)	1950-1989	0	Vital Registration
		WHO VR (recalculated - unadjusted)	1989-2019	1	Vital Registration
Iceland	Western Europe	WHO VR (recalculated - unadjusted)	1950-1989	0	Vital Registration
		WHO VR (recalculated - unadjusted)	1989-2020	1	Vital Registration
India	South Asia	World Health Survey	2003	1	Sibling survival histories
		Sample Registration System	2019	0	Sample Vital Registration
		Sample Registration System	1968-2016	1	Sample Vital Registration
Indonesia	East Asia and Pacific	National Socio-economic Survey	1964-1965	1	Household Deaths
		Demographic and Health Survey	1994	1	Sibling survival histories
		Demographic and Health Survey	1997	0	Sibling survival histories
		Demographic and Health Survey	2002-2003	1	Sibling survival histories
		Demographic and Health Survey	2007	1	Sibling survival histories
		Census	2010	1	Household Deaths
		Demographic and Health Survey	2012	1	Sibling survival histories
Iran (Islamic Republic of)	Middle East and North Africa	Population Growth Survey	1973-1976	1	Sibling survival histories
		Census	1976	1	Household Deaths
		Census	1986	1	Household Deaths
		Census	1991	1	Household Deaths
		Demographic and Health Survey	2000	1	Household Deaths
		Based on MOH data - adjusted for incompleteness	2017	1	Vital Registration
		Based on 2004 DH - adjusted for incompleteness	2017	1	Vital Registration
		WHO VR (recalculated - unadjusted)	1983-2018	0	Vital Registration
		WHO VR (recalculated - adjusted for incompleteness)	2006-2018	1	Vital Registration
Iraq	Middle East and North Africa	Demographic Sample Survey and Sample Registration System	1973	0	Sibling survival histories
		WHO VR (recalculated - unadjusted)	1987-2018	0	Vital Registration
Ireland	Western Europe	Census	2011	1	Household Deaths

Country	Region	Series	Years	Inclusion	Type
		WHO VR (recalculated - unadjusted)	1950-1989	0	Vital Registration
		WHO VR (recalculated - unadjusted)	1989-2018	1	Vital Registration
Israel	Middle East and North Africa	WHO VR (recalculated - unadjusted)	1975-1989	0	Vital Registration
		WHO VR (recalculated - unadjusted)	1989-2020	1	Vital Registration
Italy	Western Europe	WHO VR (recalculated - unadjusted)	1950-1989	0	Vital Registration
		WHO VR (recalculated - unadjusted)	1989-2018	1	Vital Registration
Jamaica	Latin America and Caribbean	WHO VR (recalculated - unadjusted)	1960-2019	0	Vital Registration
Japan	East Asia and Pacific	WHO VR (recalculated - unadjusted)	1950-1989	0	Vital Registration
		WHO VR (recalculated - unadjusted)	1989-2019	1	Vital Registration
Jordan	Middle East and North Africa	Population and Family Health Survey	1997	1	Sibling survival histories
		Census	2015	1	Household Deaths
		WHO VR (recalculated - unadjusted)	1965-2019	0	Vital Registration
		WHO VR (recalculated - adjusted for incompleteness)	2004-2019	1	Vital Registration
Kazakhstan	Eastern Europe and Central Asia	World Health Survey	2003	1	Sibling survival histories
		WHO VR (recalculated - unadjusted)	1981-1989	0	Vital Registration
		WHO VR (recalculated - unadjusted)	1989-2020	1	Vital Registration
Kenya	Eastern and Southern Africa	Demographic and Health Survey	1998	1	Sibling survival histories
		Demographic and Health Survey	2003	1	Sibling survival histories
		World Health Survey	2003	1	Sibling survival histories
		Demographic and Health Survey	2008-2009	1	Sibling survival histories
		Census	2009	1	Household Deaths
		Demographic and Health Survey	2014	1	Sibling survival histories
Kiribati	East Asia and Pacific	WHO VR (recalculated - unadjusted)	1991-2017	0	Vital Registration
Kuwait	Middle East and North Africa	WHO VR (recalculated - unadjusted)	1975-1989	0	Vital Registration
		WHO VR (recalculated - unadjusted)	1989-2018	1	Vital Registration
Kyrgyzstan	Eastern Europe and Central Asia	WHO VR (recalculated - unadjusted)	1981-1999	0	Vital Registration
		WHO VR (recalculated - unadjusted)	1999-2020	1	Vital Registration
		WHO VR (recalculated - adjusted for incompleteness)	1989-1999	1	Vital Registration
		WHO VR (recalculated - adjusted for incompleteness)	1999-2020	0	Vital Registration
Lao People's Democratic Republic	East Asia and Pacific	Census	1995	1	Household Deaths
		World Health Survey	2003	1	Sibling survival histories
		Census	2005	1	Household Deaths
		Multiple Indicator Cluster Survey	2012	0	Sibling survival histories
		Census	2015	1	Household Deaths
Latvia	Western Europe	WHO VR (recalculated - unadjusted)	1959-1989	0	Vital Registration
		WHO VR (recalculated - unadjusted)	1989-2019	1	Vital Registration
Lebanon	Middle East and North Africa	WHO VR (recalculated - unadjusted)	2017-2018	0	Vital Registration
Lesotho	Eastern and Southern Africa	Demographic Survey	1971-1973	0	Household Deaths
		World Fertility Survey	1977	0	Household Deaths
		Census	1986	0	Household Deaths
		Census	1996	1	Household Deaths
		Demographic and Health Survey	2004	1	Sibling survival histories
		Census	2006	0	Household Deaths
		Demographic and Health Survey	2009	1	Sibling survival histories
		Demographic and Health Survey	2014	1	Sibling survival histories
		Census	2016	1	Household Deaths
Liberia	West and Central Africa	Demographic and Health Survey	2006-2007	1	Sibling survival histories

Country	Region	Series	Years	Inclusion	Type
		Census	2008	0	Household Deaths
		Demographic and Health Survey	2013	1	Sibling survival histories
Libya	Middle East and North Africa	WHO VR (recalculated - unadjusted)	1981-2011	0	Vital Registration
Lithuania	Western Europe	Census	2011	1	Household Deaths
		Statistical Survey	2018	1	Household Deaths
		WHO VR (recalculated - unadjusted)	1959-1989	0	Vital Registration
		WHO VR (recalculated - unadjusted)	1989-2020	1	Vital Registration
Luxembourg	Western Europe	WHO VR (recalculated - unadjusted)	1960-1989	0	Vital Registration
		WHO VR (recalculated - unadjusted)	1989-2018	1	Vital Registration
Madagascar	Eastern and Southern Africa	Census	1993	0	Household Deaths
		Demographic and Health Survey	1997	1	Sibling survival histories
		Demographic and Health Survey	2003-2004	1	Sibling survival histories
		Demographic and Health Survey	2008-2009	1	Sibling survival histories
Malawi	Eastern and Southern Africa	Population Change Survey	1970-1972	1	Household Deaths
		Census	1977	0	Household Deaths
		Family Formation Survey	1984	0	Household Deaths
		Census	1987	0	Household Deaths
		Demographic and Health Survey	1992	1	Sibling survival histories
		Census	1998	1	Household Deaths
		Demographic and Health Survey	2000	1	Sibling survival histories
		World Health Survey	2003	1	Sibling survival histories
		Demographic and Health Survey	2004	1	Sibling survival histories
		Second Integrated Household Survey	2004-2005	1	Household Deaths
		Census	2008	1	Household Deaths
		Demographic and Health Survey	2010	0	Household Deaths
		Demographic and Health Survey	2010	1	Sibling survival histories
		Multiple Indicator Cluster Survey	2014	1	Sibling survival histories
		Demographic and Health Survey	2015-2016	1	Sibling survival histories
Malaysia	East Asia and Pacific	World Health Survey	2003	0	Sibling survival histories
		WHO VR (recalculated - unadjusted)	1990-2000	0	Vital Registration
		WHO VR (recalculated - unadjusted)	1991-2019	1	Vital Registration
Maldives	South Asia	WHO VR (recalculated - unadjusted)	1984-1989	0	Vital Registration
		WHO VR (recalculated - unadjusted)	1989-2020	1	Vital Registration
Mali	West and Central Africa	Census	1976	0	Household Deaths
		Census	1987	1	Household Deaths
		Demographic and Health Survey	1995-1996	0	Sibling survival histories
		Census	1998	0	Household Deaths
		Demographic and Health Survey	2001	1	Sibling survival histories
		World Health Survey	2003	1	Sibling survival histories
		Demographic and Health Survey	2006	1	Sibling survival histories
		Census	2009	0	Household Deaths
		Demographic and Health Survey	2012-2013	1	Sibling survival histories
		Demographic and Health Survey	2018	1	Sibling survival histories
Malta	Western Europe	WHO VR (recalculated - unadjusted)	1955-1989	0	Vital Registration
		WHO VR (recalculated - unadjusted)	1989-2018	1	Vital Registration
Marshall Islands	East Asia and Pacific	WHO VR (recalculated - unadjusted)	1986-2018	0	Vital Registration
Mauritania	West and Central Africa	Census	1976-1977	0	Household Deaths

Country	Region	Series	Years	Inclusion	Type
		Census	1988	0	Household Deaths
		World Health Survey	2003	0	Sibling survival histories
		Multiple Indicator Cluster Survey	2011	0	Sibling survival histories
		Census	2013	0	Household Deaths
Mauritius	Eastern and Southern Africa	WHO VR (recalculated - unadjusted)	1957-1989	0	Vital Registration
		WHO VR (recalculated - unadjusted)	1989-2020	1	Vital Registration
Mexico	Latin America and Caribbean	World Health Survey	2003	0	Sibling survival histories
		WHO VR (recalculated - unadjusted)	1955-1989	0	Vital Registration
		WHO VR (recalculated - unadjusted)	1989-2019	1	Vital Registration
Monaco	Western Europe	WHO VR (recalculated - unadjusted)	1981-1984	0	Vital Registration
Mongolia	East Asia and Pacific	WHO VR (recalculated - unadjusted)	1991-2020	0	Vital Registration
		WHO VR (recalculated - adjusted for incompleteness)	1991-2020	1	Vital Registration
Montenegro	Eastern Europe and Central Asia	WHO VR (recalculated - unadjusted)	1985-1989	0	Vital Registration
		WHO VR (recalculated - unadjusted)	1989-2019	1	Vital Registration
Morocco	Middle East and North Africa	Demographic and Health Survey	1992	0	Sibling survival histories
		World Health Survey	2003	0	Sibling survival histories
		Demographic and Health Survey	2003-2004	0	Sibling survival histories
		National Demographic Survey with repeated passages	2009-2010	0	Household Deaths
		WHO VR (recalculated - unadjusted)	1991-2015	0	Vital Registration
Mozambique	Eastern and Southern Africa	Census	1997	1	Household Deaths
		Demographic and Health Survey	1997	1	Sibling survival histories
		Demographic and Health Survey	2003	1	Sibling survival histories
		Census	2007	0	Household Deaths
		Demographic and Health Survey	2011	1	Sibling survival histories
		Census	2017	0	Household Deaths
		WHO VR (recalculated - unadjusted)	2019-2020	0	Vital Registration
Myanmar	East Asia and Pacific	Population Change and Fertility Survey	1991	1	Household Deaths
		Census	2014	1	Household Deaths
		Demographic and Health Survey	2015-2016	1	Sibling survival histories
		WHO VR (recalculated - unadjusted)	2014-2019	0	Vital Registration
Namibia	Eastern and Southern Africa	Demographic and Health Survey	1992	1	Sibling survival histories
		Demographic and Health Survey	2000	1	Sibling survival histories
		Census	2001	1	Household Deaths
		World Health Survey	2003	1	Sibling survival histories
		Demographic and Health Survey	2006-2007	1	Sibling survival histories
		Census	2011	1	Household Deaths
		Demographic and Health Survey	2013	1	Sibling survival histories
Nauru	East Asia and Pacific	WHO VR (recalculated - unadjusted)	1997-2003	0	Vital Registration
Nepal	South Asia	Demographic and Health Survey	1996	1	Sibling survival histories
		Census	2001	1	Household Deaths
		World Health Survey	2003	1	Sibling survival histories
		Demographic and Health Survey	2006	1	Sibling survival histories
		Census	2011	1	Household Deaths
		Demographic and Health Survey	2016	1	Sibling survival histories
Netherlands	Western Europe	WHO VR (recalculated - unadjusted)	1950-1989	0	Vital Registration
		WHO VR (recalculated - unadjusted)	1989-2020	1	Vital Registration
New Zealand	East Asia and Pacific	Census	2006	1	Household Deaths

Country	Region	Series	Years	Inclusion	Type
		WHO VR (recalculated - unadjusted)	1950-1989	0	Vital Registration
		WHO VR (recalculated - unadjusted)	1989-2017	1	Vital Registration
Nicaragua	Latin America and Caribbean	Census	1995	0	Household Deaths
		Demographic and Health Survey 2001	2001	0	Household Deaths
		Living Standards Measurement Survey	2001	0	Household Deaths
		Census	2005	0	Household Deaths
		WHO VR (recalculated - unadjusted)	1959-2019	0	Vital Registration
Niger	West and Central Africa	Demographic and Health Survey	1992	1	Sibling survival histories
		Census	2001	1	Household Deaths
		Demographic and Health Survey	2006	1	Sibling survival histories
		Demographic and Health Survey	2012	1	Sibling survival histories
Nigeria	West and Central Africa	Rural Demographic Sample Survey	1965-1966	1	Household Deaths
		Demographic and Health Survey	1999	0	Sibling survival histories
		Demographic and Health Survey	2008	1	Sibling survival histories
		GHS Panel Survey	2010	1	Household Deaths
		GHS Panel Survey	2012-2013	1	Household Deaths
		Demographic and Health Survey	2013	1	Sibling survival histories
		Demographic and Health Survey	2018	1	Sibling survival histories
North Macedonia	Eastern Europe and Central Asia	WHO VR (recalculated - unadjusted)	1982-1989	0	Vital Registration
		WHO VR (recalculated - unadjusted)	1989-2020	1	Vital Registration
Norway	Western Europe	WHO VR (recalculated - unadjusted)	1950-1989	0	Vital Registration
		WHO VR (recalculated - unadjusted)	1989-2020	1	Vital Registration
Oman	Middle East and North Africa	WHO VR (recalculated - unadjusted)	2009-2020	0	Vital Registration
Pakistan	South Asia	Population Growth Estimation Experiment	1962-1965	0	Household Deaths
		World Health Survey	2003	0	Sibling survival histories
		Pakistan Demographic Survey	1984-2008	1	Sample Vital Registration
Palau	East Asia and Pacific	WHO VR (recalculated - unadjusted)	1985-2007	1	Vital Registration
Panama	Latin America and Caribbean	TABLAS DE VIDA NACIONALES	0	0	Unknown
		Census	2000	0	Household Deaths
		WHO VR (recalculated - unadjusted)	1955-2020	0	Vital Registration
		WHO VR (recalculated - adjusted for incompleteness)	1990-2020	1	Vital Registration
Papua New Guinea	East Asia and Pacific	Demographic and Health Survey	2018	0	Sibling survival histories
		WHO VR (recalculated - unadjusted)	1977-1999	0	Vital Registration
Paraguay	Latin America and Caribbean	Census	2002	0	Household Deaths
		World Health Survey	2003	0	Sibling survival histories
		WHO VR (recalculated - unadjusted)	1980-2018	0	Vital Registration
Peru	Latin America and Caribbean	Demographic and Health Survey	1991-1992	1	Sibling survival histories
		Demographic and Health Survey	1996	1	Sibling survival histories
		Demographic and Health Survey	2000	1	Sibling survival histories
		Demographic and Health Survey	2006	1	Sibling survival histories
		Demographic and Health Survey	2008	1	Sibling survival histories
		Demographic and Health Survey	2009	0	Sibling survival histories
		Demographic and Health Survey	2010	0	Sibling survival histories
		Demographic and Health Survey	2011	1	Sibling survival histories
		Demographic and Health Survey	2012	0	Sibling survival histories
		WHO VR (recalculated - unadjusted)	1966-2018	0	Vital Registration
Philippines	East Asia and Pacific	National Demographic and Health Survey	1993	1	Sibling survival histories

Country	Region	Series	Years	Inclusion	Type
		National Demographic and Health Survey	1998	1	Sibling survival histories
		World Health Survey	2003	0	Sibling survival histories
		WHO VR (recalculated - unadjusted)	1963-1993	0	Vital Registration
		WHO VR (recalculated - unadjusted)	1993-2017	1	Vital Registration
Poland	Western Europe	WHO VR (recalculated - unadjusted)	1958-1989	0	Vital Registration
		WHO VR (recalculated - unadjusted)	1989-2020	1	Vital Registration
Portugal	Western Europe	Census	2011	1	Household Deaths
		WHO VR (recalculated - unadjusted)	1950-1989	0	Vital Registration
		WHO VR (recalculated - unadjusted)	1989-2020	1	Vital Registration
Qatar	Middle East and North Africa	WHO VR (recalculated - unadjusted)	1981-1990	0	Vital Registration
		WHO VR (recalculated - unadjusted)	1990-2018	1	Vital Registration
Republic of Korea	East Asia and Pacific	National Life Table 1970-2015	2017	0	Others
		WHO VR (recalculated - unadjusted)	1980-1989	0	Vital Registration
		WHO VR (recalculated - unadjusted)	1989-2019	1	Vital Registration
Republic of Moldova	Eastern Europe and Central Asia	WHO VR (recalculated - unadjusted)	1981-1989	0	Vital Registration
		WHO VR (recalculated - unadjusted)	1989-2020	1	Vital Registration
Romania	Eastern Europe and Central Asia	WHO VR (recalculated - unadjusted)	1963-1989	0	Vital Registration
		WHO VR (recalculated - unadjusted)	1989-2020	1	Vital Registration
Russian Federation	Eastern Europe and Central Asia	WHO VR (recalculated - unadjusted)	1959-1989	0	Vital Registration
		WHO VR (recalculated - unadjusted)	1989-2020	1	Vital Registration
Rwanda	Eastern and Southern Africa	Demographic and Health Survey	2000	1	Sibling survival histories
		Demographic and Health Survey	2000	0	Sibling survival histories
		Census	2002	1	Household Deaths
		Demographic and Health Survey	2005	1	Sibling survival histories
		Demographic and Health Survey	2010	1	Sibling survival histories
		Census	2012	1	Household Deaths
		Demographic and Health Survey	2014-2015	1	Sibling survival histories
Saint Kitts and Nevis	Latin America and Caribbean	WHO VR (recalculated - unadjusted)	1970-1989	0	Vital Registration
		WHO VR (recalculated - unadjusted)	1989-2017	1	Vital Registration
Saint Lucia	Latin America and Caribbean	WHO VR (recalculated - unadjusted)	1971-1989	0	Vital Registration
		WHO VR (recalculated - unadjusted)	1989-2019	1	Vital Registration
Saint Vincent and the Grenadines	Latin America and Caribbean	WHO VR (recalculated - unadjusted)	1970-1988	0	Vital Registration
		WHO VR (recalculated - unadjusted)	1988-2018	1	Vital Registration
Samoa	East Asia and Pacific	Demographic and Health Survey	1999	0	Household Deaths
		Demographic and Vital Statistics Survey	2000	0	Household Deaths
		Population and Housing Census	2011	0	Household Deaths
		Population and Housing Census	2016	0	Household Deaths
San Marino	Western Europe	WHO VR (recalculated - unadjusted)	1980-2012	0	Vital Registration
		WHO VR (recalculated - unadjusted)	2014-2019	0	Vital Registration
Sao Tome and Principe	West and Central Africa	Demographic and Health Survey	2008-2009	1	Sibling survival histories
		Census	2012	1	Household Deaths
		Multiple Indicator Cluster Survey	2014	1	Sibling survival histories
		WHO VR (recalculated - unadjusted)	1984-1988	0	Vital Registration
Saudi Arabia	Middle East and North Africa	Demographic Survey	1999	1	Household Deaths
		Census	2004	1	Household Deaths
		Demographic Survey	2007	1	Household Deaths
		Census	2010	1	Household Deaths

Country	Region	Series	Years	Inclusion	Type
		Demographic Survey	2016	0	Household Deaths
		WHO VR (recalculated - unadjusted)	2009-2013	0	Vital Registration
Senegal	West and Central Africa	Demographic and Health Survey	1992-1993	1	Sibling survival histories
		Census	2002	1	Household Deaths
		World Health Survey	2003	0	Sibling survival histories
		Demographic and Health Survey	2005	1	Sibling survival histories
		Demographic and Health Survey	2010-2011	1	Sibling survival histories
		Census	2013	1	Household Deaths
Serbia	Eastern Europe and Central Asia	WHO VR (recalculated - unadjusted)	1985-1989	0	Vital Registration
		WHO VR (recalculated - unadjusted)	1989-2018	1	Vital Registration
Seychelles	Eastern and Southern Africa	Census	1960	1	Household Deaths
		WHO VR (recalculated - unadjusted)	1968-1989	0	Vital Registration
		WHO VR (recalculated - unadjusted)	1989-2017	1	Vital Registration
Sierra Leone	West and Central Africa	Census	2004	1	Household Deaths
		Demographic and Health Survey	2008	1	Sibling survival histories
		Demographic and Health Survey	2013	1	Sibling survival histories
		Census	2015	1	Household Deaths
Singapore	East Asia and Pacific	WHO VR (recalculated - unadjusted)	1955-1989	0	Vital Registration
		WHO VR (recalculated - unadjusted)	1989-2020	1	Vital Registration
Slovakia	Western Europe	WHO VR (recalculated - unadjusted)	1950-1989	0	Vital Registration
		WHO VR (recalculated - unadjusted)	1989-2020	1	Vital Registration
Slovenia	Western Europe	WHO VR (recalculated - unadjusted)	1982-1989	0	Vital Registration
		WHO VR (recalculated - unadjusted)	1989-2019	1	Vital Registration
Solomon Islands	East Asia and Pacific	WHO VR (recalculated - unadjusted)	2017-2019	0	Vital Registration
South Africa	Eastern and Southern Africa	Household Survey	1993	0	Household Deaths
		Household Survey	1995	0	Household Deaths
		Household Survey	1996	0	Household Deaths
		Household Survey	1997	0	Household Deaths
		Household Survey	1998	0	Household Deaths
		Demographic and Health Survey	1998	1	Sibling survival histories
		Census	2001	1	Household Deaths
		World Health Survey	2003	1	Sibling survival histories
		Community Survey	2007	0	Household Deaths
		Census	2011	1	Household Deaths
		Demographic and Health Survey	2016	1	Sibling survival histories
		WHO VR (recalculated - unadjusted)	1980-2016	0	Vital Registration
		WHO VR (recalculated - adjusted for incompleteness)	1991-2016	0	Vital Registration
		Rapid Mortality Surveillance	2020	1	Others
Spain	Western Europe	WHO VR (recalculated - unadjusted)	1950-1989	0	Vital Registration
		WHO VR (recalculated - unadjusted)	1989-2019	1	Vital Registration
Sri Lanka	South Asia	World Health Survey	2003	1	Sibling survival histories
		WHO VR (recalculated - unadjusted)	1950-1989	0	Vital Registration
		WHO VR (recalculated - unadjusted)	1989-2015	1	Vital Registration
State of Palestine	Middle East and North Africa	Census	2017	0	Household Deaths
		WHO VR (recalculated - unadjusted)	2008-2018	0	Vital Registration
Sudan	Eastern and Southern Africa	Demographic and Health Survey	1989-1990	0	Sibling survival histories
		Census	1993	0	Household Deaths

Country	Region	Series	Years	Inclusion	Type
		Census	2008	0	Household Deaths
Suriname	Latin America and Caribbean	WHO VR (recalculated - unadjusted)	1971-2015	0	Vital Registration
		WHO VR (recalculated - adjusted for incompleteness)	1989-2015	1	Vital Registration
Sweden	Western Europe	WHO VR (recalculated - unadjusted)	1950-1989	0	Vital Registration
		WHO VR (recalculated - unadjusted)	1989-2020	1	Vital Registration
Switzerland	Western Europe	WHO VR (recalculated - unadjusted)	1950-1989	0	Vital Registration
		WHO VR (recalculated - unadjusted)	1989-2019	1	Vital Registration
Syrian Arab Republic	Middle East and North Africa	PAPCHILD Maternal and Child Health Survey	1993	1	Household Deaths
		WHO VR (recalculated - unadjusted)	1983-1985	0	Vital Registration
		WHO VR (recalculated - unadjusted)	1998-2011	1	Vital Registration
Tajikistan	Eastern Europe and Central Asia	WHO VR (recalculated - unadjusted)	1981-2000	0	Vital Registration
		WHO VR (recalculated - unadjusted)	2000-2006	1	Vital Registration
		WHO VR (recalculated - adjusted for incompleteness)	1989-2000	1	Vital Registration
		WHO VR (recalculated - adjusted for incompleteness)	2000-2006	0	Vital Registration
Thailand	East Asia and Pacific	National Life Tables	0	0	Unknown
		Survey of Population Change	1995-1996	1	Household Deaths
		Survey of Population Change	2005-2006	1	Household Deaths
		WHO VR (recalculated - unadjusted)	1955-2019	0	Vital Registration
		WHO VR (recalculated - adjusted for incompleteness)	1990-2019	1	Vital Registration
Timor-Leste	East Asia and Pacific	Demographic and Health Survey	1997	1	Sibling survival histories
		Demographic and Health Survey	2009-2010	1	Sibling survival histories
		Census	2015	1	Household Deaths
		Demographic and Health Survey	2016	1	Sibling survival histories
Togo	West and Central Africa	Demographic survey	1961	1	Household Deaths
		Census	1981	1	Household Deaths
		Demographic and Health Survey	1998	1	Sibling survival histories
		Census	2010	1	Household Deaths
		Demographic and Health Survey	2013-2014	1	Sibling survival histories
Tonga	East Asia and Pacific	Census	2006	1	Household Deaths
		WHO VR (recalculated - unadjusted)	1992-2019	1	Vital Registration
Trinidad and Tobago	Latin America and Caribbean	WHO VR (recalculated - unadjusted)	1951-1989	0	Vital Registration
		WHO VR (recalculated - unadjusted)	1989-2013	1	Vital Registration
Tunisia	Middle East and North Africa	National Demographic Survey	1968-1969	0	Household Deaths
		World Health Survey	2003	1	Sibling survival histories
		WHO VR (recalculated - unadjusted)	1980-2014	0	Vital Registration
		WHO VR (recalculated - unadjusted)	2015-2018	1	Vital Registration
		WHO VR (recalculated - adjusted for incompleteness)	1994-2001	1	Vital Registration
		WHO VR (recalculated - adjusted for incompleteness)	2009-2018	0	Vital Registration
Turkey	Eastern Europe and Central Asia	National Verbal Autopsy Survey	2003	1	Household Deaths
		WHO VR (recalculated - unadjusted)	1999-2009	0	Vital Registration
		WHO VR (recalculated - unadjusted)	2009-2020	1	Vital Registration
Turkmenistan	Eastern Europe and Central Asia	WHO VR (recalculated - unadjusted)	1981-2016	0	Vital Registration
		WHO VR (recalculated - adjusted for incompleteness)	1989-2016	1	Vital Registration
Tuvalu	East Asia and Pacific	WHO VR (recalculated - unadjusted)	1991-2006	0	Vital Registration
Uganda	Eastern and Southern Africa	Demographic and Health Survey	1995	1	Sibling survival histories
		Demographic and Health Survey	2000-2001	1	Sibling survival histories
		Census	2002	1	Household Deaths

Country	Region	Series	Years	Inclusion	Type
		Demographic and Health Survey	2006	1	Sibling survival histories
		Demographic and Health Survey	2011	1	Sibling survival histories
		Census	2014	1	Household Deaths
		Demographic and Health Survey	2016	1	Sibling survival histories
Ukraine	Eastern Europe and Central Asia	World Health Survey	2003	0	Sibling survival histories
		WHO VR (recalculated - unadjusted)	1959-1989	0	Vital Registration
		WHO VR (recalculated - unadjusted)	1989-2020	1	Vital Registration
United Arab Emirates	Middle East and North Africa	World Health Survey	2003	0	Sibling survival histories
		WHO VR (recalculated - unadjusted)	2003-2018	0	Vital Registration
United Kingdom	Western Europe	WHO VR (recalculated - unadjusted)	1950-1989	0	Vital Registration
		WHO VR (recalculated - unadjusted)	1989-2019	1	Vital Registration
United Republic of Tanzania	Eastern and Southern Africa	Census	1967	1	Household Deaths
		National Demographic Survey	1973	0	Household Deaths
		Census	1988	1	Household Deaths
		Demographic and Health Survey	1996	1	Sibling survival histories
		Census	2002	1	Household Deaths
		Demographic and Health Survey	2004-2005	1	Sibling survival histories
		Demographic and Health Survey	2010	1	Sibling survival histories
		National Panel Survey	2012-2013	1	Household Deaths
		Demographic and Health Survey	2015-2016	1	Sibling survival histories
United States of America	North America	WHO VR (recalculated - unadjusted)	1950-1989	0	Vital Registration
		WHO VR (recalculated - unadjusted)	1989-2018	1	Vital Registration
Uruguay	Latin America and Caribbean	World Health Survey	2003	0	Sibling survival histories
		WHO VR (recalculated - unadjusted)	1955-1989	0	Vital Registration
		WHO VR (recalculated - unadjusted)	1989-2020	1	Vital Registration
Uzbekistan	Eastern Europe and Central Asia	WHO VR (recalculated - unadjusted)	1981-2017	0	Vital Registration
		WHO VR (recalculated - adjusted for incompleteness)	1989-2017	1	Vital Registration
Venezuela (Bolivarian Republic of)	Latin America and Caribbean	WHO VR (recalculated - unadjusted)	1955-2017	0	Vital Registration
		WHO VR (recalculated - unadjusted)	1990-2001	1	Vital Registration
		WHO VR (recalculated - adjusted for incompleteness)	2001-2017	1	Vital Registration
Viet Nam	East Asia and Pacific	Census	1989	1	Household Deaths
		Census	1999	1	Household Deaths
		World Health Survey	2003	1	Sibling survival histories
		Population Change and Family Planning Survey	2007	1	Household Deaths
		Population Change and Family Planning Survey	2008	1	Household Deaths
		Population Change and Family Planning Survey	2013	1	Household Deaths
Zambia	Eastern and Southern Africa	Demographic and Health Survey	1996	1	Sibling survival histories
		Demographic and Health Survey	2001-2002	1	Sibling survival histories
		World Health Survey	2003	1	Sibling survival histories
		Demographic and Health Survey	2007	1	Sibling survival histories
		Census	2010	1	Household Deaths
		Demographic and Health Survey	2013-2014	1	Sibling survival histories
		Demographic and Health Survey	2018	1	Sibling survival histories
Zimbabwe	Eastern and Southern Africa	Census	1992	1	Household Deaths
		Demographic and Health Survey	1994	1	Sibling survival histories
		Inter-Censal Demographic Survey	1997	1	Household Deaths
		Demographic and Health Survey	1999	1	Sibling survival histories

Country	Region	Series	Years	Inclusion	Type
		Census	2002	1	Household Deaths
		World Health Survey	2003	1	Sibling survival histories
		Demographic and Health Survey	2005-2006	1	Sibling survival histories
		Demographic and Health Survey	2010-2011	1	Sibling survival histories
		Census	2012	1	Household Deaths
		Multiple Indicator Cluster Survey	2014	1	Sibling survival histories
		Demographic and Health Survey	2015	1	Sibling survival histories
		Inter-Censal Demographic Survey	2017	1	Household Deaths
		WHO VR (recalculated - unadjusted)	1982-2003	0	Vital Registration

Table S7 – *Data series included in the analysis*

4 Mortality levels and numbers of deaths in 1990 and 2019

Table S8 presents the estimates of the probability of dying among youth aged 15-24 (deaths per 1,000 youths aged 15), and Table S9 presents the estimates of the probability of dying among adolescents aged 15-19 (deaths per 1,000 adolescents aged 15). Tables S10 and S11 present the numbers of deaths among youth aged 15-24 and adolescents aged 15-19 in 1990 and 2019.

Country	1990			2019		
	$_{10}q_{15}$	Lower bound	Upper bound	$_{10}q_{15}$	Lower bound	Upper bound
Afghanistan	49.7	23.8	136.0	30.9	17.6	54.8
Albania	8.0	7.7	8.3	3.5	3.2	3.9
Algeria	10.6	9.0	12.5	5.9	5.7	6.1
Andorra	8.8	6.9	11.2	3.4	2.6	4.3
Angola	70.7	55.1	90.0	32.0	24.9	40.7
Antigua and Barbuda	13.7	6.4	30.0	5.6	3.7	8.6
Argentina	8.6	8.4	8.8	8.3	7.8	8.8
Armenia	6.9	6.5	7.2	4.4	3.4	5.3
Australia	7.7	7.5	7.9	3.4	3.2	3.7
Austria	7.9	7.6	8.2	2.8	2.4	3.3
Azerbaijan	9.1	8.8	9.3	5.7	5.1	6.5
Bahamas	12.3	10.7	14.2	17.3	12.9	23.2
Bahrain	6.1	5.4	6.8	4.8	3.5	6.5
Bangladesh	23.7	21.5	26.4	11.7	8.6	16.1
Barbados	10.0	8.9	11.3	6.0	3.8	9.5
Belarus	11.4	11.1	11.6	3.8	3.4	4.2
Belgium	7.6	7.4	7.8	2.8	2.6	3.1
Belize	9.4	7.6	11.6	11.4	9.6	13.5
Benin	28.7	23.1	35.5	22.2	15.5	33.1
Bhutan	30.0	17.3	51.4	12.2	6.3	23.0
Bolivia (Plurin. State of)	23.4	19.1	28.0	9.8	4.8	20.0
Bosnia and Herz.	6.5	6.2	6.8	4.2	3.4	5.3
Botswana	38.3	28.9	50.9	15.2	7.7	29.0
Brazil	13.4	13.1	13.7	13.6	12.2	15.1
Brunei Darussalam	14.0	11.7	16.8	3.9	3.2	4.7
Bulgaria	8.1	7.9	8.3	5.3	5.0	5.7
Burkina Faso	37.0	30.8	44.1	16.0	8.4	29.8
Burundi	59.8	34.2	99.9	17.5	10.6	30.1
Cabo Verde	9.2	8.0	10.7	6.1	4.1	9.1
Cambodia	37.3	29.1	47.1	8.2	4.5	14.8
Cameroon	30.5	25.6	35.9	29.8	21.4	41.1
Canada	7.5	7.3	7.7	4.6	4.4	4.9
Central Afr. Rep.	45.1	36.8	55.3	34.7	14.6	82.4
Chad	46.8	38.5	57.0	34.8	21.9	54.3
Chile	8.4	8.2	8.6	5.0	3.9	6.4

Country	1990 $_{10}q_{15}$	1990 Lower bound	1990 Upper bound	2019 $_{10}q_{15}$	2019 Lower bound	2019 Upper bound
China	8.7	7.1	11.1	3.8	2.3	6.3
Colombia	26.3	22.3	31.1	11.3	10.9	11.6
Comoros	24.1	16.4	38.3	9.0	4.6	17.9
Congo	53.7	30.6	89.4	16.3	9.0	29.2
Cook Islands	17.3	13.7	21.8	9.4	4.7	19.4
Costa Rica	6.4	6.1	6.7	7.8	7.3	8.3
Côte d'Ivoire	31.0	25.3	37.6	26.4	14.5	47.9
Croatia	7.8	7.4	8.2	3.4	2.9	3.9
Cuba	10.5	10.3	10.8	4.8	4.5	5.0
Cyprus	6.4	5.8	7.1	3.0	2.4	3.8
Czechia	6.9	6.7	7.1	3.9	3.7	4.2
Dem. People's Rep. of Korea	14.7	11.5	18.8	9.2	7.2	11.7
Dem. Rep. of the Congo	45.8	27.6	77.9	37.3	22.6	63.7
Denmark	5.2	5.0	5.5	2.4	2.1	2.6
Djibouti	42.8	33.3	54.7	27.1	21.1	34.6
Dominica	6.4	5.5	7.6	6.0	3.6	10.0
Dominican Republic	13.1	8.8	18.5	12.6	6.8	22.3
Ecuador	14.3	13.8	14.8	10.2	5.3	19.7
Egypt	12.4	12.1	12.7	7.4	7.1	7.6
El Salvador	27.3	26.5	28.0	24.4	20.3	29.2
Equatorial Guinea	40.0	31.3	51.4	24.9	19.5	32.0
Eritrea	50.6	39.5	64.6	21.7	16.9	27.7
Estonia	13.9	13.2	14.6	4.2	3.6	4.8
Eswatini	29.9	23.3	38.3	25.3	19.8	32.4
Ethiopia	91.8	74.0	113.2	16.5	10.6	25.4
Fiji	16.4	9.3	29.8	9.9	8.4	11.8
Finland	8.3	8.0	8.7	4.9	4.5	5.3
France	7.7	7.6	7.9	3.2	3.0	3.4
Gabon	23.1	16.3	32.2	15.1	7.2	29.9
Gambia	38.6	30.2	49.5	18.8	14.7	24.2
Georgia	9.4	9.1	9.8	7.0	6.3	7.8
Germany	6.5	6.4	6.6	2.6	2.5	2.8
Ghana	30.1	18.6	50.6	15.1	8.5	27.0
Greece	6.9	6.7	7.1	3.0	2.7	3.4
Grenada	10.8	8.9	13.0	5.8	4.2	8.2
Guatemala	20.1	19.6	20.6	14.9	13.3	16.6
Guinea	33.7	27.2	42.1	31.5	19.4	50.7
Guinea-Bissau	45.6	35.5	58.6	24.2	18.8	31.1
Guyana	14.3	13.6	15.1	17.2	12.5	23.6
Haiti	40.7	31.7	52.7	18.5	12.0	28.0
Honduras	18.4	14.5	23.6	10.0	7.9	12.8
Hungary	8.5	8.3	8.7	3.4	3.1	3.8
Iceland	6.9	5.9	8.0	2.9	2.2	3.8

Country	1990 $_{10}q_{15}$	1990 Lower bound	1990 Upper bound	2019 $_{10}q_{15}$	2019 Lower bound	2019 Upper bound
India	24.3	22.0	26.9	9.9	7.5	13.1
Indonesia	12.5	10.3	15.0	10.1	5.6	18.0
Iran (Islamic Rep. of)	23.2	15.6	34.5	10.0	9.0	11.2
Iraq	10.9	8.5	13.9	9.5	7.4	12.2
Ireland	6.2	6.0	6.5	2.4	2.0	2.8
Israel	5.2	5.0	5.4	2.8	2.6	3.0
Italy	6.4	6.2	6.5	2.6	2.3	3.1
Jamaica	13.4	10.5	17.1	9.1	7.1	11.6
Japan	4.8	4.7	4.9	2.6	2.4	2.7
Jordan	8.1	6.1	10.8	5.6	5.2	6.0
Kazakhstan	13.8	13.5	14.1	7.4	7.1	7.6
Kenya	23.1	18.7	28.4	16.0	9.3	26.8
Kiribati	22.0	17.2	28.2	16.0	12.5	20.4
Kuwait	11.9	10.9	13.0	6.1	5.2	7.4
Kyrgyzstan	12.0	11.7	12.4	6.4	6.1	6.7
Lao People's Dem. Rep.	27.6	14.8	46.8	12.1	6.6	22.5
Latvia	14.6	14.0	15.2	6.1	5.3	7.0
Lebanon	21.9	17.1	27.9	5.1	4.0	6.5
Lesotho	30.1	16.6	48.6	27.7	17.3	44.4
Liberia	61.2	23.3	178.1	28.7	15.4	52.0
Libya	9.9	7.7	12.7	10.1	7.9	13.0
Lithuania	12.4	11.9	12.8	5.5	5.0	6.1
Luxembourg	9.6	8.4	10.9	1.7	1.2	2.3
Madagascar	31.1	25.4	37.9	22.6	11.4	45.7
Malawi	40.5	34.0	47.6	18.6	11.2	29.9
Malaysia	10.7	10.0	11.4	6.5	6.1	6.9
Maldives	12.8	11.6	14.2	4.7	3.8	5.8
Mali	36.3	30.1	44.2	21.8	15.6	31.4
Malta	4.5	3.9	5.3	2.5	1.8	3.6
Marshall Islands	15.7	12.3	20.1	12.5	9.8	16.1
Mauritania	31.2	24.3	40.0	23.3	18.2	29.8
Mauritius	8.5	8.0	9.0	8.0	7.3	8.7
Mexico	11.5	11.2	11.8	11.5	10.7	12.4
Micronesia (Fed. States of)	16.6	12.9	21.1	12.1	9.4	15.4
Monaco	6.3	4.9	8.0	3.5	2.7	4.4
Mongolia	15.3	13.2	17.5	8.8	8.2	9.5
Montenegro	6.4	5.7	7.1	3.4	2.7	4.3
Morocco	12.7	9.9	16.2	7.7	6.0	9.8
Mozambique	37.9	30.1	47.5	30.7	16.5	57.2
Myanmar	26.7	17.9	44.6	9.1	5.4	15.1
Namibia	27.4	22.4	32.6	21.3	12.2	36.7
Nauru	17.4	13.6	22.3	12.3	9.6	15.8
Nepal	20.4	16.8	24.9	10.4	6.7	16.2

Country	1990 $_{10}q_{15}$	1990 Lower bound	1990 Upper bound	2019 $_{10}q_{15}$	2019 Lower bound	2019 Upper bound
Netherlands	4.6	4.5	4.8	2.5	2.3	2.6
New Zealand	11.4	11.0	11.8	4.2	3.4	5.2
Nicaragua	19.7	15.4	25.2	9.9	7.8	12.7
Niger	39.6	31.5	48.2	27.1	15.1	48.4
Nigeria	37.6	27.3	54.1	18.9	14.4	25.0
Niue	8.0	6.3	10.3	10.6	8.4	13.7
North Macedonia	5.3	5.0	5.6	2.9	2.5	3.2
Norway	6.0	5.7	6.4	3.3	3.0	3.6
Oman	9.7	7.6	12.3	6.1	4.7	7.7
Pakistan	21.8	18.5	25.8	11.5	6.1	22.0
Palau	16.1	12.9	20.1	16.6	7.8	35.5
Panama	11.3	10.7	11.9	9.2	8.7	9.7
Papua New Guinea	20.7	16.2	26.4	14.9	11.7	19.1
Paraguay	16.3	12.7	20.9	10.7	8.4	13.8
Peru	19.9	17.4	23.4	6.5	3.5	12.2
Philippines	14.3	13.0	15.7	9.0	7.6	10.7
Poland	8.6	8.4	8.8	4.6	4.4	4.7
Portugal	11.0	10.8	11.3	2.7	2.5	2.9
Qatar	7.1	6.0	8.4	3.1	2.5	3.9
Republic of Korea	8.8	8.6	9.0	2.7	2.5	2.9
Republic of Moldova	12.7	12.3	13.1	7.3	6.8	7.8
Romania	8.7	8.5	8.9	4.6	4.4	4.9
Russian Federation	13.8	13.4	14.2	7.5	7.2	7.8
Rwanda	70.5	54.1	92.0	15.2	8.1	29.6
Saint Kitts and Nevis	9.1	6.8	12.0	16.1	10.9	24.1
Saint Lucia	11.4	9.9	13.1	9.2	7.1	11.9
Saint Vincent and the Gr.	8.8	7.6	10.1	13.8	10.3	18.3
Samoa	12.1	9.5	15.5	8.5	6.7	10.9
San Marino	9.2	7.2	11.7	2.3	1.8	3.0
Sao Tome and Principe	25.0	7.1	83.3	17.8	8.6	34.4
Saudi Arabia	15.3	5.1	92.3	12.1	5.8	25.1
Senegal	25.0	20.5	30.3	19.1	11.6	31.6
Serbia	6.2	6.0	6.5	3.3	2.7	4.1
Seychelles	8.5	7.0	10.5	11.6	7.9	16.9
Sierra Leone	61.1	29.7	271.8	52.1	31.8	83.2
Singapore	5.9	5.6	6.2	2.1	1.9	2.4
Slovakia	7.0	6.8	7.2	4.2	3.9	4.5
Slovenia	8.6	8.2	9.1	3.2	2.7	3.8
Solomon Islands	13.8	10.8	17.7	9.8	7.7	12.5
Somalia	66.7	52.1	85.6	42.6	33.3	54.7
South Africa	21.3	16.2	27.8	20.0	17.2	23.5
South Sudan	69.3	53.8	88.8	37.7	29.3	48.3
Spain	8.4	8.2	8.6	2.3	2.1	2.5

Country	1990 $_{10}q_{15}$	1990 Lower bound	1990 Upper bound	2019 $_{10}q_{15}$	2019 Lower bound	2019 Upper bound
Sri Lanka	27.6	26.5	28.6	4.0	3.0	5.4
State of Palestine	10.2	7.9	13.0	7.4	5.8	9.5
Sudan	52.3	40.9	67.2	27.4	21.4	35.1
Suriname	14.8	13.7	16.1	7.8	5.5	11.0
Sweden	5.1	4.9	5.3	3.5	3.3	3.7
Switzerland	8.6	8.3	8.8	2.5	2.2	2.9
Syrian Arab Republic	14.6	10.0	22.5	16.8	8.4	32.9
Tajikistan	10.7	10.4	11.0	4.5	2.3	8.8
Thailand	15.6	15.1	16.1	12.7	11.9	13.5
Timor-Leste	21.9	10.3	44.0	26.9	13.4	54.4
Togo	24.6	19.4	31.5	17.7	10.0	30.2
Tonga	6.8	4.7	9.9	8.1	6.2	10.6
Trinidad and Tobago	10.8	10.2	11.4	10.5	6.6	16.8
Tunisia	8.6	6.9	10.8	7.5	6.7	8.5
Turkey	27.9	6.6	114.8	4.3	4.1	4.4
Turkmenistan	12.7	12.4	13.1	10.2	8.0	13.0
Tuvalu	16.3	12.8	21.0	10.8	8.5	13.9
Uganda	47.7	40.3	56.4	27.2	17.7	43.2
Ukraine	11.5	11.2	11.8	6.1	5.9	6.4
United Arab Emirates	7.0	5.4	9.0	5.2	4.0	6.6
United Kingdom	5.7	5.5	5.8	3.2	3.0	3.4
United Rep. of Tanzania	28.4	22.8	34.7	17.7	10.5	31.4
United States	9.8	9.6	10.1	7.8	7.0	8.7
Uruguay	8.2	7.8	8.5	9.0	8.4	9.5
Uzbekistan	11.2	10.9	11.4	8.0	6.7	9.6
Vanuatu	13.2	10.3	16.9	11.3	8.8	14.4
Venezuela (Bolivarian Rep. of)	12.3	11.9	12.7	26.2	22.1	31.0
Viet Nam	14.2	9.4	20.4	6.6	3.6	12.4
Yemen	15.2	11.9	19.4	25.1	19.6	32.0
Zambia	48.7	41.5	57.2	21.7	15.0	30.7
Zimbabwe	21.6	18.2	25.4	24.0	15.8	35.9

Table S8 – *Probability of dying among youth aged 15-24 (deaths per 1,000 youths aged 15)*

Country	1990			2019		
	$_5q_{15}$	Lower bound	Upper bound	$_5q_{15}$	Lower bound	Upper bound
Afghanistan	16.9	11.5	24.5	13.6	9.1	19.3
Albania	3.3	3.1	3.5	1.7	1.5	1.9
Algeria	5.0	4.6	5.5	2.7	2.6	2.8
Andorra	3.6	2.8	4.6	1.3	1.1	1.7
Angola	28.7	22.4	36.6	12.8	10.0	16.3
Antigua and Barbuda	6.9	3.7	10.5	2.4	1.6	3.6
Argentina	3.7	3.6	3.7	3.4	3.3	3.6
Armenia	2.8	2.6	3.0	2.0	1.8	2.3
Australia	3.3	3.2	3.3	1.5	1.4	1.5
Austria	3.5	3.4	3.7	1.2	1.1	1.4
Azerbaijan	3.7	3.5	3.8	2.6	2.3	2.8
Bahamas	4.1	3.3	4.9	4.8	3.5	6.2
Bahrain	3.0	2.6	3.4	2.0	1.6	2.5
Bangladesh	9.9	8.2	11.2	6.5	4.8	8.8
Barbados	4.4	3.7	5.2	2.3	1.5	3.2
Belarus	4.2	4.1	4.4	1.5	1.3	1.6
Belgium	3.1	3.0	3.2	1.0	1.0	1.1
Belize	3.1	2.3	3.9	4.0	3.2	4.9
Benin	14.2	10.8	18.8	11.2	7.5	16.3
Bhutan	16.4	10.1	23.9	5.5	3.1	8.9
Bolivia (Plurin. State of)	11.4	9.3	14.0	4.6	2.6	6.9
Bosnia and Herz.	2.6	2.4	2.8	1.7	1.4	2.0
Botswana	13.2	9.6	17.8	3.9	2.2	6.5
Brazil	5.4	5.3	5.5	5.8	5.4	6.2
Brunei Darussalam	6.8	5.6	8.1	1.3	1.0	1.7
Bulgaria	3.4	3.3	3.6	2.4	2.2	2.6
Burkina Faso	18.0	14.8	22.0	6.1	3.5	9.3
Burundi	24.0	17.7	34.2	8.7	5.8	12.2
Cabo Verde	3.4	2.8	4.2	2.3	1.7	3.0
Cambodia	17.9	13.3	24.0	3.5	2.1	5.7
Cameroon	13.4	11.1	16.0	12.3	8.5	17.9
Canada	3.4	3.3	3.5	1.7	1.6	1.8
Central Afr. Rep.	17.9	14.2	22.8	11.3	5.6	19.8
Chad	25.8	20.0	33.1	15.5	9.9	23.0
Chile	3.3	3.3	3.4	2.1	1.8	2.4
China	5.0	4.3	5.9	1.3	1.0	1.8
Colombia	9.7	8.6	10.8	4.5	4.3	4.6
Comoros	12.7	8.9	18.1	3.3	1.6	5.6
Congo	24.6	13.0	39.5	5.4	2.7	9.5
Cook Islands	7.7	5.5	10.8	5.8	3.4	8.2
Costa Rica	2.7	2.6	2.9	2.9	2.7	3.1
Côte d'Ivoire	12.4	9.5	16.0	11.1	6.2	17.9
Croatia	3.0	2.8	3.2	1.4	1.2	1.6

Country	1990 $_5q_{15}$	1990 Lower bound	1990 Upper bound	2019 $_5q_{15}$	2019 Lower bound	2019 Upper bound
Cuba	4.6	4.5	4.7	2.0	1.9	2.1
Cyprus	2.9	2.5	3.3	1.2	0.9	1.5
Czechia	2.8	2.7	3.0	1.5	1.4	1.6
Dem. People's Rep. of Korea	6.5	5.1	8.3	4.0	3.1	5.1
Dem. Rep. of the Congo	19.5	10.4	28.4	18.0	11.3	26.7
Denmark	2.2	2.0	2.3	1.0	0.9	1.1
Djibouti	17.3	13.5	22.1	10.8	8.4	13.7
Dominica	2.6	2.0	3.3	1.9	1.0	3.1
Dominican Republic	6.2	4.0	8.9	4.2	2.5	6.6
Ecuador	5.9	5.7	6.1	3.8	2.3	5.7
Egypt	5.8	5.7	6.0	3.5	3.4	3.6
El Salvador	9.8	9.5	10.1	11.7	10.6	12.8
Equatorial Guinea	17.8	13.9	22.9	10.9	8.5	14.0
Eritrea	20.6	16.0	26.2	8.5	6.7	10.9
Estonia	5.8	5.4	6.2	1.9	1.6	2.2
Eswatini	11.9	9.3	15.3	10.0	7.8	12.9
Ethiopia	38.6	29.1	50.8	8.0	4.9	11.6
Fiji	6.7	3.0	10.1	3.7	2.9	4.5
Finland	3.5	3.3	3.7	1.8	1.7	2.0
France	2.8	2.7	2.9	1.2	1.1	1.2
Gabon	9.1	5.6	14.6	7.0	3.1	12.5
Gambia	17.2	13.4	22.0	8.1	6.4	10.4
Georgia	3.4	3.2	3.6	2.8	2.5	3.2
Germany	2.8	2.8	2.9	1.1	1.1	1.2
Ghana	11.9	6.3	20.6	7.4	4.6	11.1
Greece	2.7	2.5	2.8	1.2	1.0	1.3
Grenada	3.9	3.0	5.1	2.5	1.6	3.6
Guatemala	7.7	7.5	7.9	6.3	5.9	6.8
Guinea	18.3	13.9	23.7	13.3	8.4	19.5
Guinea-Bissau	20.5	15.9	26.3	10.6	8.2	13.6
Guyana	5.4	4.9	5.8	5.8	4.6	7.2
Haiti	17.9	12.6	24.7	6.8	4.1	11.1
Honduras	7.5	5.9	9.6	4.0	3.1	5.1
Hungary	3.4	3.3	3.5	1.4	1.3	1.5
Iceland	3.5	2.9	4.2	1.5	1.1	1.9
India	10.5	9.7	11.5	4.0	3.3	4.6
Indonesia	6.6	5.4	8.1	4.7	3.1	6.8
Iran (Islamic Rep. of)	11.4	8.6	14.5	4.7	4.4	5.0
Iraq	5.1	4.0	6.6	4.3	3.4	5.5
Ireland	2.4	2.3	2.6	0.8	0.7	1.0
Israel	2.2	2.1	2.3	1.2	1.1	1.3
Italy	2.8	2.7	2.8	1.1	1.0	1.1
Jamaica	5.4	4.2	6.8	3.6	2.8	4.6

Country	1990 $_5q_{15}$	1990 Lower bound	1990 Upper bound	2019 $_5q_{15}$	2019 Lower bound	2019 Upper bound
Japan	2.2	2.1	2.2	0.9	0.9	1.0
Jordan	3.7	2.6	5.2	2.3	2.1	2.5
Kazakhstan	5.4	5.3	5.5	3.0	2.9	3.1
Kenya	8.9	6.8	11.6	6.3	4.0	9.4
Kiribati	9.9	7.8	12.7	7.1	5.5	9.1
Kuwait	5.0	4.4	5.6	2.0	1.5	2.6
Kyrgyzstan	4.4	4.2	4.6	2.7	2.5	2.9
Lao People's Dem. Rep.	16.6	5.5	26.4	4.6	2.4	9.5
Latvia	6.2	5.9	6.6	2.5	2.1	2.9
Lebanon	8.8	6.8	11.2	2.3	1.8	2.9
Lesotho	13.2	8.6	20.2	10.2	6.0	15.9
Liberia	18.3	3.6	60.6	18.5	9.9	26.8
Libya	4.6	3.6	5.9	4.3	3.4	5.5
Lithuania	5.1	4.9	5.4	2.3	2.0	2.5
Luxembourg	4.2	3.5	5.1	0.8	0.6	1.1
Madagascar	14.2	10.8	18.4	10.7	6.0	16.7
Malawi	18.8	15.3	22.5	7.0	4.6	10.2
Malaysia	4.3	4.1	4.6	3.0	2.9	3.1
Maldives	5.5	4.8	6.4	2.4	1.9	2.9
Mali	17.9	14.4	22.6	10.0	6.8	14.5
Malta	2.0	1.6	2.4	1.1	0.7	1.5
Marshall Islands	7.0	5.5	9.0	5.5	4.3	7.1
Mauritania	13.7	10.7	17.6	10.1	7.9	12.9
Mauritius	3.6	3.3	3.9	3.0	2.7	3.4
Mexico	4.6	4.5	4.7	4.4	4.2	4.5
Micronesia (Fed. States of)	7.4	5.7	9.4	5.3	4.1	6.8
Monaco	2.6	2.0	3.3	1.4	1.1	1.8
Mongolia	5.6	5.2	6.1	3.8	3.5	4.0
Montenegro	2.4	2.0	2.7	1.2	0.9	1.6
Morocco	6.0	4.7	7.7	3.5	2.8	4.5
Mozambique	19.0	13.8	25.7	9.6	4.8	16.8
Myanmar	14.2	9.1	23.1	3.3	2.0	5.2
Namibia	11.2	9.1	14.0	7.4	4.6	11.0
Nauru	7.8	6.1	10.0	5.4	4.3	7.0
Nepal	10.1	7.8	13.2	4.5	2.8	6.9
Netherlands	2.0	2.0	2.1	1.0	1.0	1.1
New Zealand	5.0	4.8	5.3	1.8	1.5	2.1
Nicaragua	8.0	6.2	10.2	4.0	3.1	5.1
Niger	19.5	15.2	24.7	10.3	6.0	16.3
Nigeria	17.1	11.9	23.6	8.5	6.1	12.0
Niue	3.5	2.7	4.5	4.7	3.7	6.0
North Macedonia	2.3	2.1	2.5	1.3	1.1	1.5
Norway	2.8	2.7	3.0	1.2	1.1	1.4

Country	1990			2019		
	$_5q_{15}$	Lower bound	Upper bound	$_5q_{15}$	Lower bound	Upper bound
Oman	4.5	3.5	5.7	2.8	2.2	3.5
Pakistan	10.6	8.8	12.7	6.0	3.6	9.2
Palau	6.0	4.2	8.6	6.5	3.2	11.0
Panama	4.7	4.4	5.0	3.3	3.1	3.6
Papua New Guinea	9.3	7.3	11.9	6.6	5.2	8.5
Paraguay	6.6	5.2	8.5	4.3	3.4	5.5
Peru	9.4	7.9	11.0	2.3	1.3	3.6
Philippines	5.4	5.0	5.8	3.4	3.0	3.7
Poland	3.5	3.4	3.6	1.8	1.7	1.8
Portugal	4.7	4.6	4.9	1.1	1.0	1.2
Qatar	3.7	3.1	4.6	1.7	1.3	2.0
Republic of Korea	3.8	3.7	3.9	1.1	1.0	1.2
Republic of Moldova	4.7	4.5	4.9	3.0	2.8	3.3
Romania	3.6	3.5	3.7	2.1	2.0	2.2
Russian Federation	5.5	5.4	5.6	2.9	2.8	2.9
Rwanda	34.3	25.8	45.8	5.9	3.5	8.9
Saint Kitts and Nevis	4.1	2.8	5.7	6.0	4.0	8.7
Saint Lucia	4.4	3.6	5.3	4.2	3.1	5.4
Saint Vincent and the Gr.	3.7	3.0	4.4	6.0	4.4	7.9
Samoa	5.3	4.2	6.8	3.7	2.9	4.7
San Marino	3.7	2.9	4.8	0.9	0.7	1.2
Sao Tome and Principe	10.0	2.6	21.1	6.1	3.1	10.3
Saudi Arabia	7.8	2.3	13.6	5.3	2.9	8.4
Senegal	11.8	9.2	15.1	10.1	6.7	14.1
Serbia	2.7	2.6	2.8	1.4	1.3	1.6
Seychelles	2.6	1.9	3.5	10.3	9.0	11.1
Sierra Leone	32.0	16.2	52.4	24.9	15.9	35.7
Singapore	2.4	2.2	2.6	0.9	0.8	1.1
Slovakia	2.9	2.8	3.0	1.7	1.6	1.9
Slovenia	3.3	3.0	3.6	1.3	1.1	1.6
Solomon Islands	6.1	4.8	7.8	4.3	3.3	5.5
Somalia	26.7	20.8	34.3	17.2	13.4	22.1
South Africa	8.0	5.9	11.2	7.6	6.8	8.6
South Sudan	28.5	22.2	36.6	15.1	11.8	19.4
Spain	3.3	3.2	3.3	0.9	0.8	1.0
Sri Lanka	9.4	9.1	9.7	1.6	1.3	1.8
State of Palestine	4.8	3.7	6.1	3.4	2.7	4.4
Sudan	20.9	16.4	26.8	10.9	8.5	14.0
Suriname	5.9	5.2	6.7	3.2	2.5	4.1
Sweden	2.3	2.2	2.4	1.2	1.1	1.3
Switzerland	3.2	3.0	3.3	1.1	1.0	1.2
Syrian Arab Republic	8.5	6.5	10.4	7.1	5.5	8.7
Tajikistan	4.3	4.2	4.5	1.6	1.1	2.3

Country	1990 $_5q_{15}$	1990 Lower bound	1990 Upper bound	2019 $_5q_{15}$	2019 Lower bound	2019 Upper bound
Thailand	6.8	6.6	7.1	6.1	5.8	6.4
Timor-Leste	9.3	4.8	16.6	15.0	9.3	21.8
Togo	11.8	9.2	15.4	7.6	4.7	11.2
Tonga	2.4	1.5	3.6	3.1	2.2	4.2
Trinidad and Tobago	4.0	3.8	4.4	3.6	2.7	4.5
Tunisia	3.7	3.1	4.3	3.1	2.8	3.4
Turkey	14.8	5.4	23.1	2.0	1.9	2.0
Turkmenistan	5.1	4.9	5.3	4.5	3.9	5.1
Tuvalu	7.3	5.7	9.3	4.7	3.7	6.1
Uganda	18.5	15.0	22.8	13.4	9.1	18.8
Ukraine	4.4	4.3	4.5	2.4	2.3	2.5
United Arab Emirates	3.2	2.5	4.1	2.3	1.8	3.0
United Kingdom	2.6	2.5	2.6	1.3	1.2	1.4
United Rep. of Tanzania	13.3	10.9	16.1	7.0	4.8	10.2
United States	4.4	4.3	4.5	2.6	2.5	2.8
Uruguay	3.9	3.7	4.1	3.5	3.2	3.8
Uzbekistan	4.4	4.3	4.5	3.7	3.3	4.0
Vanuatu	5.8	4.6	7.5	5.0	3.9	6.3
Venezuela (Bolivarian Rep. of)	5.2	5.0	5.4	10.7	9.6	11.8
Viet Nam	6.4	4.2	9.4	3.0	1.8	4.8
Yemen	7.2	5.6	9.2	10.7	8.4	13.7
Zambia	18.5	15.0	23.0	9.3	6.5	13.3
Zimbabwe	7.5	6.1	9.2	9.7	7.0	13.3

Table S9 – *Probability of dying among adolescents aged 15-19 (deaths per 1,000 adolescents aged 15)*

Country	1990 Deaths (15-24)	1990 Lower bound	1990 Upper bound	2019 Deaths (15-24)	2019 Lower bound	2019 Upper bound
Afghanistan	11.6	5.9	31.7	25.8	15.3	44.9
Albania	0.5	0.5	0.5	0.2	0.1	0.2
Algeria	5.6	4.8	6.5	3.6	3.4	3.7
Andorra	0.0	0.0	0.0	0.0	0.0	0.0
Angola	15.8	12.2	20.3	19.7	15.3	25.2
Antigua and Barbuda	0.0	0.0	0.0	0.0	0.0	0.0
Argentina	4.6	4.5	4.7	5.8	5.5	6.2
Armenia	0.4	0.4	0.4	0.2	0.1	0.2
Australia	2.1	2.1	2.2	1.1	1.0	1.2
Austria	0.9	0.9	1.0	0.3	0.2	0.3
Azerbaijan	1.2	1.2	1.2	0.8	0.7	0.9
Bahamas	0.1	0.1	0.1	0.1	0.1	0.2
Bahrain	0.0	0.0	0.1	0.1	0.1	0.1
Bangladesh	50.6	46.0	56.1	36.3	26.9	50.0
Barbados	0.0	0.0	0.1	0.0	0.0	0.0
Belarus	1.6	1.5	1.6	0.3	0.3	0.4
Belgium	1.1	1.0	1.1	0.4	0.3	0.4
Belize	0.0	0.0	0.0	0.1	0.1	0.1
Benin	2.7	2.2	3.3	5.3	3.8	7.7
Bhutan	0.3	0.2	0.5	0.2	0.1	0.3
Bolivia (Plurin. State of)	3.1	2.6	3.7	2.2	1.1	4.4
Bosnia and Herz.	0.5	0.5	0.5	0.2	0.1	0.2
Botswana	1.0	0.8	1.3	0.6	0.3	1.2
Brazil	39.0	38.1	39.9	46.2	41.3	51.5
Brunei Darussalam	0.1	0.1	0.1	0.0	0.0	0.0
Bulgaria	1.0	1.0	1.0	0.3	0.3	0.4
Burkina Faso	6.4	5.4	7.6	6.4	3.5	11.8
Burundi	5.9	3.4	9.9	3.9	2.4	6.5
Cabo Verde	0.1	0.1	0.1	0.1	0.0	0.1
Cambodia	6.4	5.1	8.1	2.5	1.3	4.6
Cameroon	6.7	5.7	7.8	15.3	11.2	20.9
Canada	3.0	3.0	3.1	2.1	2.0	2.2
Central Afr. Rep.	2.4	2.0	2.9	3.5	1.6	8.4
Chad	5.3	4.4	6.3	11.5	7.5	17.7
Chile	2.1	2.0	2.1	1.4	1.1	1.8
China	222.2	179.3	285.1	66.4	39.3	110.2
Colombia	17.7	15.0	21.0	9.9	9.6	10.2
Comoros	0.2	0.1	0.3	0.1	0.1	0.3
Congo	2.6	1.6	4.3	1.6	0.9	2.8
Cook Islands	0.0	0.0	0.0	0.0	0.0	0.0
Costa Rica	0.4	0.3	0.4	0.6	0.6	0.7
Côte d'Ivoire	6.7	5.5	8.0	14.0	7.9	25.3
Croatia	0.5	0.5	0.5	0.2	0.1	0.2

Country	1990 Deaths (15-24)	1990 Lower bound	1990 Upper bound	2019 Deaths (15-24)	2019 Lower bound	2019 Upper bound
Cuba	2.4	2.3	2.4	0.7	0.6	0.7
Cyprus	0.1	0.1	0.1	0.1	0.0	0.1
Czechia	1.0	1.0	1.1	0.4	0.4	0.4
Dem. People's Rep. of Korea	6.9	5.4	8.8	3.6	2.8	4.6
Dem. Rep. of the Congo	30.2	18.9	50.6	62.7	39.2	105.0
Denmark	0.4	0.4	0.4	0.2	0.2	0.2
Djibouti	0.5	0.4	0.6	0.5	0.4	0.6
Dominica	0.0	0.0	0.0	0.0	0.0	0.0
Dominican Republic	1.9	1.3	2.7	2.4	1.3	4.3
Ecuador	3.0	2.9	3.1	3.2	1.7	6.2
Egypt	12.7	12.4	13.0	12.6	12.1	13.0
El Salvador	2.8	2.7	2.9	3.1	2.5	3.7
Equatorial Guinea	0.3	0.2	0.4	0.6	0.5	0.8
Eritrea	2.2	1.7	2.8	1.4	1.1	1.7
Estonia	0.3	0.3	0.3	0.1	0.0	0.1
Eswatini	0.5	0.4	0.6	0.6	0.5	0.8
Ethiopia	83.3	68.1	102.3	40.2	26.4	61.4
Fiji	0.2	0.1	0.4	0.1	0.1	0.2
Finland	0.6	0.5	0.6	0.3	0.3	0.3
France	6.6	6.5	6.8	2.4	2.2	2.6
Gabon	0.4	0.3	0.5	0.6	0.3	1.1
Gambia	0.7	0.6	0.9	0.9	0.7	1.1
Georgia	0.8	0.8	0.8	0.3	0.3	0.4
Germany	7.3	7.1	7.5	2.3	2.2	2.5
Ghana	9.3	6.0	15.4	8.9	5.2	15.8
Greece	1.1	1.1	1.1	0.3	0.3	0.4
Grenada	0.0	0.0	0.0	0.0	0.0	0.0
Guatemala	3.6	3.5	3.7	5.5	5.0	6.1
Guinea	4.1	3.4	5.0	8.6	5.5	13.8
Guinea-Bissau	0.8	0.6	1.0	0.9	0.7	1.2
Guyana	0.2	0.2	0.2	0.3	0.2	0.4
Haiti	5.3	4.2	6.8	4.0	2.7	6.1
Honduras	1.8	1.4	2.3	2.0	1.6	2.6
Hungary	1.2	1.2	1.3	0.4	0.3	0.4
Iceland	0.0	0.0	0.0	0.0	0.0	0.0
India	406.8	369.7	448.1	246.5	185.1	323.4
Indonesia	48.0	39.9	57.1	46.3	25.9	82.4
Iran (Islamic Rep. of)	24.6	17.1	35.7	11.3	10.1	12.6
Iraq	4.0	3.1	5.1	7.4	5.8	9.5
Ireland	0.4	0.4	0.4	0.1	0.1	0.2
Israel	0.4	0.4	0.4	0.3	0.3	0.4
Italy	5.7	5.6	5.9	1.5	1.4	1.8
Jamaica	0.7	0.5	0.9	0.5	0.4	0.6

Country	1990 Deaths (15-24)	1990 Lower bound	1990 Upper bound	2019 Deaths (15-24)	2019 Lower bound	2019 Upper bound
Japan	9.1	8.9	9.3	3.0	2.9	3.2
Jordan	0.6	0.5	0.8	1.1	1.0	1.2
Kazakhstan	3.8	3.7	3.9	1.7	1.6	1.7
Kenya	10.8	8.9	13.1	17.4	10.3	28.7
Kiribati	0.0	0.0	0.0	0.0	0.0	0.0
Kuwait	0.4	0.4	0.5	0.3	0.2	0.3
Kyrgyzstan	0.9	0.9	1.0	0.7	0.6	0.7
Lao People's Dem. Rep.	2.4	1.4	4.0	1.7	0.9	3.2
Latvia	0.5	0.5	0.6	0.1	0.1	0.1
Lebanon	1.3	1.0	1.7	0.6	0.5	0.8
Lesotho	1.0	0.6	1.6	1.2	0.7	1.9
Liberia	2.4	1.0	7.2	2.9	1.7	5.1
Libya	0.9	0.7	1.2	1.1	0.9	1.4
Lithuania	0.7	0.7	0.7	0.2	0.1	0.2
Luxembourg	0.0	0.0	0.1	0.0	0.0	0.0
Madagascar	7.1	5.9	8.6	12.6	6.6	25.2
Malawi	7.3	6.2	8.5	7.1	4.5	11.3
Malaysia	3.7	3.5	3.9	3.7	3.4	3.9
Maldives	0.1	0.0	0.1	0.0	0.0	0.1
Mali	6.0	5.1	7.2	8.4	6.3	11.8
Malta	0.0	0.0	0.0	0.0	0.0	0.0
Marshall Islands	0.0	0.0	0.0	0.0	0.0	0.0
Mauritania	1.3	1.0	1.6	2.0	1.6	2.6
Mauritius	0.2	0.2	0.2	0.2	0.1	0.2
Mexico	20.3	19.8	20.7	25.6	23.7	27.6
Micronesia (Fed. States of)	0.0	0.0	0.0	0.0	0.0	0.0
Monaco	0.0	0.0	0.0	0.0	0.0	0.0
Mongolia	0.7	0.6	0.8	0.4	0.4	0.4
Montenegro	0.1	0.1	0.1	0.0	0.0	0.0
Morocco	6.5	5.1	8.3	4.5	3.5	5.8
Mozambique	9.7	7.9	11.9	18.7	10.3	34.5
Myanmar	23.2	16.0	38.2	8.9	5.3	14.6
Namibia	0.8	0.7	1.0	1.1	0.6	1.8
Nauru	0.0	0.0	0.0	0.0	0.0	0.0
Nepal	7.3	6.1	8.8	6.7	4.3	10.4
Netherlands	1.1	1.1	1.1	0.5	0.5	0.5
New Zealand	0.6	0.6	0.7	0.3	0.2	0.3
Nicaragua	1.6	1.2	2.1	1.2	0.9	1.5
Niger	5.7	4.6	6.9	12.0	7.0	21.1
Nigeria	67.7	50.9	95.4	73.3	57.0	94.9
Niue	0.0	0.0	0.0	0.0	0.0	0.0
North Macedonia	0.2	0.2	0.2	0.1	0.1	0.1
Norway	0.4	0.4	0.4	0.2	0.2	0.3

Country	1990 Deaths (15-24)	1990 Lower bound	1990 Upper bound	2019 Deaths (15-24)	2019 Lower bound	2019 Upper bound
Oman	0.3	0.2	0.4	0.4	0.3	0.5
Pakistan	44.2	37.8	51.9	49.2	26.3	93.3
Palau	0.0	0.0	0.0	0.0	0.0	0.0
Panama	0.6	0.5	0.6	0.6	0.6	0.7
Papua New Guinea	1.9	1.5	2.4	2.6	2.0	3.3
Paraguay	1.3	1.0	1.7	1.4	1.1	1.9
Peru	9.0	7.9	10.5	3.4	1.8	6.4
Philippines	17.7	16.2	19.3	18.4	15.5	21.9
Poland	4.6	4.5	4.7	1.8	1.7	1.8
Portugal	1.8	1.7	1.8	0.3	0.3	0.3
Qatar	0.0	0.0	0.1	0.1	0.1	0.1
Republic of Korea	7.8	7.6	8.0	1.7	1.5	1.8
Republic of Moldova	0.8	0.8	0.8	0.3	0.3	0.4
Romania	3.4	3.3	3.5	0.9	0.9	1.0
Russian Federation	27.1	26.3	27.8	10.3	9.9	10.7
Rwanda	9.9	7.8	12.8	3.7	2.0	7.2
Saint Kitts and Nevis	0.0	0.0	0.0	0.0	0.0	0.0
Saint Lucia	0.0	0.0	0.0	0.0	0.0	0.0
Saint Vincent and the Gr.	0.0	0.0	0.0	0.0	0.0	0.0
Samoa	0.0	0.0	0.1	0.0	0.0	0.0
San Marino	0.0	0.0	0.0	0.0	0.0	0.0
Sao Tome and Principe	0.1	0.0	0.2	0.1	0.0	0.1
Saudi Arabia	4.6	1.4	29.7	5.7	2.6	12.1
Senegal	3.6	3.1	4.3	6.2	3.9	10.0
Serbia	0.9	0.8	0.9	0.4	0.3	0.4
Seychelles	0.0	0.0	0.0	0.0	0.0	0.0
Sierra Leone	5.1	2.7	23.8	8.5	5.3	13.4
Singapore	0.3	0.3	0.4	0.2	0.1	0.2
Slovakia	0.6	0.5	0.6	0.2	0.2	0.3
Slovenia	0.3	0.2	0.3	0.1	0.1	0.1
Solomon Islands	0.1	0.1	0.1	0.1	0.1	0.2
Somalia	9.5	7.4	12.3	13.9	10.8	17.9
South Africa	15.0	11.6	19.3	19.9	17.0	23.4
South Sudan	7.5	5.7	9.7	8.5	6.6	10.9
Spain	5.5	5.4	5.7	1.0	1.0	1.1
Sri Lanka	9.4	9.0	9.7	1.2	0.9	1.7
State of Palestine	0.4	0.3	0.6	0.8	0.6	1.0
Sudan	21.2	16.5	27.4	23.8	18.6	30.7
Suriname	0.1	0.1	0.1	0.1	0.1	0.1
Sweden	0.6	0.6	0.6	0.4	0.4	0.4
Switzerland	0.8	0.8	0.9	0.2	0.2	0.3
Syrian Arab Republic	3.8	2.8	5.7	5.4	2.7	10.6
Tajikistan	1.1	1.0	1.1	0.8	0.4	1.5

Country	1990			2019		
	Deaths (15-24)	Lower bound	Upper bound	Deaths (15-24)	Lower bound	Upper bound
Thailand	18.8	18.2	19.5	11.9	11.2	12.7
Timor-Leste	0.3	0.2	0.7	0.8	0.4	1.5
Togo	1.8	1.5	2.3	2.8	1.6	4.7
Tonga	0.0	0.0	0.0	0.0	0.0	0.0
Trinidad and Tobago	0.2	0.2	0.2	0.2	0.1	0.3
Tunisia	1.4	1.1	1.7	1.2	1.1	1.4
Turkey	29.9	8.5	122.4	5.8	5.6	6.0
Turkmenistan	0.9	0.9	0.9	1.0	0.8	1.2
Tuvalu	0.0	0.0	0.0	0.0	0.0	0.0
Uganda	16.1	13.7	18.9	25.4	17.2	39.3
Ukraine	8.0	7.8	8.2	2.6	2.5	2.7
United Arab Emirates	0.2	0.2	0.3	0.6	0.5	0.8
United Kingdom	4.8	4.7	5.0	2.5	2.4	2.7
United Rep. of Tanzania	14.2	11.6	17.2	19.8	12.2	34.3
United States	37.2	36.3	38.1	34.4	30.7	38.4
Uruguay	0.4	0.4	0.4	0.5	0.4	0.5
Uzbekistan	4.3	4.2	4.4	4.4	3.6	5.3
Vanuatu	0.0	0.0	0.0	0.1	0.0	0.1
Venezuela (Bolivarian Rep. of)	4.8	4.7	5.0	12.1	10.3	14.3
Viet Nam	19.6	13.3	27.9	9.1	4.8	17.4
Yemen	3.4	2.6	4.3	15.3	11.9	19.6
Zambia	7.8	6.7	9.1	8.1	5.8	11.2
Zimbabwe	4.6	3.9	5.4	7.0	4.8	10.4

Table S10 – *Number of deaths among youth aged 15-24 (thousands) in 1990 and 2019*

Country	1990 Deaths (15-19)	1990 Lower bound	1990 Upper bound	2019 Deaths (15-19)	2019 Lower bound	2019 Upper bound
Afghanistan	4.5	3.0	6.5	12.4	8.2	17.6
Albania	0.2	0.2	0.2	0.1	0.1	0.1
Algeria	2.9	2.7	3.2	1.5	1.5	1.6
Andorra	0.0	0.0	0.0	0.0	0.0	0.0
Angola	7.4	5.7	9.4	8.7	6.8	11.1
Antigua and Barbuda	0.0	0.0	0.0	0.0	0.0	0.0
Argentina	2.1	2.0	2.1	2.4	2.3	2.5
Armenia	0.2	0.1	0.2	0.1	0.1	0.1
Australia	0.9	0.9	0.9	0.4	0.4	0.5
Austria	0.4	0.4	0.4	0.1	0.1	0.1
Azerbaijan	0.5	0.5	0.5	0.3	0.3	0.4
Bahamas	0.0	0.0	0.0	0.0	0.0	0.0
Bahrain	0.0	0.0	0.0	0.0	0.0	0.0
Bangladesh	22.4	18.6	25.4	20.3	15.0	27.8
Barbados	0.0	0.0	0.0	0.0	0.0	0.0
Belarus	0.6	0.6	0.6	0.1	0.1	0.1
Belgium	0.4	0.4	0.4	0.1	0.1	0.1
Belize	0.0	0.0	0.0	0.0	0.0	0.0
Benin	1.5	1.1	1.9	2.8	1.9	4.1
Bhutan	0.2	0.1	0.3	0.1	0.0	0.1
Bolivia (Plurin. State of)	1.6	1.3	2.0	1.0	0.6	1.6
Bosnia and Herz.	0.2	0.2	0.2	0.1	0.0	0.1
Botswana	0.4	0.3	0.5	0.2	0.1	0.3
Brazil	16.1	15.8	16.5	19.1	17.8	20.4
Brunei Darussalam	0.0	0.0	0.0	0.0	0.0	0.0
Bulgaria	0.4	0.4	0.5	0.1	0.1	0.2
Burkina Faso	3.4	2.8	4.2	2.7	1.6	4.2
Burundi	2.5	1.8	3.6	2.1	1.4	2.9
Cabo Verde	0.0	0.0	0.0	0.0	0.0	0.0
Cambodia	3.2	2.4	4.4	1.0	0.6	1.7
Cameroon	3.3	2.7	3.9	6.8	4.7	10.0
Canada	1.3	1.3	1.3	0.7	0.7	0.7
Central Afr. Rep.	1.0	0.8	1.3	1.3	0.7	2.3
Chad	3.2	2.4	4.1	5.6	3.6	8.4
Chile	0.8	0.8	0.9	0.5	0.5	0.6
China	126.2	107.2	148.9	22.3	16.0	29.7
Colombia	6.6	5.9	7.3	3.8	3.7	4.0
Comoros	0.1	0.1	0.2	0.1	0.0	0.1
Congo	1.3	0.7	2.1	0.6	0.3	1.1
Cook Islands	0.0	0.0	0.0	0.0	0.0	0.0
Costa Rica	0.2	0.1	0.2	0.2	0.2	0.2
Côte d'Ivoire	2.9	2.2	3.8	6.3	3.5	10.3
Croatia	0.2	0.2	0.2	0.1	0.0	0.1

Country	1990 Deaths (15-19)	1990 Lower bound	1990 Upper bound	2019 Deaths (15-19)	2019 Lower bound	2019 Upper bound
Cuba	1.0	1.0	1.1	0.3	0.2	0.3
Cyprus	0.0	0.0	0.0	0.0	0.0	0.0
Czechia	0.5	0.5	0.5	0.1	0.1	0.2
Dem. People's Rep. of Korea	3.0	2.4	3.9	1.5	1.2	2.0
Dem. Rep. of the Congo	14.0	7.4	20.4	33.1	20.7	49.4
Denmark	0.2	0.1	0.2	0.1	0.1	0.1
Djibouti	0.2	0.2	0.3	0.2	0.2	0.3
Dominica	0.0	0.0	0.0	0.0	0.0	0.0
Dominican Republic	1.0	0.6	1.4	0.8	0.5	1.3
Ecuador	1.3	1.3	1.4	1.2	0.7	1.8
Egypt	6.4	6.3	6.6	6.1	5.9	6.2
El Salvador	1.1	1.1	1.1	1.4	1.3	1.6
Equatorial Guinea	0.1	0.1	0.2	0.3	0.2	0.3
Eritrea	1.0	0.7	1.2	0.6	0.5	0.8
Estonia	0.1	0.1	0.1	0.0	0.0	0.0
Eswatini	0.2	0.2	0.3	0.3	0.2	0.3
Ethiopia	38.5	28.9	51.0	20.6	12.6	29.8
Fiji	0.1	0.0	0.1	0.1	0.0	0.1
Finland	0.2	0.2	0.2	0.1	0.1	0.1
France	2.4	2.3	2.4	0.9	0.8	0.9
Gabon	0.2	0.1	0.3	0.3	0.1	0.5
Gambia	0.3	0.3	0.4	0.4	0.3	0.5
Georgia	0.3	0.3	0.3	0.1	0.1	0.1
Germany	2.5	2.5	2.6	0.9	0.9	1.0
Ghana	4.0	2.1	6.9	4.6	2.8	6.9
Greece	0.4	0.4	0.4	0.1	0.1	0.1
Grenada	0.0	0.0	0.0	0.0	0.0	0.0
Guatemala	1.6	1.5	1.6	2.5	2.3	2.6
Guinea	2.4	1.8	3.1	3.9	2.5	5.8
Guinea-Bissau	0.4	0.3	0.5	0.4	0.3	0.6
Guyana	0.1	0.1	0.1	0.1	0.1	0.1
Haiti	2.5	1.7	3.4	1.6	0.9	2.5
Honduras	0.8	0.6	1.0	0.8	0.7	1.1
Hungary	0.5	0.5	0.5	0.1	0.1	0.1
Iceland	0.0	0.0	0.0	0.0	0.0	0.0
India	186.5	171.3	203.2	99.8	82.9	116.6
Indonesia	26.2	21.4	32.4	22.1	14.3	31.6
Iran (Islamic Rep. of)	13.0	9.8	16.6	5.1	4.8	5.5
Iraq	2.1	1.6	2.7	3.5	2.8	4.5
Ireland	0.2	0.2	0.2	0.1	0.0	0.1
Israel	0.2	0.2	0.2	0.2	0.1	0.2
Italy	2.4	2.3	2.4	0.6	0.6	0.7
Jamaica	0.3	0.2	0.4	0.2	0.1	0.2

Country	1990 Deaths (15-19)	1990 Lower bound	1990 Upper bound	2019 Deaths (15-19)	2019 Lower bound	2019 Upper bound
Japan	4.4	4.3	4.5	1.1	1.0	1.1
Jordan	0.3	0.2	0.4	0.5	0.4	0.5
Kazakhstan	1.5	1.5	1.6	0.6	0.6	0.7
Kenya	4.7	3.6	6.2	7.5	4.7	11.1
Kiribati	0.0	0.0	0.0	0.0	0.0	0.0
Kuwait	0.2	0.2	0.2	0.1	0.1	0.1
Kyrgyzstan	0.4	0.4	0.4	0.3	0.2	0.3
Lao People's Dem. Rep.	1.6	0.5	2.5	0.7	0.3	1.3
Latvia	0.2	0.2	0.2	0.0	0.0	0.0
Lebanon	0.6	0.4	0.7	0.3	0.2	0.3
Lesotho	0.5	0.3	0.8	0.4	0.3	0.7
Liberia	0.8	0.2	2.7	2.0	1.1	2.9
Libya	0.5	0.4	0.6	0.5	0.4	0.6
Lithuania	0.3	0.3	0.3	0.1	0.1	0.1
Luxembourg	0.0	0.0	0.0	0.0	0.0	0.0
Madagascar	3.5	2.7	4.6	6.4	3.6	10.0
Malawi	3.7	3.0	4.4	3.0	1.9	4.3
Malaysia	1.6	1.5	1.7	1.6	1.6	1.7
Maldives	0.0	0.0	0.0	0.0	0.0	0.0
Mali	3.3	2.6	4.2	4.3	2.9	6.3
Malta	0.0	0.0	0.0	0.0	0.0	0.0
Marshall Islands	0.0	0.0	0.0	0.0	0.0	0.0
Mauritania	0.6	0.5	0.8	0.9	0.7	1.2
Mauritius	0.1	0.1	0.1	0.1	0.1	0.1
Mexico	9.0	8.8	9.2	9.8	9.5	10.2
Micronesia (Fed. States of)	0.0	0.0	0.0	0.0	0.0	0.0
Monaco	0.0	0.0	0.0	0.0	0.0	0.0
Mongolia	0.3	0.3	0.3	0.2	0.2	0.2
Montenegro	0.0	0.0	0.0	0.0	0.0	0.0
Morocco	3.2	2.5	4.1	2.1	1.6	2.7
Mozambique	5.5	4.0	7.4	6.6	3.3	11.6
Myanmar	13.0	8.3	21.2	3.4	2.1	5.3
Namibia	0.4	0.3	0.5	0.4	0.2	0.5
Nauru	0.0	0.0	0.0	0.0	0.0	0.0
Nepal	3.9	3.0	5.1	2.9	1.8	4.5
Netherlands	0.4	0.4	0.5	0.2	0.2	0.2
New Zealand	0.3	0.3	0.3	0.1	0.1	0.1
Nicaragua	0.7	0.6	0.9	0.5	0.4	0.6
Niger	3.1	2.4	3.9	5.2	3.0	8.3
Nigeria	34.0	23.7	47.1	36.4	26.0	51.3
Niue	0.0	0.0	0.0	0.0	0.0	0.0
North Macedonia	0.1	0.1	0.1	0.0	0.0	0.0
Norway	0.2	0.2	0.2	0.1	0.1	0.1

Country	1990 Deaths (15-19)	1990 Lower bound	1990 Upper bound	2019 Deaths (15-19)	2019 Lower bound	2019 Upper bound
Oman	0.1	0.1	0.2	0.1	0.1	0.2
Pakistan	22.8	19.0	27.5	26.2	15.8	40.3
Palau	0.0	0.0	0.0	0.0	0.0	0.0
Panama	0.3	0.2	0.3	0.2	0.2	0.3
Papua New Guinea	0.9	0.7	1.2	1.2	0.9	1.5
Paraguay	0.6	0.4	0.7	0.6	0.4	0.7
Peru	4.5	3.8	5.3	1.1	0.6	1.8
Philippines	7.1	6.6	7.7	7.0	6.3	7.7
Poland	2.0	1.9	2.0	0.6	0.6	0.6
Portugal	0.8	0.8	0.8	0.1	0.1	0.1
Qatar	0.0	0.0	0.0	0.0	0.0	0.0
Republic of Korea	3.4	3.3	3.5	0.6	0.5	0.6
Republic of Moldova	0.3	0.3	0.3	0.1	0.1	0.1
Romania	1.4	1.4	1.5	0.4	0.4	0.4
Russian Federation	11.2	11.0	11.5	3.9	3.8	4.0
Rwanda	5.3	4.0	7.1	1.6	0.9	2.3
Saint Kitts and Nevis	0.0	0.0	0.0	0.0	0.0	0.0
Saint Lucia	0.0	0.0	0.0	0.0	0.0	0.0
Saint Vincent and the Gr.	0.0	0.0	0.0	0.0	0.0	0.0
Samoa	0.0	0.0	0.0	0.0	0.0	0.0
San Marino	0.0	0.0	0.0	0.0	0.0	0.0
Sao Tome and Principe	0.0	0.0	0.1	0.0	0.0	0.0
Saudi Arabia	2.3	0.7	4.0	2.4	1.3	3.8
Senegal	1.9	1.5	2.5	3.5	2.3	4.9
Serbia	0.4	0.4	0.4	0.1	0.1	0.2
Seychelles	0.0	0.0	0.0	0.0	0.0	0.0
Sierra Leone	2.9	1.4	4.7	4.3	2.7	6.2
Singapore	0.1	0.1	0.1	0.1	0.0	0.1
Slovakia	0.3	0.2	0.3	0.1	0.1	0.1
Slovenia	0.1	0.1	0.1	0.0	0.0	0.0
Solomon Islands	0.0	0.0	0.1	0.1	0.0	0.1
Somalia	4.1	3.2	5.3	6.1	4.8	7.9
South Africa	6.1	4.5	8.6	7.5	6.6	8.4
South Sudan	3.3	2.6	4.2	3.6	2.8	4.7
Spain	2.2	2.1	2.2	0.4	0.4	0.4
Sri Lanka	3.2	3.1	3.3	0.5	0.4	0.6
State of Palestine	0.2	0.2	0.3	0.4	0.3	0.4
Sudan	9.3	7.3	12.0	10.3	8.0	13.2
Suriname	0.0	0.0	0.1	0.0	0.0	0.0
Sweden	0.3	0.2	0.3	0.1	0.1	0.1
Switzerland	0.3	0.3	0.3	0.1	0.1	0.1
Syrian Arab Republic	2.4	1.9	3.0	2.2	1.7	2.7
Tajikistan	0.5	0.4	0.5	0.3	0.2	0.4

Country	1990			2019		
	Deaths (15-19)	Lower bound	Upper bound	Deaths (15-19)	Lower bound	Upper bound
Thailand	8.5	8.2	8.7	5.4	5.2	5.7
Timor-Leste	0.1	0.1	0.3	0.5	0.3	0.7
Togo	1.0	0.7	1.3	1.3	0.8	1.9
Tonga	0.0	0.0	0.0	0.0	0.0	0.0
Trinidad and Tobago	0.1	0.1	0.1	0.1	0.0	0.1
Tunisia	0.6	0.5	0.7	0.5	0.4	0.5
Turkey	16.8	6.1	26.3	2.7	2.6	2.8
Turkmenistan	0.4	0.4	0.4	0.4	0.4	0.5
Tuvalu	0.0	0.0	0.0	0.0	0.0	0.0
Uganda	6.8	5.5	8.4	13.7	9.3	19.3
Ukraine	3.2	3.1	3.3	0.9	0.9	1.0
United Arab Emirates	0.1	0.1	0.1	0.2	0.1	0.2
United Kingdom	2.0	1.9	2.0	1.0	0.9	1.0
United Rep. of Tanzania	7.3	5.9	8.8	8.8	6.0	12.7
United States	15.8	15.5	16.1	11.3	10.6	12.1
Uruguay	0.2	0.2	0.2	0.2	0.2	0.2
Uzbekistan	1.8	1.8	1.9	1.9	1.7	2.1
Vanuatu	0.0	0.0	0.0	0.0	0.0	0.0
Venezuela (Bolivarian Rep. of)	2.1	2.1	2.2	5.3	4.8	5.9
Viet Nam	9.4	6.1	13.7	3.8	2.3	6.2
Yemen	1.8	1.4	2.3	6.8	5.3	8.7
Zambia	3.3	2.7	4.2	3.8	2.7	5.5
Zimbabwe	1.8	1.5	2.2	3.1	2.3	4.3

Table S11 – *Number of deaths among adolescents aged 15-19 (thousands) in 1990 and 2019*

5 Country-specific trends in the probability $_{10}q_{15}$

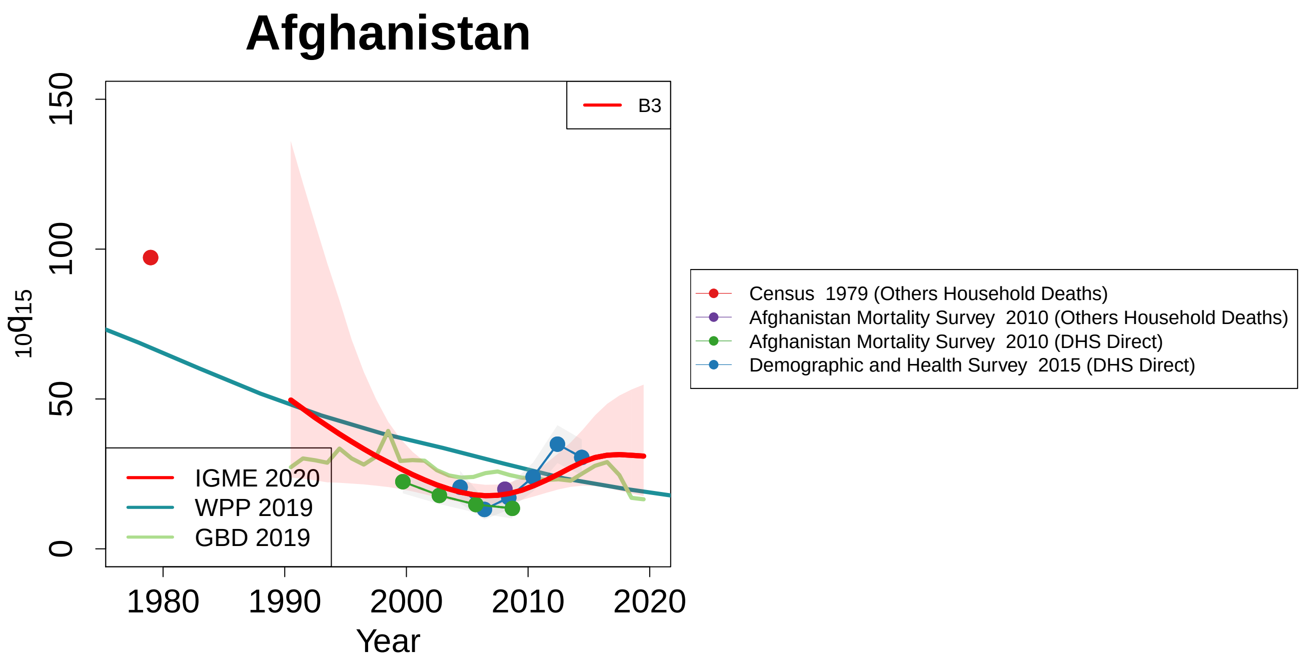

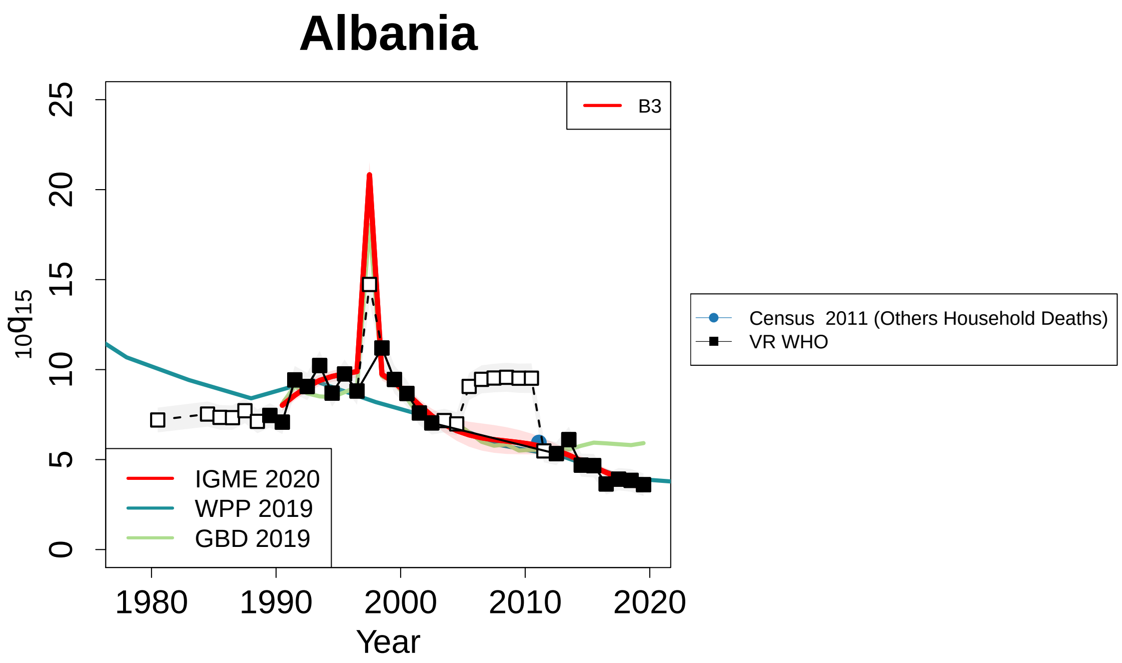

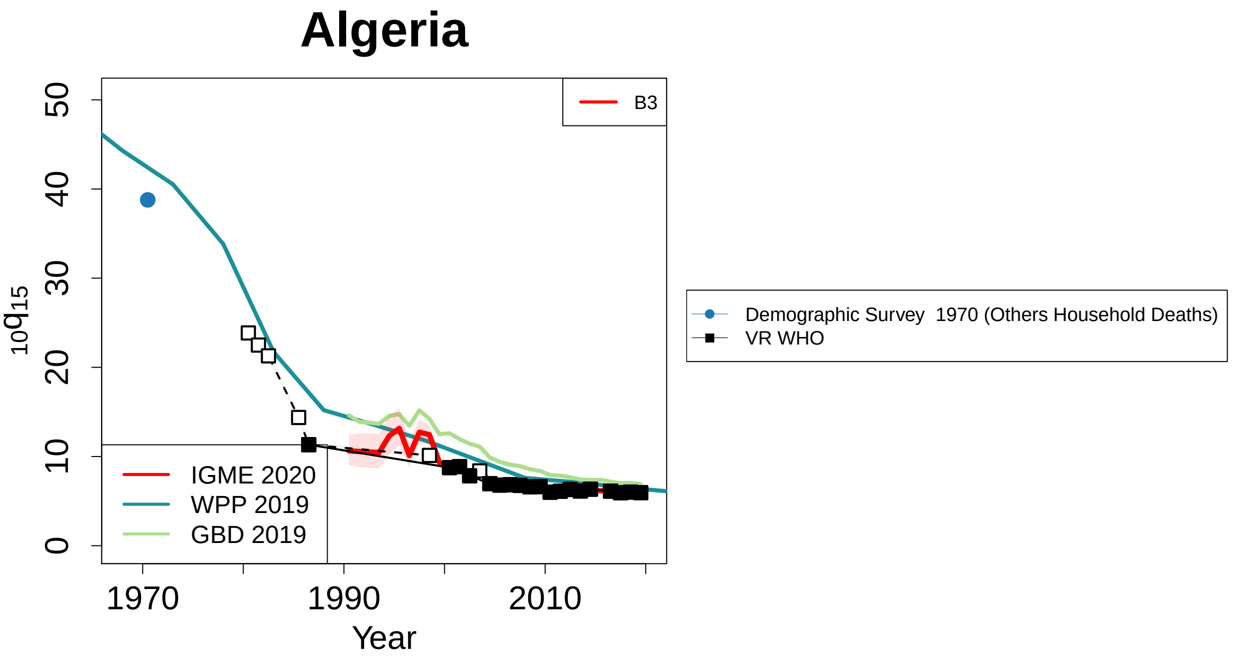
Algeria
B3
Demographic Survey 1970 (Others Household Deaths)
VR WHO
IGME 2020
WPP 2019
GBD 2019
$_{10}q_{15}$
0
10
20
30
40
50
1970
1990
2010
Year

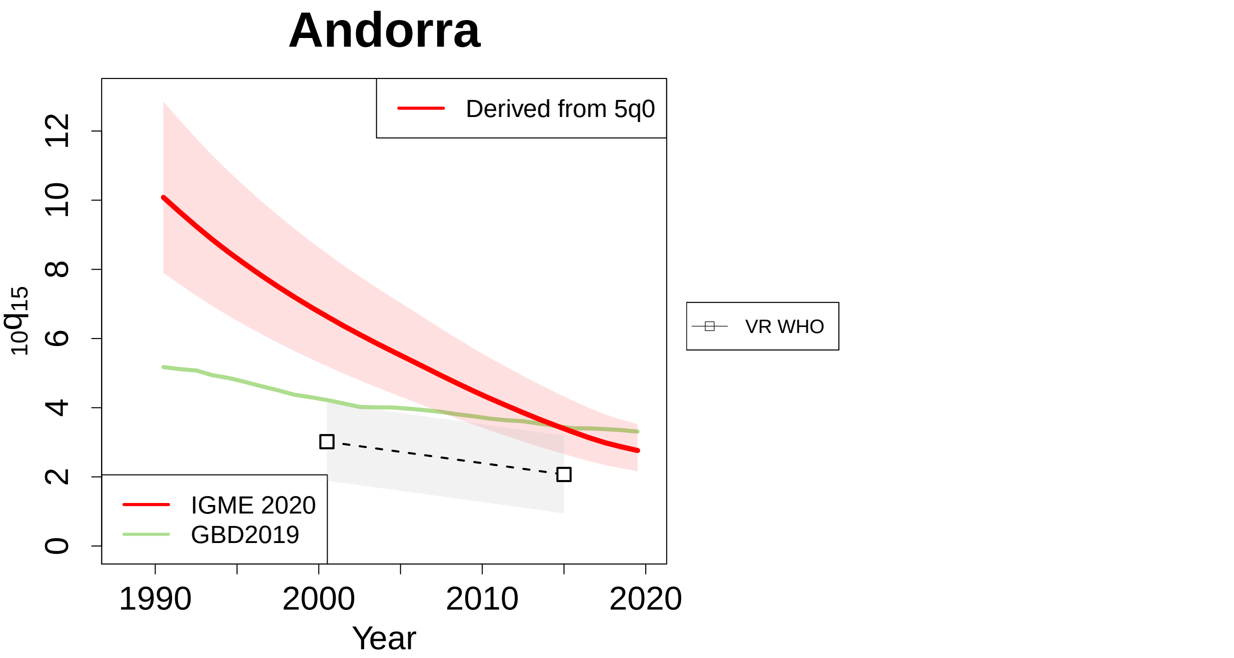
Andorra
Derived from 5q0
VR WHO
IGME 2020
GBD2019
$_{10}q_{15}$
0
2
4
6
8
10
12
1990
2000
2010
2020
Year

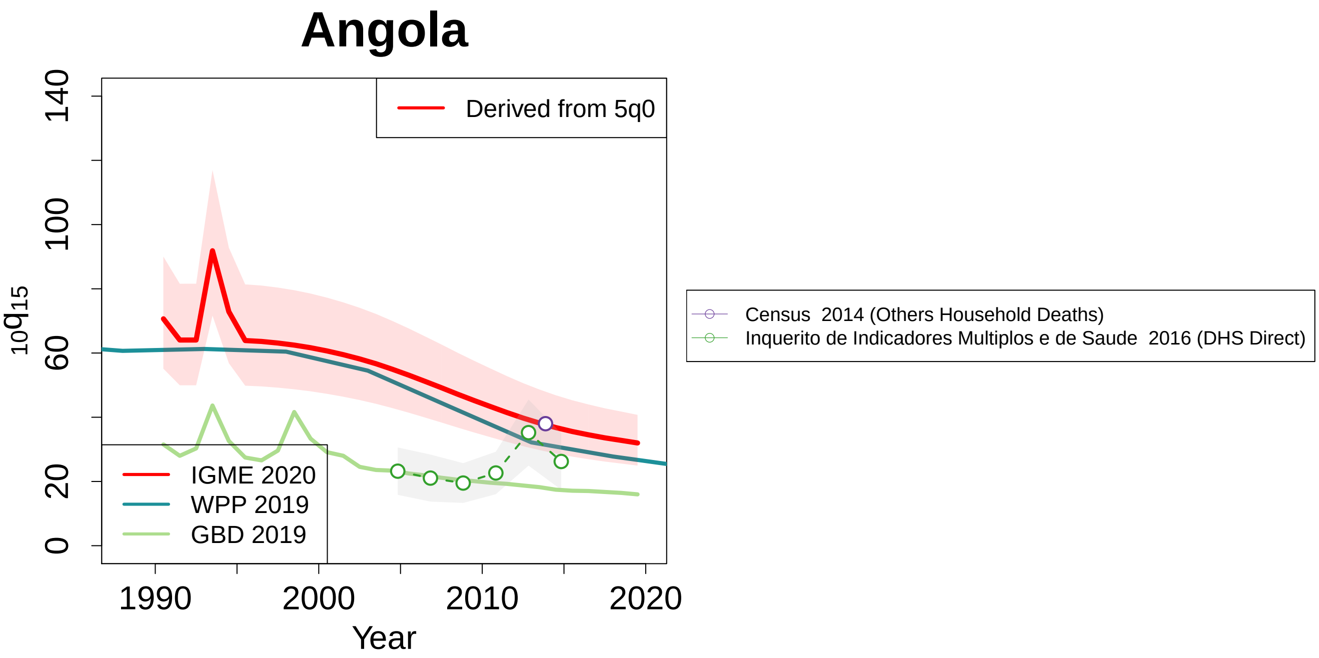
Angola
Derived from 5q0
Census 2014 (Others Household Deaths)
Inquerito de Indicadores Multiplos e de Saude 2016 (DHS Direct)
IGME 2020
WPP 2019
GBD 2019
$_{10}q_{15}$
0
20
60
100
140
1990
2000
2010
2020
Year

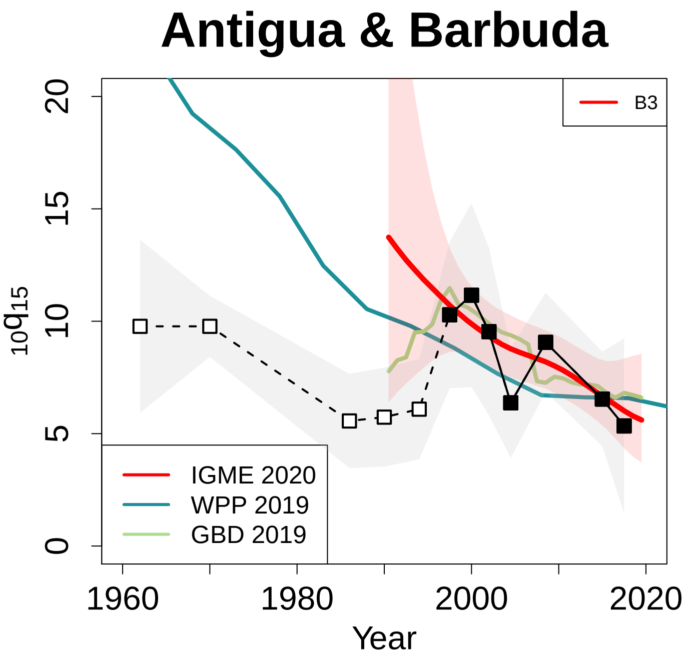
Antigua & Barbuda
B3
IGME 2020
WPP 2019
GBD 2019
$_{10}q_{15}$
0
5
10
15
20
1960
1980
2000
2020
Year

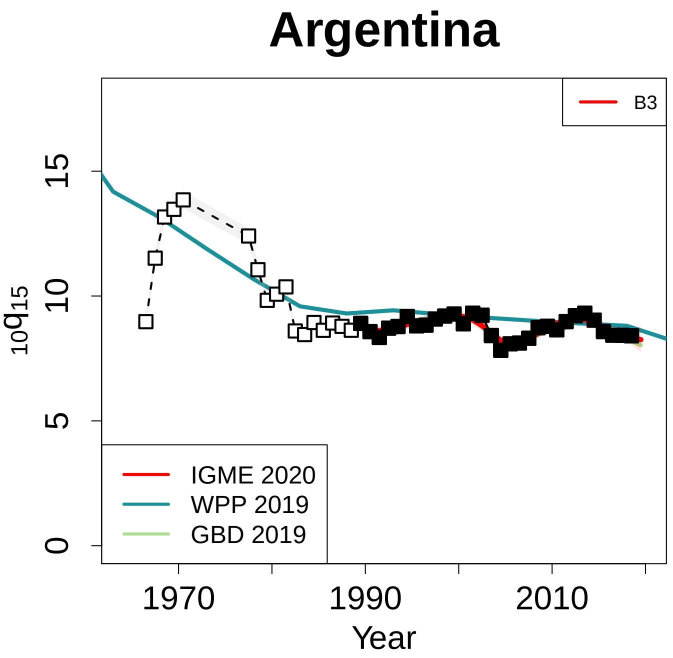
Argentina
B3
15
10
5
0
$_{10}q_{15}$
IGME 2020
WPP 2019
GBD 2019
1970
1990
2010
Year

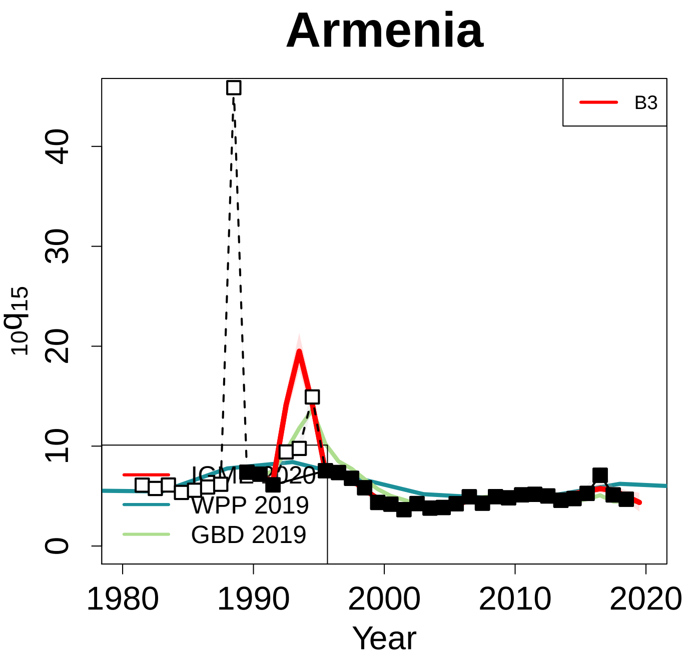
Armenia
B3
40
30
20
10
0
$_{10}q_{15}$
IGME 2020
WPP 2019
GBD 2019
1980
1990
2000
2010
2020
Year

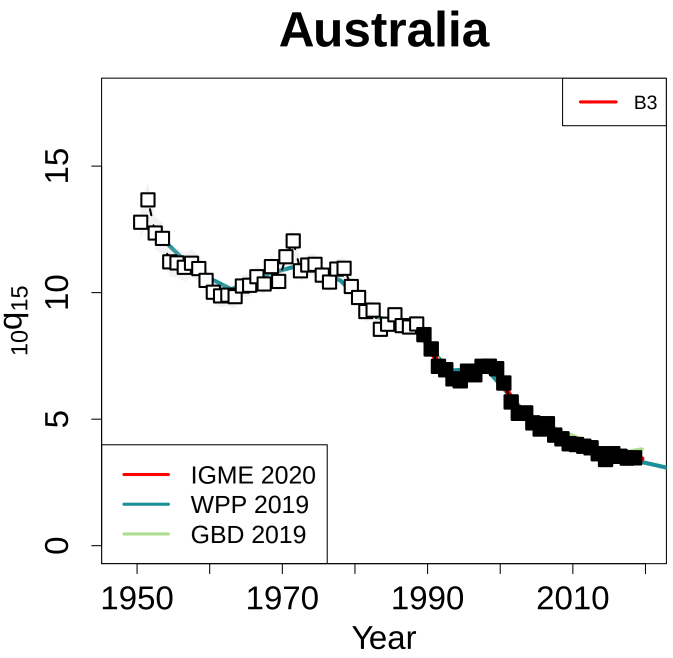
Australia
B3
IGME 2020
WPP 2019
GBD 2019
$_{10}q_{15}$
0
5
10
15
1950
1970
1990
2010
Year

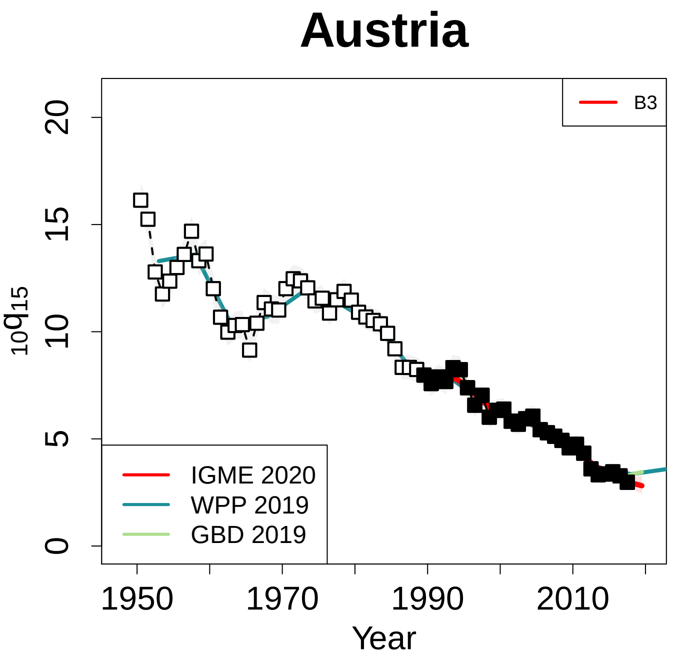
Austria
B3
IGME 2020
WPP 2019
GBD 2019
$_{10}q_{15}$
0
5
10
15
20
1950
1970
1990
2010
Year

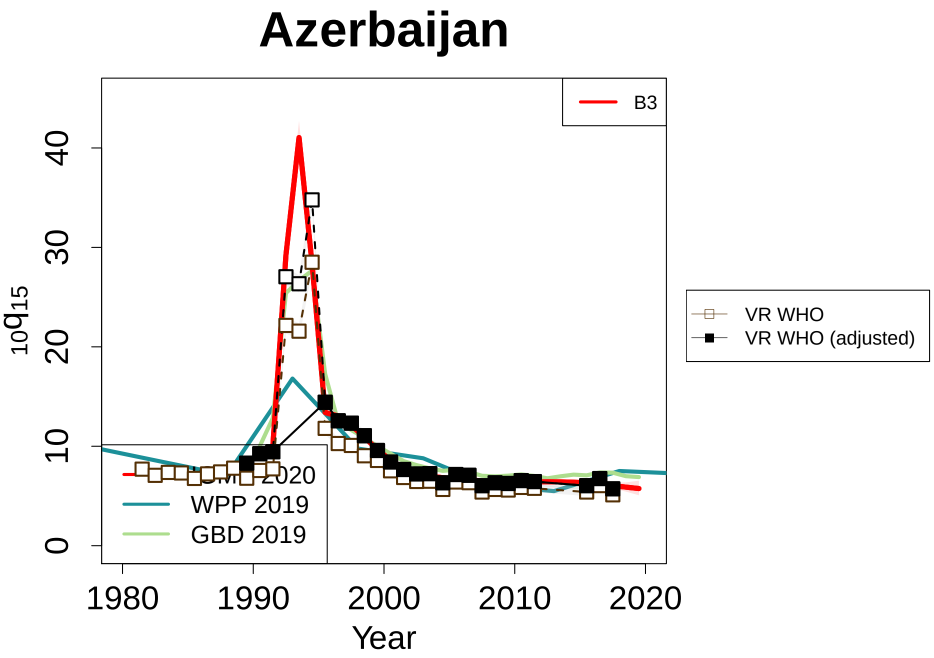
Azerbaijan
B3
VR WHO
VR WHO (adjusted)
WPP 2019
GBD 2019
$_{10}q_{15}$
0
10
20
30
40
1980
1990
2000
2010
2020
Year

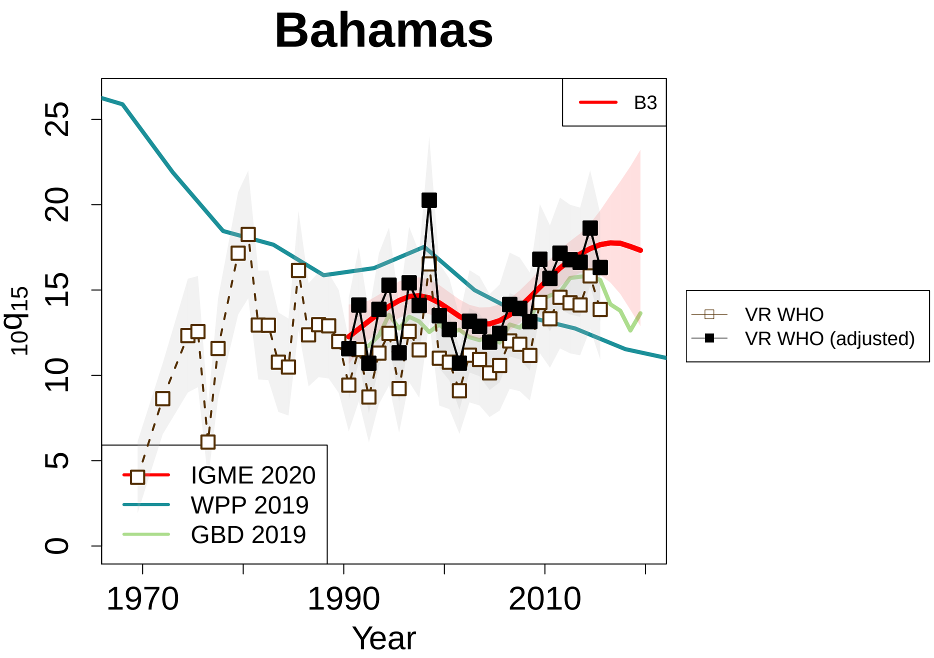
Bahamas
B3
VR WHO
VR WHO (adjusted)
IGME 2020
WPP 2019
GBD 2019
$_{10}q_{15}$
0
5
10
15
20
25
1970
1990
2010
Year

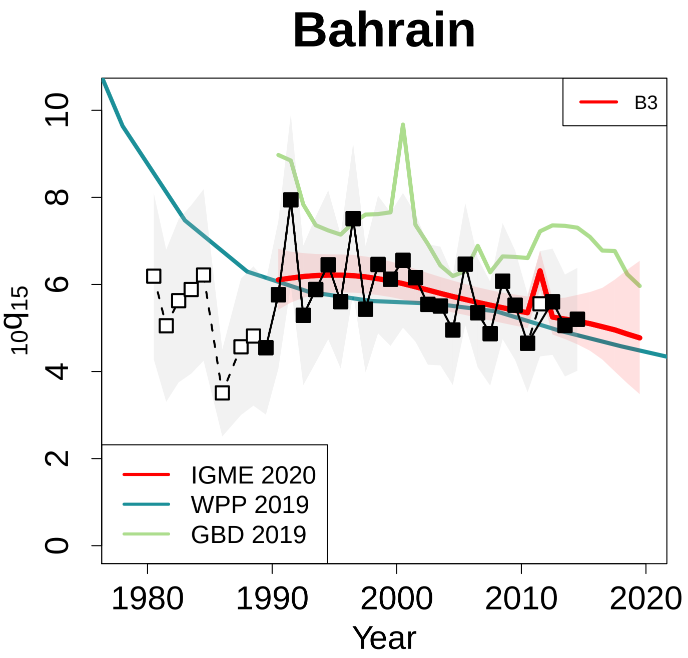
Bahrain
B3
IGME 2020
WPP 2019
GBD 2019
0
2
4
6
8
10
1980
1990
2000
2010
2020
Year
$_{10}q_{15}$

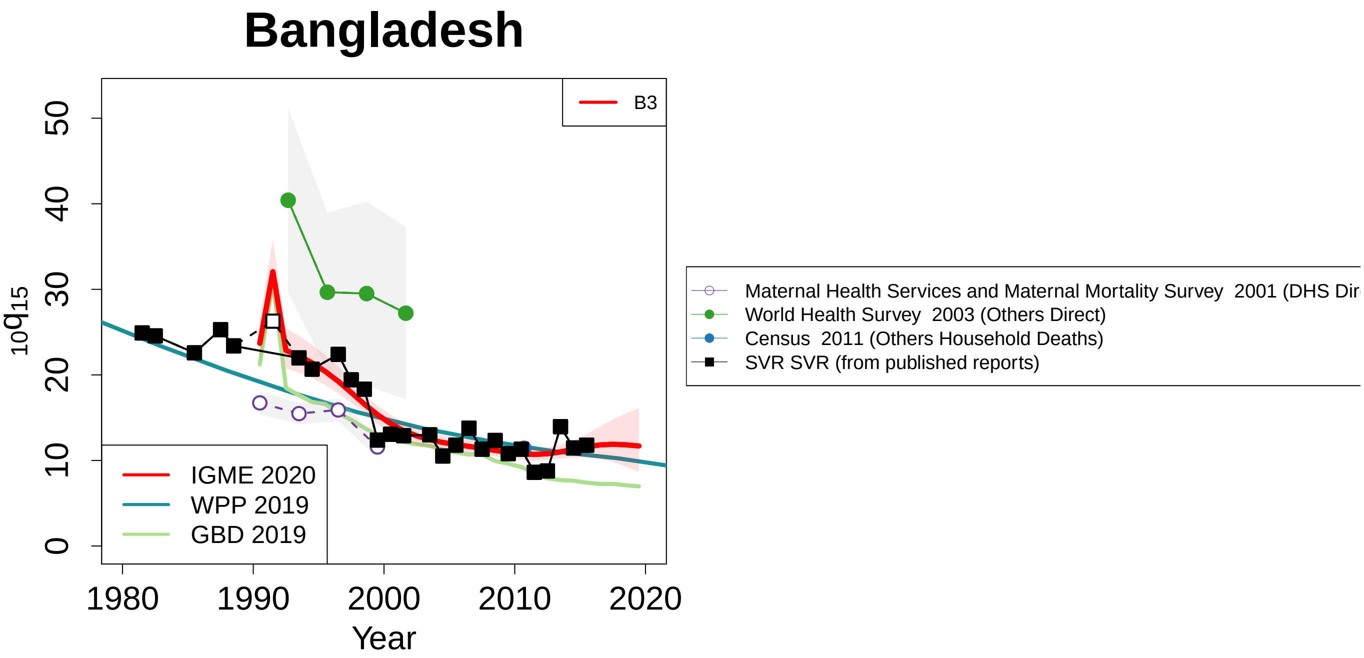
Bangladesh
B3
IGME 2020
WPP 2019
GBD 2019
0
10
20
30
40
50
1980
1990
2000
2010
2020
Year
$_{10}q_{15}$
Maternal Health Services and Maternal Mortality Survey 2001 (DHS Dir
World Health Survey 2003 (Others Direct)
Census 2011 (Others Household Deaths)
SVR SVR (from published reports)

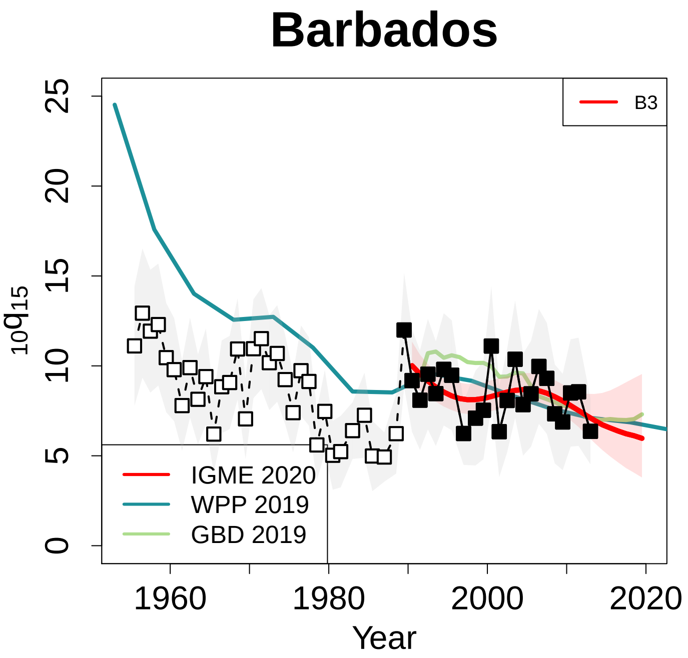
Barbados
B3
IGME 2020
WPP 2019
GBD 2019
$_{10}q_{15}$
0
5
10
15
20
25
1960
1980
2000
2020
Year

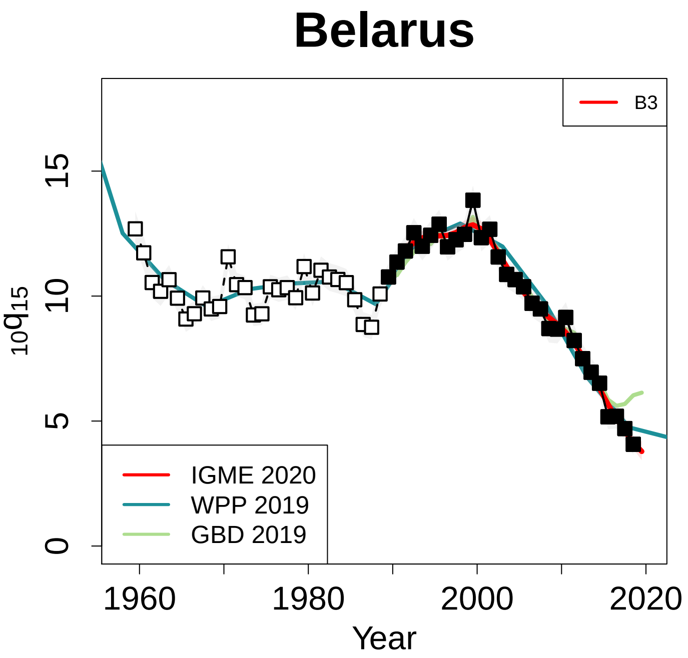
Belarus
B3
IGME 2020
WPP 2019
GBD 2019
$_{10}q_{15}$
0
5
10
15
1960
1980
2000
2020
Year

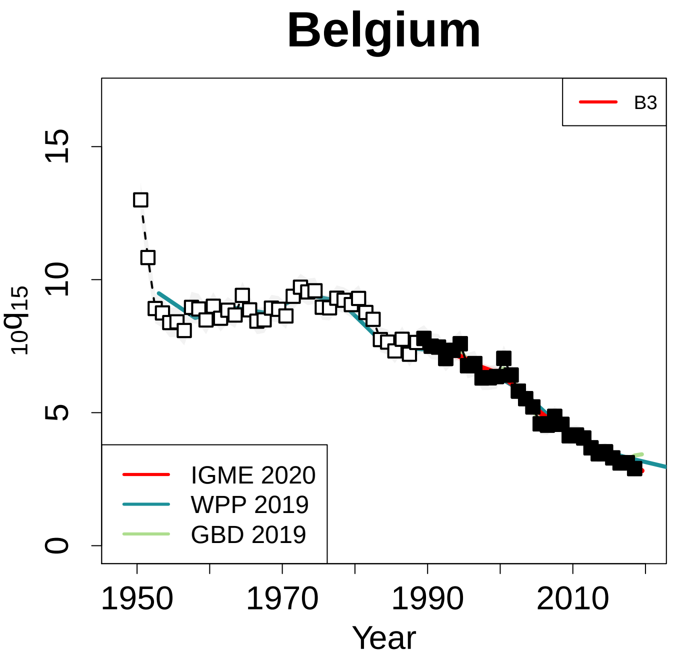
Belgium
B3
IGME 2020
WPP 2019
GBD 2019
$_{10}q_{15}$
0
5
10
15
1950
1970
1990
2010
Year

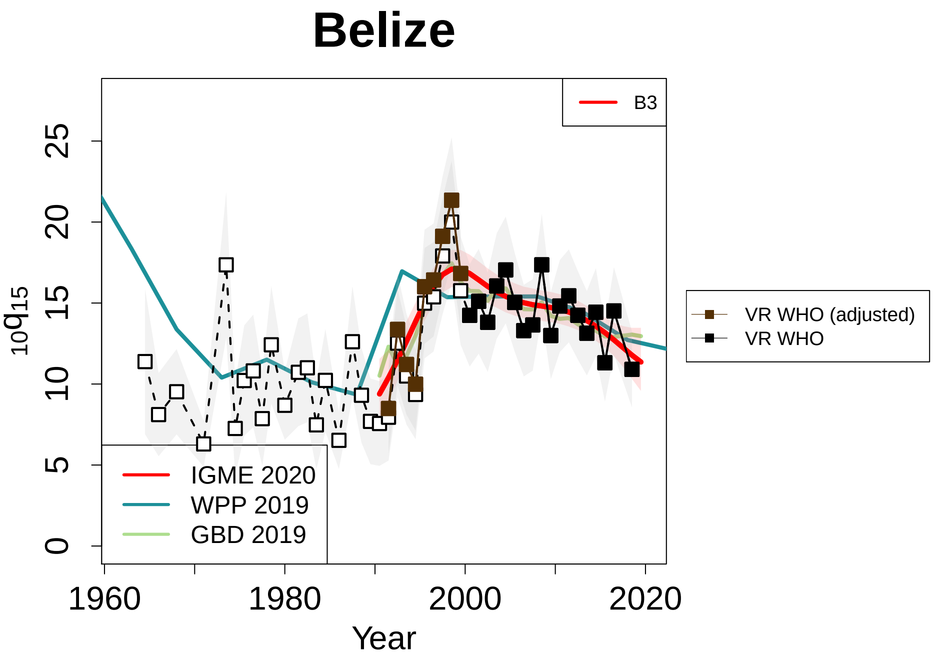
Belize
B3
IGME 2020
WPP 2019
GBD 2019
VR WHO (adjusted)
VR WHO
$_{10}q_{15}$
0
5
10
15
20
25
1960
1980
2000
2020
Year

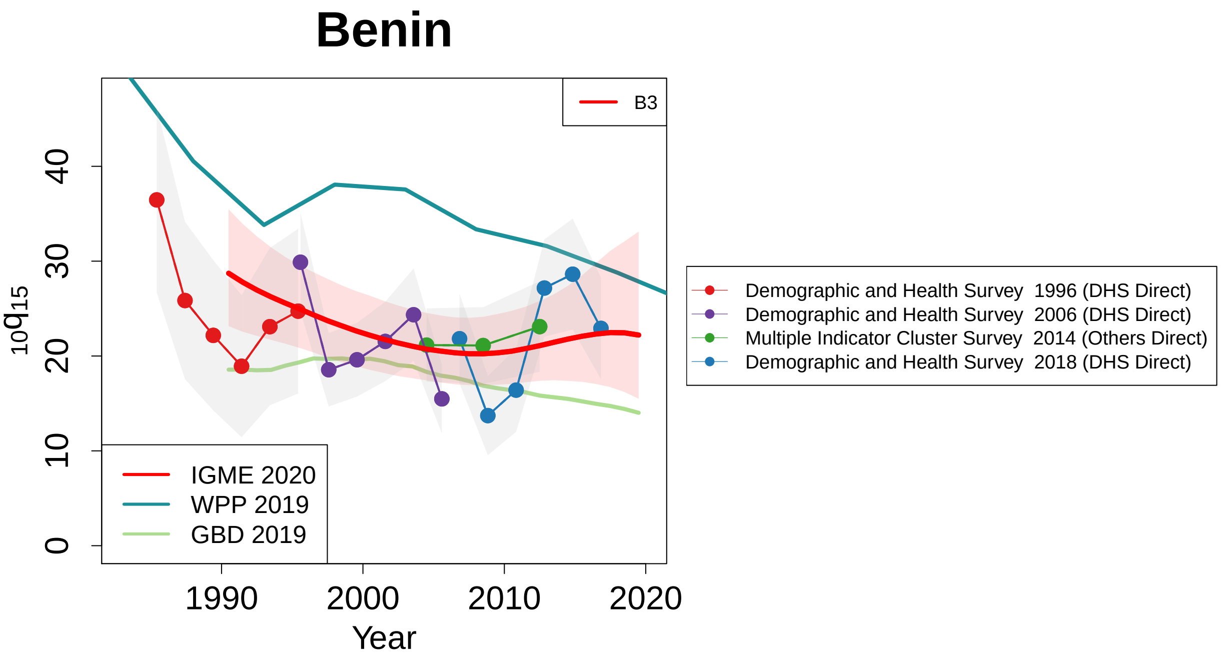
Benin
B3
40
30
20
10
0
$_{10}q_{15}$
1990
2000
2010
2020
Year
IGME 2020
WPP 2019
GBD 2019
Demographic and Health Survey 1996 (DHS Direct)
Demographic and Health Survey 2006 (DHS Direct)
Multiple Indicator Cluster Survey 2014 (Others Direct)
Demographic and Health Survey 2018 (DHS Direct)

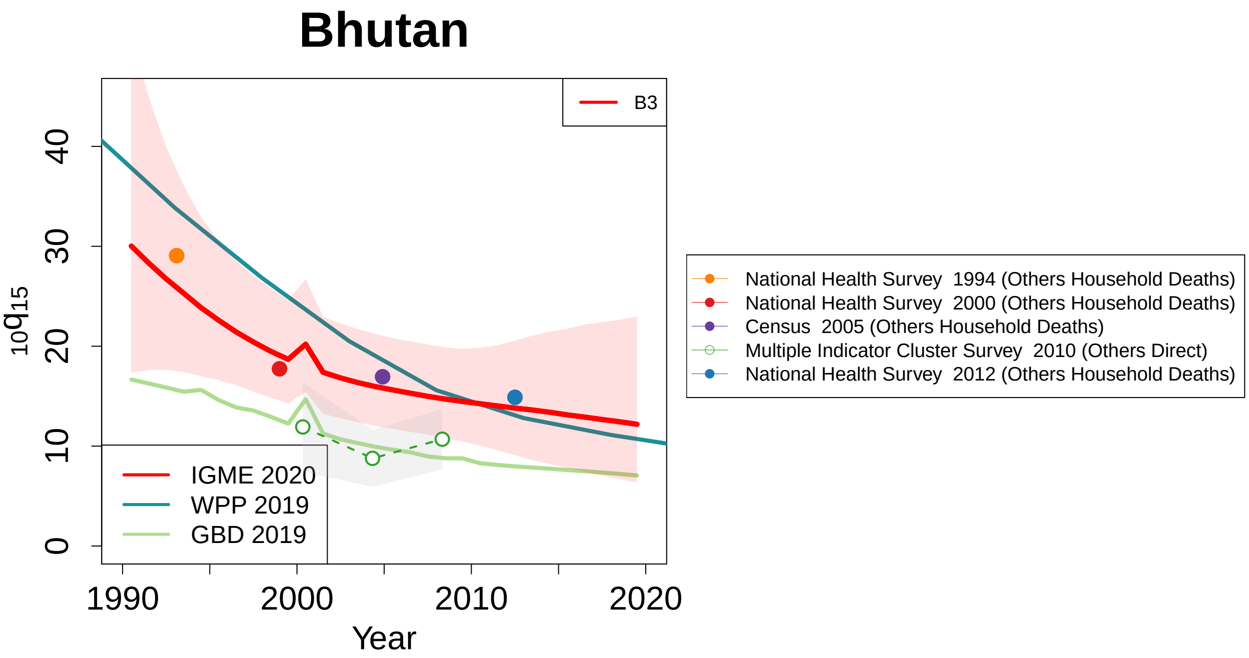
Bhutan
B3
40
30
20
10
0
$_{10}q_{15}$
1990
2000
2010
2020
Year
IGME 2020
WPP 2019
GBD 2019
National Health Survey 1994 (Others Household Deaths)
National Health Survey 2000 (Others Household Deaths)
Census 2005 (Others Household Deaths)
Multiple Indicator Cluster Survey 2010 (Others Direct)
National Health Survey 2012 (Others Household Deaths)

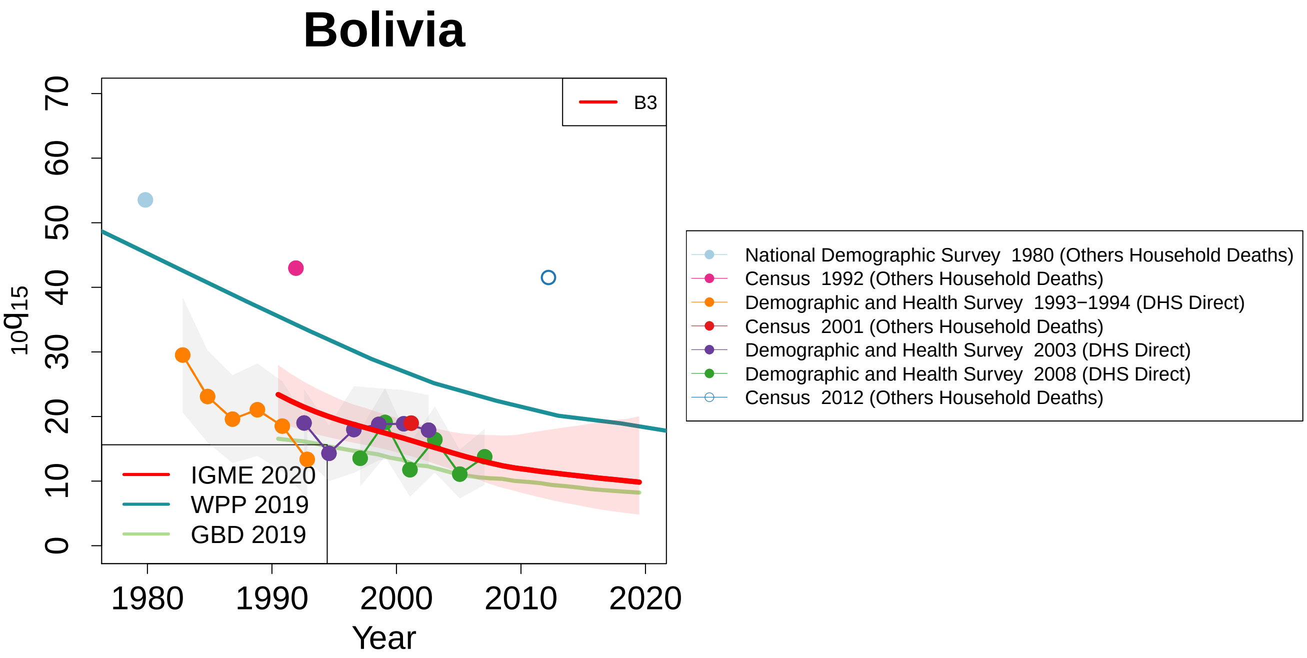
Bolivia
B3
National Demographic Survey 1980 (Others Household Deaths)
Census 1992 (Others Household Deaths)
Demographic and Health Survey 1993−1994 (DHS Direct)
Census 2001 (Others Household Deaths)
Demographic and Health Survey 2003 (DHS Direct)
Demographic and Health Survey 2008 (DHS Direct)
Census 2012 (Others Household Deaths)
IGME 2020
WPP 2019
GBD 2019
$_{10}q_{15}$
0 10 20 30 40 50 60 70
1980 1990 2000 2010 2020
Year

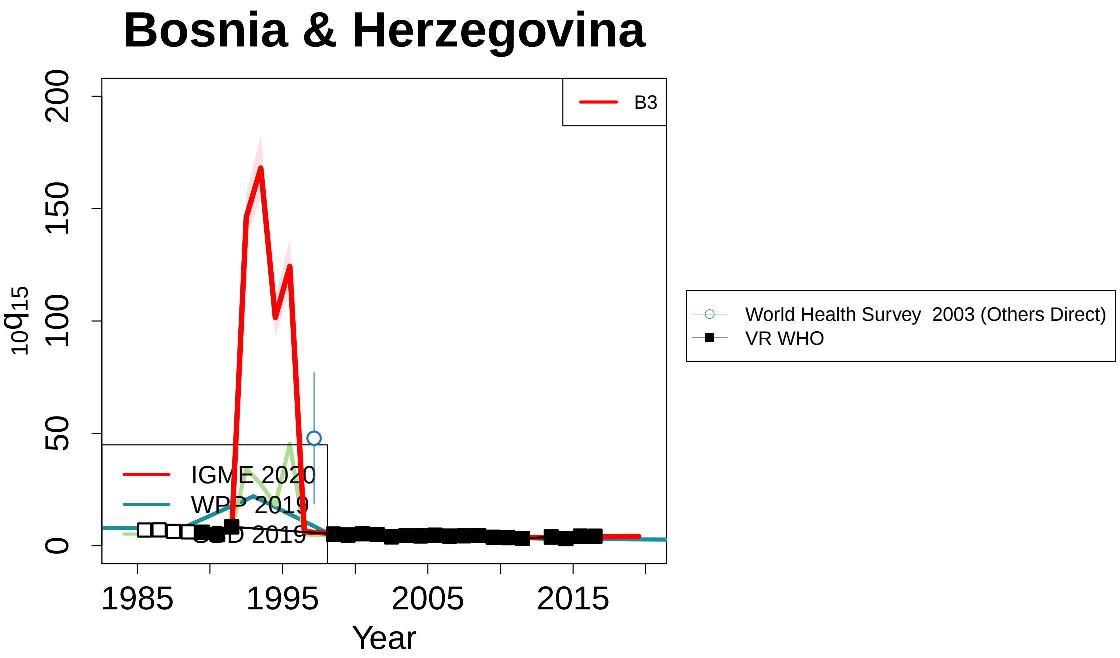
Bosnia & Herzegovina
B3
World Health Survey 2003 (Others Direct)
VR WHO
IGME 2020
WPP 2019
GBD 2019
$_{10}q_{15}$
0 50 100 150 200
1985 1995 2005 2015
Year

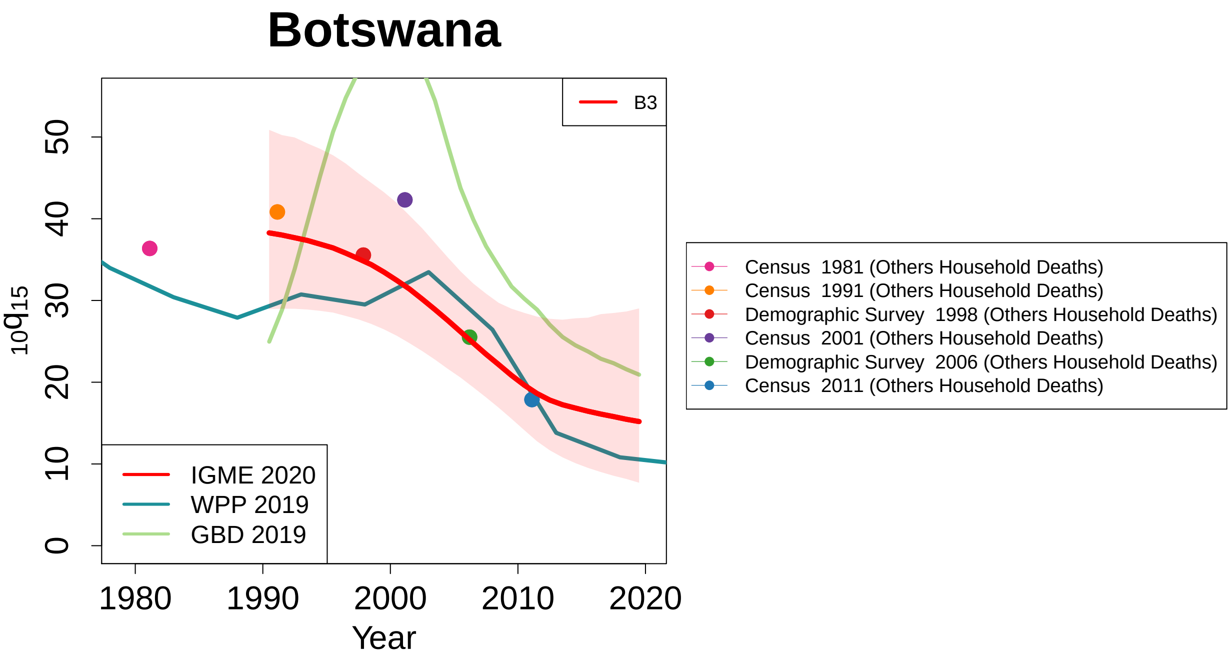
Botswana
B3
Census 1981 (Others Household Deaths)
Census 1991 (Others Household Deaths)
Demographic Survey 1998 (Others Household Deaths)
Census 2001 (Others Household Deaths)
Demographic Survey 2006 (Others Household Deaths)
Census 2011 (Others Household Deaths)
IGME 2020
WPP 2019
GBD 2019
Year

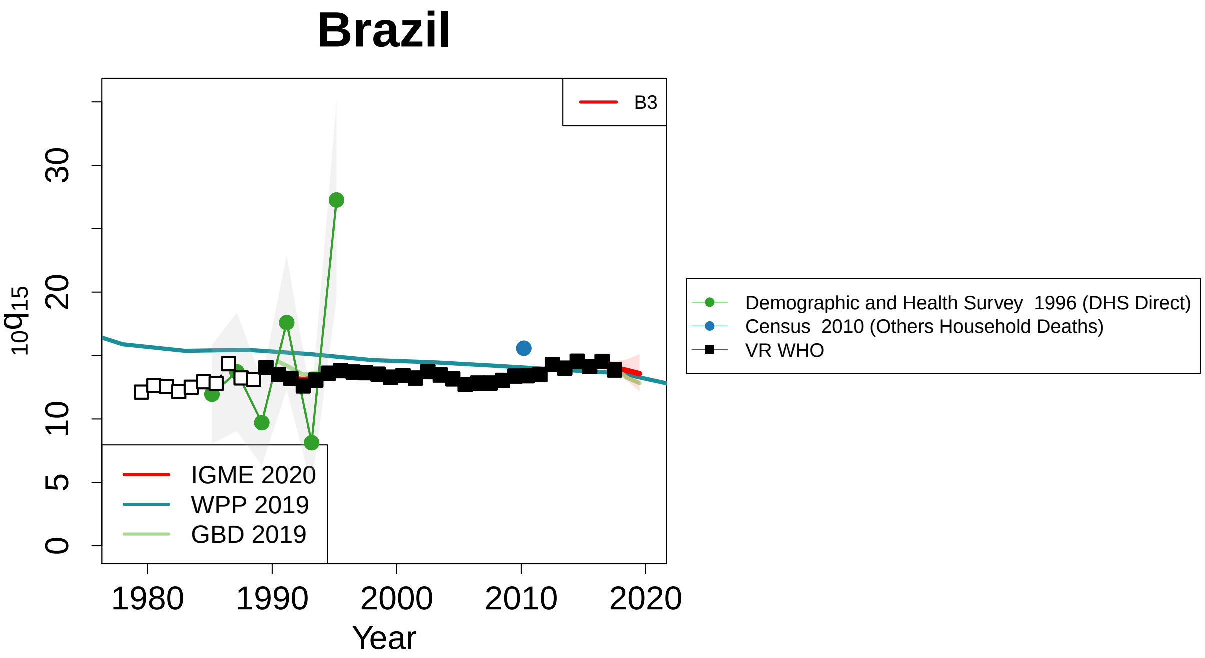
Brazil
B3
Demographic and Health Survey 1996 (DHS Direct)
Census 2010 (Others Household Deaths)
VR WHO
IGME 2020
WPP 2019
GBD 2019
Year

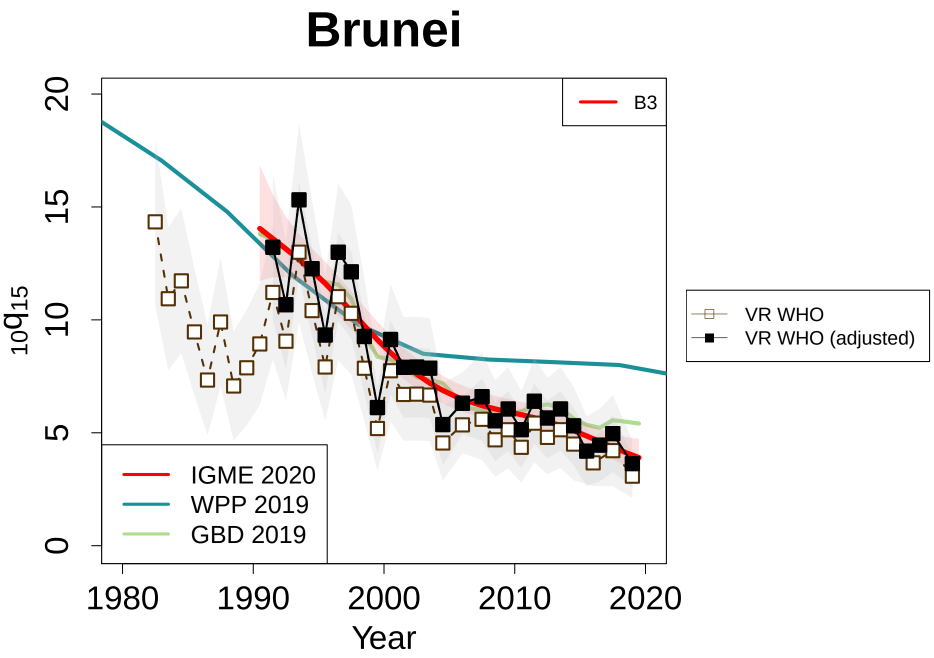
Brunei
B3
VR WHO
VR WHO (adjusted)
IGME 2020
WPP 2019
GBD 2019
$_{10}q_{15}$
0
5
10
15
20
1980
1990
2000
2010
2020
Year

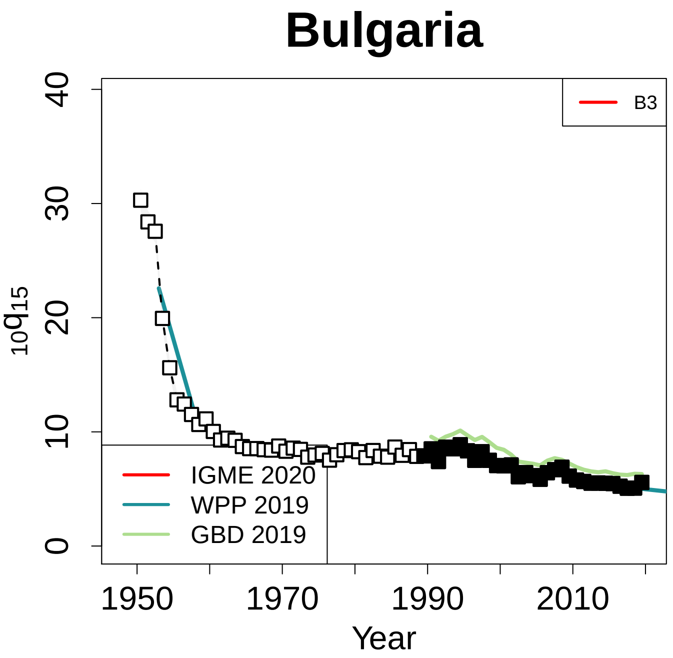
Bulgaria
B3
IGME 2020
WPP 2019
GBD 2019
$_{10}q_{15}$
0
10
20
30
40
1950
1970
1990
2010
Year

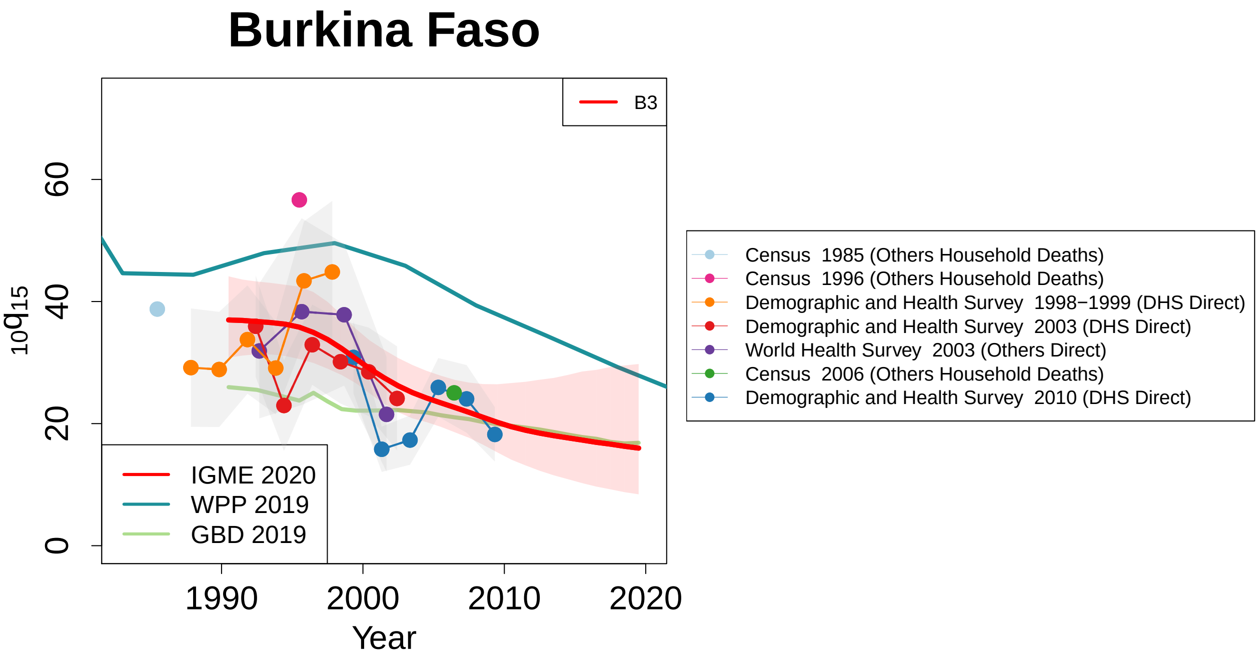
Burkina Faso
B3
IGME 2020
WPP 2019
GBD 2019
0
20
40
60
$_{10}q_{15}$
1990
2000
2010
2020
Year
Census 1985 (Others Household Deaths)
Census 1996 (Others Household Deaths)
Demographic and Health Survey 1998−1999 (DHS Direct)
Demographic and Health Survey 2003 (DHS Direct)
World Health Survey 2003 (Others Direct)
Census 2006 (Others Household Deaths)
Demographic and Health Survey 2010 (DHS Direct)

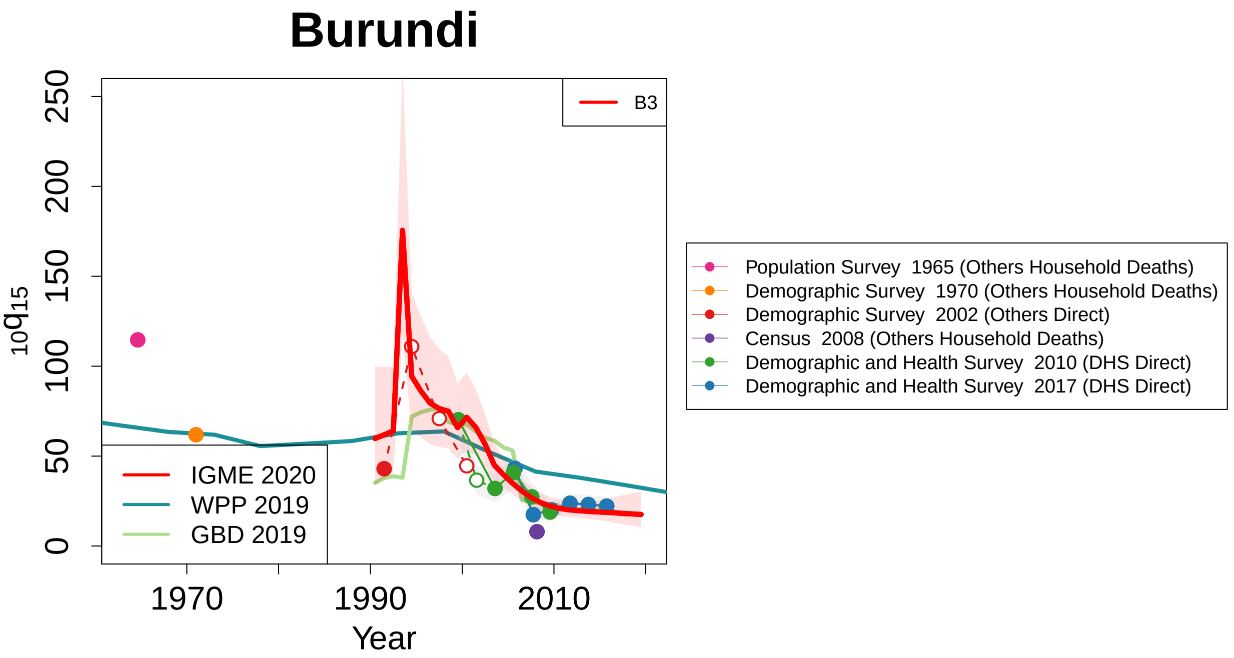
Burundi
B3
IGME 2020
WPP 2019
GBD 2019
0
50
100
150
200
250
$_{10}q_{15}$
1970
1990
2010
Year
Population Survey 1965 (Others Household Deaths)
Demographic Survey 1970 (Others Household Deaths)
Demographic Survey 2002 (Others Direct)
Census 2008 (Others Household Deaths)
Demographic and Health Survey 2010 (DHS Direct)
Demographic and Health Survey 2017 (DHS Direct)

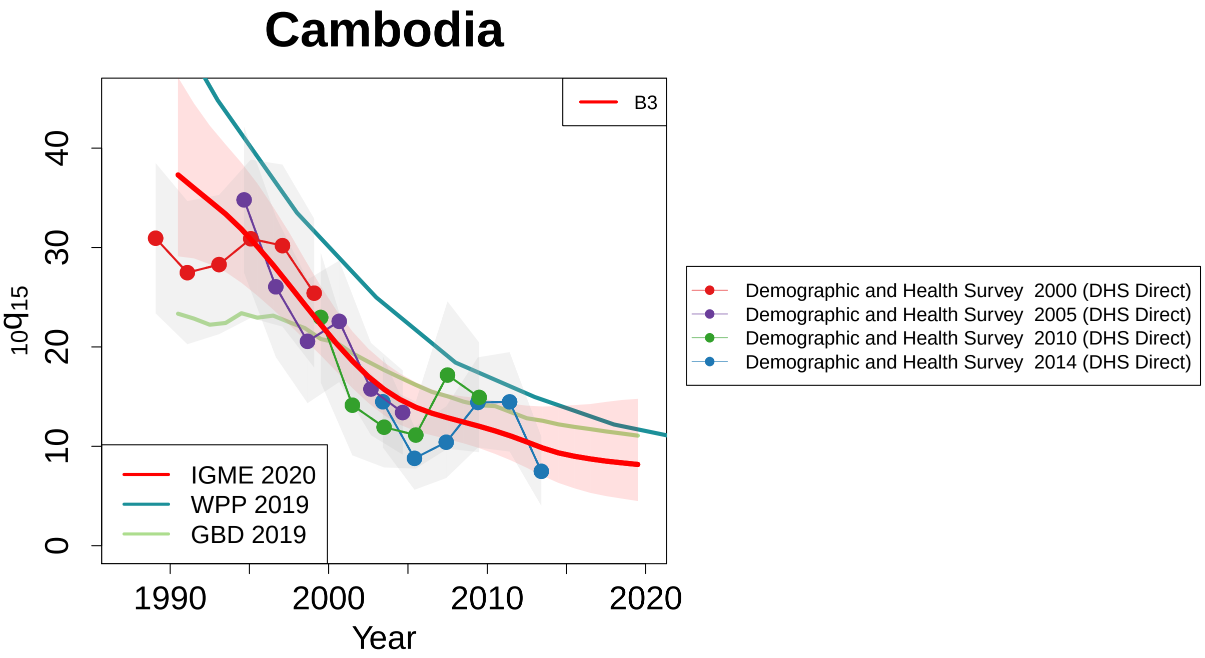
Cambodia
B3
40
30
20
10
0
$_{10}q_{15}$
IGME 2020
WPP 2019
GBD 2019
1990
2000
2010
2020
Year
Demographic and Health Survey 2000 (DHS Direct)
Demographic and Health Survey 2005 (DHS Direct)
Demographic and Health Survey 2010 (DHS Direct)
Demographic and Health Survey 2014 (DHS Direct)

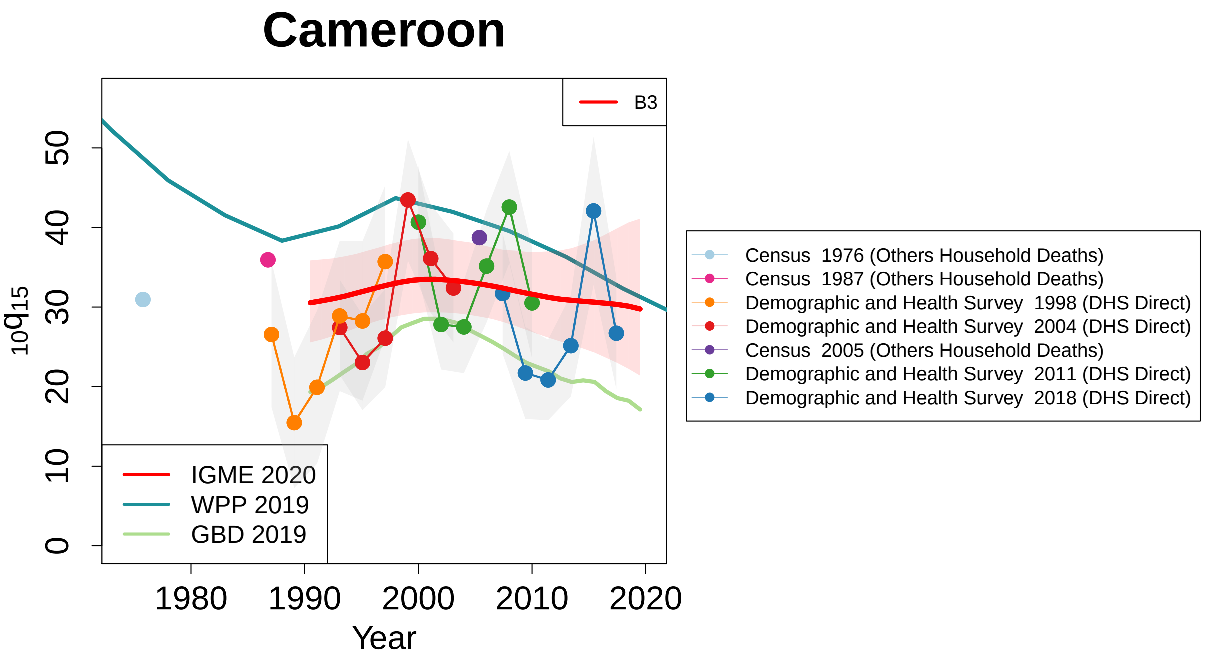
Cameroon
B3
50
40
30
20
10
0
$_{10}q_{15}$
IGME 2020
WPP 2019
GBD 2019
1980
1990
2000
2010
2020
Year
Census 1976 (Others Household Deaths)
Census 1987 (Others Household Deaths)
Demographic and Health Survey 1998 (DHS Direct)
Demographic and Health Survey 2004 (DHS Direct)
Census 2005 (Others Household Deaths)
Demographic and Health Survey 2011 (DHS Direct)
Demographic and Health Survey 2018 (DHS Direct)

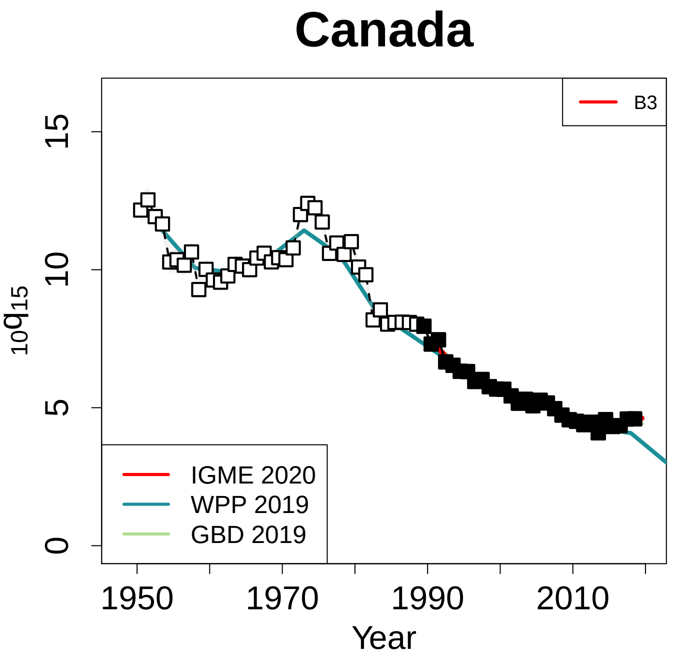
Canada
B3
15
10
5
0
$_{10}q_{15}$
IGME 2020
WPP 2019
GBD 2019
1950
1970
1990
2010
Year

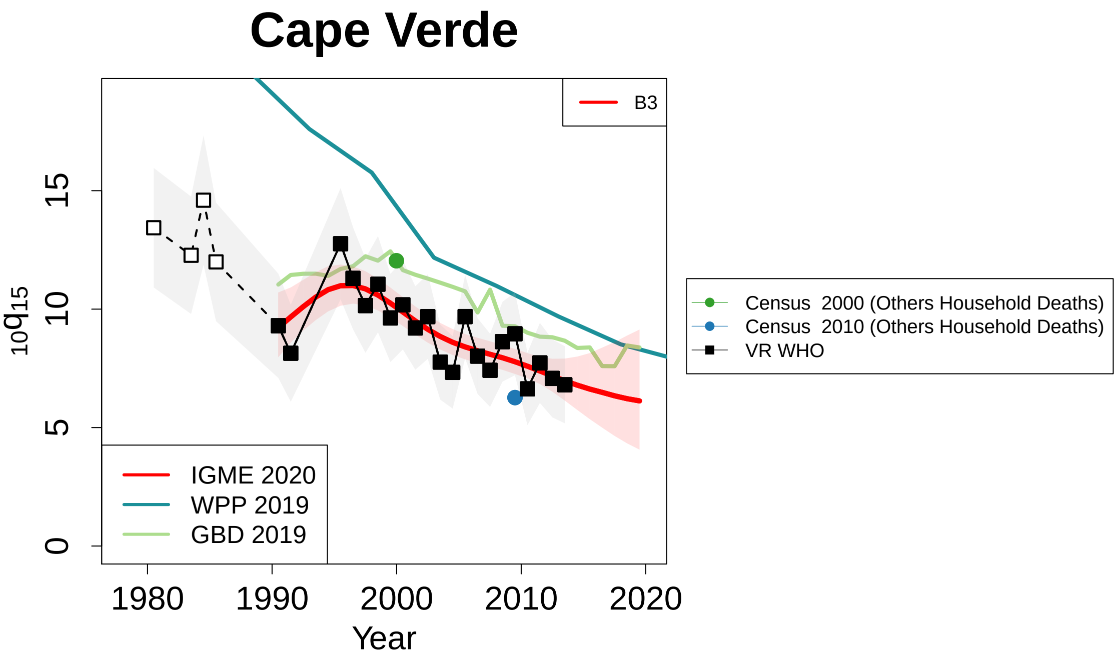
Cape Verde
B3
15
10
5
0
$_{10}q_{15}$
IGME 2020
WPP 2019
GBD 2019
1980
1990
2000
2010
2020
Year
Census 2000 (Others Household Deaths)
Census 2010 (Others Household Deaths)
VR WHO

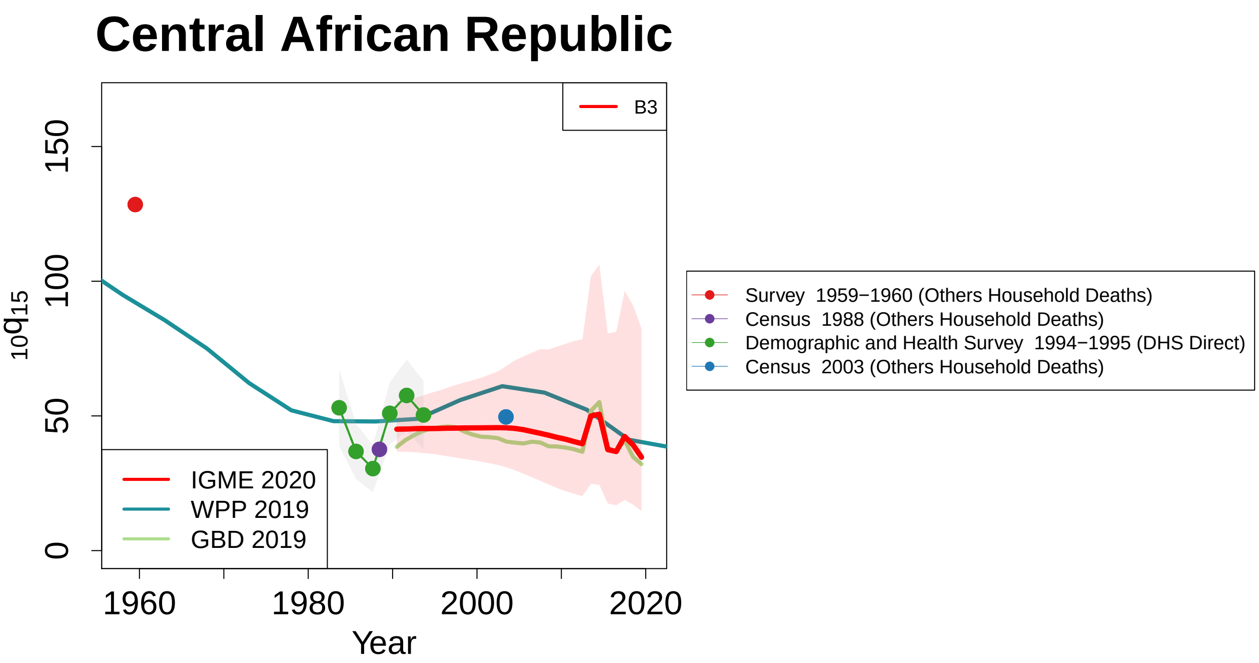
Central African Republic
B3
IGME 2020
WPP 2019
GBD 2019
$_{10}q_{15}$
Year
Survey 1959−1960 (Others Household Deaths)
Census 1988 (Others Household Deaths)
Demographic and Health Survey 1994−1995 (DHS Direct)
Census 2003 (Others Household Deaths)

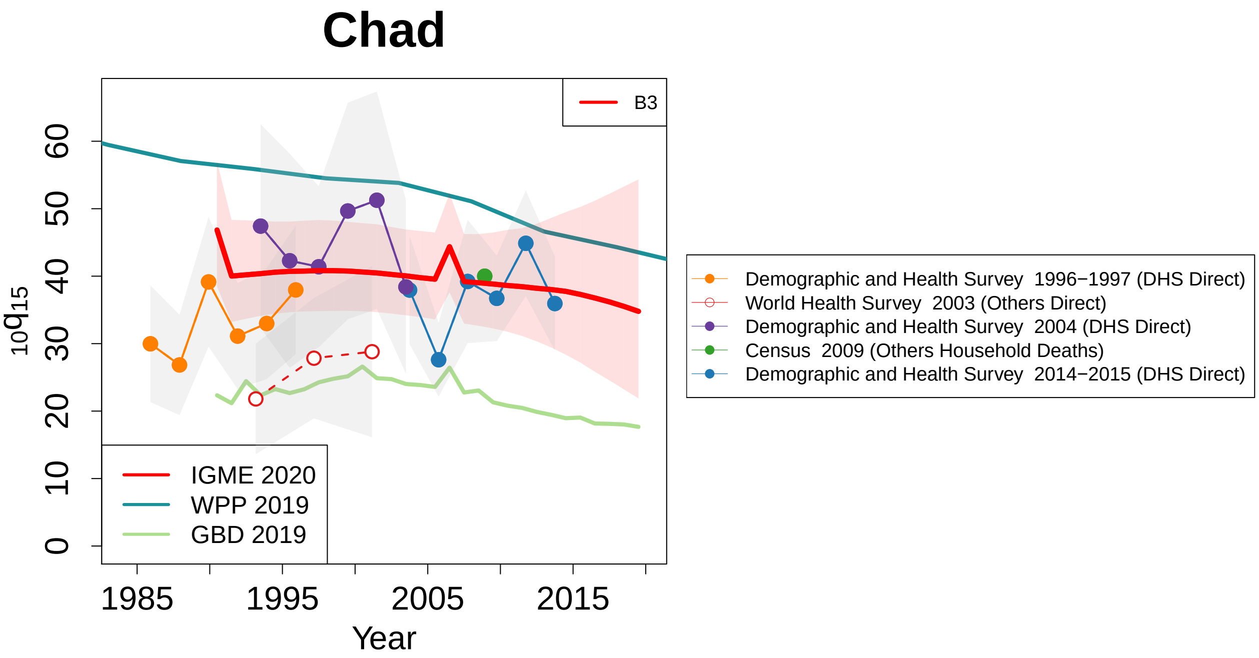
Chad
B3
IGME 2020
WPP 2019
GBD 2019
$_{10}q_{15}$
Year
Demographic and Health Survey 1996−1997 (DHS Direct)
World Health Survey 2003 (Others Direct)
Demographic and Health Survey 2004 (DHS Direct)
Census 2009 (Others Household Deaths)
Demographic and Health Survey 2014−2015 (DHS Direct)

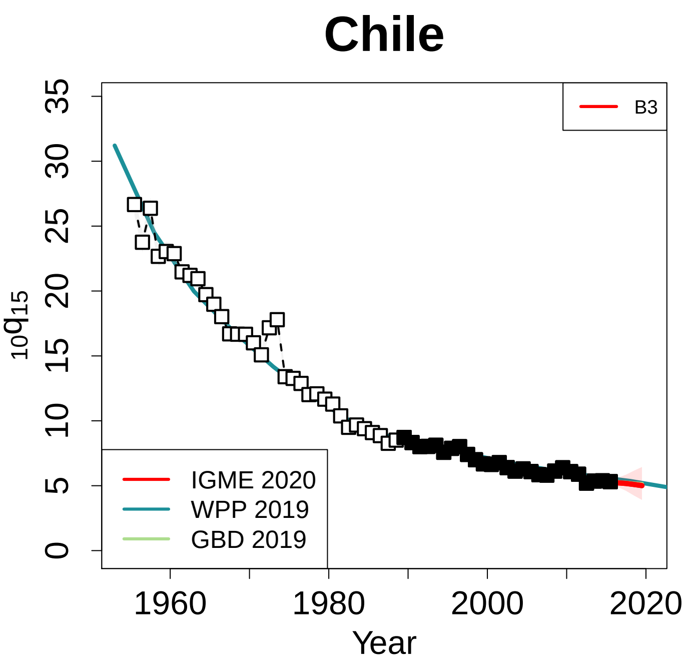
Chile
B3
IGME 2020
WPP 2019
GBD 2019
$_{10}q_{15}$
0
5
10
15
20
25
30
35
1960
1980
2000
2020
Year

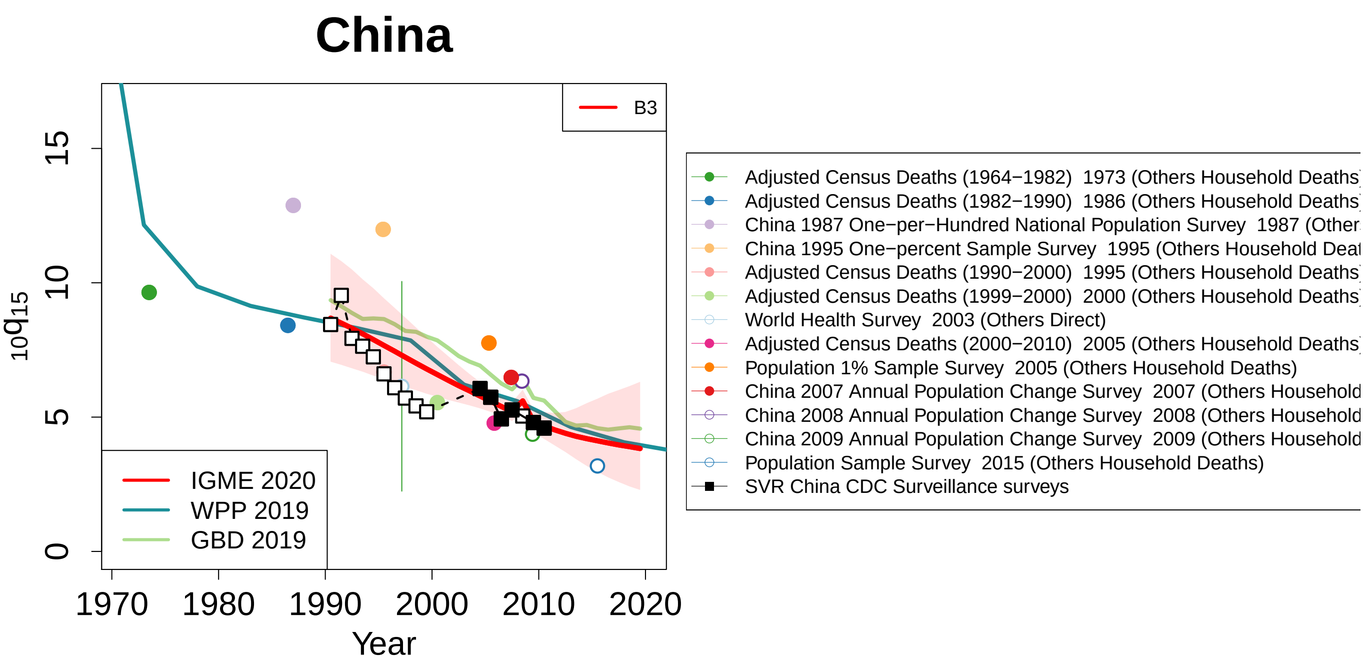
China
B3
IGME 2020
WPP 2019
GBD 2019
$_{10}q_{15}$
0
5
10
15
1970
1980
1990
2000
2010
2020
Year
Adjusted Census Deaths (1964−1982) 1973 (Others Household Deaths
Adjusted Census Deaths (1982−1990) 1986 (Others Household Deaths
China 1987 One−per−Hundred National Population Survey 1987 (Other
China 1995 One−percent Sample Survey 1995 (Others Household Deat
Adjusted Census Deaths (1990−2000) 1995 (Others Household Deaths
Adjusted Census Deaths (1999−2000) 2000 (Others Household Deaths
World Health Survey 2003 (Others Direct)
Adjusted Census Deaths (2000−2010) 2005 (Others Household Deaths
Population 1% Sample Survey 2005 (Others Household Deaths)
China 2007 Annual Population Change Survey 2007 (Others Household
China 2008 Annual Population Change Survey 2008 (Others Household
China 2009 Annual Population Change Survey 2009 (Others Household
Population Sample Survey 2015 (Others Household Deaths)
SVR China CDC Surveillance surveys

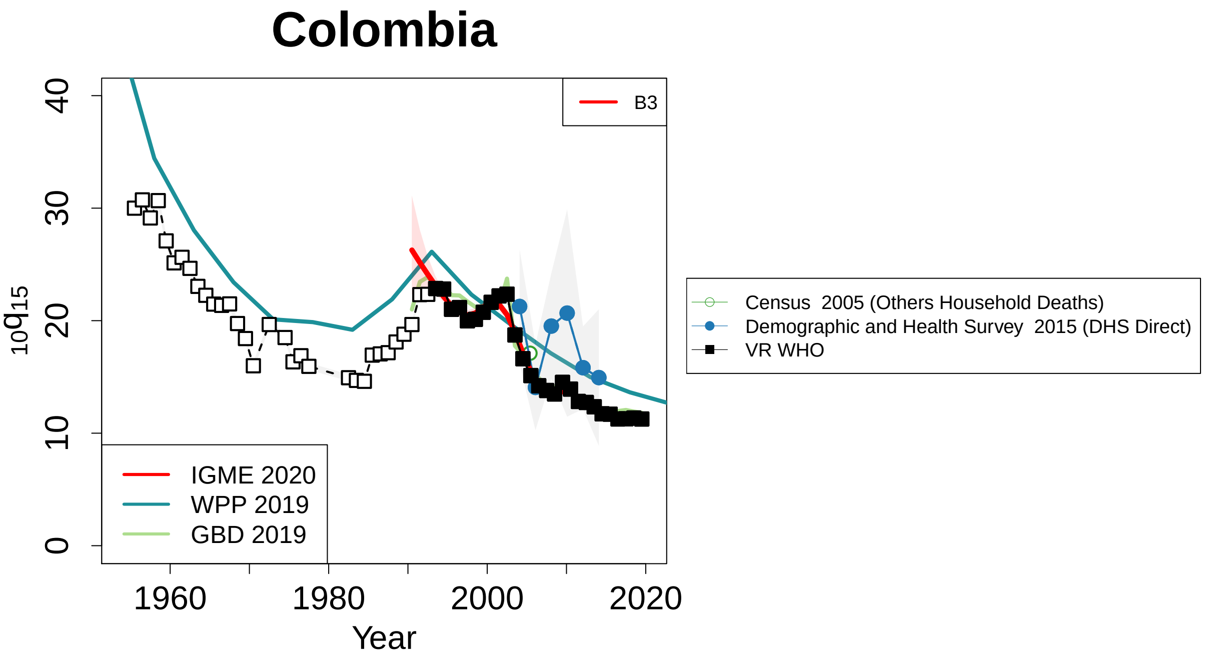
Colombia
B3
IGME 2020
WPP 2019
GBD 2019
Census 2005 (Others Household Deaths)
Demographic and Health Survey 2015 (DHS Direct)
VR WHO
$_{10}q_{15}$
0
10
20
30
40
1960
1980
2000
2020
Year

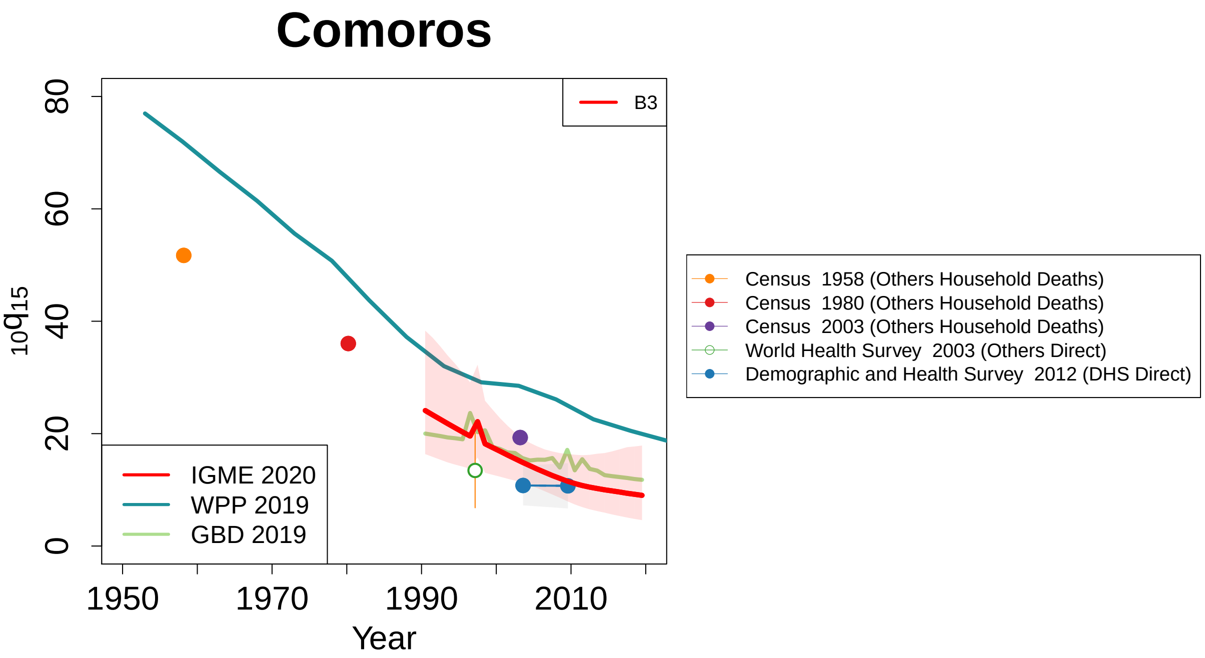
Comoros
B3
IGME 2020
WPP 2019
GBD 2019
Census 1958 (Others Household Deaths)
Census 1980 (Others Household Deaths)
Census 2003 (Others Household Deaths)
World Health Survey 2003 (Others Direct)
Demographic and Health Survey 2012 (DHS Direct)
$_{10}q_{15}$
0
20
40
60
80
1950
1970
1990
2010
Year

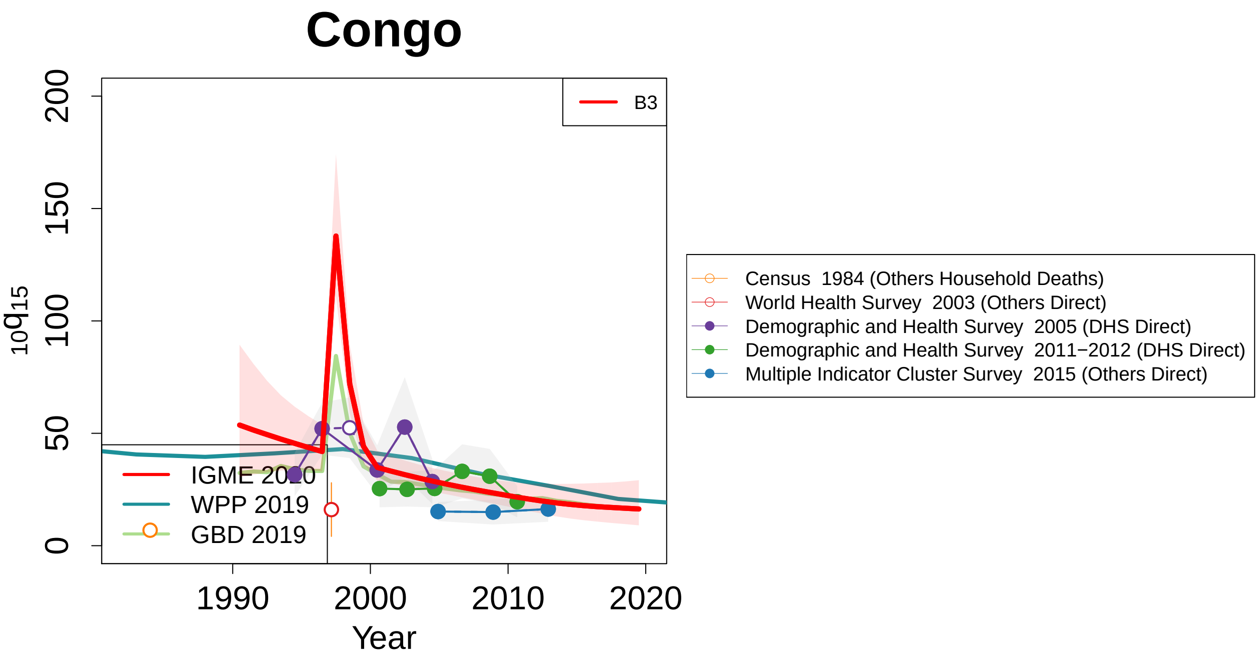
Congo
B3
$_{10}q_{15}$
0
50
100
150
200
IGME 2020
WPP 2019
GBD 2019
1990
2000
2010
2020
Year
Census 1984 (Others Household Deaths)
World Health Survey 2003 (Others Direct)
Demographic and Health Survey 2005 (DHS Direct)
Demographic and Health Survey 2011−2012 (DHS Direct)
Multiple Indicator Cluster Survey 2015 (Others Direct)

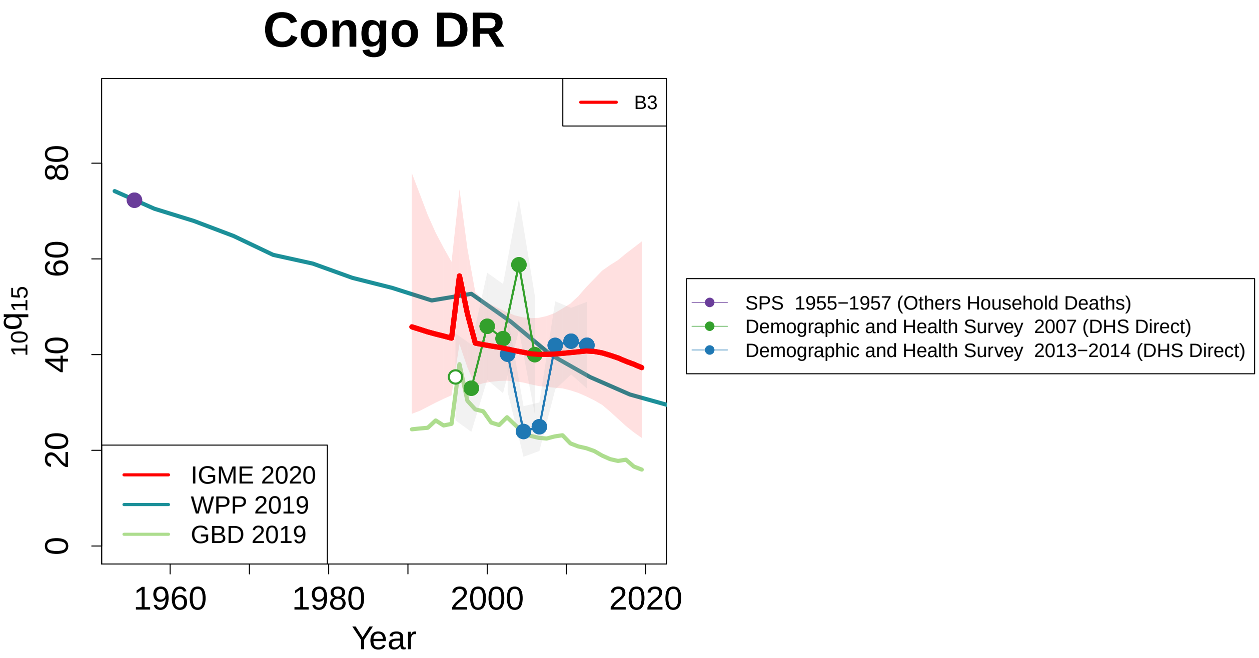
Congo DR
B3
$_{10}q_{15}$
0
20
40
60
80
IGME 2020
WPP 2019
GBD 2019
1960
1980
2000
2020
Year
SPS 1955−1957 (Others Household Deaths)
Demographic and Health Survey 2007 (DHS Direct)
Demographic and Health Survey 2013−2014 (DHS Direct)

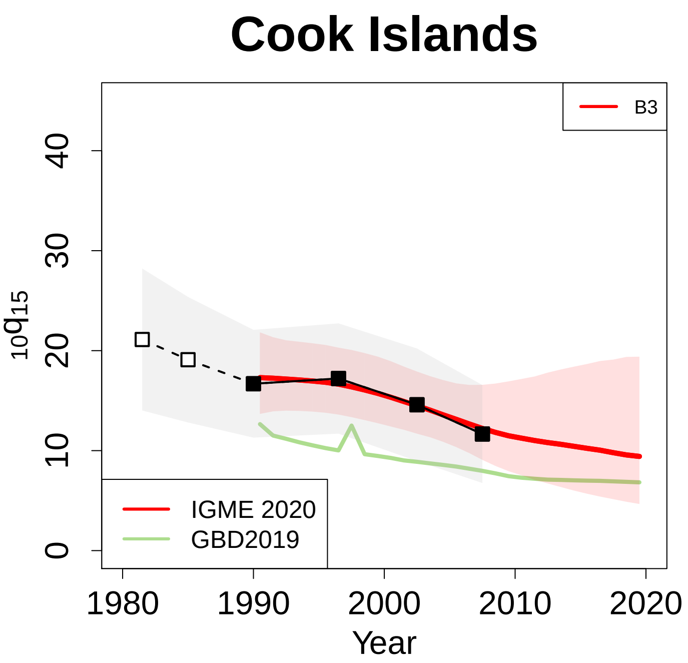
Cook Islands
B3
IGME 2020
GBD2019
$_{10}q_{15}$
0
10
20
30
40
1980
1990
2000
2010
2020
Year

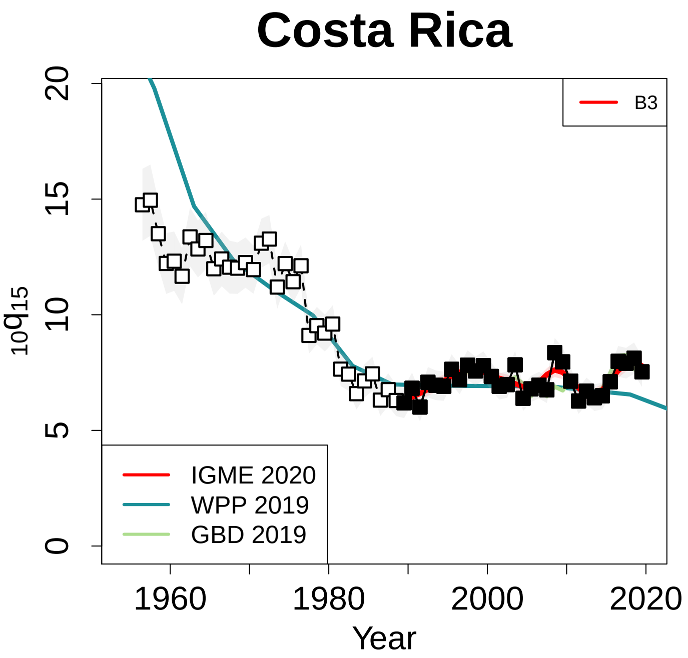
Costa Rica
B3
IGME 2020
WPP 2019
GBD 2019
$_{10}q_{15}$
0
5
10
15
20
1960
1980
2000
2020
Year

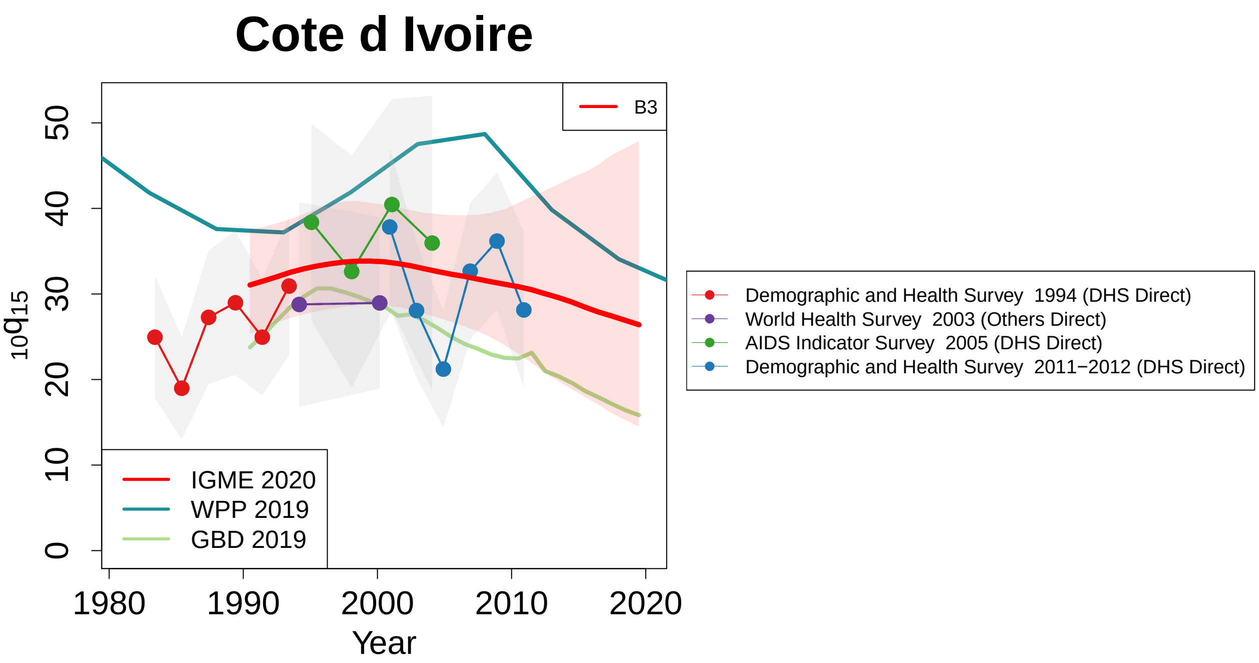
Cote d Ivoire
B3
IGME 2020
WPP 2019
GBD 2019
Demographic and Health Survey 1994 (DHS Direct)
World Health Survey 2003 (Others Direct)
AIDS Indicator Survey 2005 (DHS Direct)
Demographic and Health Survey 2011−2012 (DHS Direct)
$_{10}q_{15}$
Year

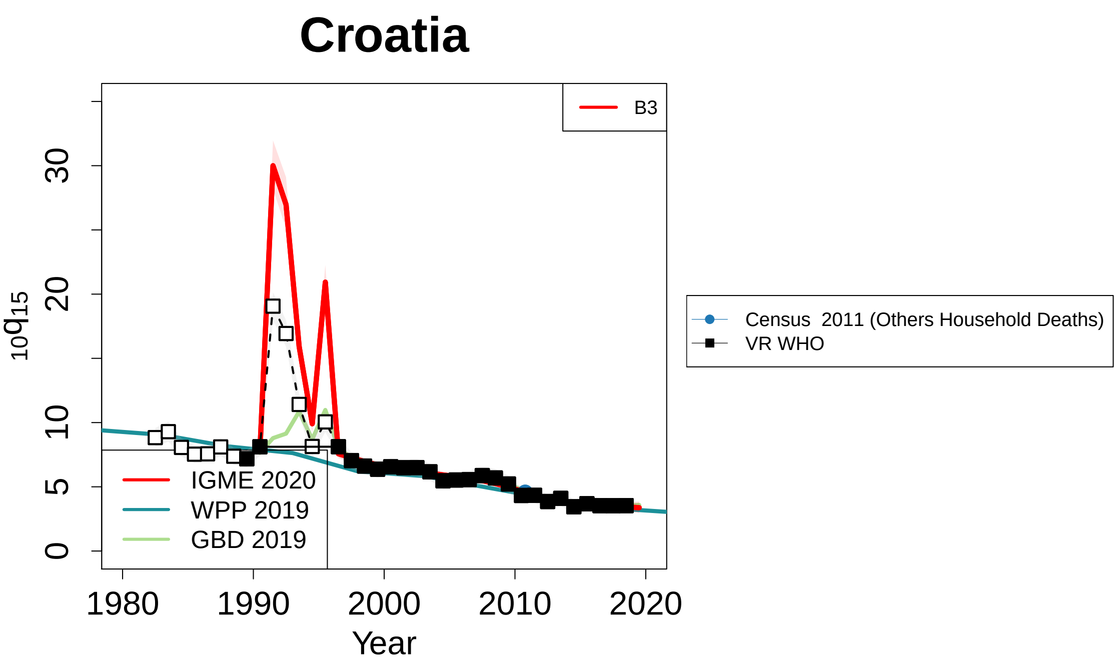
Croatia
B3
IGME 2020
WPP 2019
GBD 2019
Census 2011 (Others Household Deaths)
VR WHO
$_{10}q_{15}$
Year

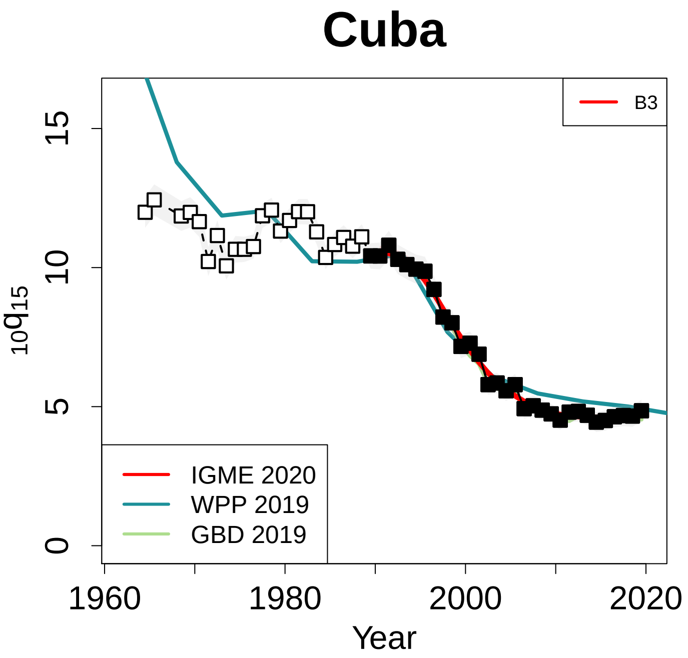
Cuba
B3
IGME 2020
WPP 2019
GBD 2019
$_{10}q_{15}$
0
5
10
15
1960
1980
2000
2020
Year

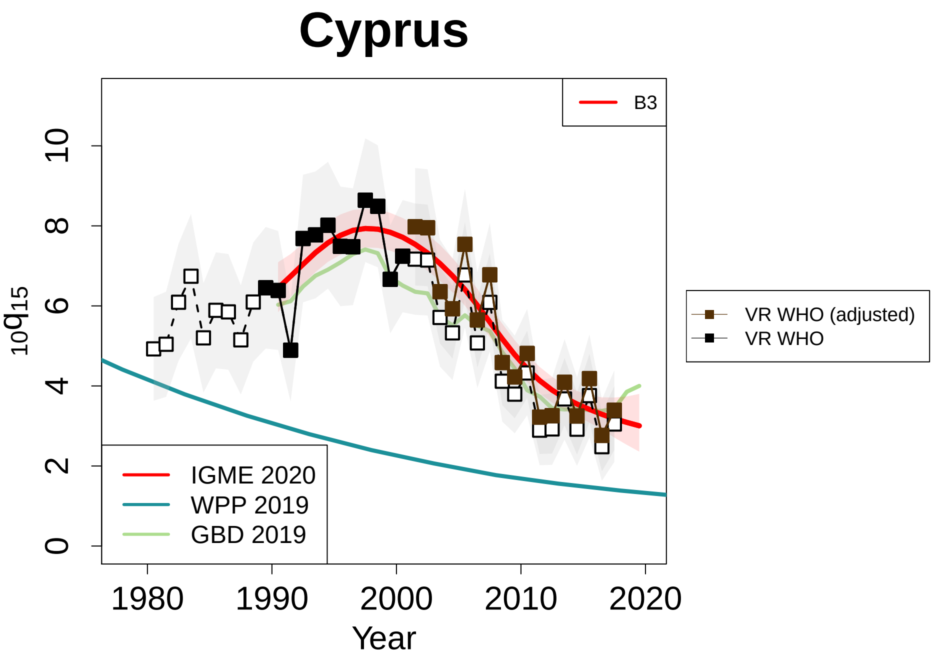
Cyprus
B3
IGME 2020
WPP 2019
GBD 2019
VR WHO (adjusted)
VR WHO
$_{10}q_{15}$
0
2
4
6
8
10
1980
1990
2000
2010
2020
Year

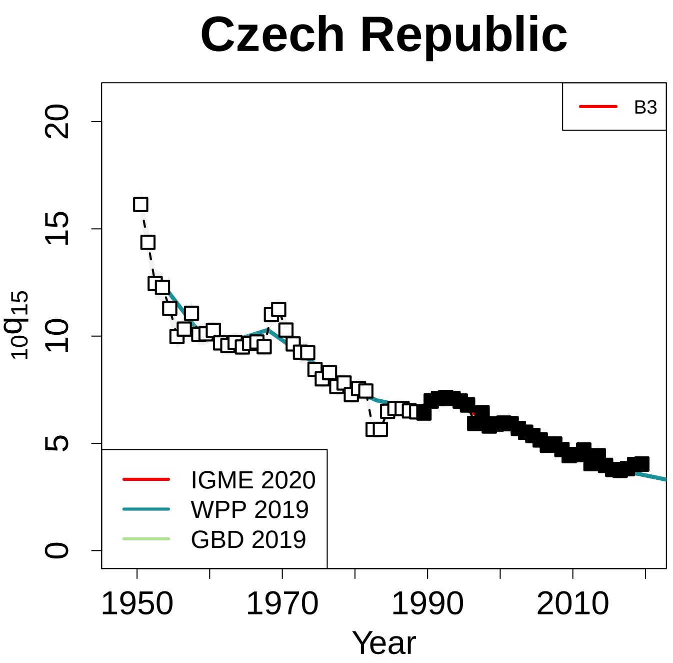
Czech Republic
B3
IGME 2020
WPP 2019
GBD 2019
0
5
10
15
20
$_{10}q_{15}$
1950
1970
1990
2010
Year

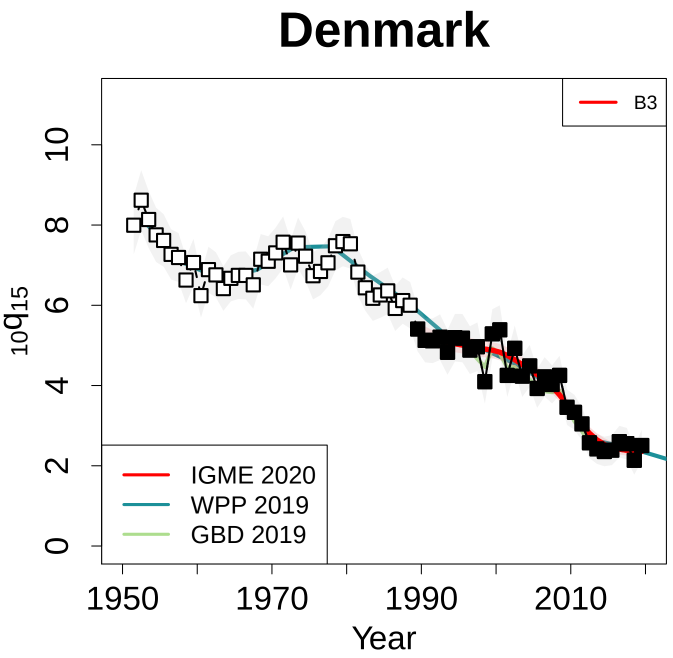
Denmark
B3
IGME 2020
WPP 2019
GBD 2019
0
2
4
6
8
10
$_{10}q_{15}$
1950
1970
1990
2010
Year

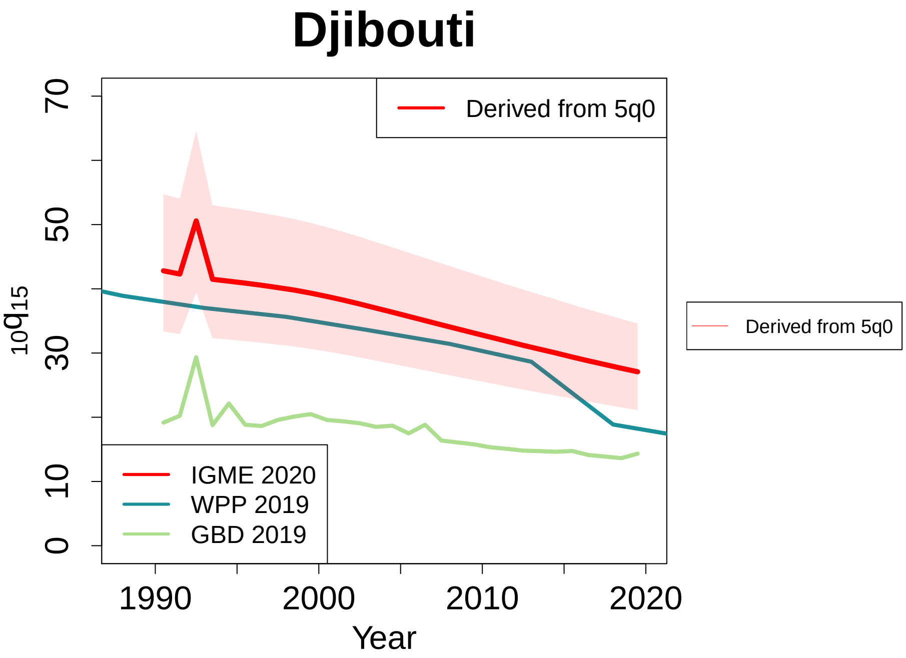
Djibouti
Derived from 5q0
IGME 2020
WPP 2019
GBD 2019
0
10
30
50
70
$_{10}q_{15}$
1990
2000
2010
2020
Year
Derived from 5q0

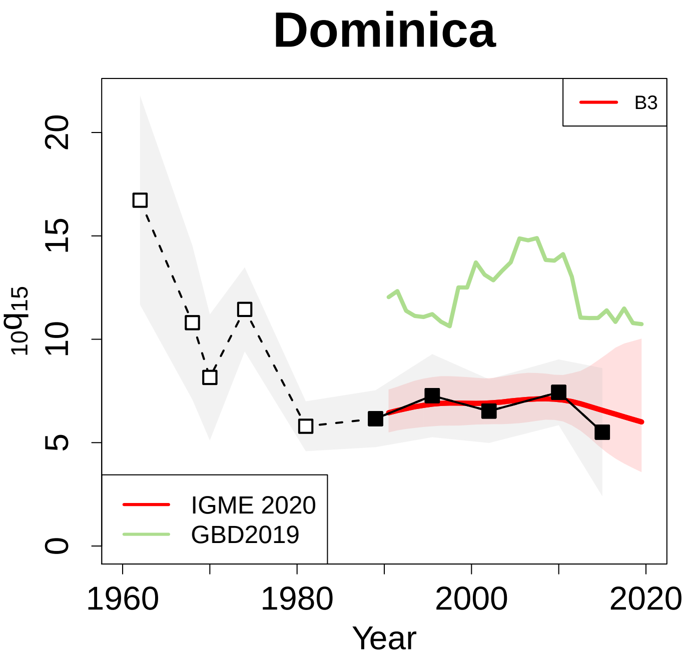
Dominica
B3
IGME 2020
GBD2019
0
5
10
15
20
$_{10}q_{15}$
1960
1980
2000
2020
Year

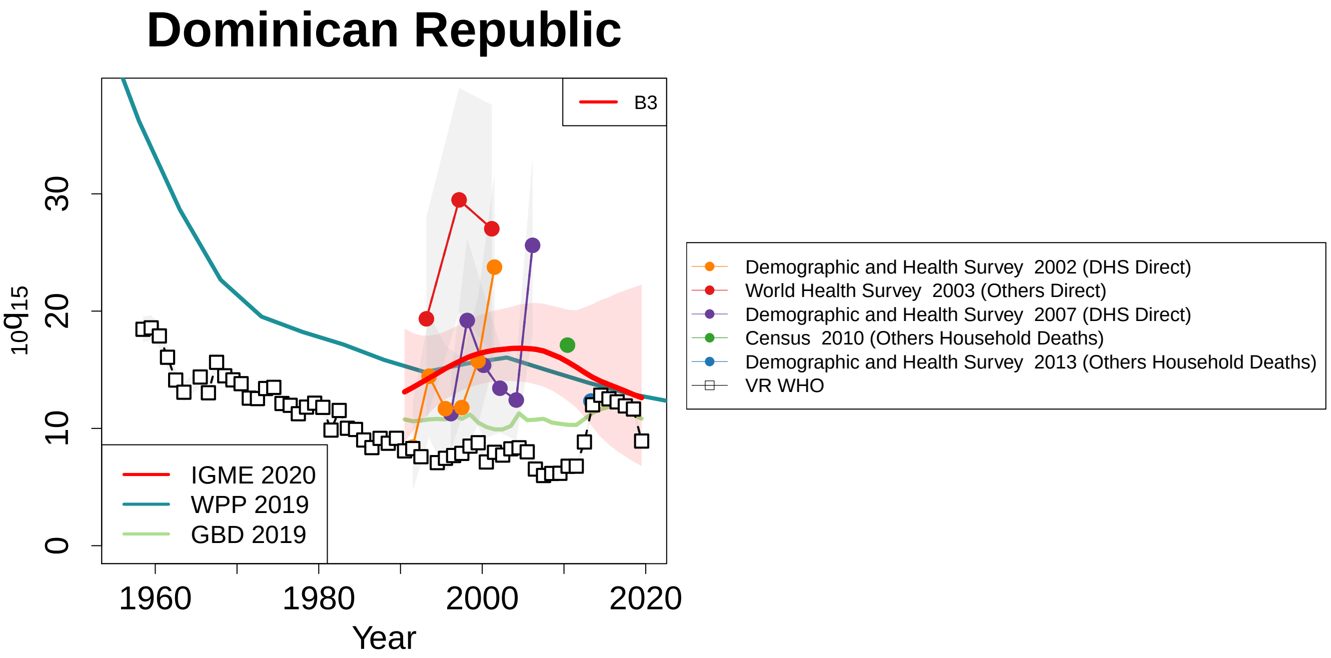
Dominican Republic
B3
IGME 2020
WPP 2019
GBD 2019
Demographic and Health Survey 2002 (DHS Direct)
World Health Survey 2003 (Others Direct)
Demographic and Health Survey 2007 (DHS Direct)
Census 2010 (Others Household Deaths)
Demographic and Health Survey 2013 (Others Household Deaths)
VR WHO
$_{10}q_{15}$
Year

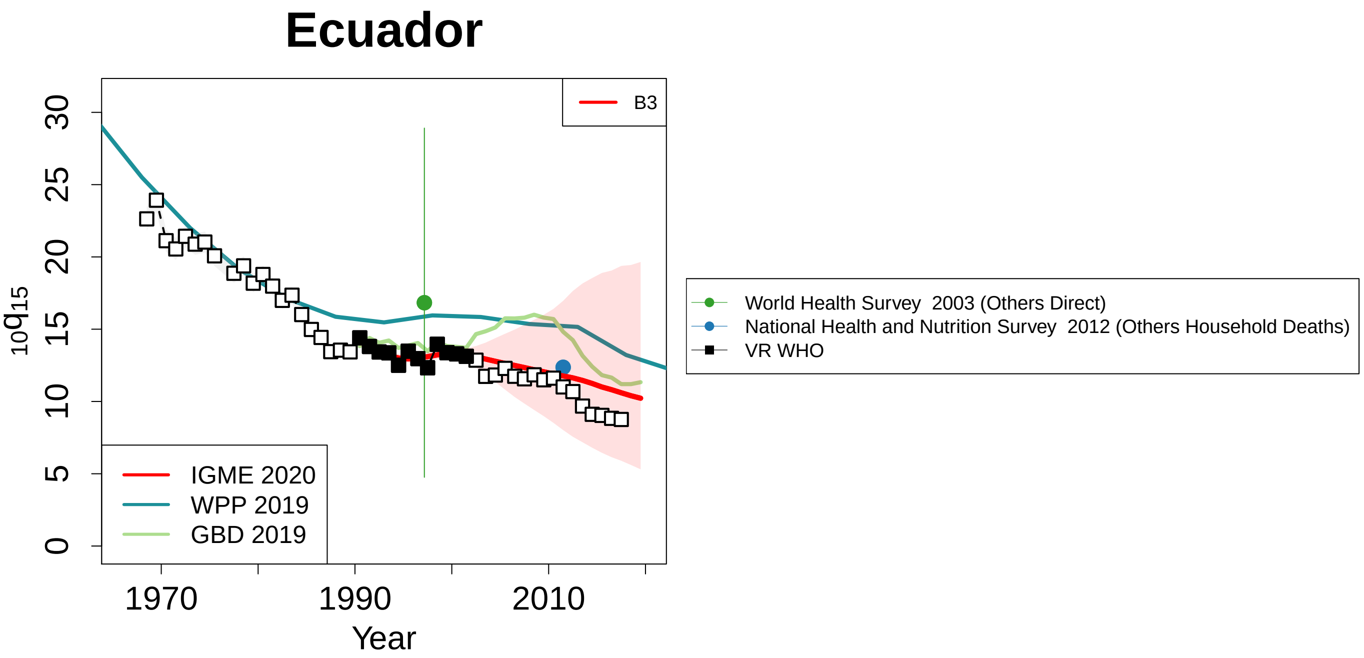
Ecuador
B3
IGME 2020
WPP 2019
GBD 2019
World Health Survey 2003 (Others Direct)
National Health and Nutrition Survey 2012 (Others Household Deaths)
VR WHO
$_{10}q_{15}$
Year

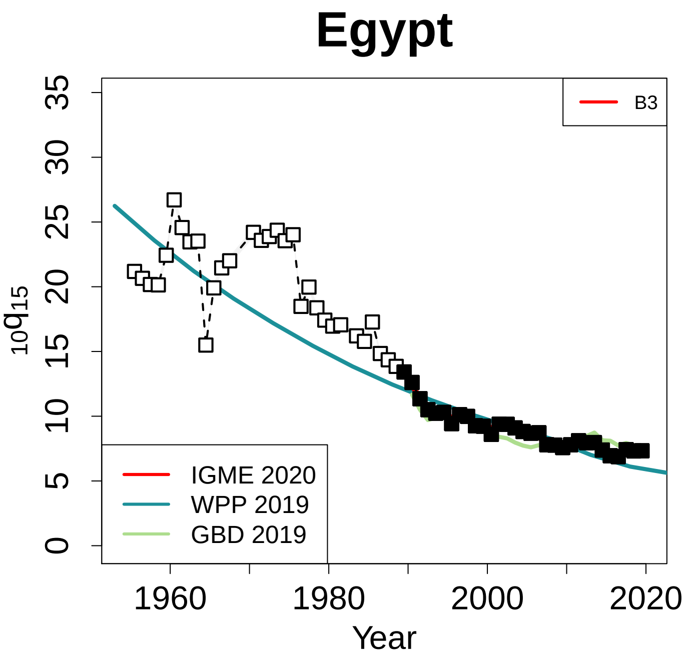
Egypt
B3
IGME 2020
WPP 2019
GBD 2019
$_{10}q_{15}$
0
5
10
15
20
25
30
35
1960
1980
2000
2020
Year

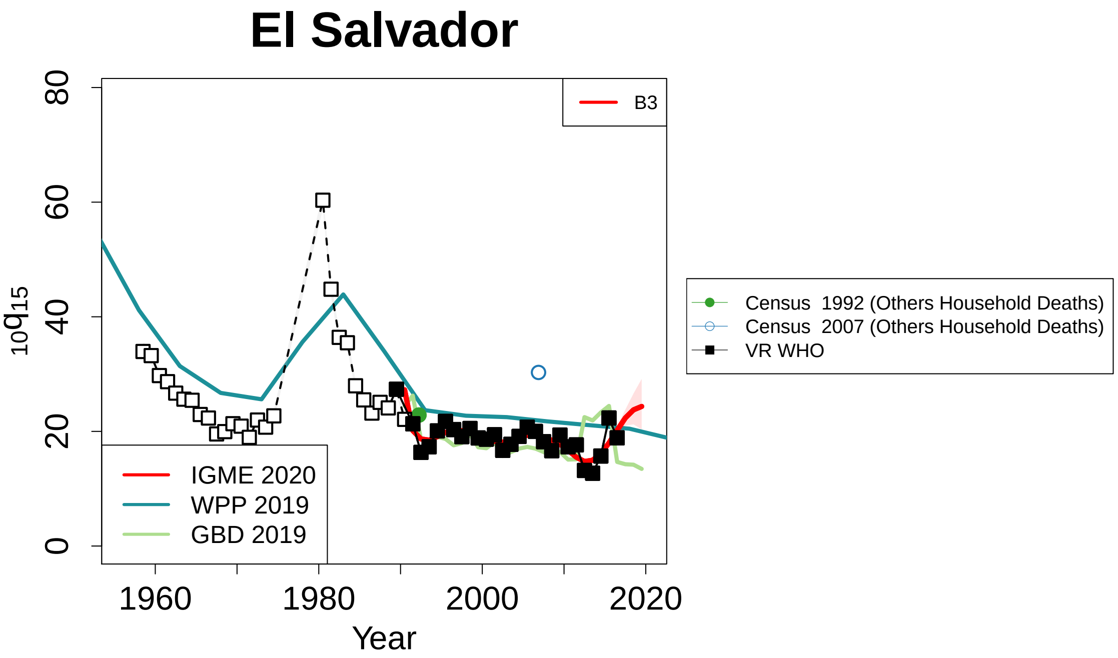
El Salvador
B3
IGME 2020
WPP 2019
GBD 2019
Census 1992 (Others Household Deaths)
Census 2007 (Others Household Deaths)
VR WHO
$_{10}q_{15}$
0
20
40
60
80
1960
1980
2000
2020
Year

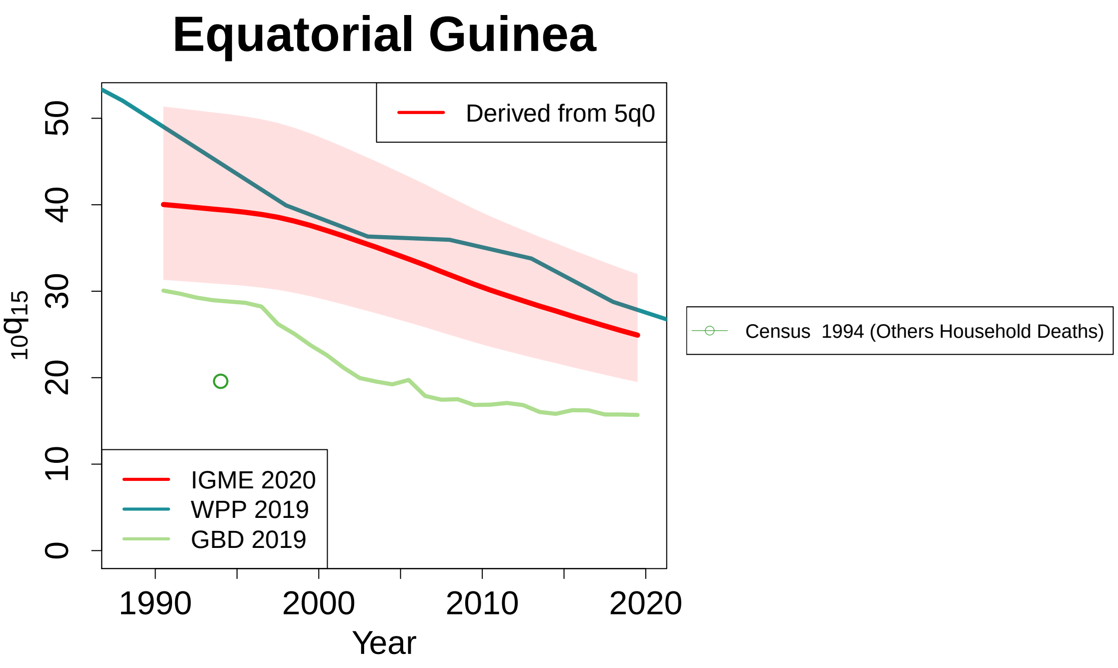
Equatorial Guinea
Derived from 5q0
Census 1994 (Others Household Deaths)
IGME 2020
WPP 2019
GBD 2019
0
10
20
30
40
50
$_{10}q_{15}$
1990
2000
2010
2020
Year

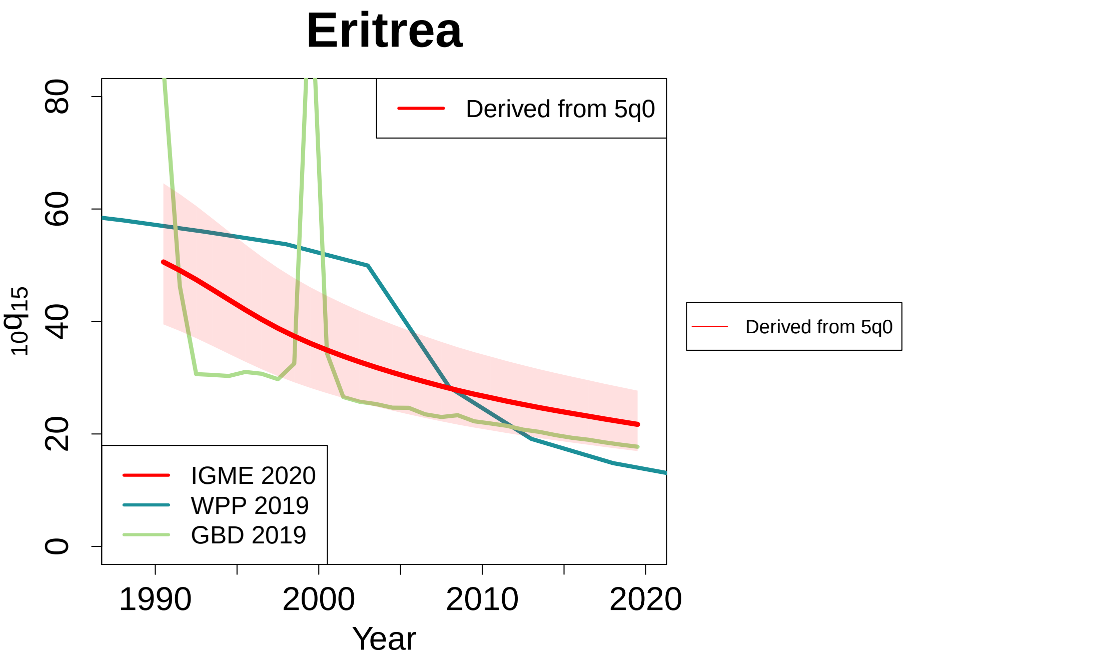
Eritrea
Derived from 5q0
Derived from 5q0
IGME 2020
WPP 2019
GBD 2019
0
20
40
60
80
$_{10}q_{15}$
1990
2000
2010
2020
Year

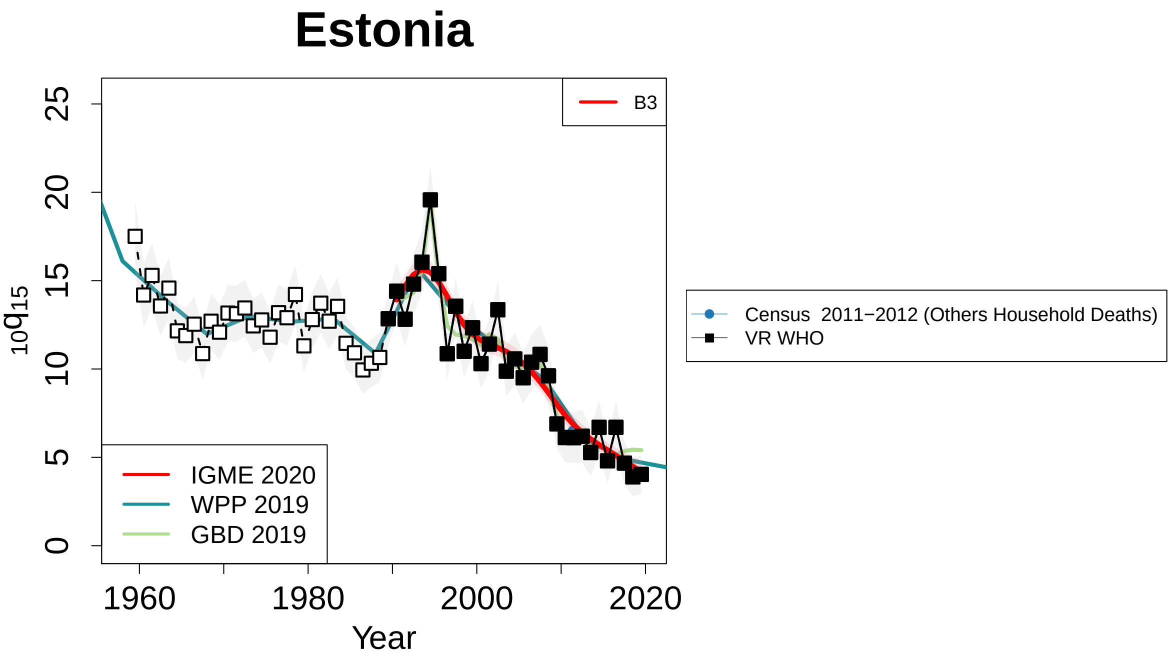
Estonia
B3
25
20
15
10
5
0
$_{10}q_{15}$
IGME 2020
WPP 2019
GBD 2019
1960
1980
2000
2020
Year
Census 2011−2012 (Others Household Deaths)
VR WHO

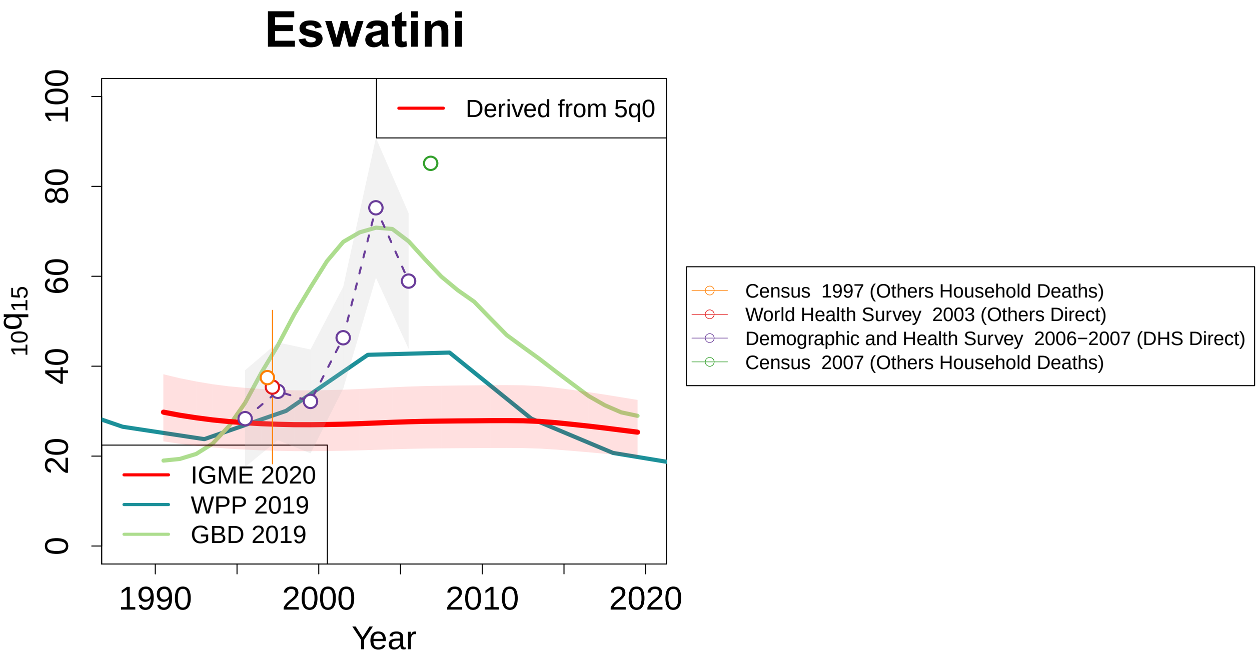
Eswatini
Derived from 5q0
100
80
60
40
20
0
$_{10}q_{15}$
IGME 2020
WPP 2019
GBD 2019
1990
2000
2010
2020
Year
Census 1997 (Others Household Deaths)
World Health Survey 2003 (Others Direct)
Demographic and Health Survey 2006−2007 (DHS Direct)
Census 2007 (Others Household Deaths)

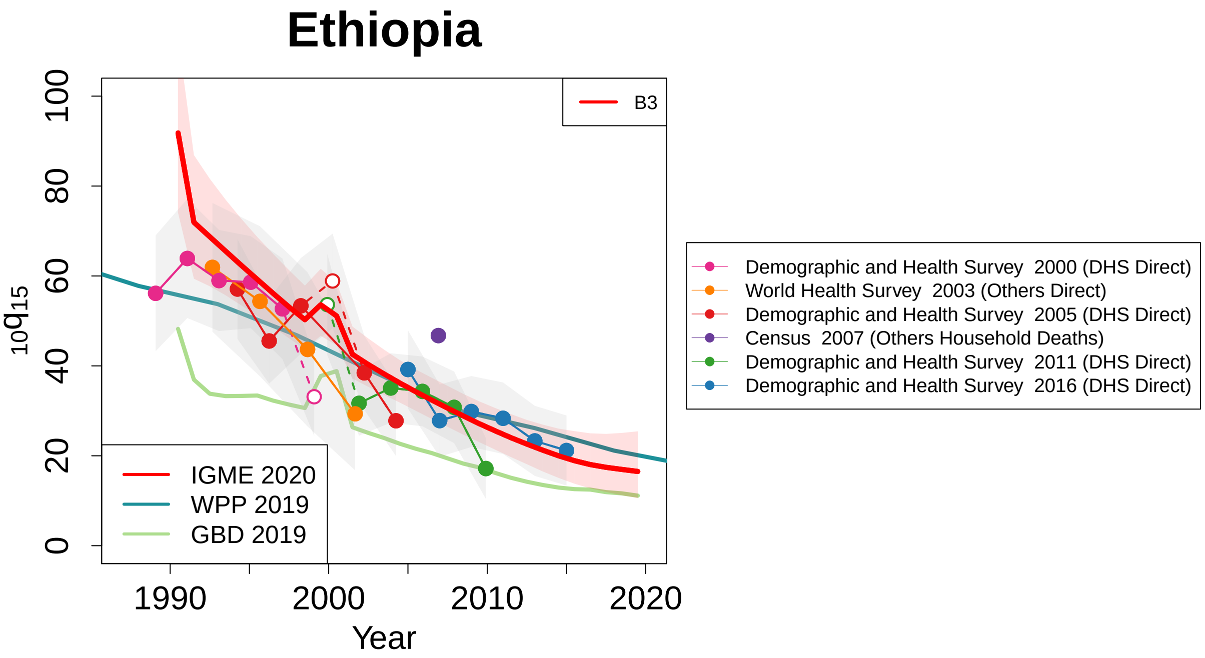
Ethiopia
B3
Demographic and Health Survey 2000 (DHS Direct)
World Health Survey 2003 (Others Direct)
Demographic and Health Survey 2005 (DHS Direct)
Census 2007 (Others Household Deaths)
Demographic and Health Survey 2011 (DHS Direct)
Demographic and Health Survey 2016 (DHS Direct)
IGME 2020
WPP 2019
GBD 2019
10q15
Year
0
20
40
60
80
100
1990
2000
2010
2020

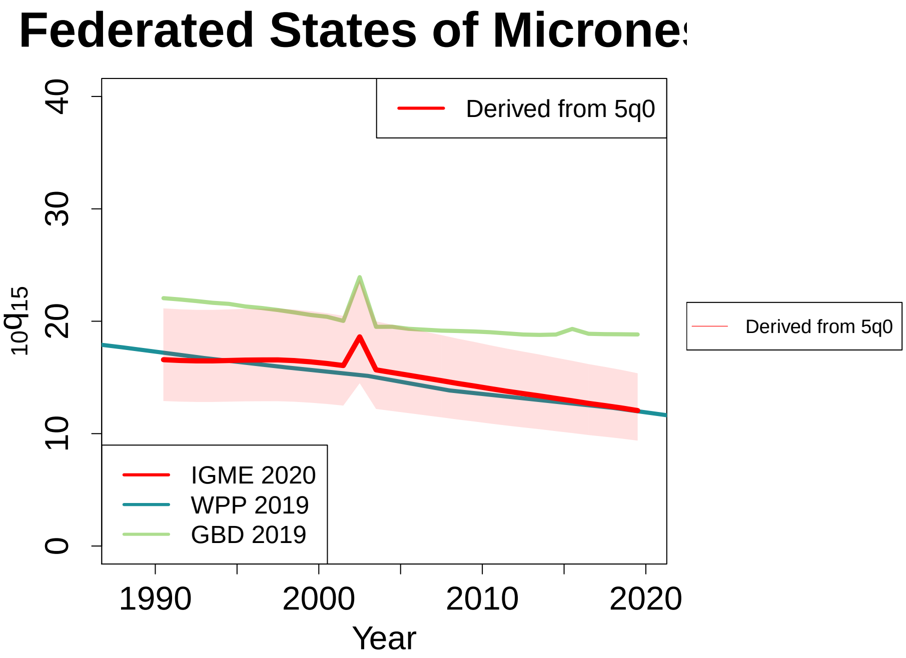
Federated States of Microne
Derived from 5q0
Derived from 5q0
IGME 2020
WPP 2019
GBD 2019
10q15
Year
0
10
20
30
40
1990
2000
2010
2020

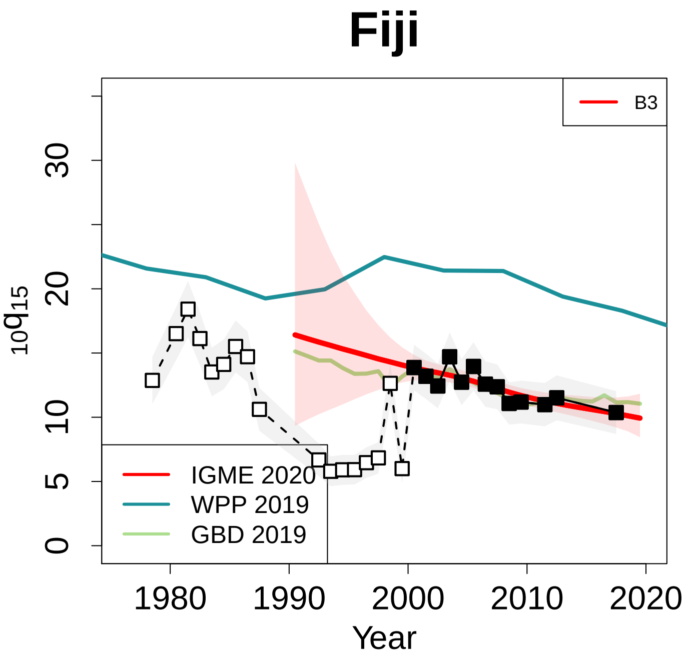
Fiji
B3
IGME 2020
WPP 2019
GBD 2019
$_{10}q_{15}$
0
5
10
20
30
1980
1990
2000
2010
2020
Year

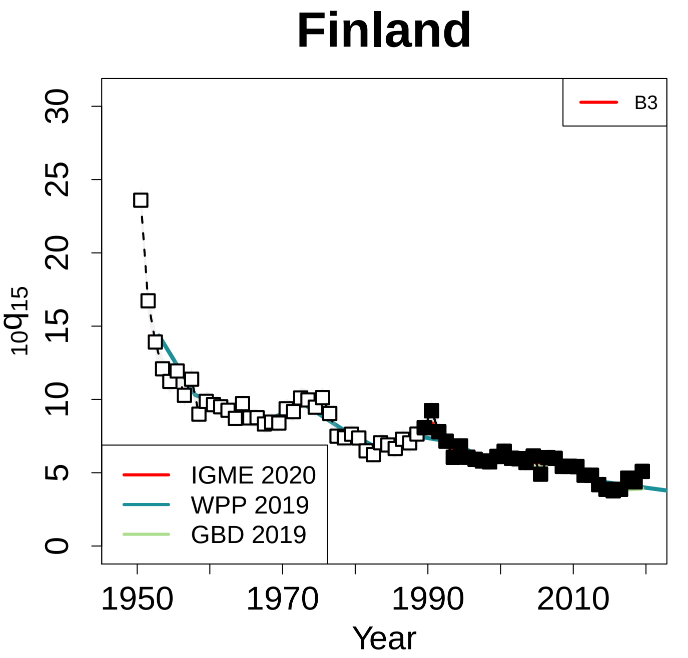
Finland
B3
IGME 2020
WPP 2019
GBD 2019
$_{10}q_{15}$
0
5
10
15
20
25
30
1950
1970
1990
2010
Year

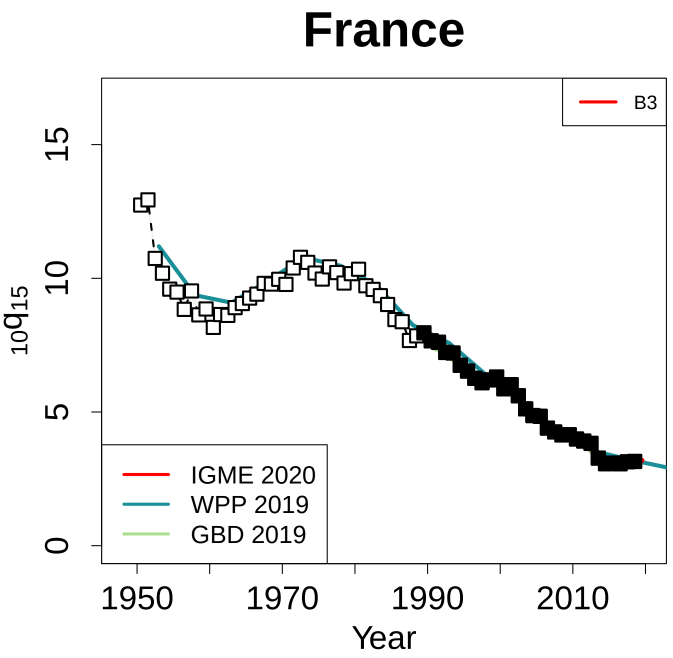
France
B3
IGME 2020
WPP 2019
GBD 2019
0
5
10
15
$_{10}q_{15}$
1950
1970
1990
2010
Year

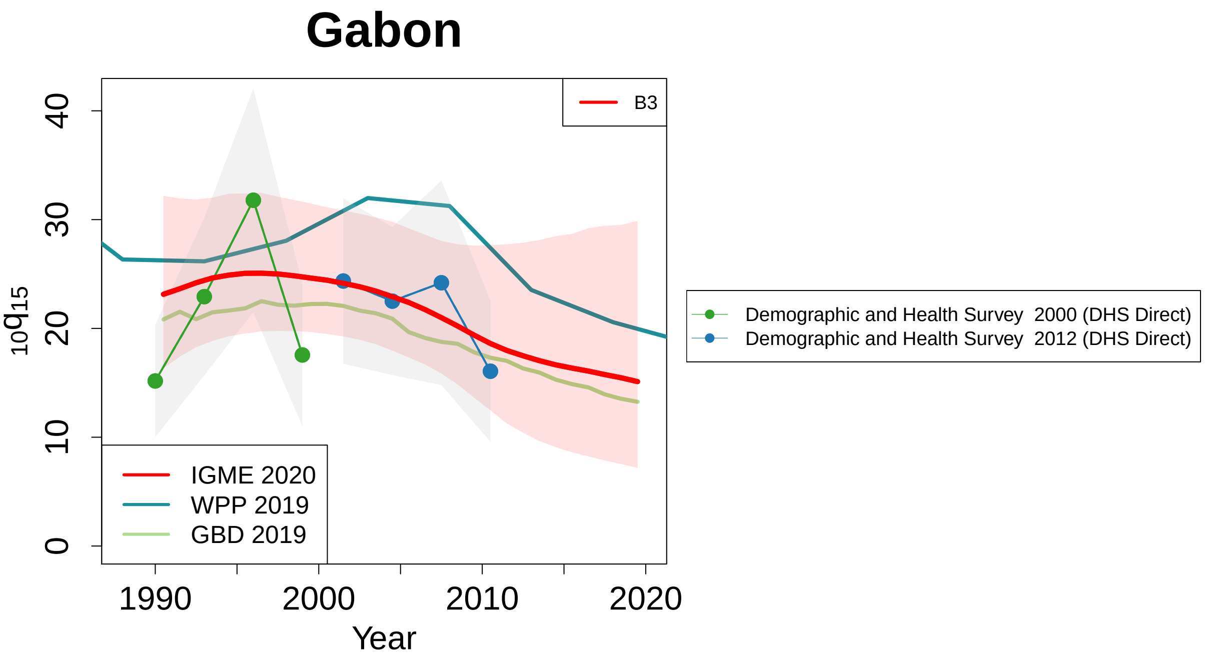
Gabon
B3
IGME 2020
WPP 2019
GBD 2019
Demographic and Health Survey 2000 (DHS Direct)
Demographic and Health Survey 2012 (DHS Direct)
0
10
20
30
40
$_{10}q_{15}$
1990
2000
2010
2020
Year

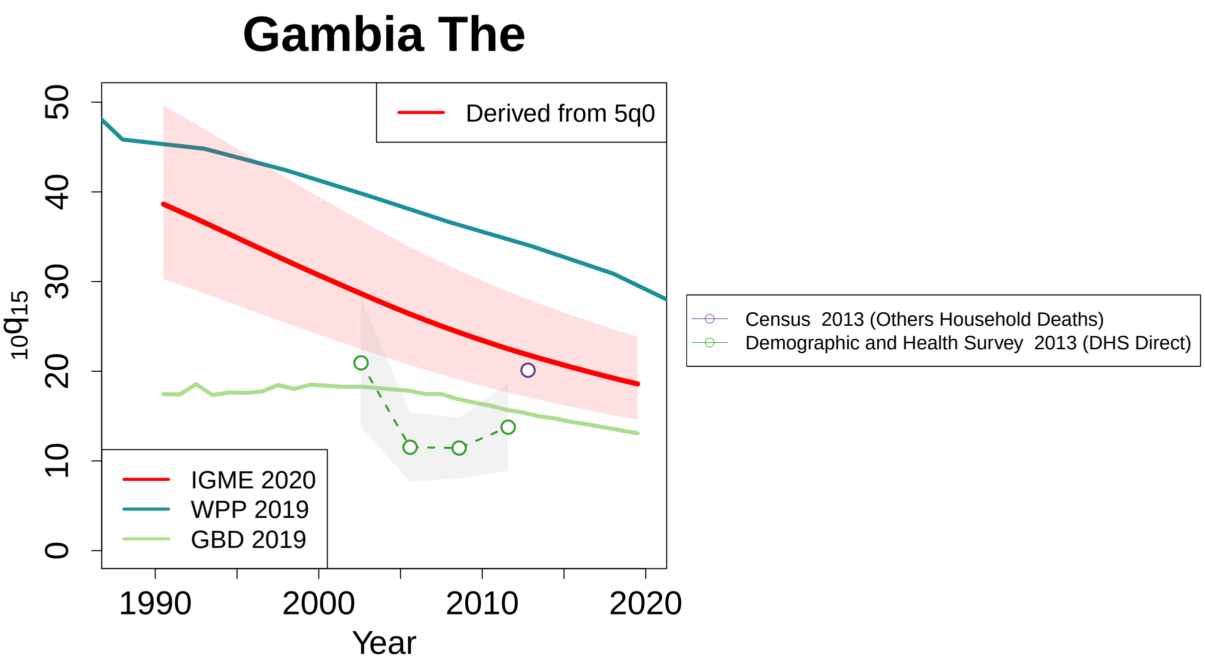
Gambia The
Derived from 5q0
Census 2013 (Others Household Deaths)
Demographic and Health Survey 2013 (DHS Direct)
IGME 2020
WPP 2019
GBD 2019
$_{10}q_{15}$
0
10
20
30
40
50
1990
2000
2010
2020
Year

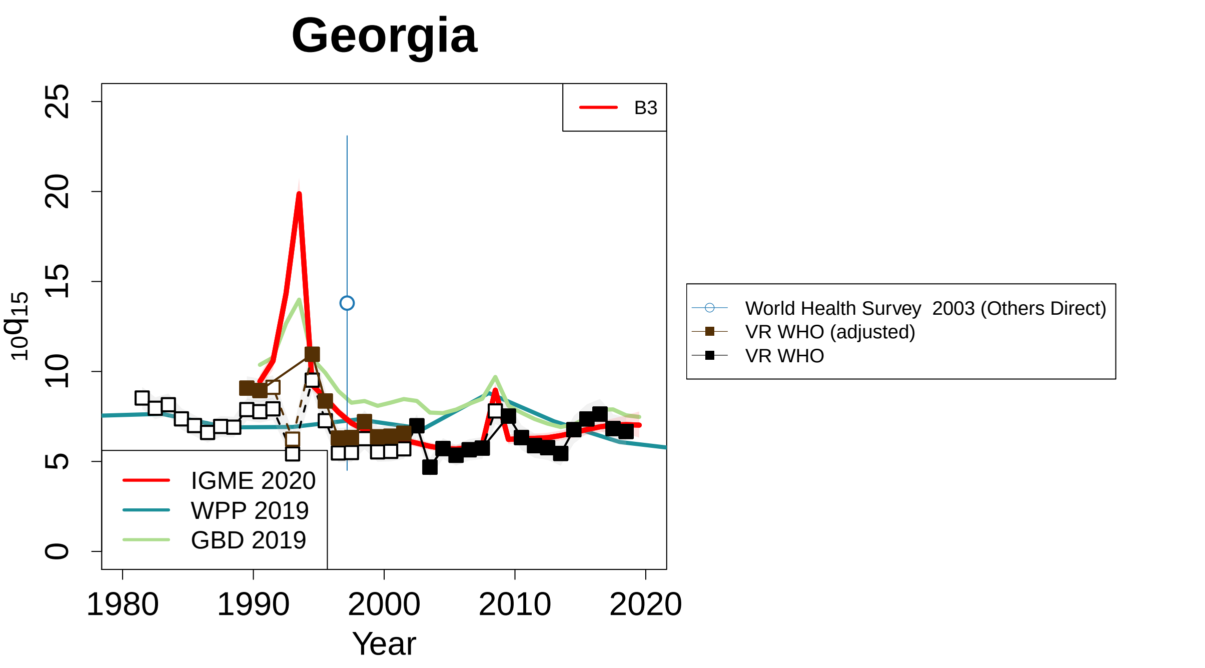
Georgia
B3
World Health Survey 2003 (Others Direct)
VR WHO (adjusted)
VR WHO
IGME 2020
WPP 2019
GBD 2019
$_{10}q_{15}$
0
5
10
15
20
25
1980
1990
2000
2010
2020
Year

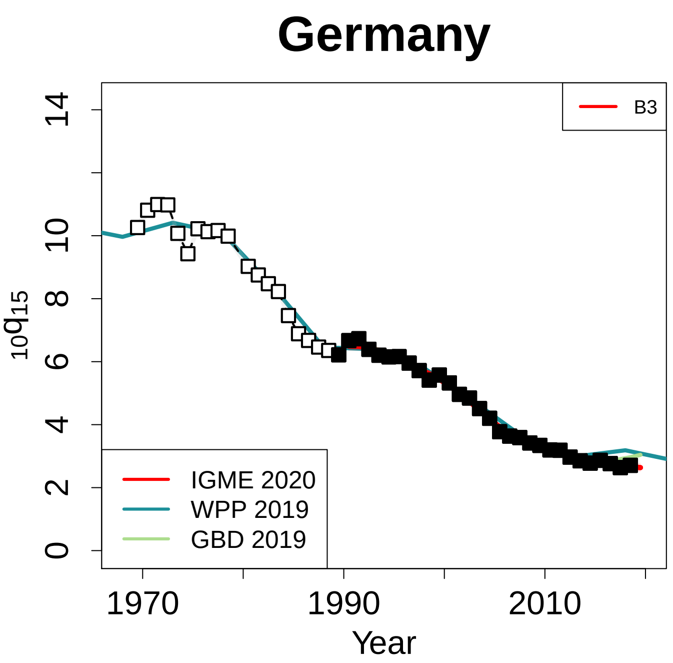
Germany
B3
14
10
8
6
4
2
0
$_{10}q_{15}$
IGME 2020
WPP 2019
GBD 2019
1970
1990
2010
Year

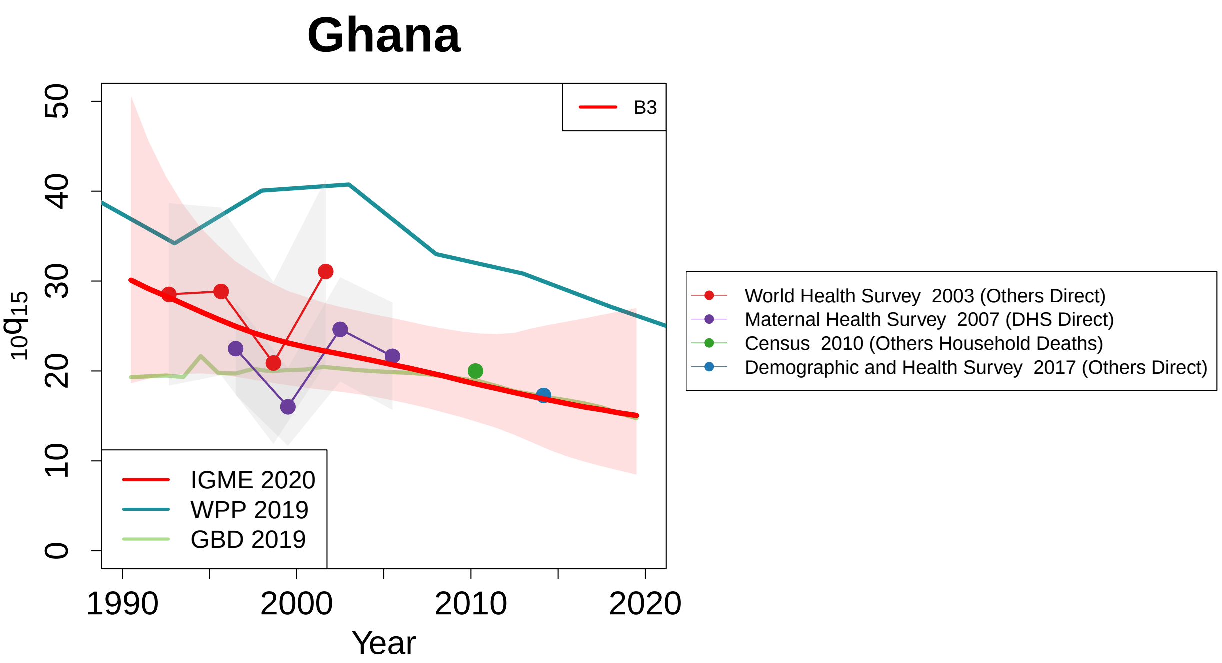
Ghana
B3
50
40
30
20
10
0
$_{10}q_{15}$
IGME 2020
WPP 2019
GBD 2019
1990
2000
2010
2020
Year
World Health Survey 2003 (Others Direct)
Maternal Health Survey 2007 (DHS Direct)
Census 2010 (Others Household Deaths)
Demographic and Health Survey 2017 (Others Direct)

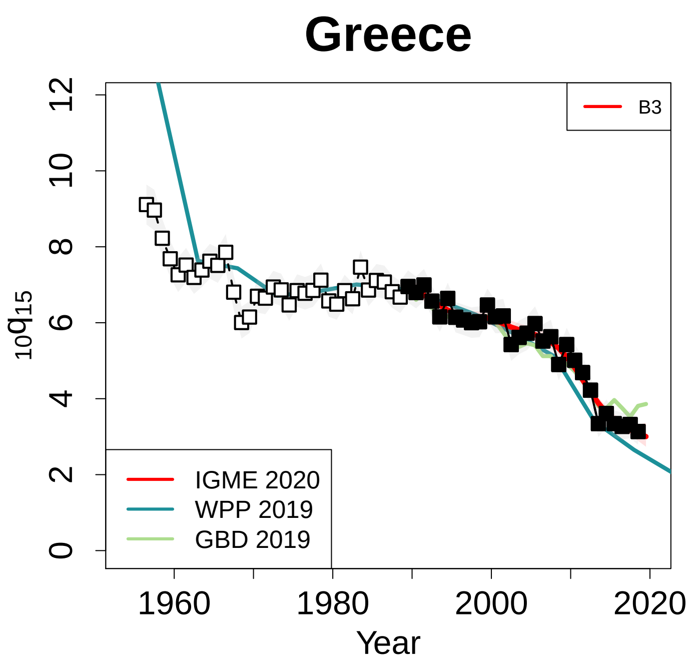
Greece
B3
IGME 2020
WPP 2019
GBD 2019
$_{10}q_{15}$
Year
0
2
4
6
8
10
12
1960
1980
2000
2020

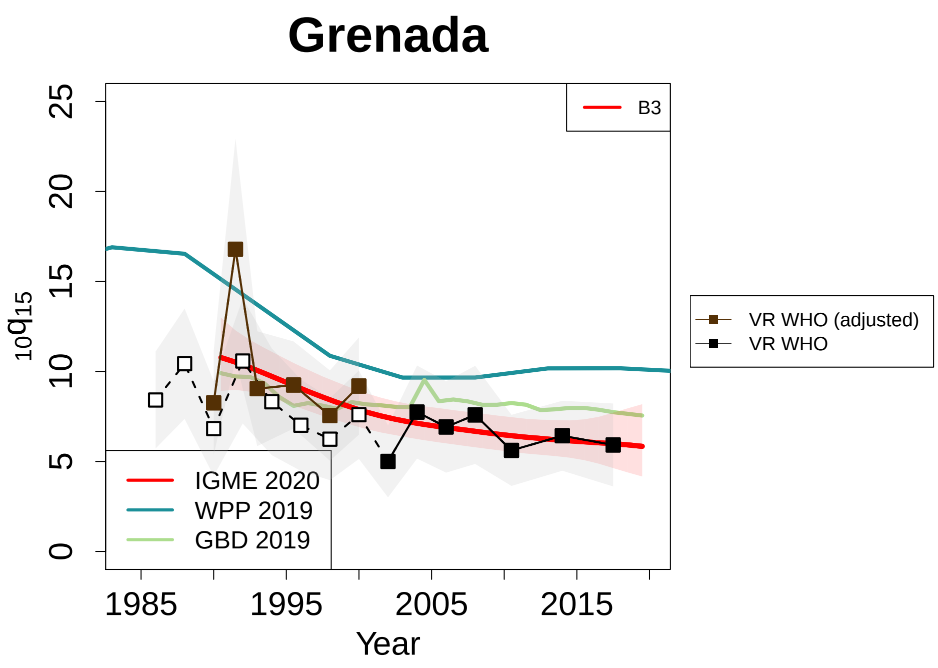
Grenada
B3
IGME 2020
WPP 2019
GBD 2019
VR WHO (adjusted)
VR WHO
$_{10}q_{15}$
Year
0
5
10
15
20
25
1985
1995
2005
2015

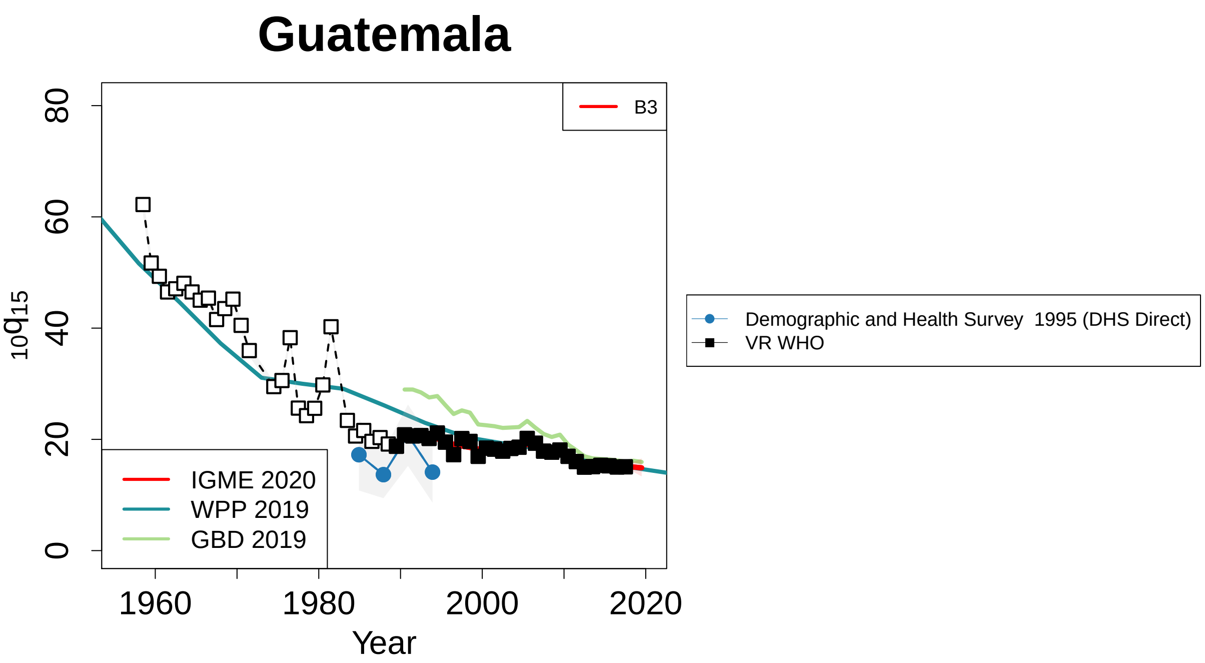
Guatemala
B3
IGME 2020
WPP 2019
GBD 2019
Demographic and Health Survey 1995 (DHS Direct)
VR WHO
$_{10}q_{15}$
Year
0
20
40
60
80
1960
1980
2000
2020

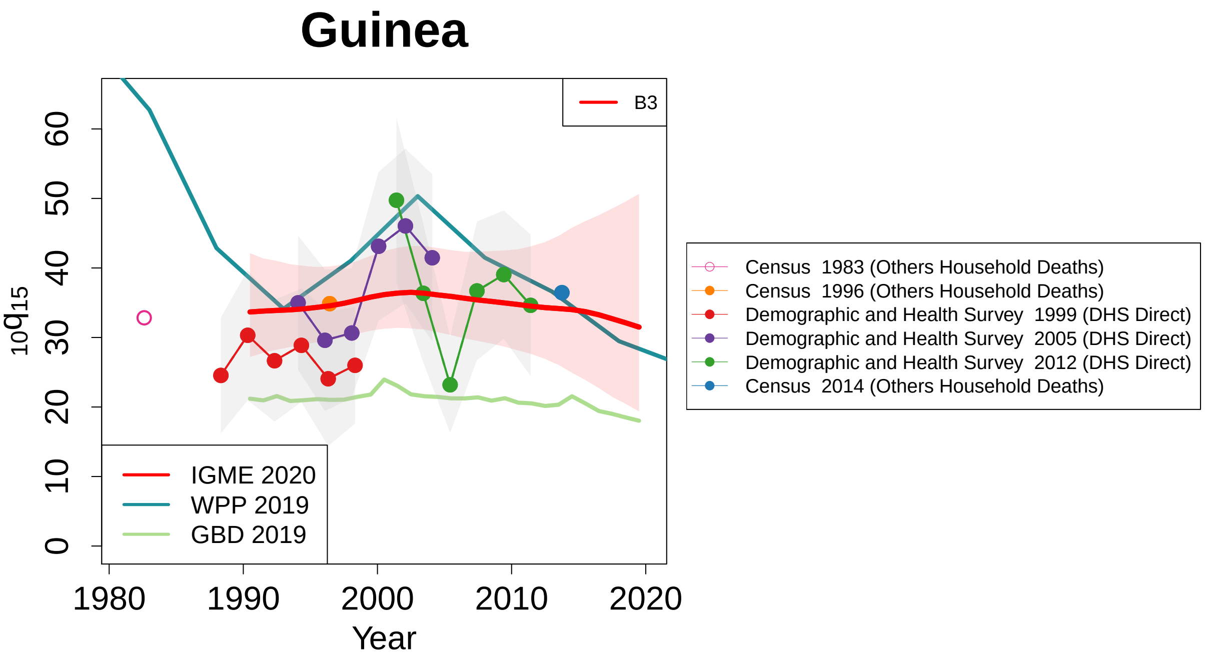
Guinea
B3
IGME 2020
WPP 2019
GBD 2019
Census 1983 (Others Household Deaths)
Census 1996 (Others Household Deaths)
Demographic and Health Survey 1999 (DHS Direct)
Demographic and Health Survey 2005 (DHS Direct)
Demographic and Health Survey 2012 (DHS Direct)
Census 2014 (Others Household Deaths)
$_{10}q_{15}$
Year
0
10
20
30
40
50
60
1980
1990
2000
2010
2020

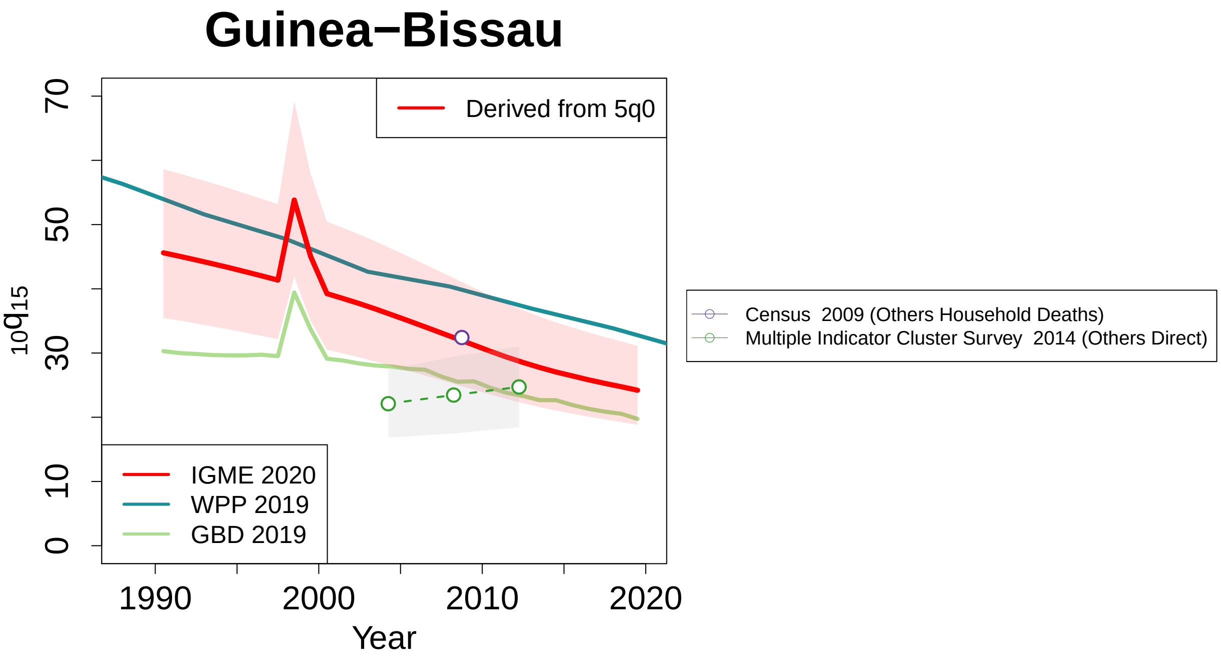
Guinea−Bissau
Derived from 5q0
IGME 2020
WPP 2019
GBD 2019
Census 2009 (Others Household Deaths)
Multiple Indicator Cluster Survey 2014 (Others Direct)
$_{10}q_{15}$
0
10
30
50
70
1990
2000
2010
2020
Year

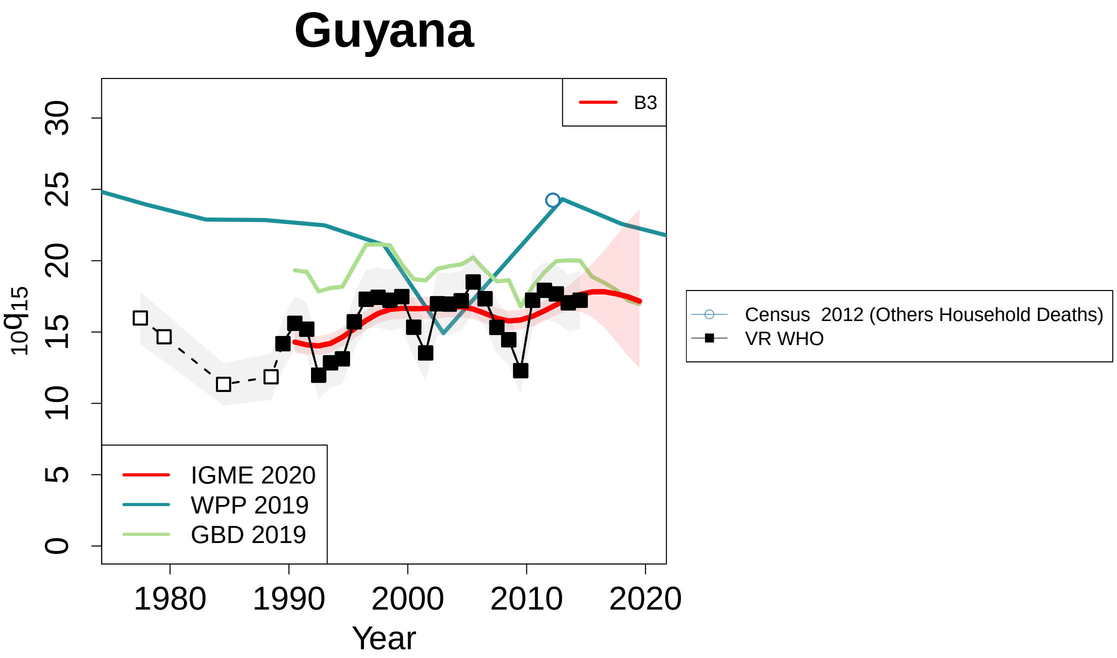
Guyana
B3
IGME 2020
WPP 2019
GBD 2019
Census 2012 (Others Household Deaths)
VR WHO
$_{10}q_{15}$
0
5
10
15
20
25
30
1980
1990
2000
2010
2020
Year

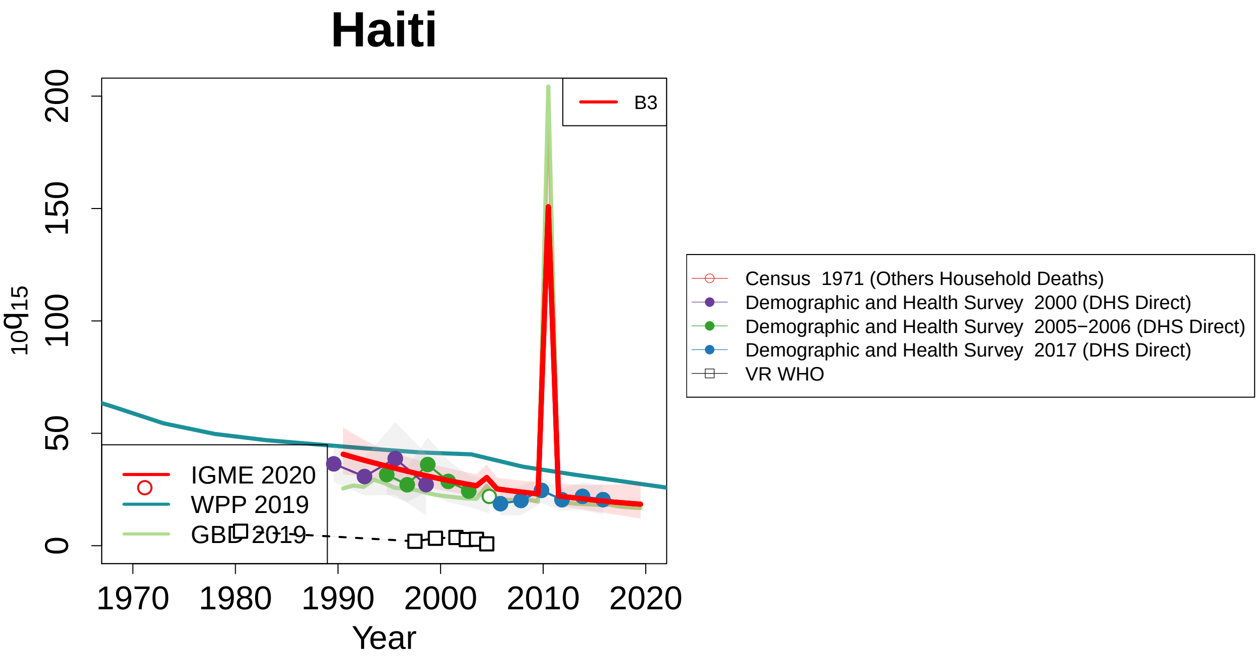
Haiti
B3
IGME 2020
WPP 2019
GBD 2019
Census 1971 (Others Household Deaths)
Demographic and Health Survey 2000 (DHS Direct)
Demographic and Health Survey 2005−2006 (DHS Direct)
Demographic and Health Survey 2017 (DHS Direct)
VR WHO
10q15
Year
0
50
100
150
200
1970
1980
1990
2000
2010
2020

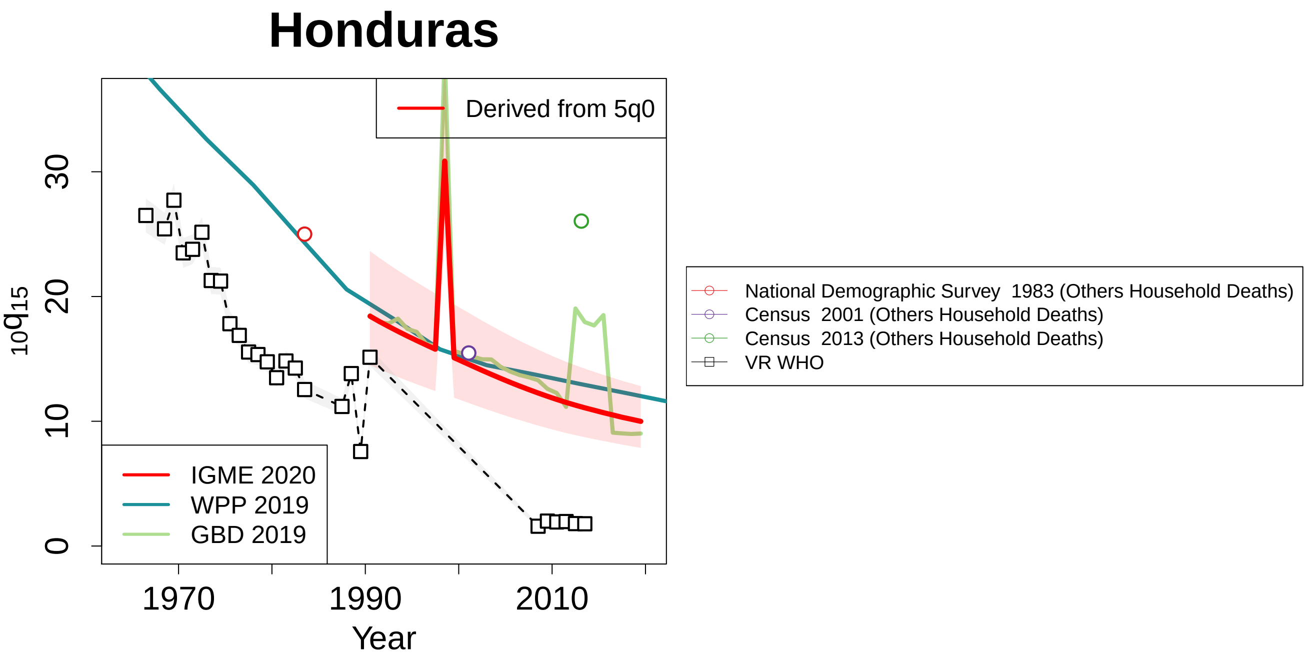
Honduras
Derived from 5q0
IGME 2020
WPP 2019
GBD 2019
National Demographic Survey 1983 (Others Household Deaths)
Census 2001 (Others Household Deaths)
Census 2013 (Others Household Deaths)
VR WHO
10q15
Year
0
10
20
30
1970
1990
2010

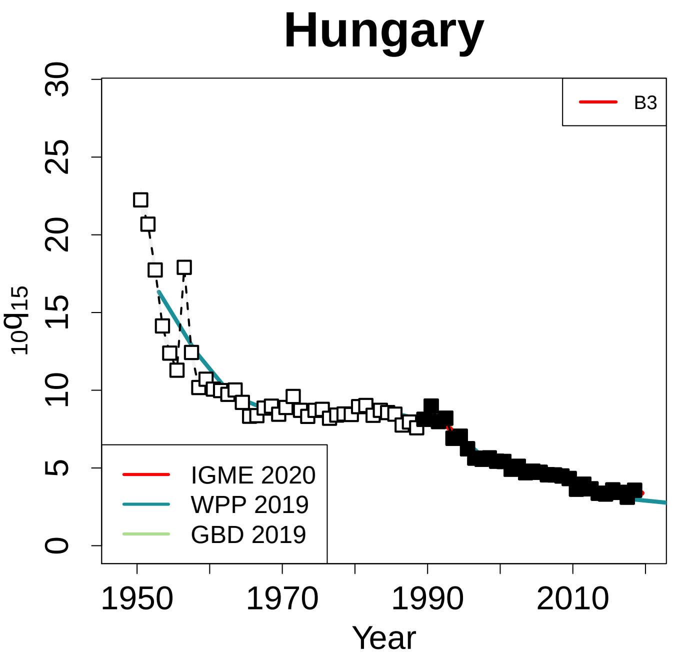
Hungary
B3
IGME 2020
WPP 2019
GBD 2019
$_{10}q_{15}$
0
5
10
15
20
25
30
1950
1970
1990
2010
Year

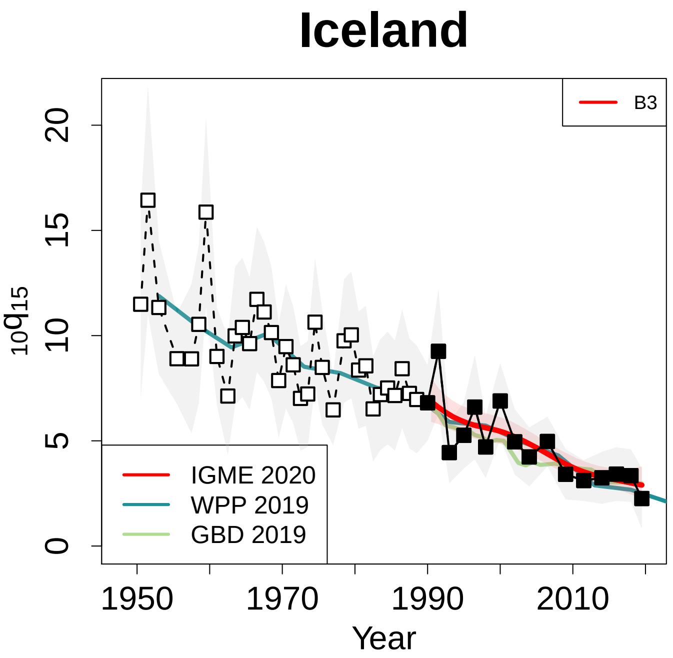
Iceland
B3
IGME 2020
WPP 2019
GBD 2019
$_{10}q_{15}$
0
5
10
15
20
1950
1970
1990
2010
Year

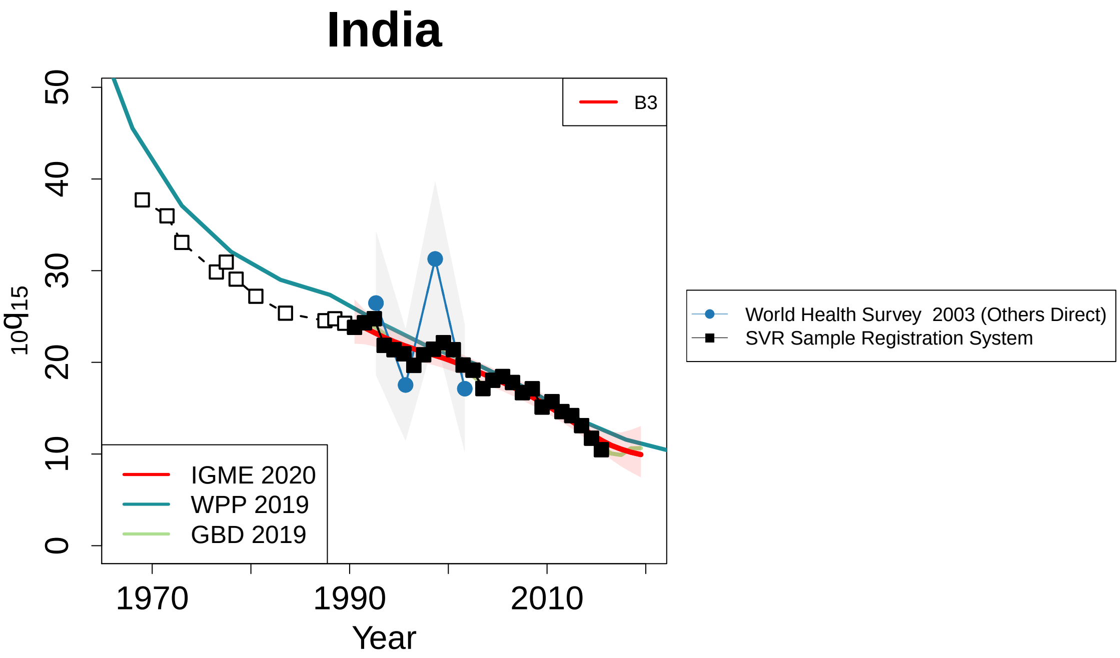
India
50
40
30
20
10
0
$_{10}q_{15}$
1970
1990
2010
Year
B3
IGME 2020
WPP 2019
GBD 2019
World Health Survey 2003 (Others Direct)
SVR Sample Registration System

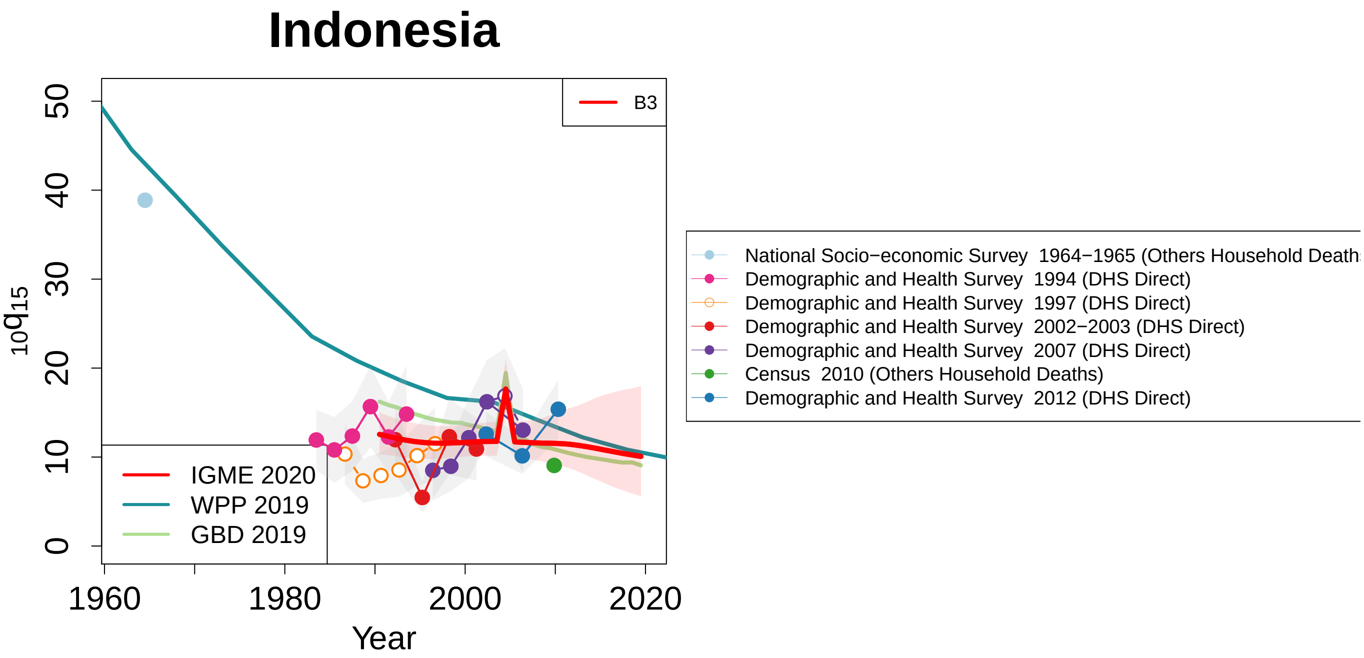
Indonesia
50
40
30
20
10
0
$_{10}q_{15}$
1960
1980
2000
2020
Year
B3
IGME 2020
WPP 2019
GBD 2019
National Socio−economic Survey 1964−1965 (Others Household Death
Demographic and Health Survey 1994 (DHS Direct)
Demographic and Health Survey 1997 (DHS Direct)
Demographic and Health Survey 2002−2003 (DHS Direct)
Demographic and Health Survey 2007 (DHS Direct)
Census 2010 (Others Household Deaths)
Demographic and Health Survey 2012 (DHS Direct)

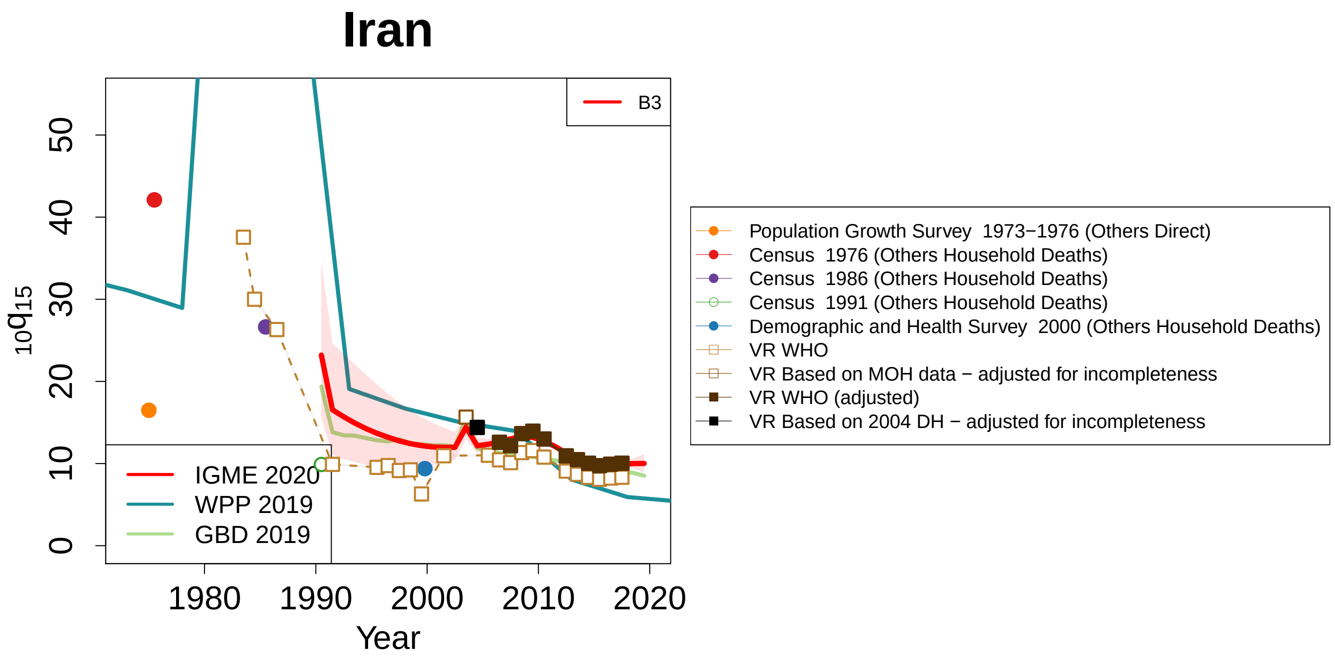
Iran
B3
Population Growth Survey 1973−1976 (Others Direct)
Census 1976 (Others Household Deaths)
Census 1986 (Others Household Deaths)
Census 1991 (Others Household Deaths)
Demographic and Health Survey 2000 (Others Household Deaths)
VR WHO
VR Based on MOH data − adjusted for incompleteness
VR WHO (adjusted)
VR Based on 2004 DH − adjusted for incompleteness
IGME 2020
WPP 2019
GBD 2019
10q15
0
10
20
30
40
50
1980
1990
2000
2010
2020
Year

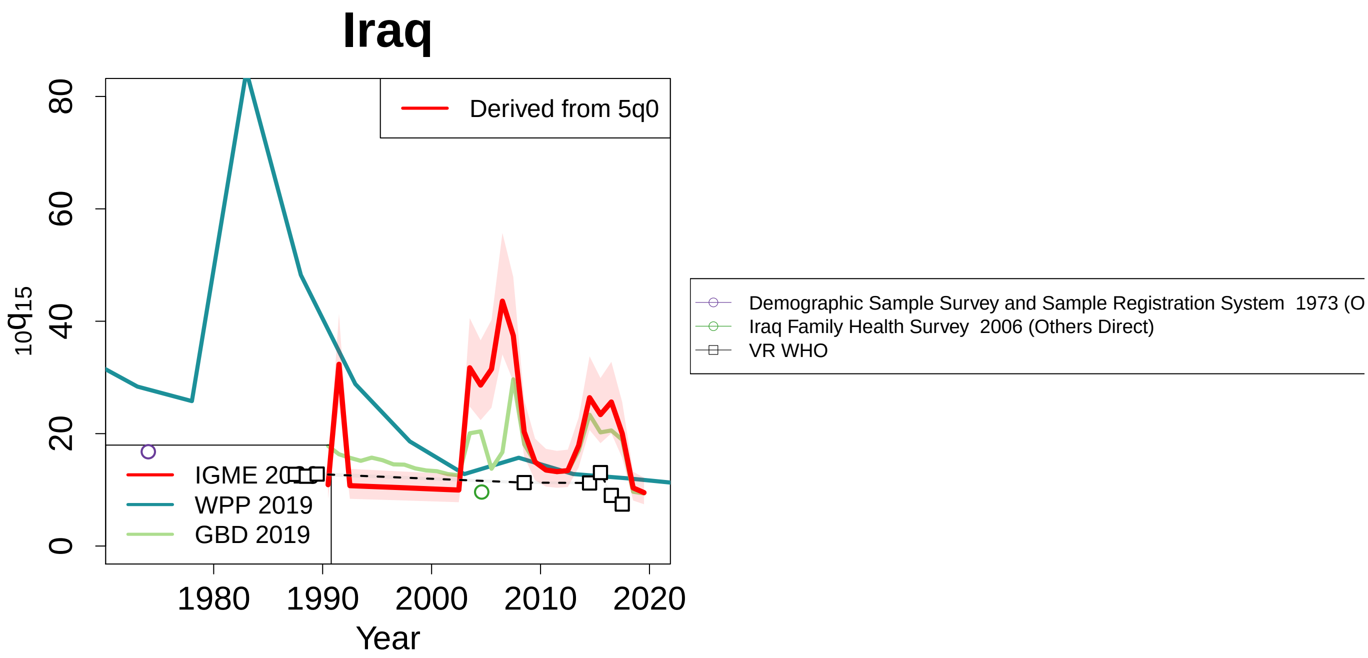
Iraq
Derived from 5q0
Demographic Sample Survey and Sample Registration System 1973 (O
Iraq Family Health Survey 2006 (Others Direct)
VR WHO
IGME 2020
WPP 2019
GBD 2019
10q15
0
20
40
60
80
1980
1990
2000
2010
2020
Year

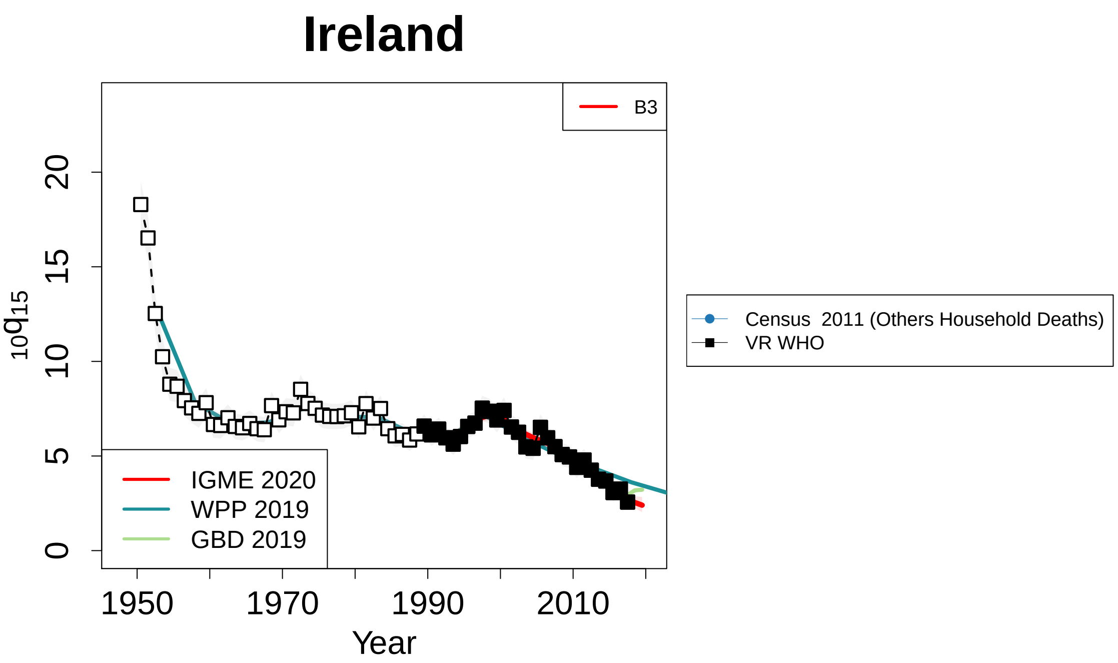
Ireland
B3
IGME 2020
WPP 2019
GBD 2019
Census 2011 (Others Household Deaths)
VR WHO
$_{10}q_{15}$
0
5
10
15
20
1950
1970
1990
2010
Year

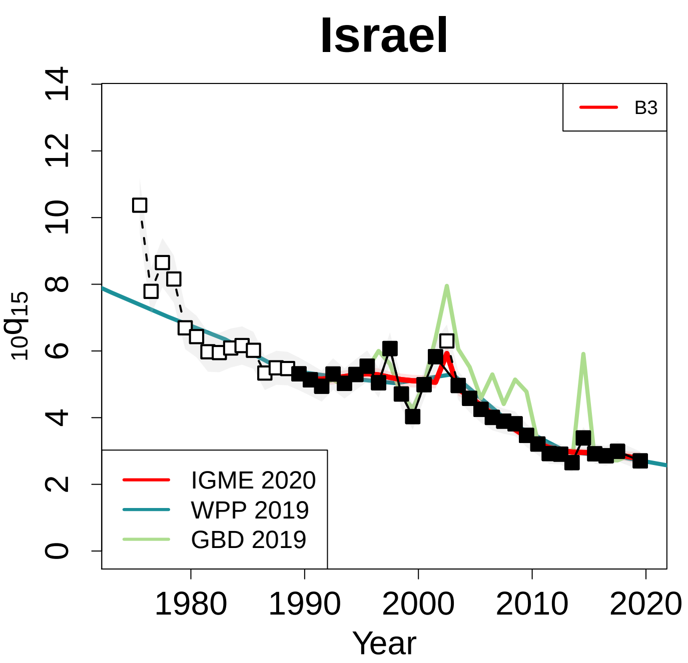
Israel
B3
IGME 2020
WPP 2019
GBD 2019
$_{10}q_{15}$
0
2
4
6
8
10
12
14
1980
1990
2000
2010
2020
Year

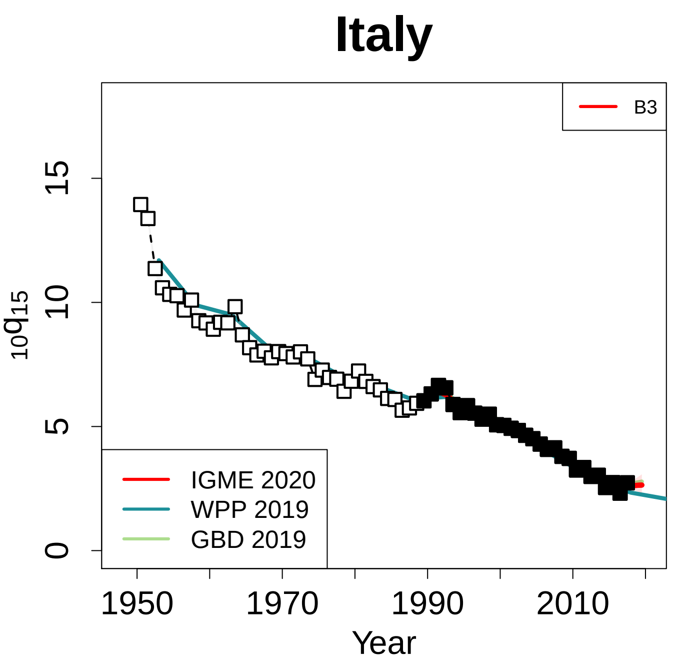
Italy
B3
IGME 2020
WPP 2019
GBD 2019
$_{10}q_{15}$
0
5
10
15
1950
1970
1990
2010
Year

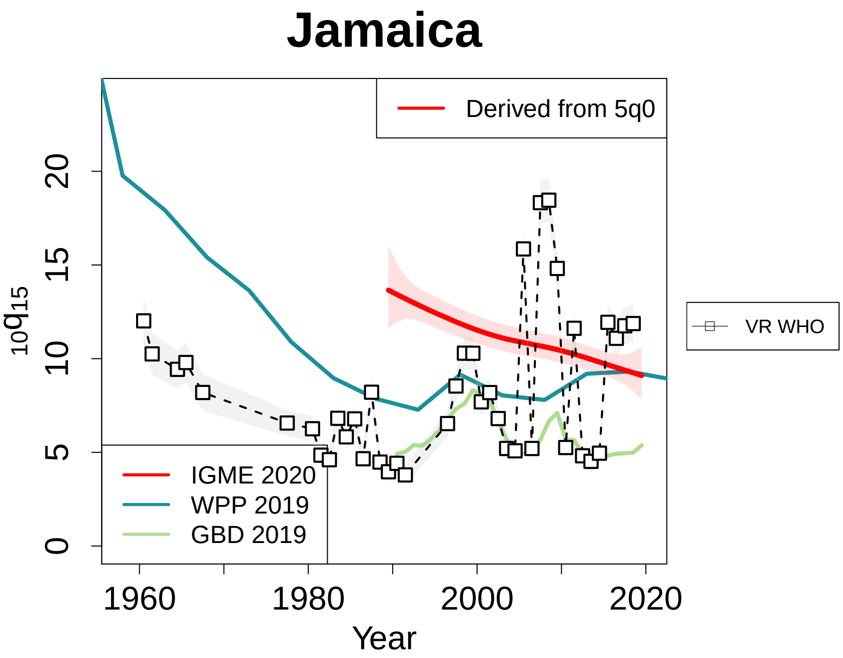
Jamaica
Derived from 5q0
VR WHO
IGME 2020
WPP 2019
GBD 2019
$_{10}q_{15}$
0
5
10
15
20
1960
1980
2000
2020
Year

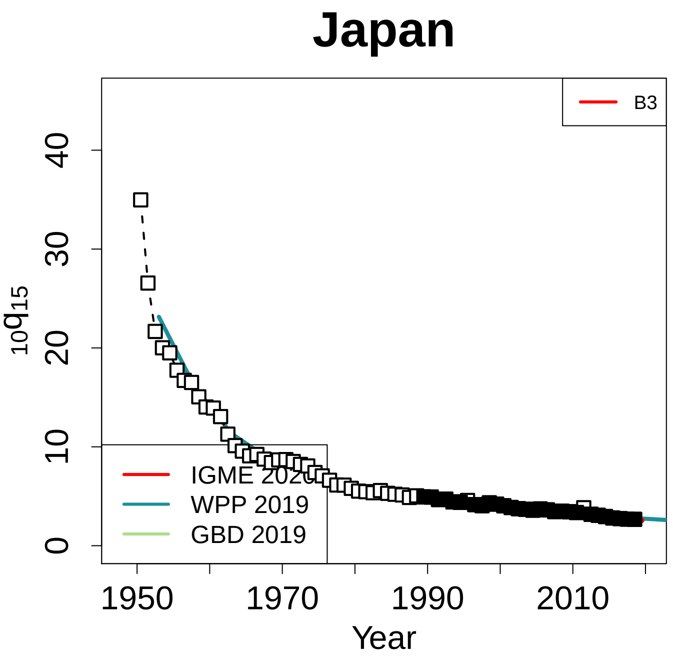
Japan
B3
IGME 2020
WPP 2019
GBD 2019
$_{10}q_{15}$
0
10
20
30
40
1950
1970
1990
2010
Year

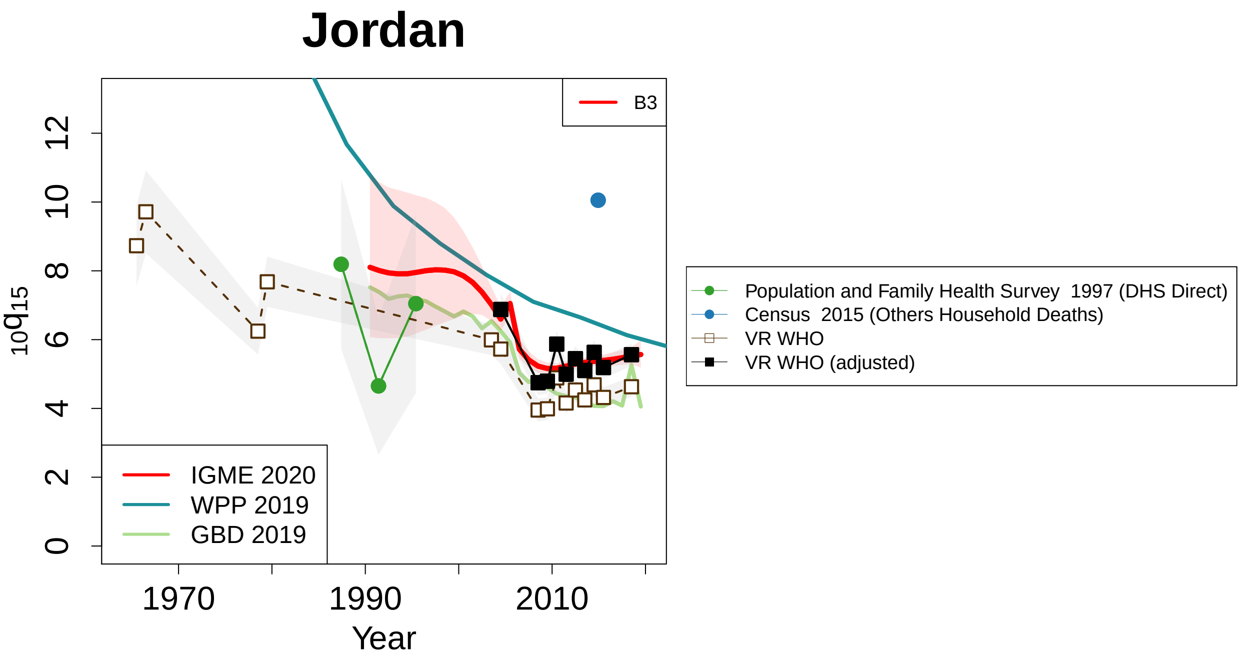
Jordan
B3
IGME 2020
WPP 2019
GBD 2019
Population and Family Health Survey 1997 (DHS Direct)
Census 2015 (Others Household Deaths)
VR WHO
VR WHO (adjusted)
$_{10}q_{15}$
0
2
4
6
8
10
12
1970
1990
2010
Year

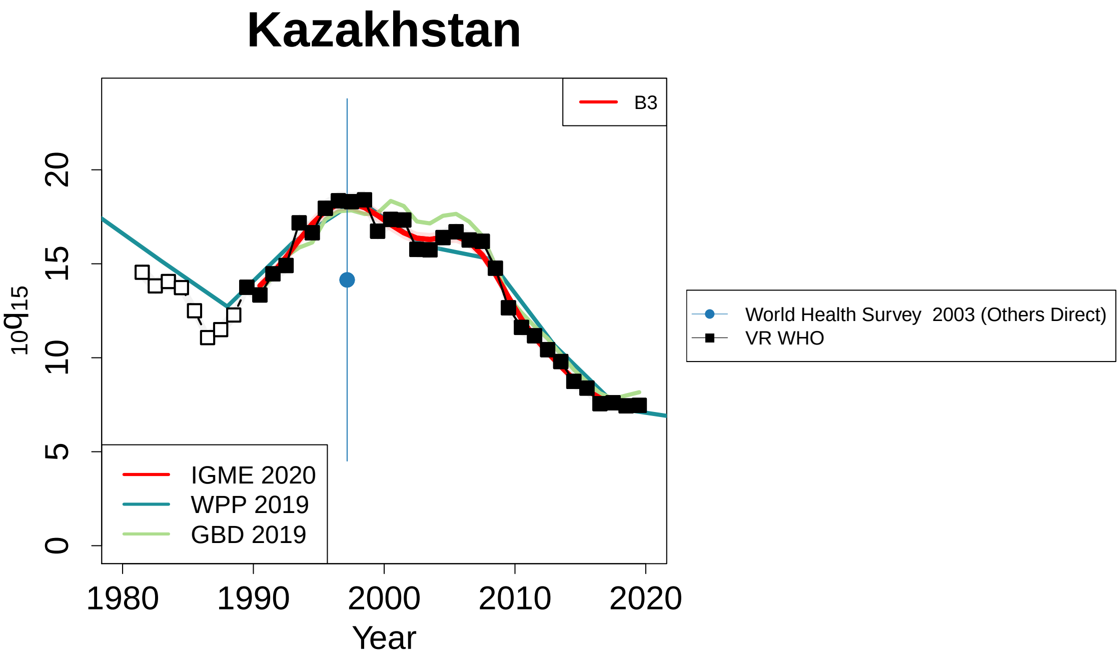
Kazakhstan
B3
$_{10}q_{15}$
0
5
10
15
20
1980
1990
2000
2010
2020
Year
IGME 2020
WPP 2019
GBD 2019
World Health Survey 2003 (Others Direct)
VR WHO

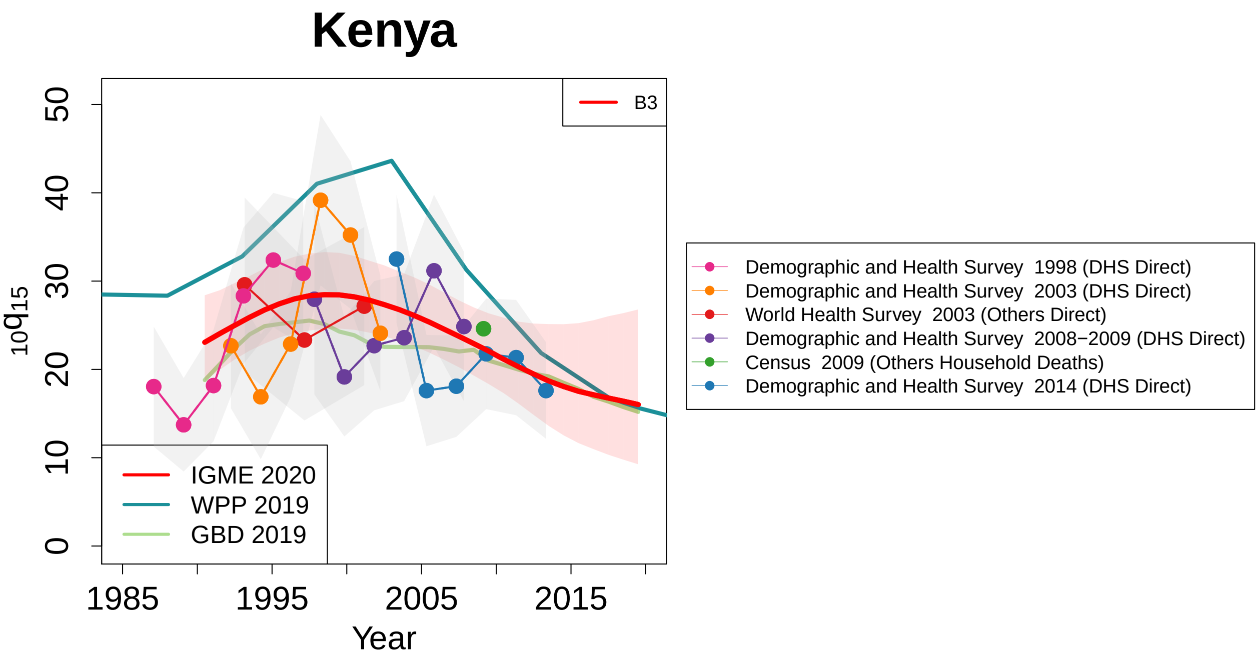
Kenya
B3
$_{10}q_{15}$
0
10
20
30
40
50
1985
1995
2005
2015
Year
IGME 2020
WPP 2019
GBD 2019
Demographic and Health Survey 1998 (DHS Direct)
Demographic and Health Survey 2003 (DHS Direct)
World Health Survey 2003 (Others Direct)
Demographic and Health Survey 2008−2009 (DHS Direct)
Census 2009 (Others Household Deaths)
Demographic and Health Survey 2014 (DHS Direct)

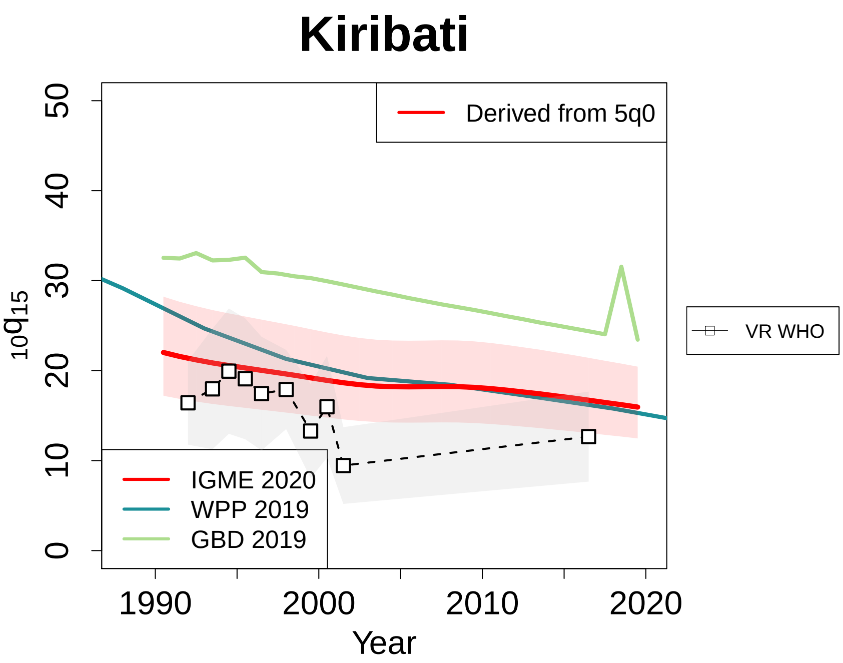
Kiribati
Derived from 5q0
VR WHO
IGME 2020
WPP 2019
GBD 2019
0
10
20
30
40
50
$_{10}q_{15}$
1990
2000
2010
2020
Year

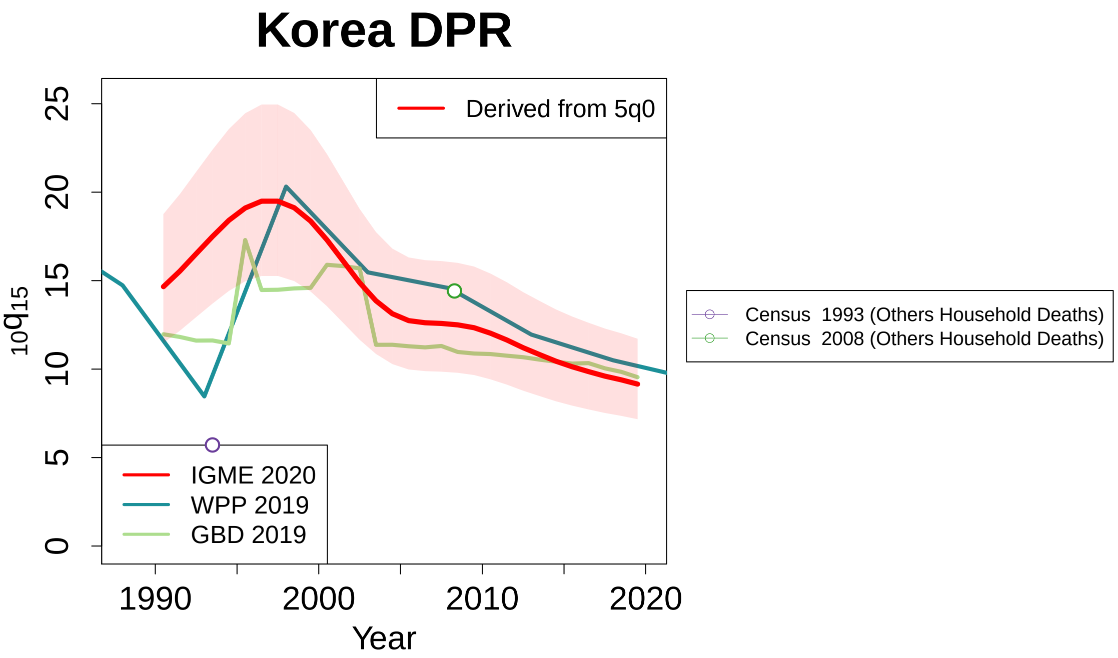
Korea DPR
Derived from 5q0
Census 1993 (Others Household Deaths)
Census 2008 (Others Household Deaths)
IGME 2020
WPP 2019
GBD 2019
0
5
10
15
20
25
$_{10}q_{15}$
1990
2000
2010
2020
Year

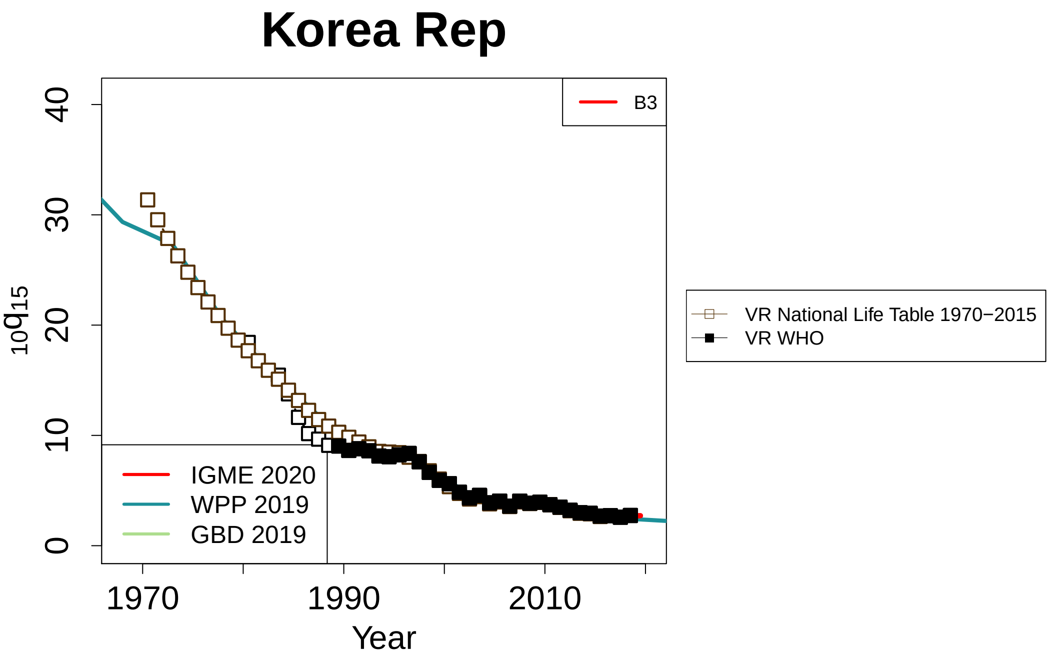

Korea Rep
B3
VR National Life Table 1970−2015
VR WHO
IGME 2020
WPP 2019
GBD 2019
$_{10}q_{15}$
0
10
20
30
40
1970
1990
2010
Year

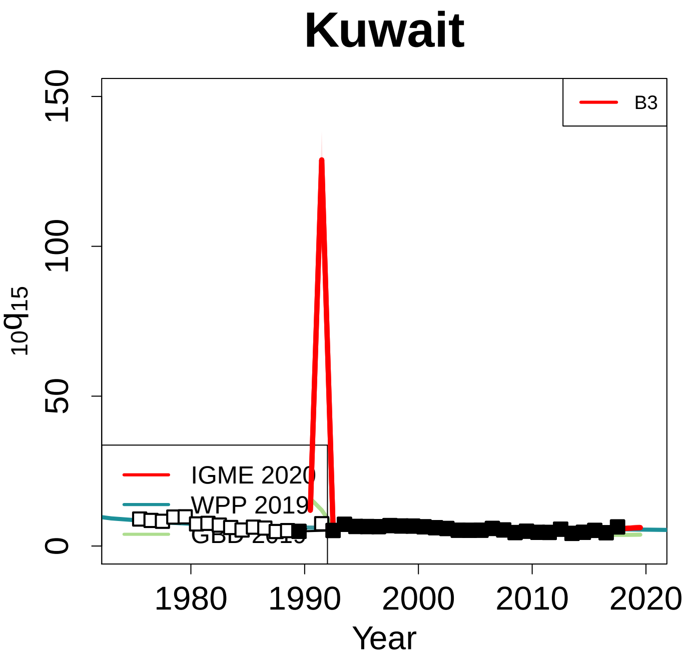

Kuwait
B3
IGME 2020
WPP 2019
$_{10}q_{15}$
0
50
100
150
1980
1990
2000
2010
2020
Year

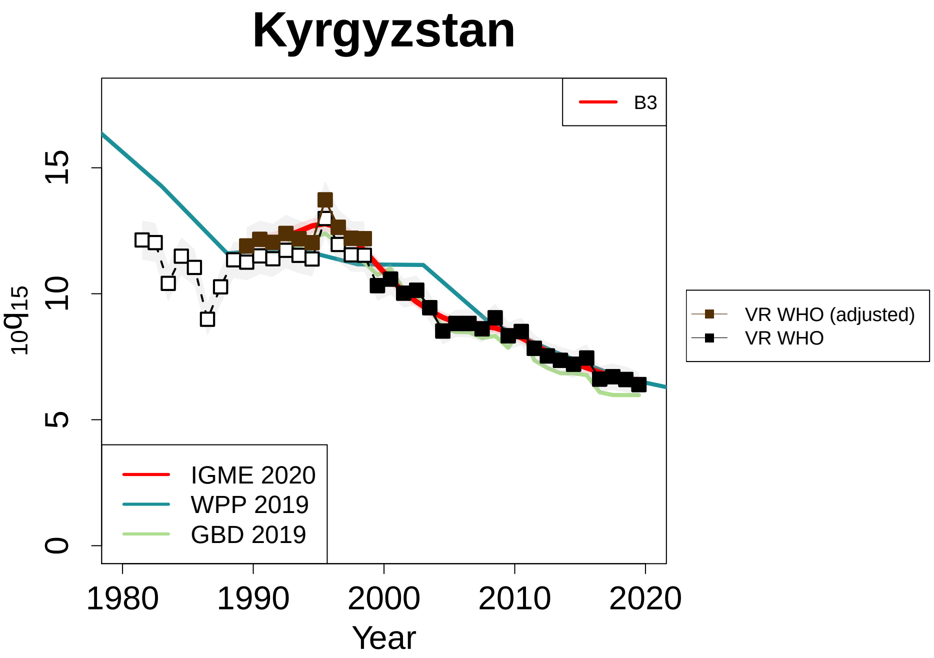
Kyrgyzstan
B3
VR WHO (adjusted)
VR WHO
IGME 2020
WPP 2019
GBD 2019
$_{10}q_{15}$
0
5
10
15
1980
1990
2000
2010
2020
Year

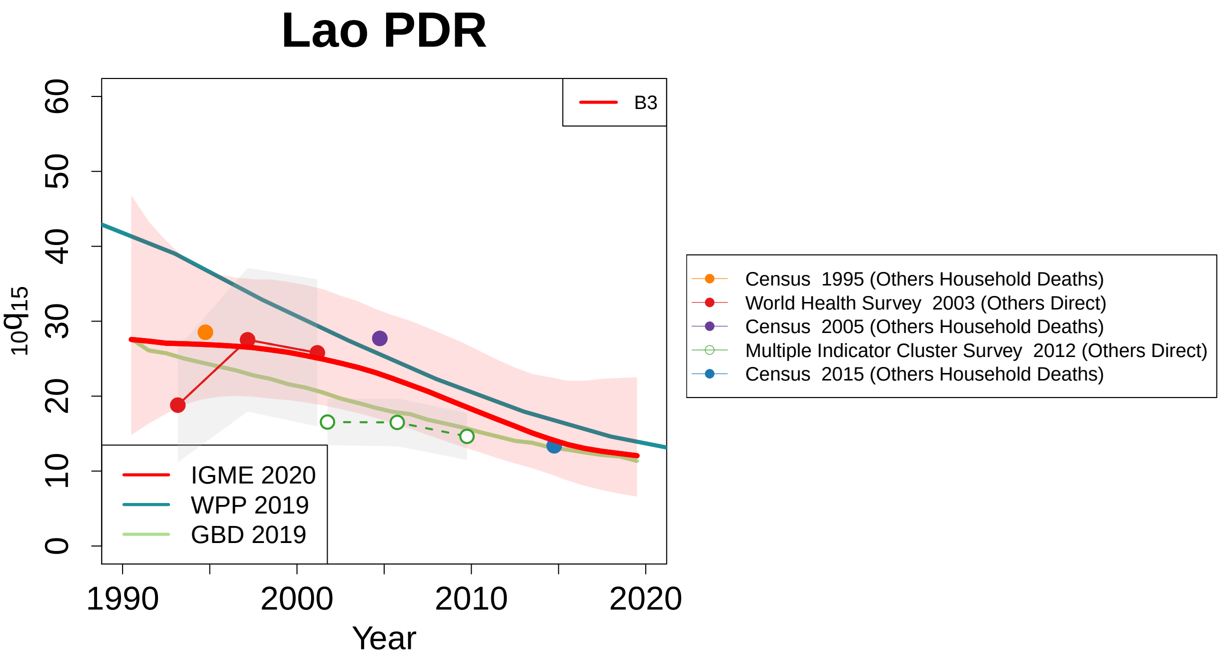
Lao PDR
B3
Census 1995 (Others Household Deaths)
World Health Survey 2003 (Others Direct)
Census 2005 (Others Household Deaths)
Multiple Indicator Cluster Survey 2012 (Others Direct)
Census 2015 (Others Household Deaths)
IGME 2020
WPP 2019
GBD 2019
$_{10}q_{15}$
0
10
20
30
40
50
60
1990
2000
2010
2020
Year

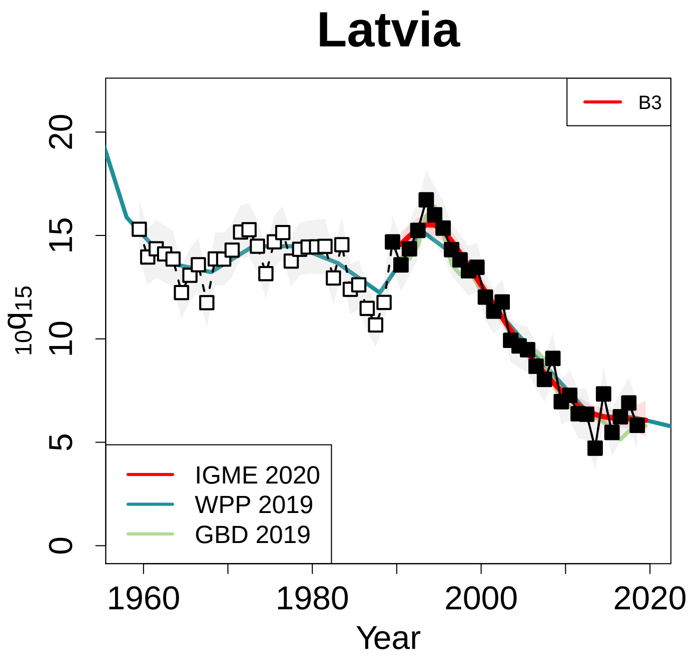
Latvia
B3
IGME 2020
WPP 2019
GBD 2019
$_{10}q_{15}$
0
5
10
15
20
1960
1980
2000
2020
Year

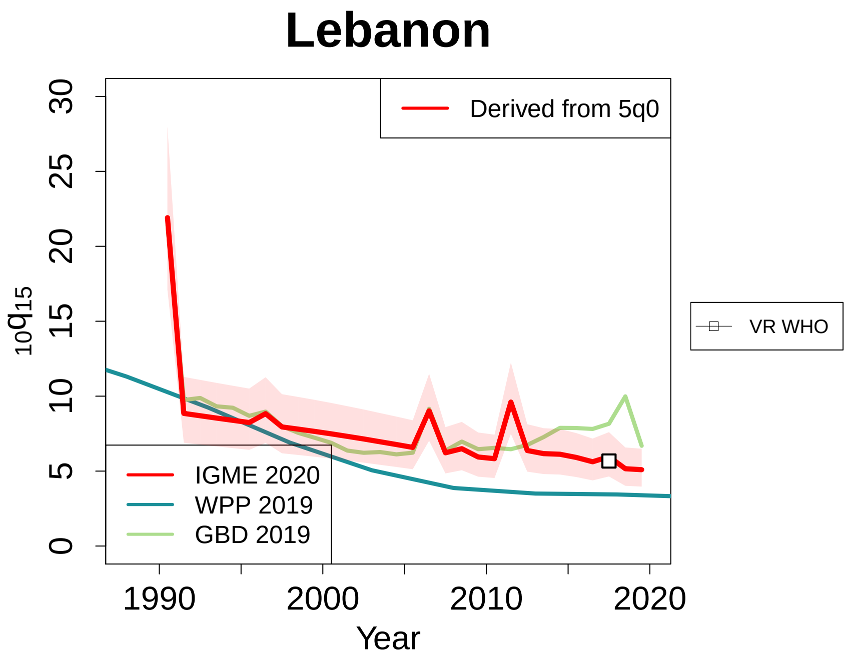
Lebanon
Derived from 5q0
VR WHO
IGME 2020
WPP 2019
GBD 2019
$_{10}q_{15}$
0
5
10
15
20
25
30
1990
2000
2010
2020
Year

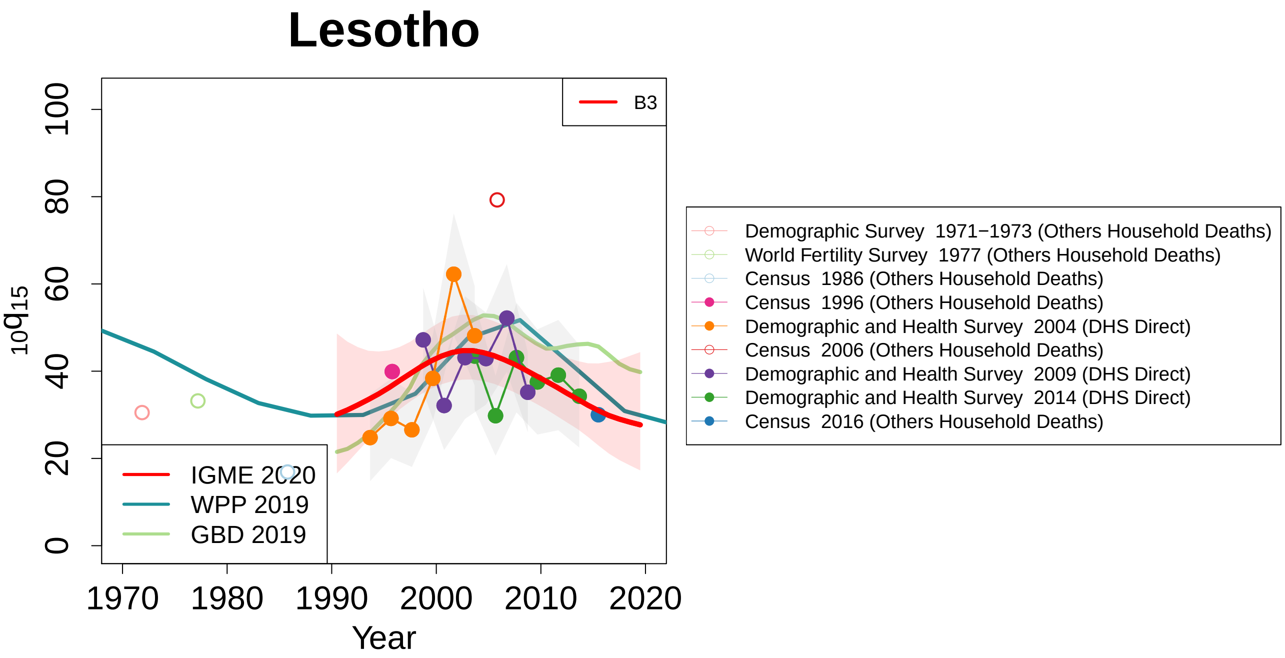
Lesotho
B3
IGME 2020
WPP 2019
GBD 2019
Year
Demographic Survey 1971−1973 (Others Household Deaths)
World Fertility Survey 1977 (Others Household Deaths)
Census 1986 (Others Household Deaths)
Census 1996 (Others Household Deaths)
Demographic and Health Survey 2004 (DHS Direct)
Census 2006 (Others Household Deaths)
Demographic and Health Survey 2009 (DHS Direct)
Demographic and Health Survey 2014 (DHS Direct)
Census 2016 (Others Household Deaths)

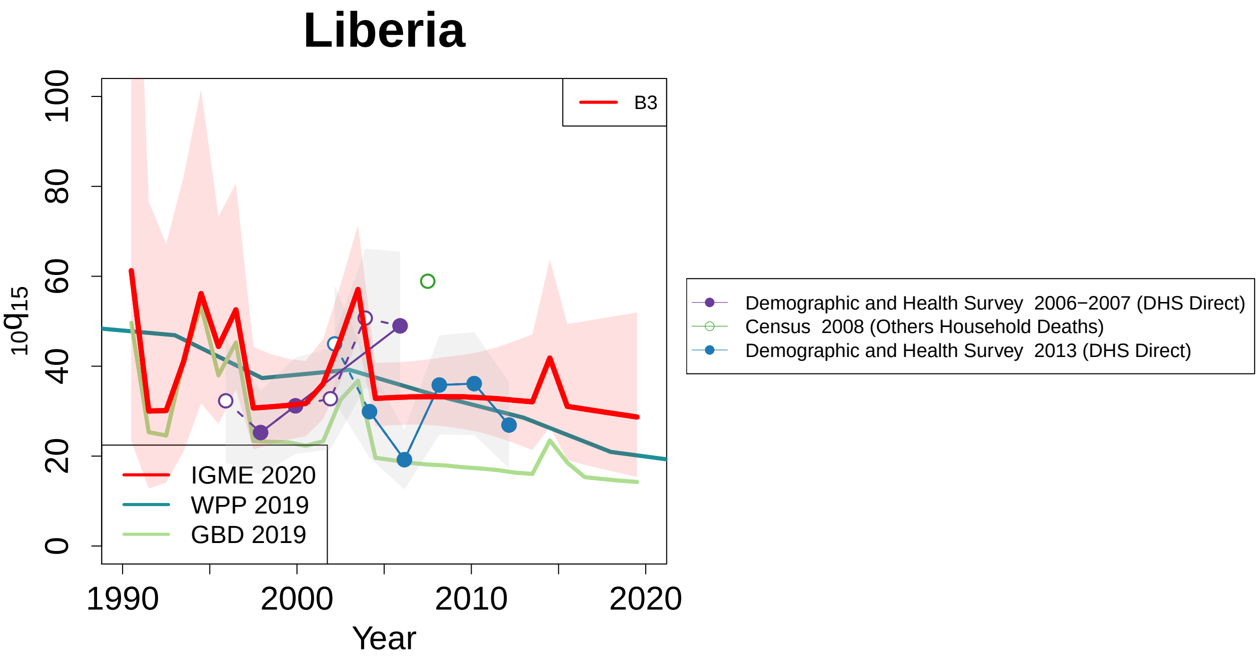
Liberia
B3
IGME 2020
WPP 2019
GBD 2019
Year
Demographic and Health Survey 2006−2007 (DHS Direct)
Census 2008 (Others Household Deaths)
Demographic and Health Survey 2013 (DHS Direct)

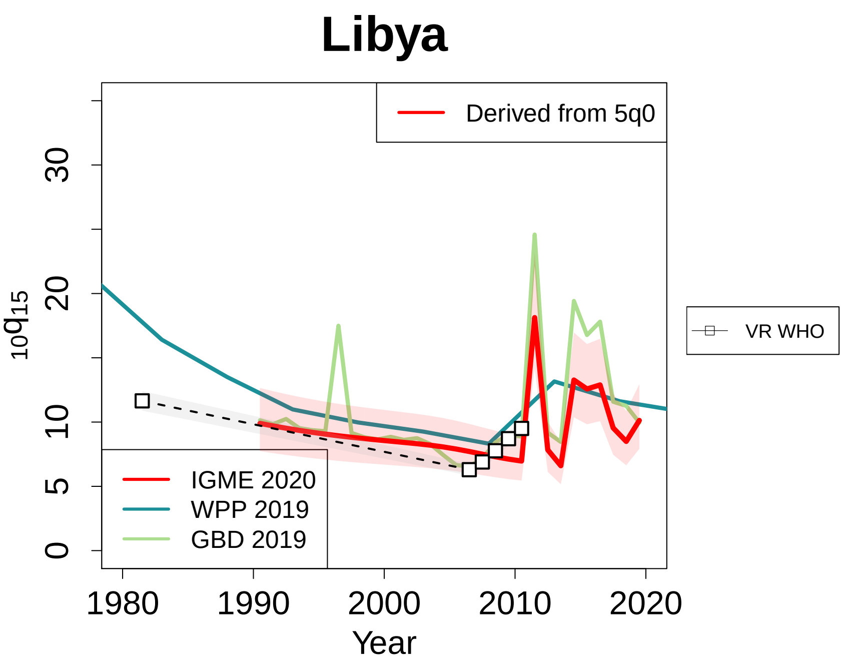
Libya
Derived from 5q0
IGME 2020
WPP 2019
GBD 2019
VR WHO
$_{10}q_{15}$
0
5
10
20
30
1980
1990
2000
2010
2020
Year

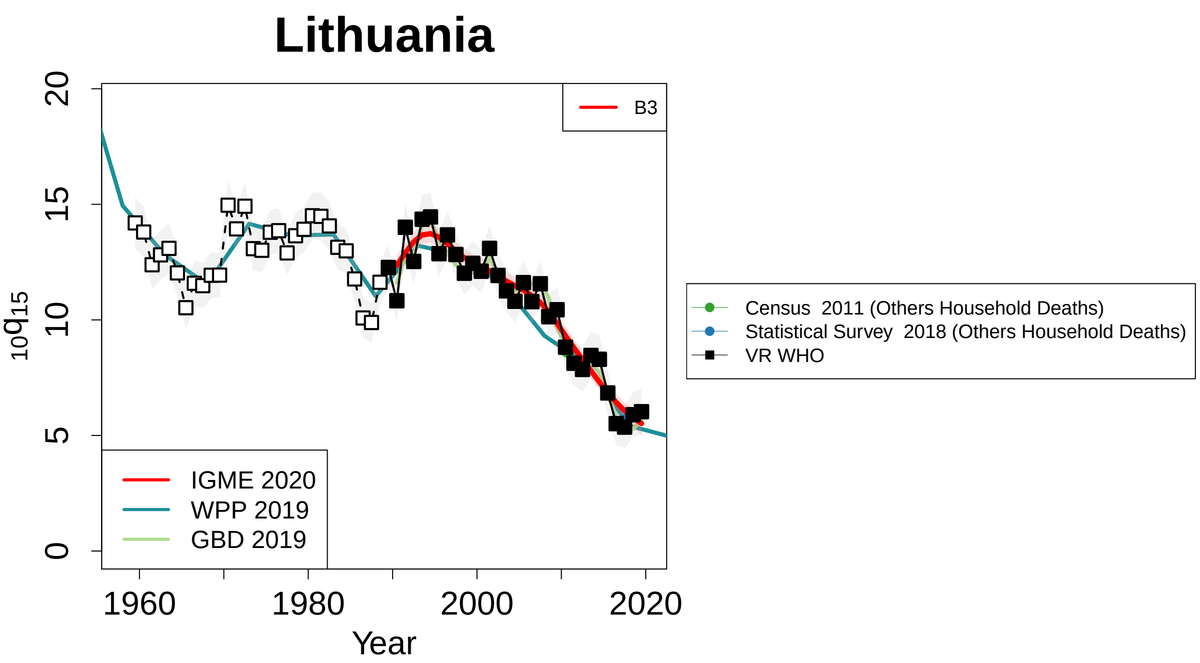
Lithuania
B3
IGME 2020
WPP 2019
GBD 2019
Census 2011 (Others Household Deaths)
Statistical Survey 2018 (Others Household Deaths)
VR WHO
$_{10}q_{15}$
0
5
10
15
20
1960
1980
2000
2020
Year

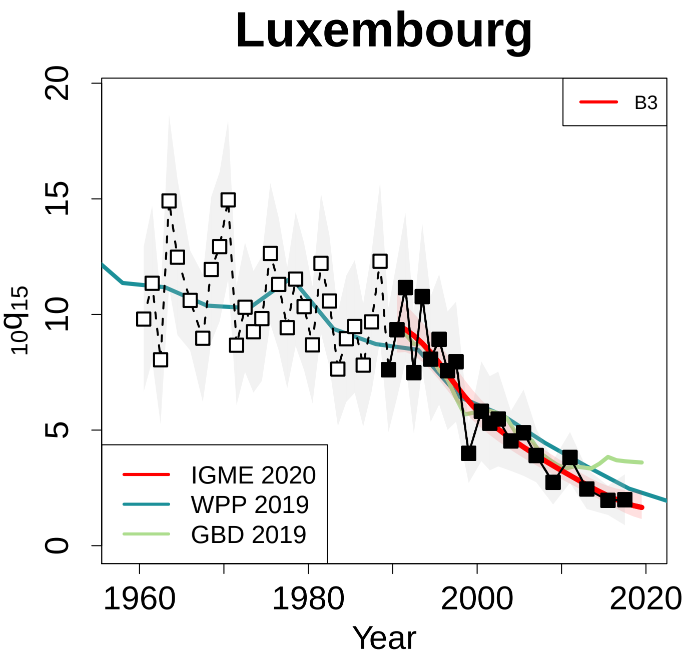
Luxembourg
B3
IGME 2020
WPP 2019
GBD 2019
$_{10}q_{15}$
0
5
10
15
20
1960
1980
2000
2020
Year

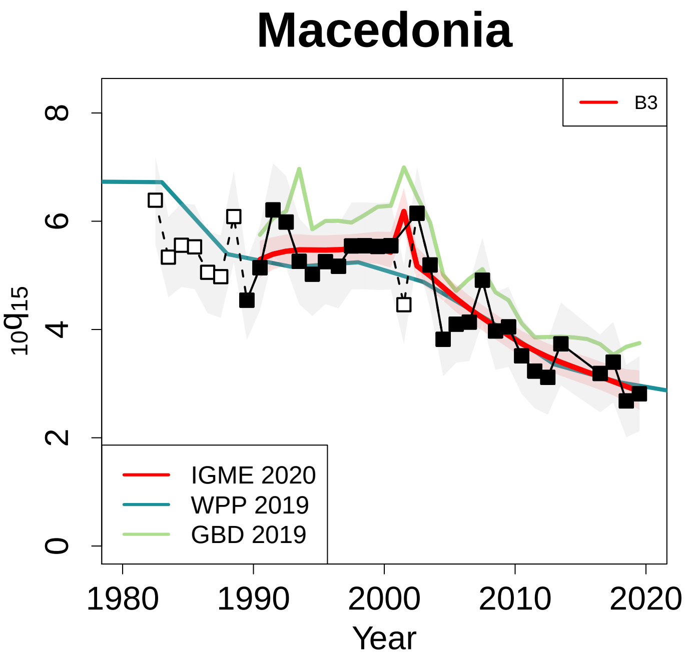
Macedonia
B3
IGME 2020
WPP 2019
GBD 2019
$_{10}q_{15}$
0
2
4
6
8
1980
1990
2000
2010
2020
Year

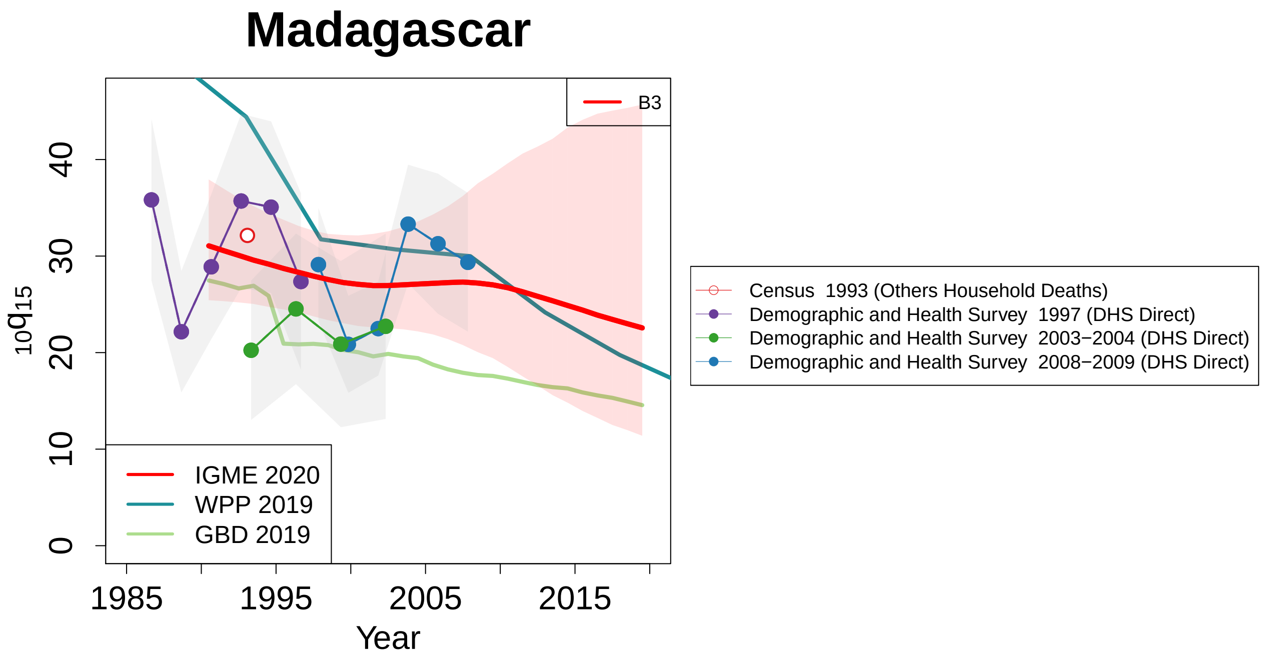
Madagascar
B3
40
30
20
10
0
$_{10}q_{15}$
IGME 2020
WPP 2019
GBD 2019
1985
1995
2005
2015
Year
Census 1993 (Others Household Deaths)
Demographic and Health Survey 1997 (DHS Direct)
Demographic and Health Survey 2003−2004 (DHS Direct)
Demographic and Health Survey 2008−2009 (DHS Direct)

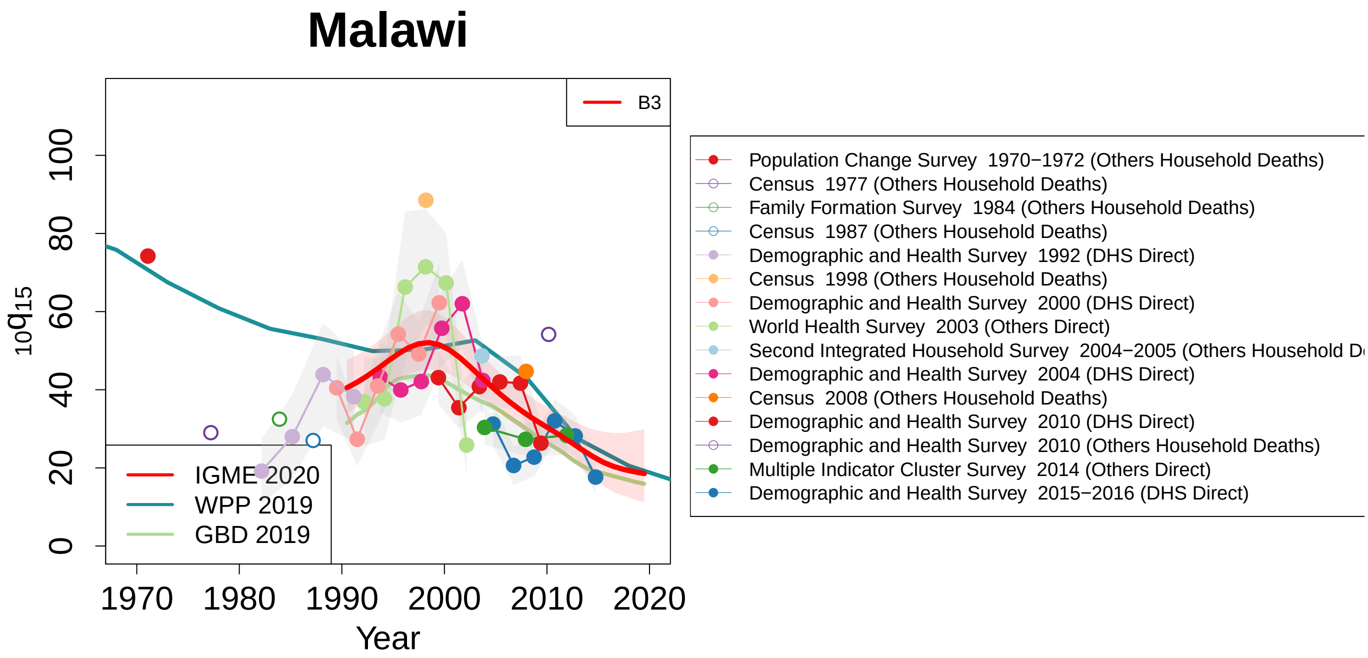
Malawi
B3
100
80
60
40
20
0
$_{10}q_{15}$
IGME 2020
WPP 2019
GBD 2019
1970
1980
1990
2000
2010
2020
Year
Population Change Survey 1970−1972 (Others Household Deaths)
Census 1977 (Others Household Deaths)
Family Formation Survey 1984 (Others Household Deaths)
Census 1987 (Others Household Deaths)
Demographic and Health Survey 1992 (DHS Direct)
Census 1998 (Others Household Deaths)
Demographic and Health Survey 2000 (DHS Direct)
World Health Survey 2003 (Others Direct)
Second Integrated Household Survey 2004−2005 (Others Household D
Demographic and Health Survey 2004 (DHS Direct)
Census 2008 (Others Household Deaths)
Demographic and Health Survey 2010 (DHS Direct)
Demographic and Health Survey 2010 (Others Household Deaths)
Multiple Indicator Cluster Survey 2014 (Others Direct)
Demographic and Health Survey 2015−2016 (DHS Direct)

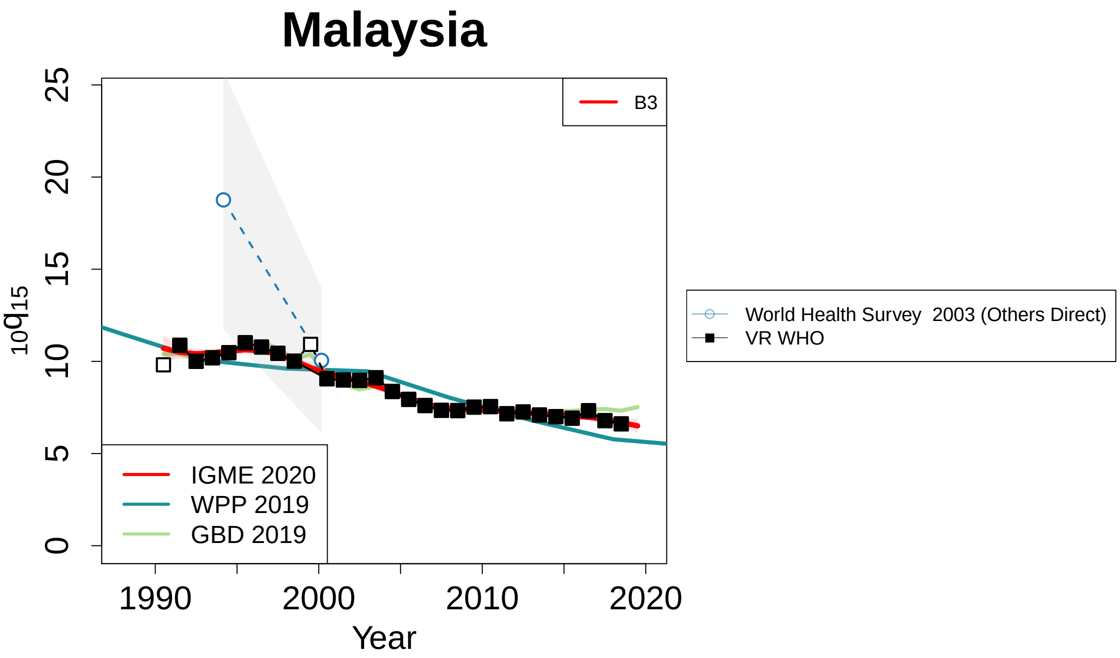
Malaysia
B3
World Health Survey 2003 (Others Direct)
VR WHO
IGME 2020
WPP 2019
GBD 2019
$_{10}q_{15}$
0
5
10
15
20
25
1990
2000
2010
2020
Year

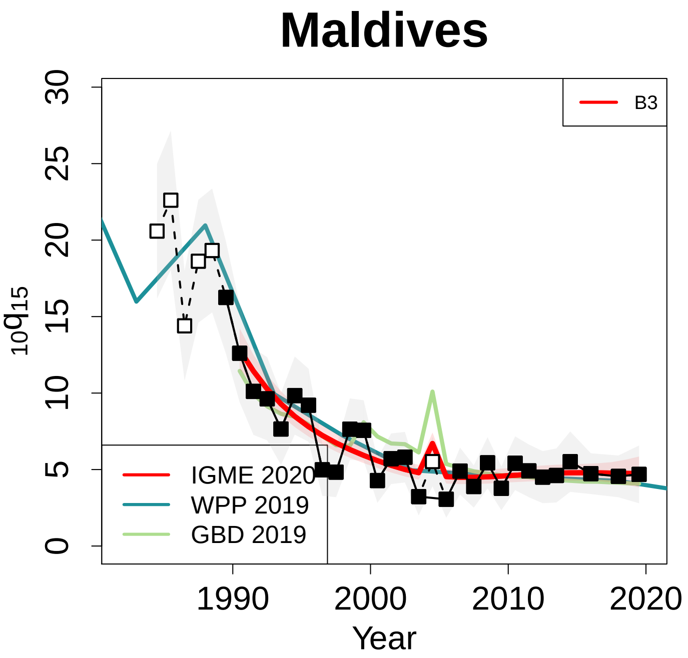
Maldives
B3
IGME 2020
WPP 2019
GBD 2019
$_{10}q_{15}$
0
5
10
15
20
25
30
1990
2000
2010
2020
Year

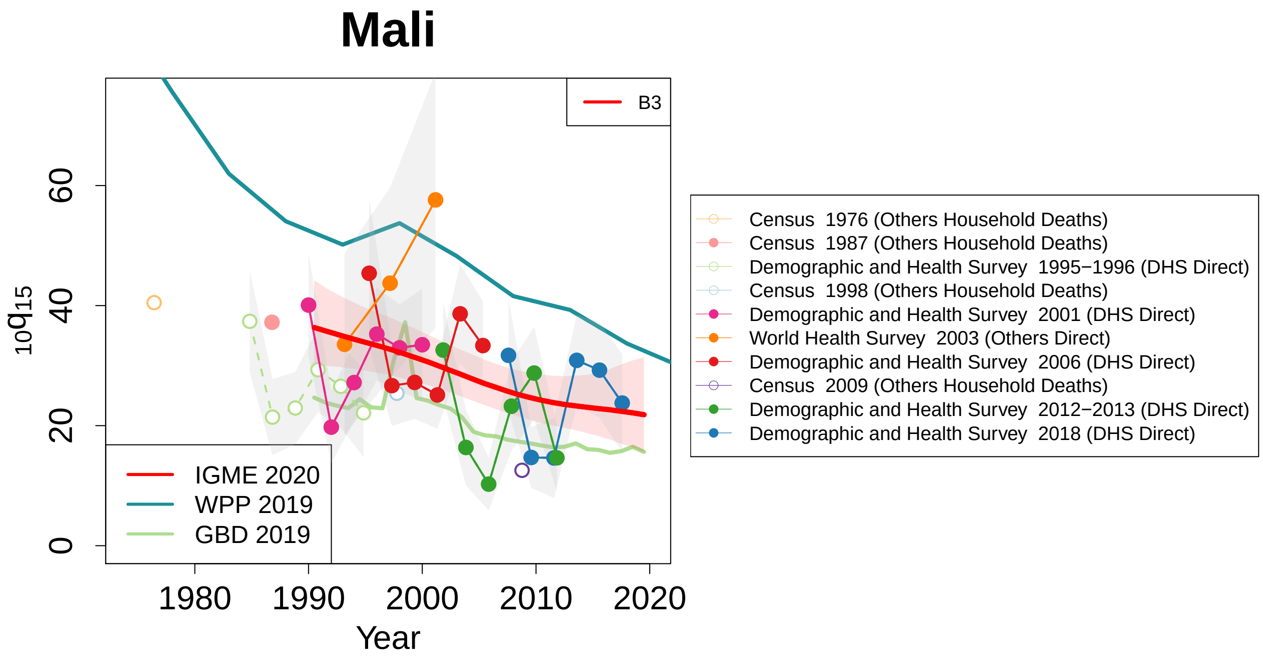
Mali
B3
IGME 2020
WPP 2019
GBD 2019
$_{10}q_{15}$
Year
Census 1976 (Others Household Deaths)
Census 1987 (Others Household Deaths)
Demographic and Health Survey 1995−1996 (DHS Direct)
Census 1998 (Others Household Deaths)
Demographic and Health Survey 2001 (DHS Direct)
World Health Survey 2003 (Others Direct)
Demographic and Health Survey 2006 (DHS Direct)
Census 2009 (Others Household Deaths)
Demographic and Health Survey 2012−2013 (DHS Direct)
Demographic and Health Survey 2018 (DHS Direct)

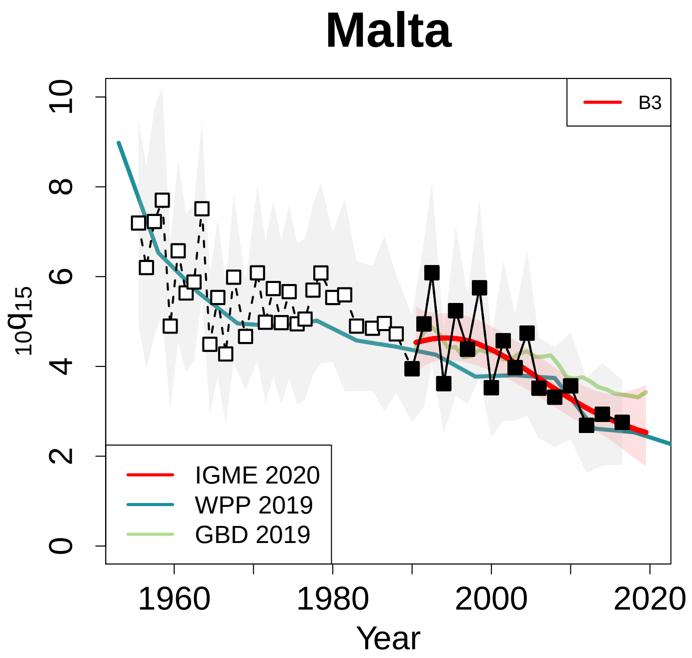
Malta
B3
IGME 2020
WPP 2019
GBD 2019
$_{10}q_{15}$
Year

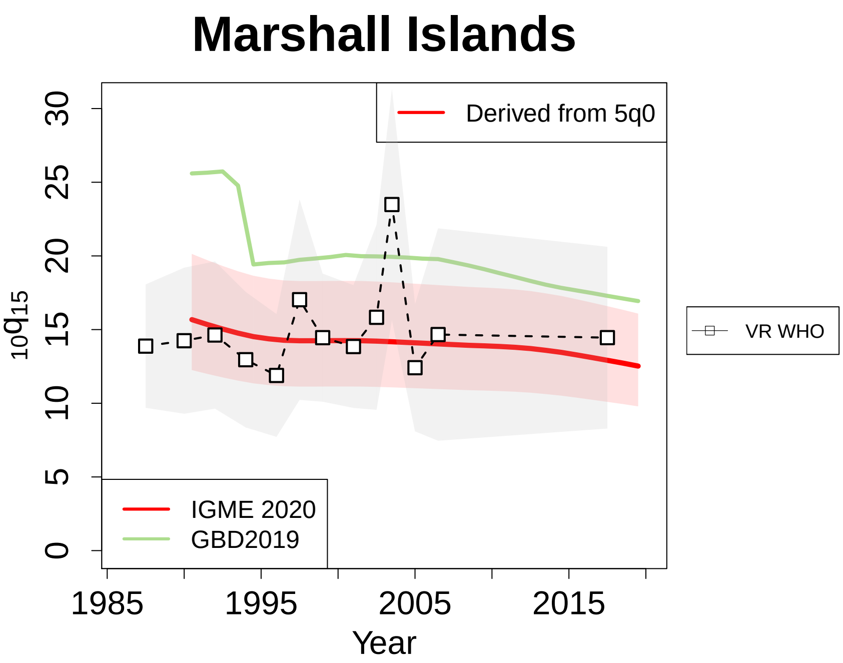
Marshall Islands
Derived from 5q0
VR WHO
IGME 2020
GBD2019
$_{10}q_{15}$
0
5
10
15
20
25
30
1985
1995
2005
2015
Year

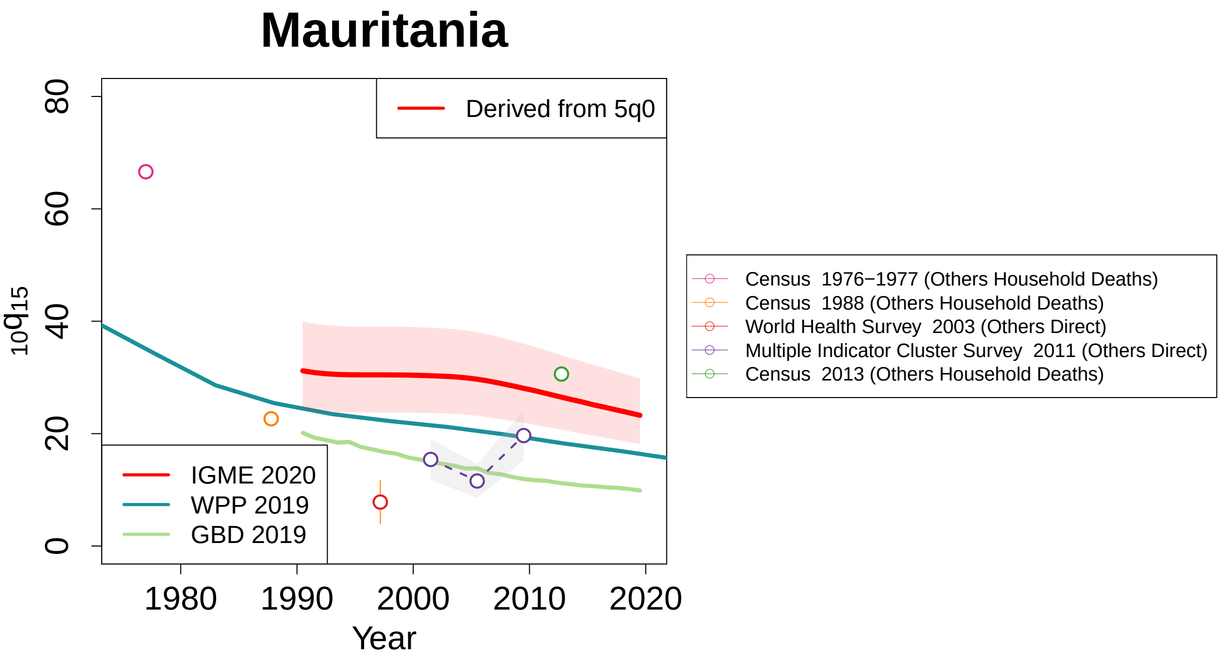
Mauritania
Derived from 5q0
Census 1976−1977 (Others Household Deaths)
Census 1988 (Others Household Deaths)
World Health Survey 2003 (Others Direct)
Multiple Indicator Cluster Survey 2011 (Others Direct)
Census 2013 (Others Household Deaths)
IGME 2020
WPP 2019
GBD 2019
$_{10}q_{15}$
0
20
40
60
80
1980
1990
2000
2010
2020
Year

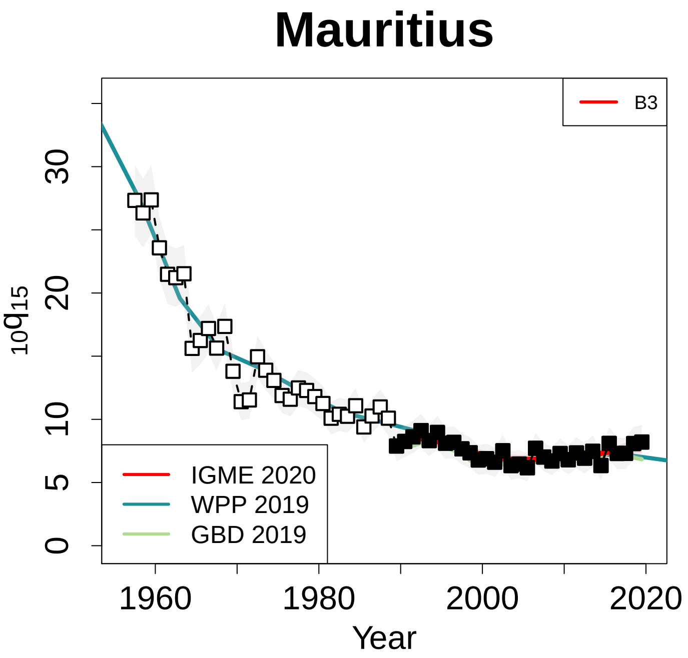
Mauritius
B3
IGME 2020
WPP 2019
GBD 2019
$_{10}q_{15}$
Year

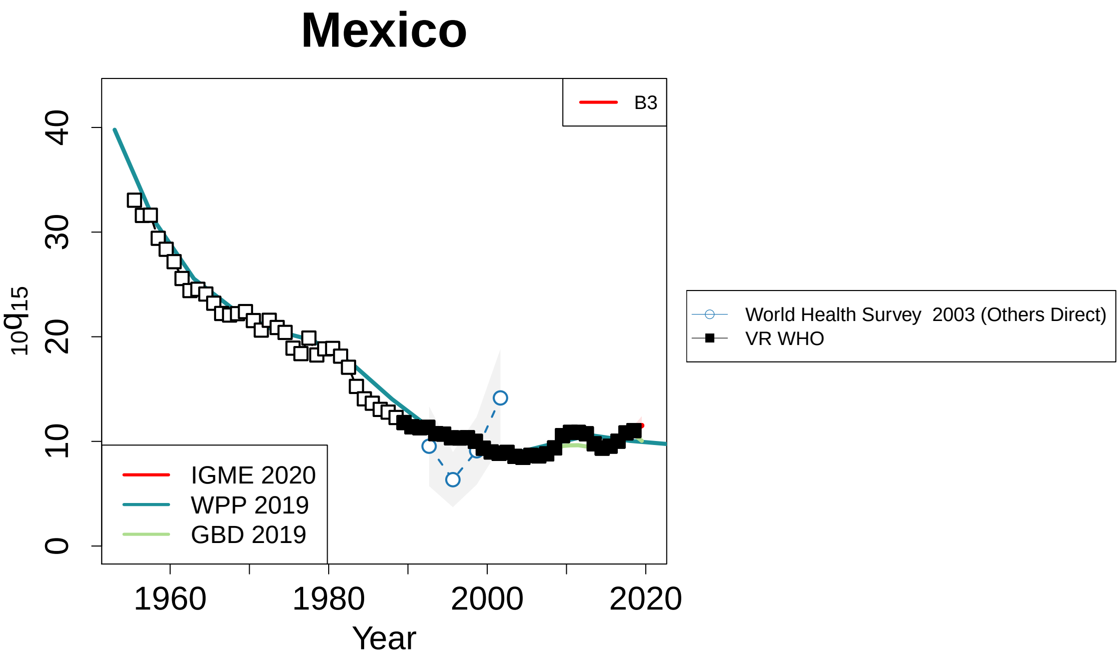
Mexico
B3
IGME 2020
WPP 2019
GBD 2019
$_{10}q_{15}$
Year
World Health Survey 2003 (Others Direct)
VR WHO

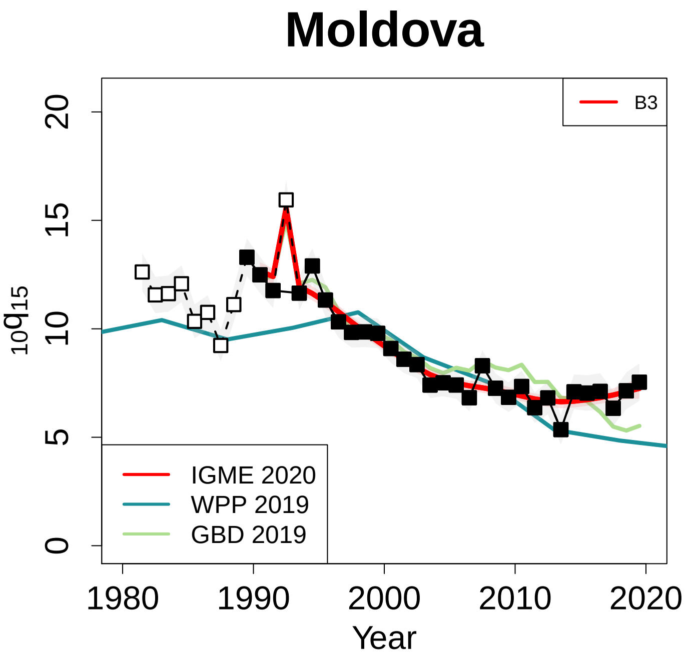
Moldova
B3
IGME 2020
WPP 2019
GBD 2019
0
5
10
15
20
$_{10}q_{15}$
1980
1990
2000
2010
2020
Year

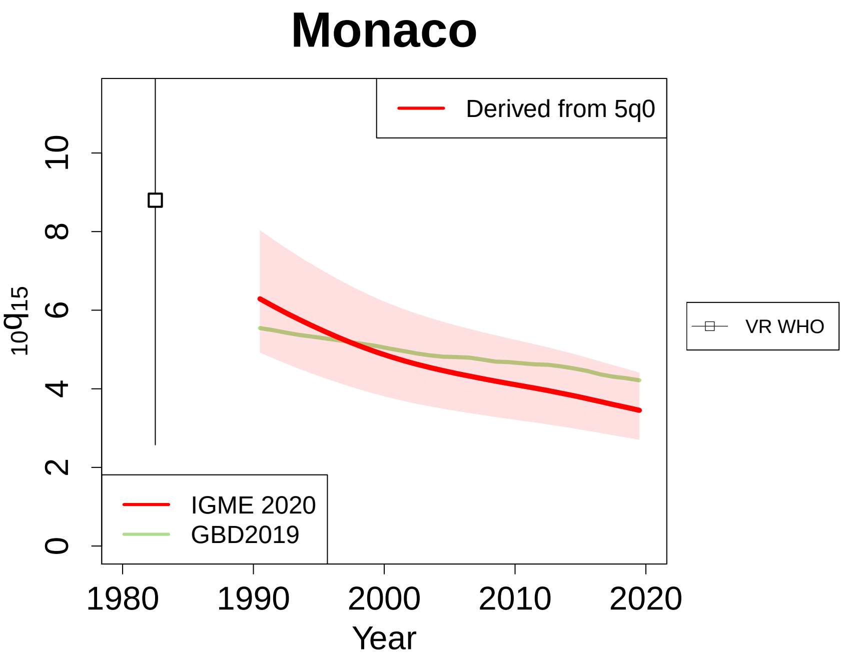
Monaco
Derived from 5q0
VR WHO
IGME 2020
GBD2019
0
2
4
6
8
10
$_{10}q_{15}$
1980
1990
2000
2010
2020
Year

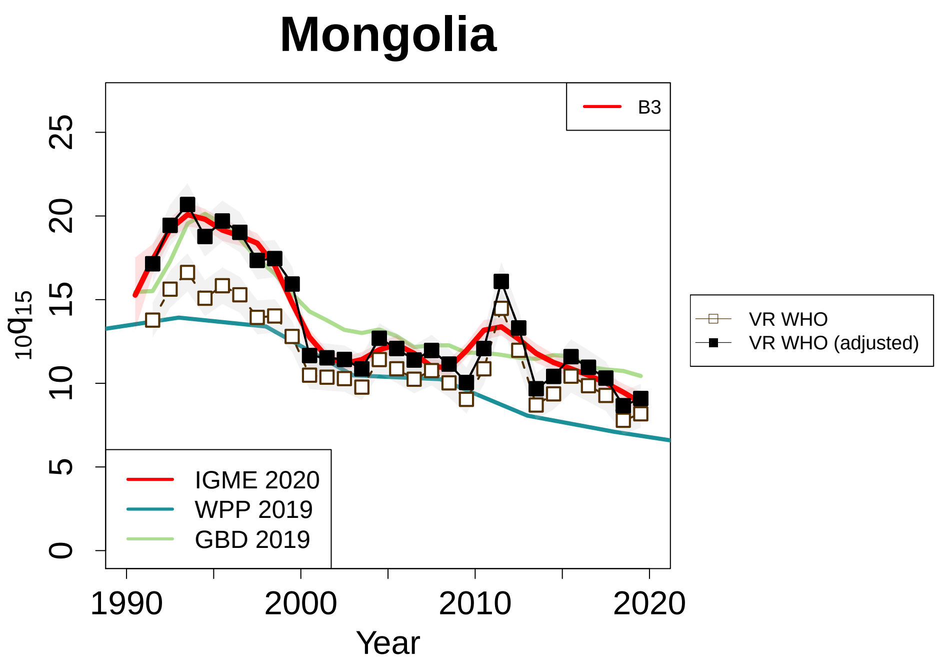
Mongolia
B3
IGME 2020
WPP 2019
GBD 2019
VR WHO
VR WHO (adjusted)
1990
2000
2010
2020
Year
0
5
10
15
20
25
$_{10}q_{15}$

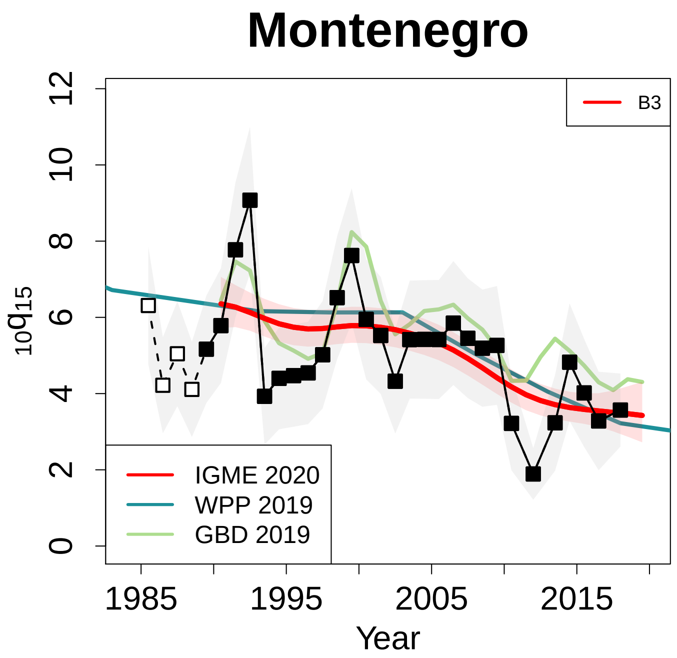
Montenegro
B3
IGME 2020
WPP 2019
GBD 2019
1985
1995
2005
2015
Year
0
2
4
6
8
10
12
$_{10}q_{15}$

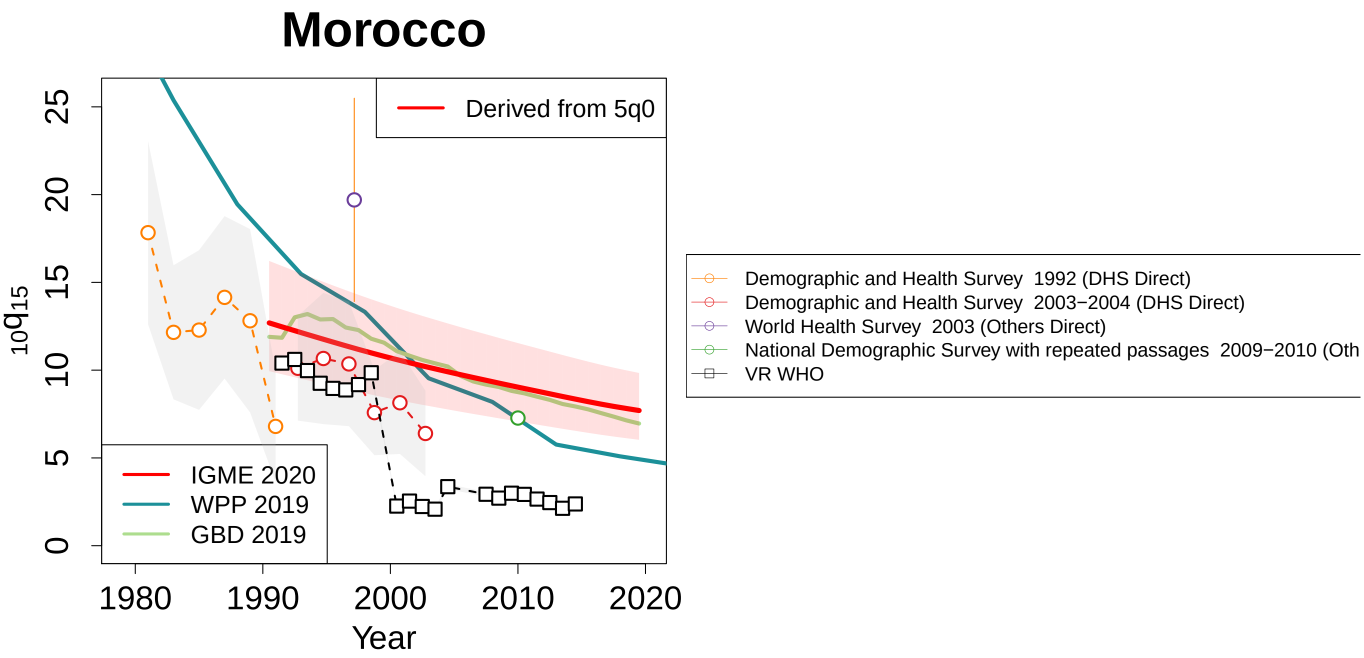
Morocco
Derived from 5q0
IGME 2020
WPP 2019
GBD 2019
10q15
0
5
10
15
20
25
1980
1990
2000
2010
2020
Year
Demographic and Health Survey 1992 (DHS Direct)
Demographic and Health Survey 2003−2004 (DHS Direct)
World Health Survey 2003 (Others Direct)
National Demographic Survey with repeated passages 2009−2010 (Oth
VR WHO

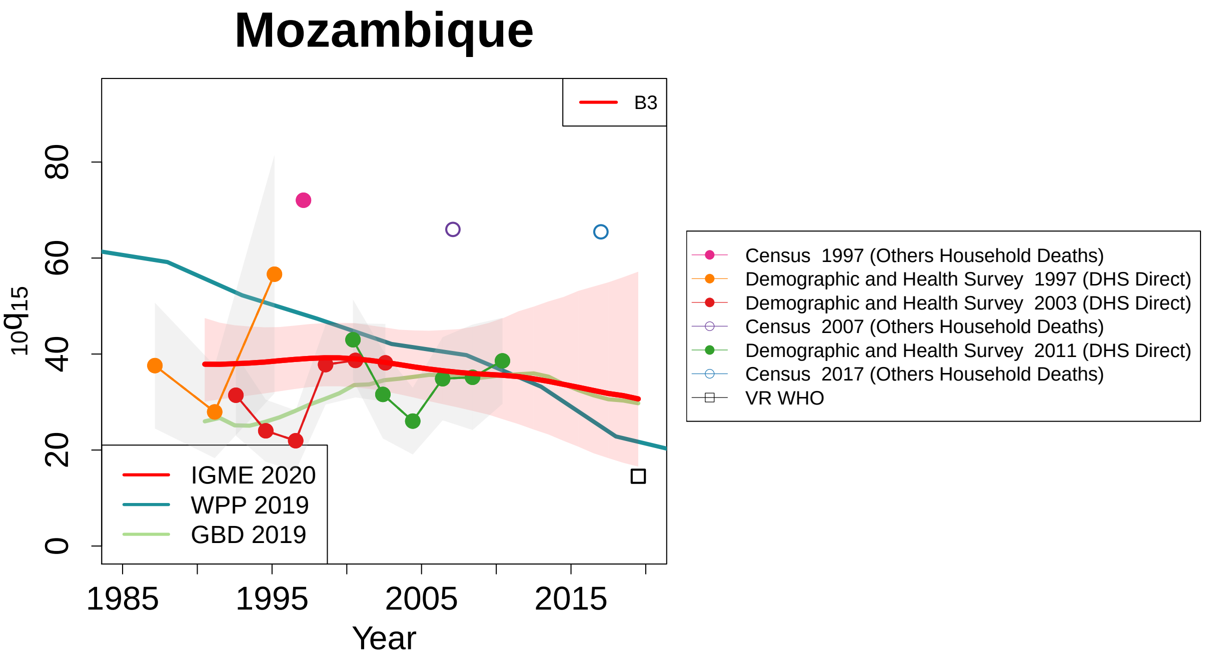
Mozambique
B3
IGME 2020
WPP 2019
GBD 2019
10q15
0
20
40
60
80
1985
1995
2005
2015
Year
Census 1997 (Others Household Deaths)
Demographic and Health Survey 1997 (DHS Direct)
Demographic and Health Survey 2003 (DHS Direct)
Census 2007 (Others Household Deaths)
Demographic and Health Survey 2011 (DHS Direct)
Census 2017 (Others Household Deaths)
VR WHO

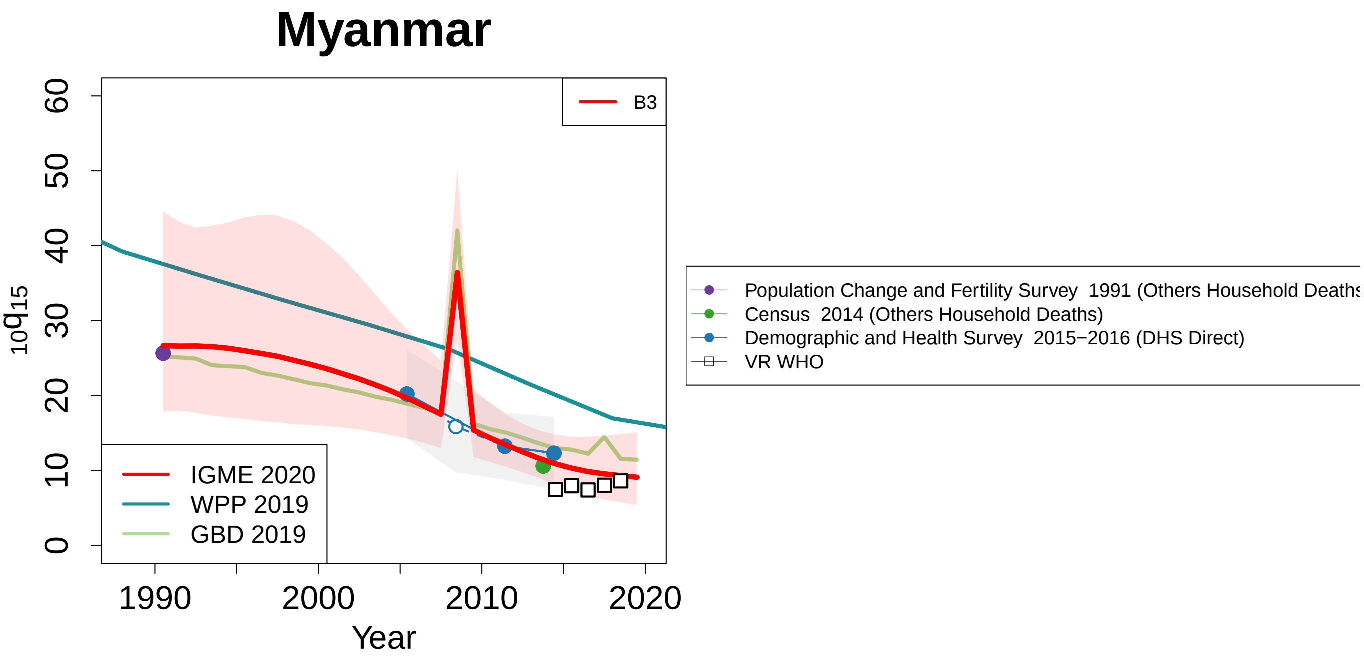

Myanmar
B3
IGME 2020
WPP 2019
GBD 2019
Year
$_{10}q_{15}$
Population Change and Fertility Survey 1991 (Others Household Deaths
Census 2014 (Others Household Deaths)
Demographic and Health Survey 2015−2016 (DHS Direct)
VR WHO

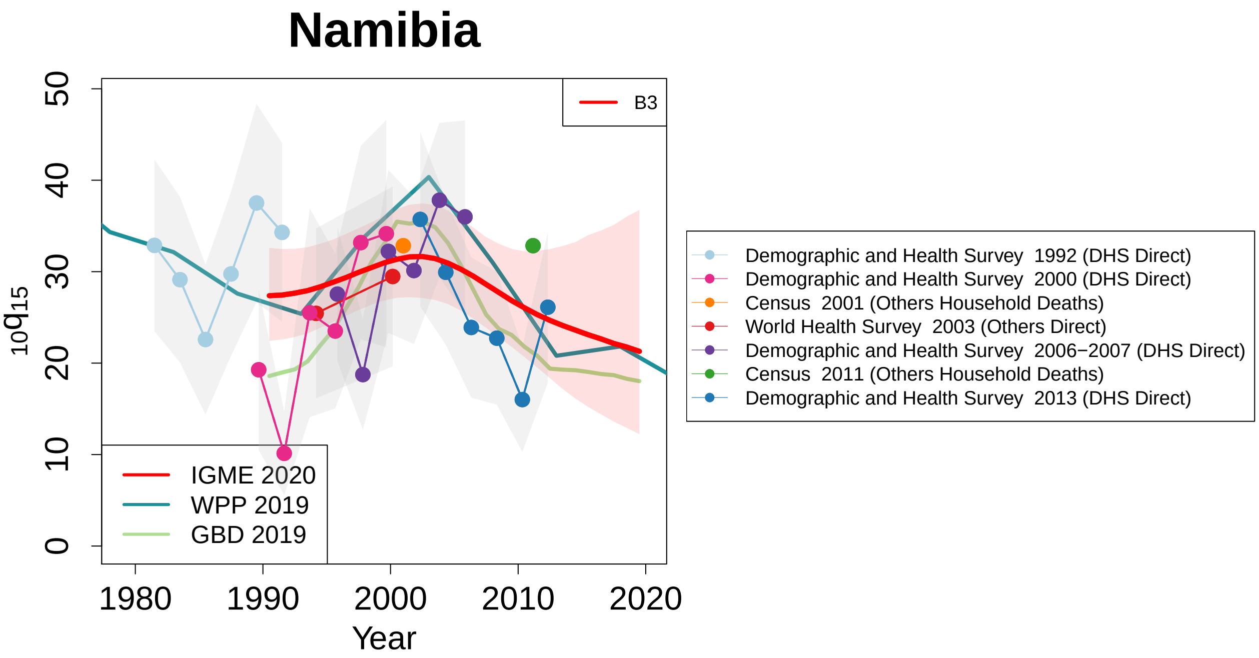

Namibia
B3
IGME 2020
WPP 2019
GBD 2019
Year
$_{10}q_{15}$
Demographic and Health Survey 1992 (DHS Direct)
Demographic and Health Survey 2000 (DHS Direct)
Census 2001 (Others Household Deaths)
World Health Survey 2003 (Others Direct)
Demographic and Health Survey 2006−2007 (DHS Direct)
Census 2011 (Others Household Deaths)
Demographic and Health Survey 2013 (DHS Direct)

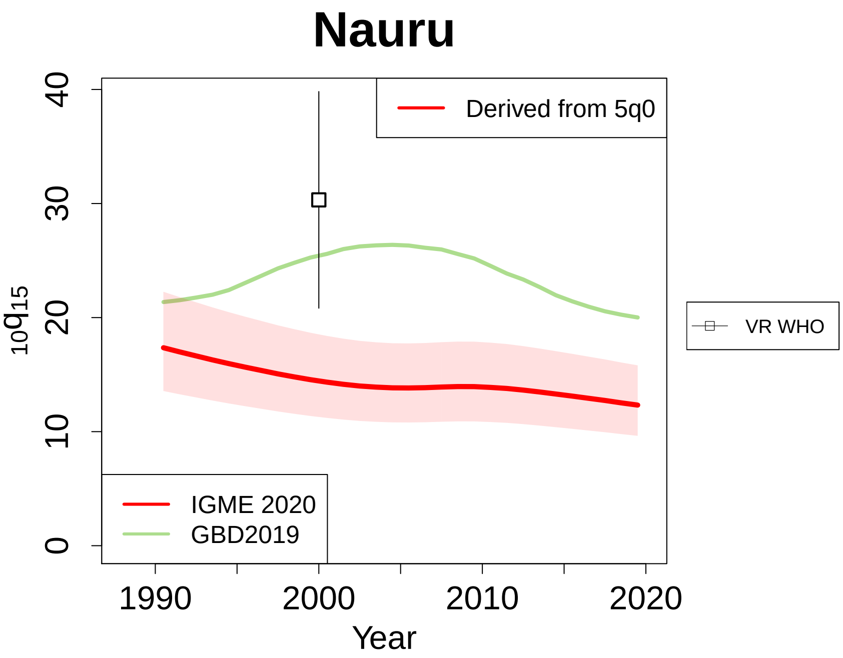
Nauru
Derived from 5q0
VR WHO
IGME 2020
GBD2019
$_{10}q_{15}$
0
10
20
30
40
1990
2000
2010
2020
Year

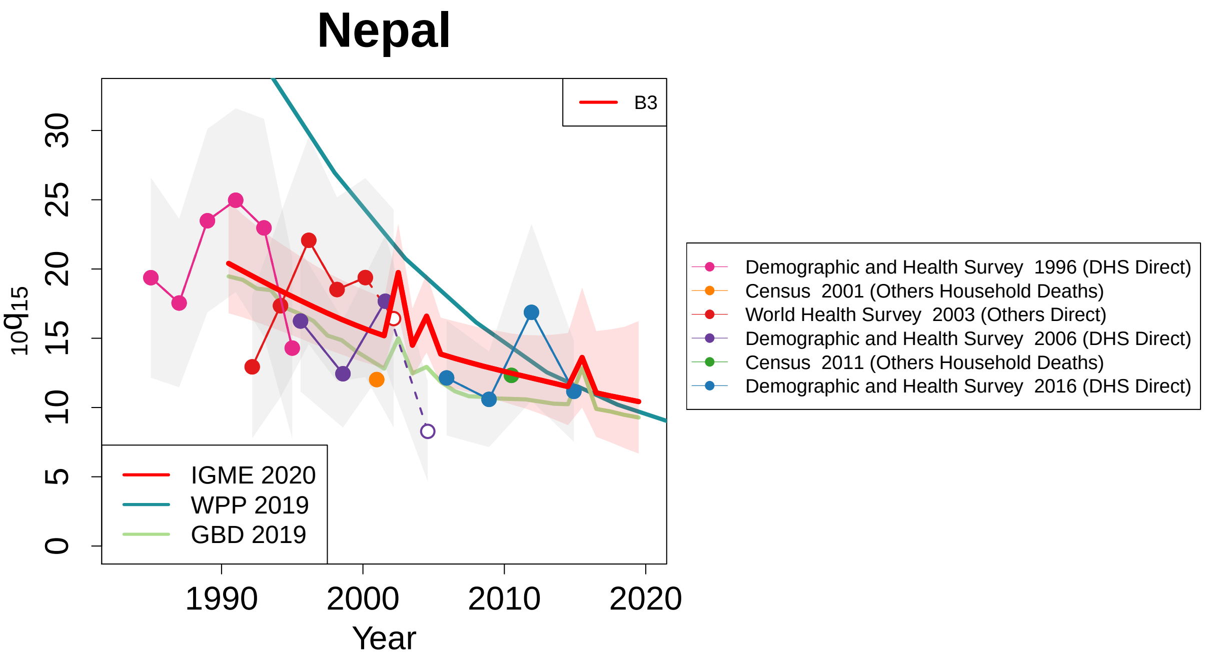
Nepal
B3
Demographic and Health Survey 1996 (DHS Direct)
Census 2001 (Others Household Deaths)
World Health Survey 2003 (Others Direct)
Demographic and Health Survey 2006 (DHS Direct)
Census 2011 (Others Household Deaths)
Demographic and Health Survey 2016 (DHS Direct)
IGME 2020
WPP 2019
GBD 2019
$_{10}q_{15}$
0
5
10
15
20
25
30
1990
2000
2010
2020
Year

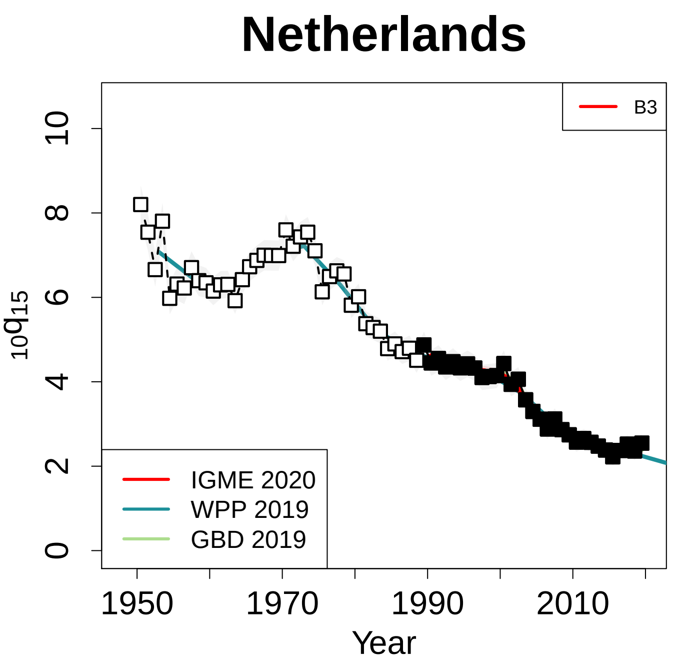
Netherlands
B3
IGME 2020
WPP 2019
GBD 2019
10q15
0
2
4
6
8
10
1950
1970
1990
2010
Year

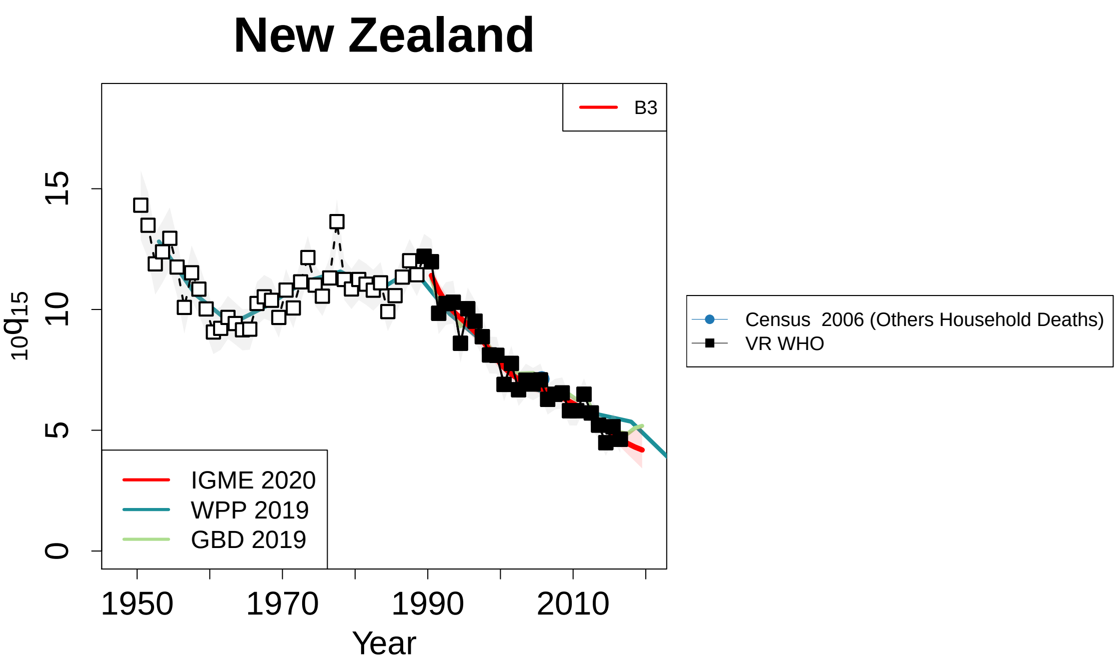
New Zealand
B3
IGME 2020
WPP 2019
GBD 2019
10q15
0
5
10
15
1950
1970
1990
2010
Year
Census 2006 (Others Household Deaths)
VR WHO

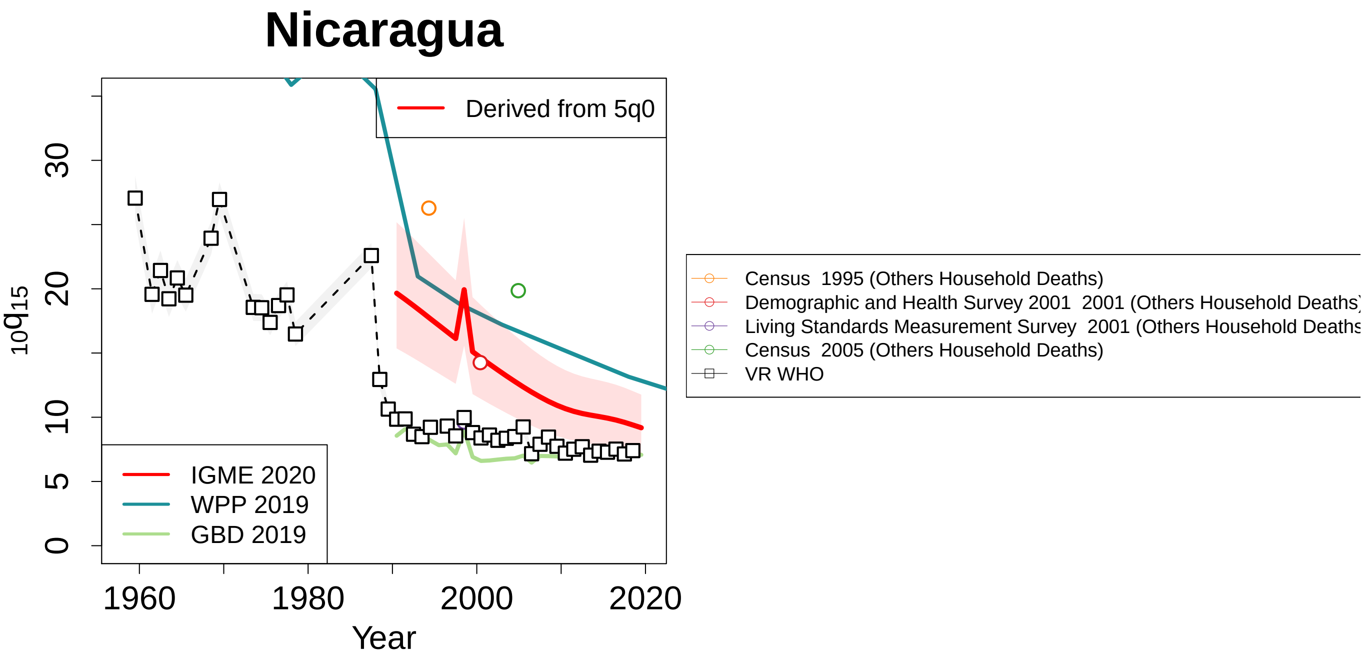
Nicaragua
Derived from 5q0
Census 1995 (Others Household Deaths)
Demographic and Health Survey 2001 2001 (Others Household Deaths)
Living Standards Measurement Survey 2001 (Others Household Deaths
Census 2005 (Others Household Deaths)
VR WHO
IGME 2020
WPP 2019
GBD 2019
$_{10}q_{15}$
Year

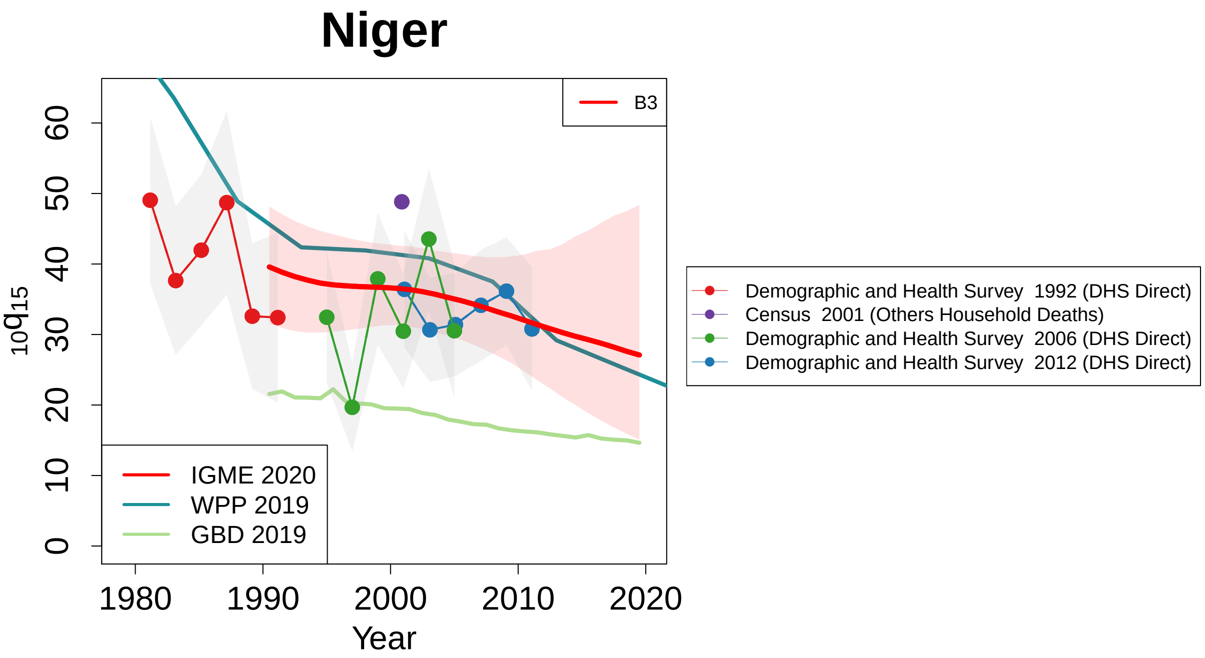
Niger
B3
Demographic and Health Survey 1992 (DHS Direct)
Census 2001 (Others Household Deaths)
Demographic and Health Survey 2006 (DHS Direct)
Demographic and Health Survey 2012 (DHS Direct)
IGME 2020
WPP 2019
GBD 2019
$_{10}q_{15}$
Year

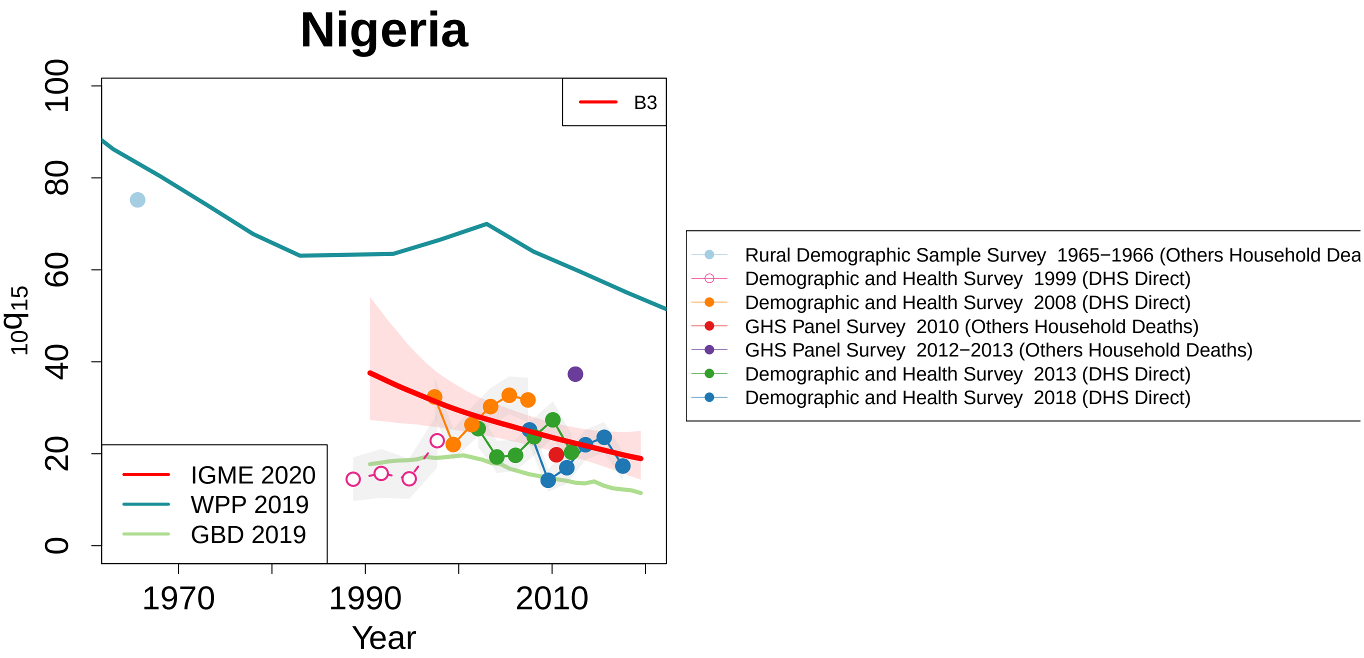
Nigeria
B3
IGME 2020
WPP 2019
GBD 2019
$_{10}q_{15}$
Year
Rural Demographic Sample Survey 1965−1966 (Others Household Dea
Demographic and Health Survey 1999 (DHS Direct)
Demographic and Health Survey 2008 (DHS Direct)
GHS Panel Survey 2010 (Others Household Deaths)
GHS Panel Survey 2012−2013 (Others Household Deaths)
Demographic and Health Survey 2013 (DHS Direct)
Demographic and Health Survey 2018 (DHS Direct)

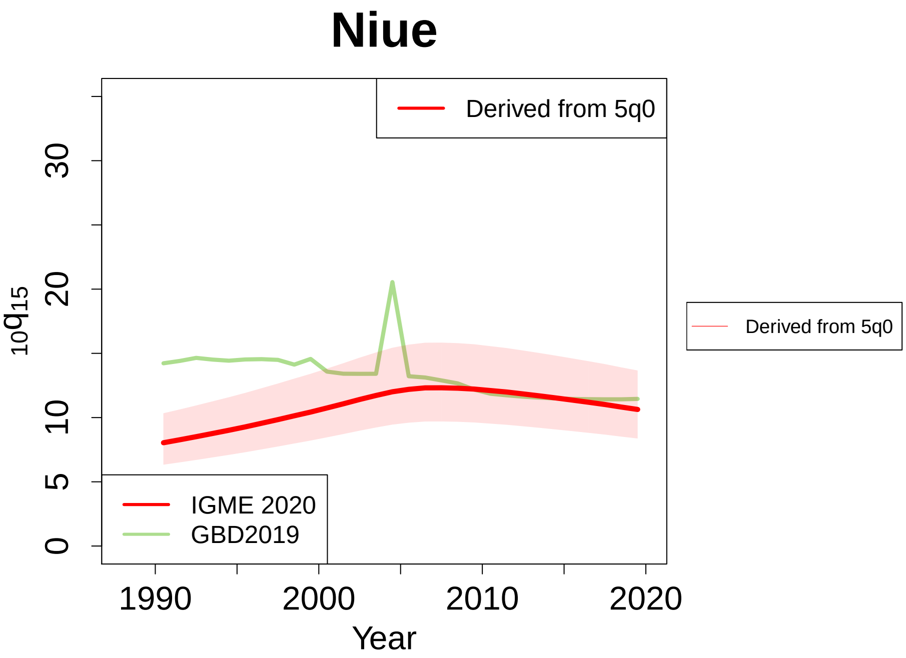
Niue
Derived from 5q0
IGME 2020
GBD2019
$_{10}q_{15}$
Year
Derived from 5q0

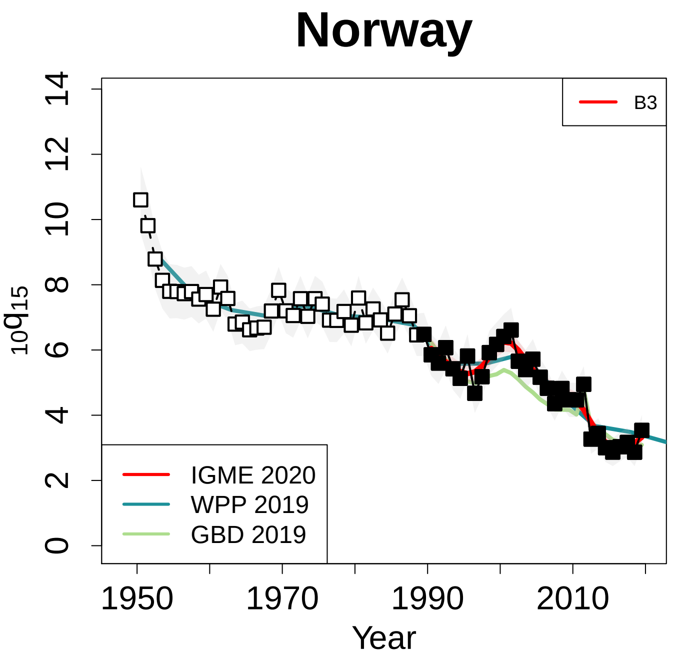
Norway
B3
IGME 2020
WPP 2019
GBD 2019
Year
10q15

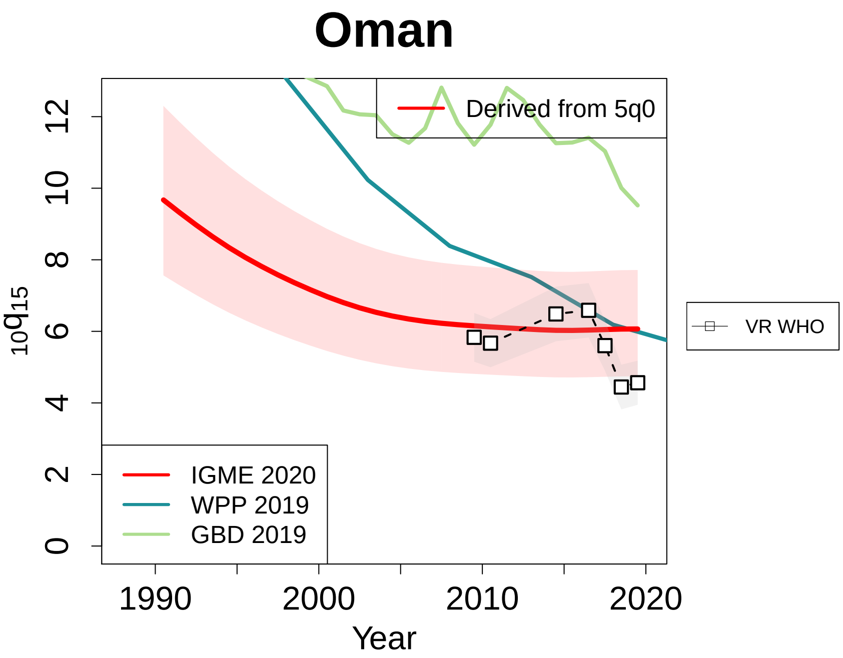
Oman
Derived from 5q0
VR WHO
IGME 2020
WPP 2019
GBD 2019
Year
10q15

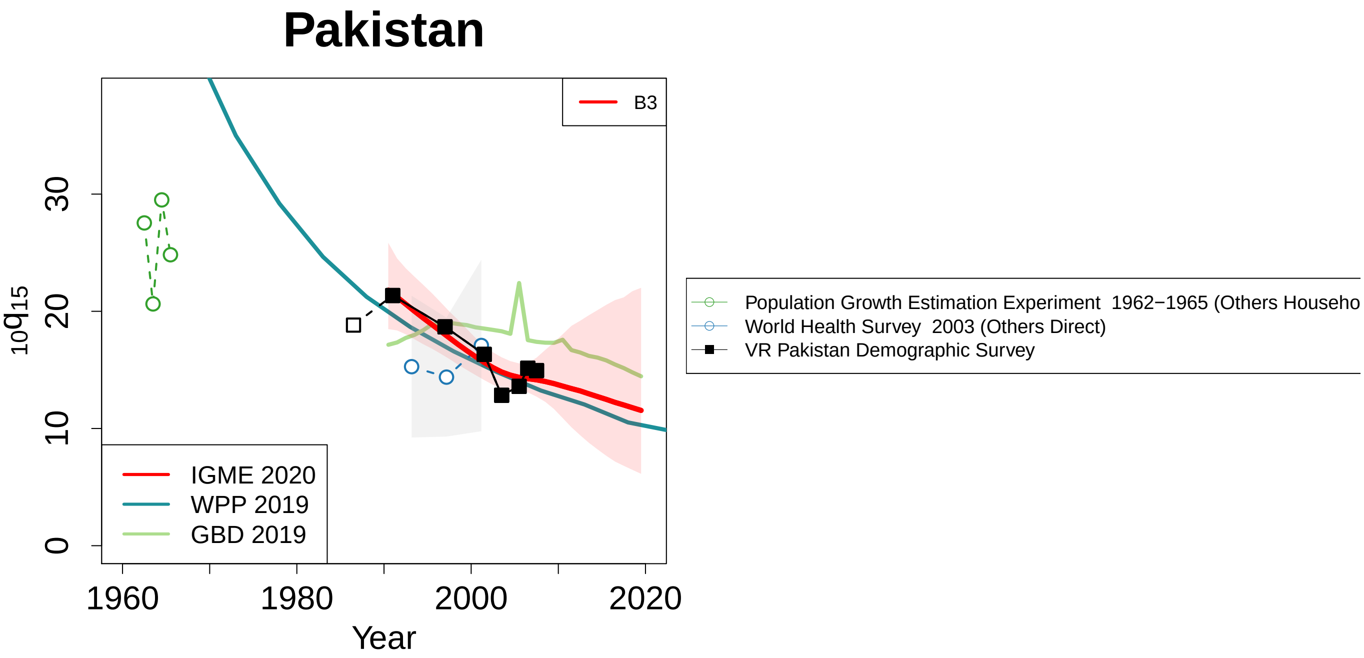

Pakistan
B3
IGME 2020
WPP 2019
GBD 2019
Population Growth Estimation Experiment 1962−1965 (Others Househo
World Health Survey 2003 (Others Direct)
VR Pakistan Demographic Survey
$_{10}q_{15}$
Year

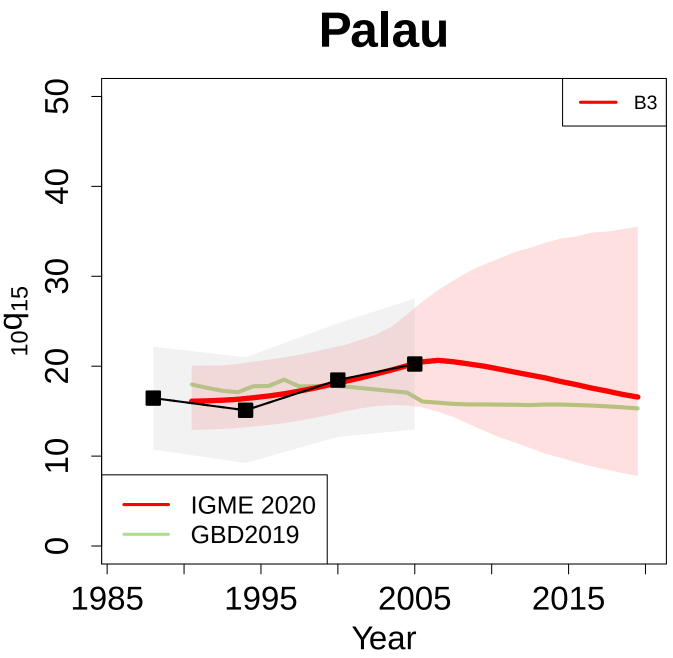

Palau
B3
IGME 2020
GBD2019
$_{10}q_{15}$
Year

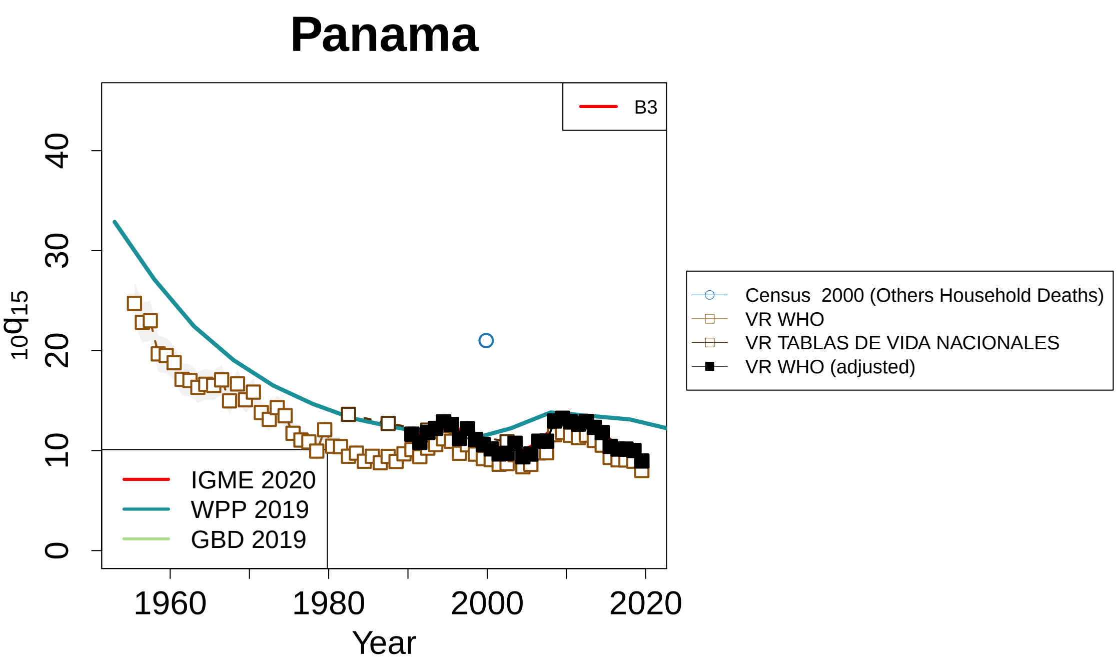
Panama
B3
Census 2000 (Others Household Deaths)
VR WHO
VR TABLAS DE VIDA NACIONALES
VR WHO (adjusted)
IGME 2020
WPP 2019
GBD 2019
$_{10}q_{15}$
Year

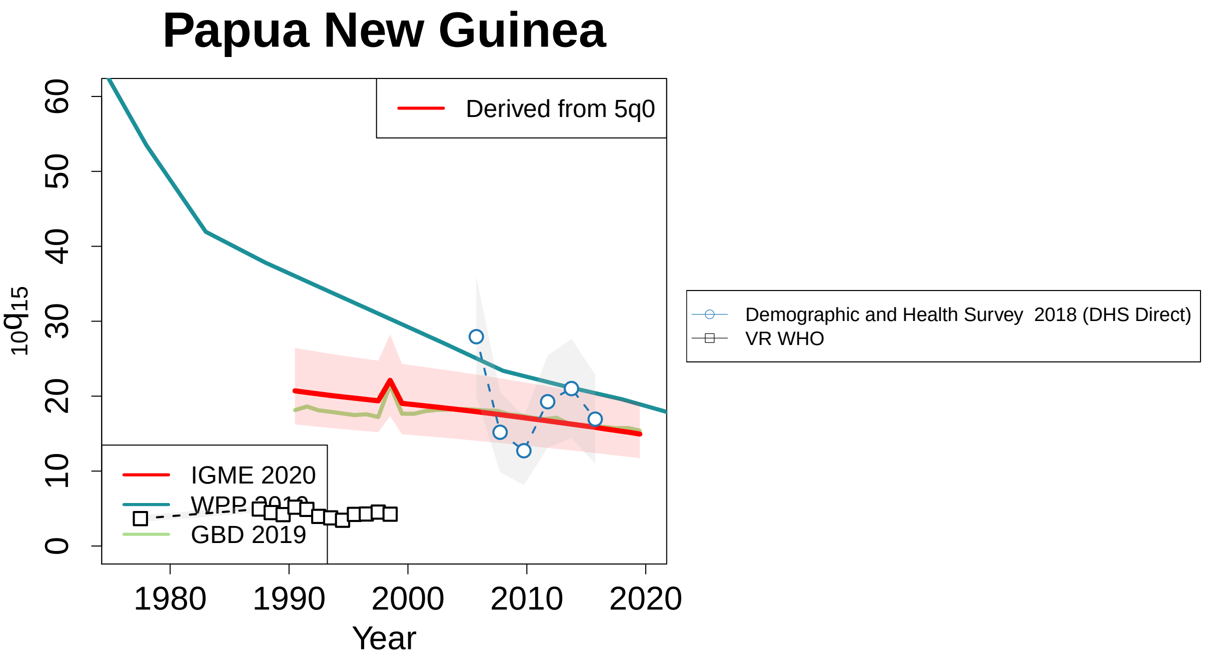
Papua New Guinea
Derived from 5q0
Demographic and Health Survey 2018 (DHS Direct)
VR WHO
IGME 2020
WPP 2019
GBD 2019
$_{10}q_{15}$
Year

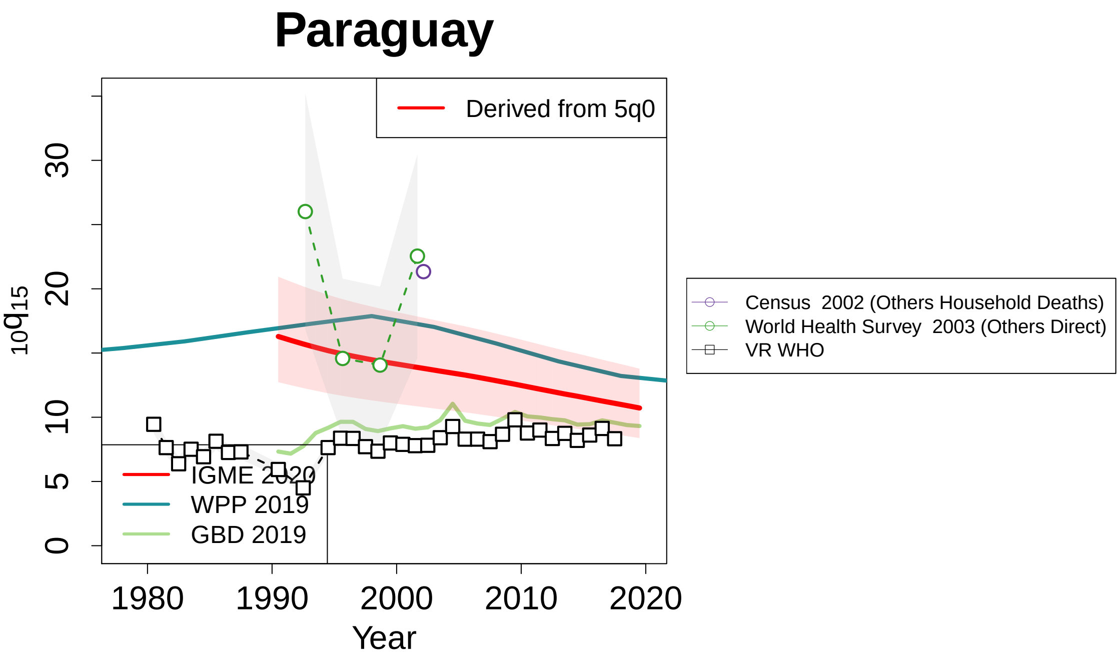
Paraguay
Derived from 5q0
IGME 2020
WPP 2019
GBD 2019
Census 2002 (Others Household Deaths)
World Health Survey 2003 (Others Direct)
VR WHO
0
5
10
20
30
1980
1990
2000
2010
2020
Year

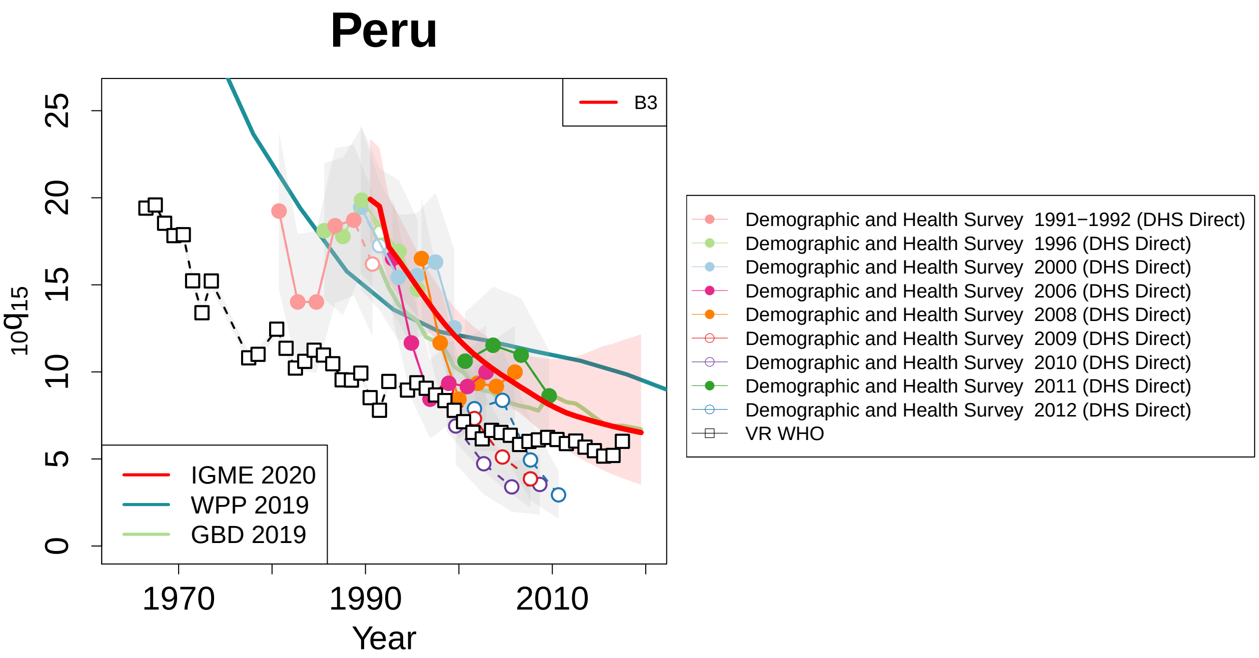
Peru
B3
IGME 2020
WPP 2019
GBD 2019
Demographic and Health Survey 1991−1992 (DHS Direct)
Demographic and Health Survey 1996 (DHS Direct)
Demographic and Health Survey 2000 (DHS Direct)
Demographic and Health Survey 2006 (DHS Direct)
Demographic and Health Survey 2008 (DHS Direct)
Demographic and Health Survey 2009 (DHS Direct)
Demographic and Health Survey 2010 (DHS Direct)
Demographic and Health Survey 2011 (DHS Direct)
Demographic and Health Survey 2012 (DHS Direct)
VR WHO
0
5
10
15
20
25
1970
1990
2010
Year

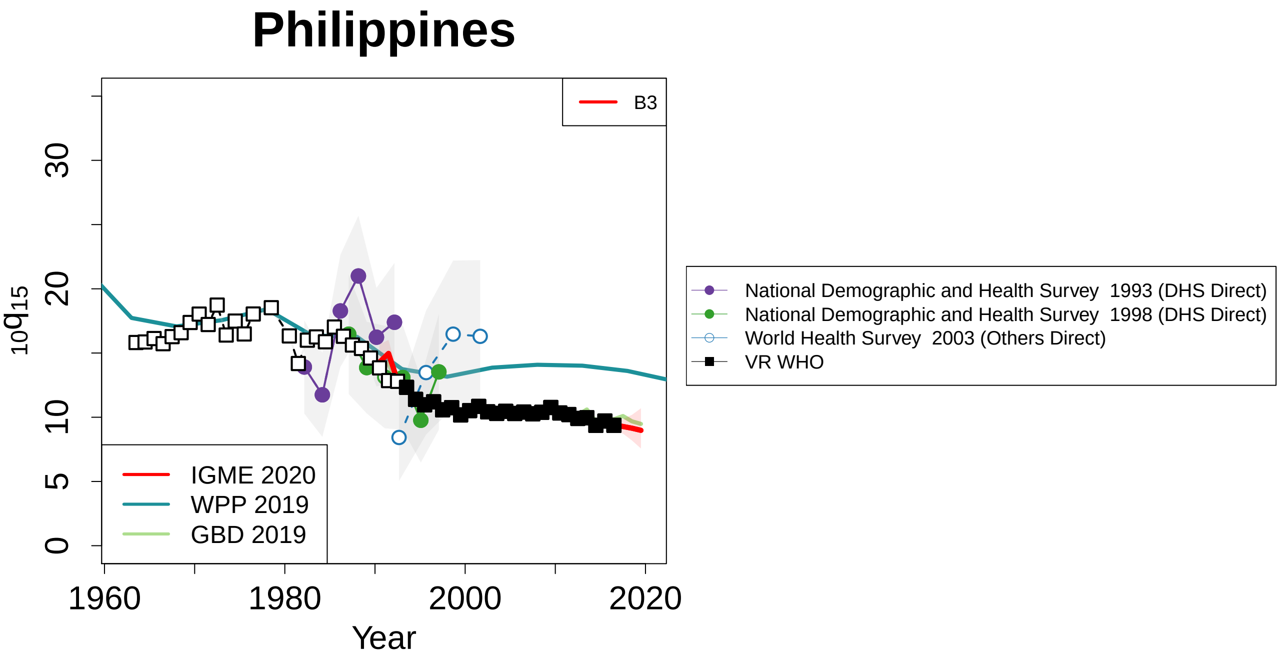
Philippines
B3
IGME 2020
WPP 2019
GBD 2019
National Demographic and Health Survey 1993 (DHS Direct)
National Demographic and Health Survey 1998 (DHS Direct)
World Health Survey 2003 (Others Direct)
VR WHO
$_{10}q_{15}$
0
5
10
20
30
1960
1980
2000
2020
Year

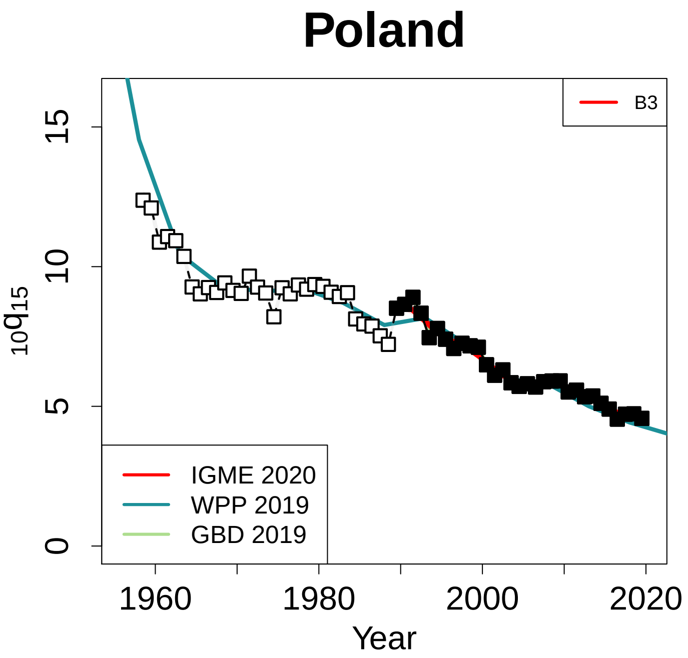
Poland
B3
IGME 2020
WPP 2019
GBD 2019
$_{10}q_{15}$
0
5
10
15
1960
1980
2000
2020
Year

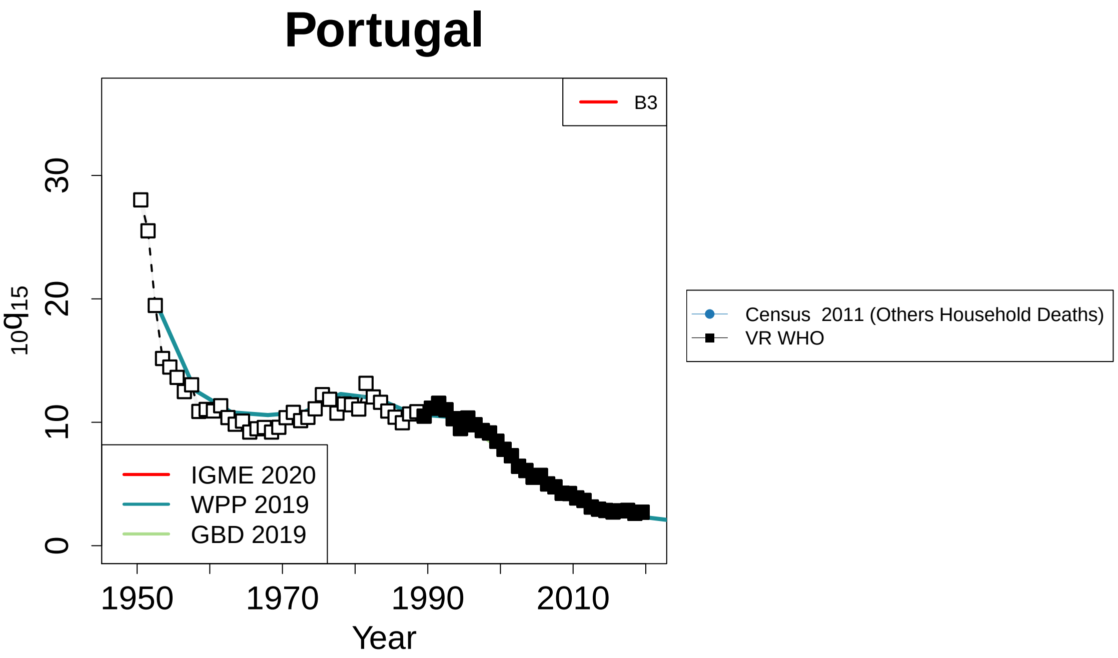
Portugal
B3
Census 2011 (Others Household Deaths)
VR WHO
IGME 2020
WPP 2019
GBD 2019
$_{10}q_{15}$
0
10
20
30
1950
1970
1990
2010
Year

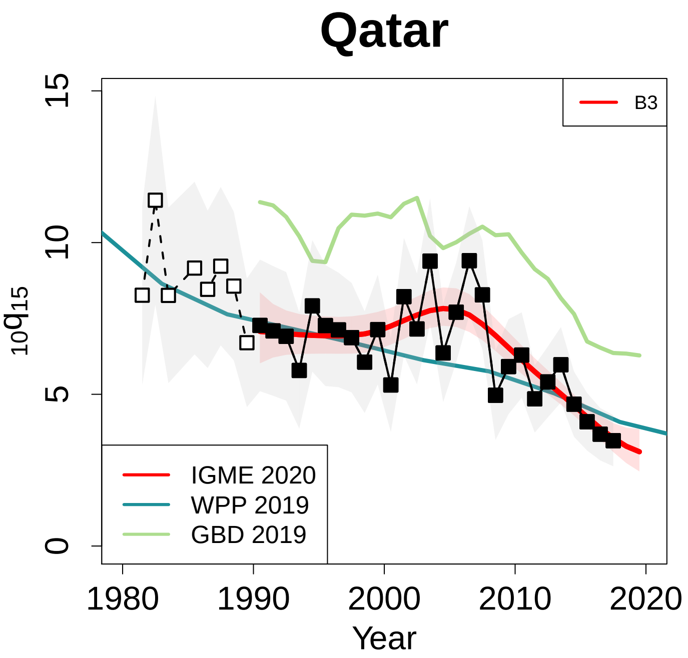
Qatar
B3
IGME 2020
WPP 2019
GBD 2019
$_{10}q_{15}$
0
5
10
15
1980
1990
2000
2010
2020
Year

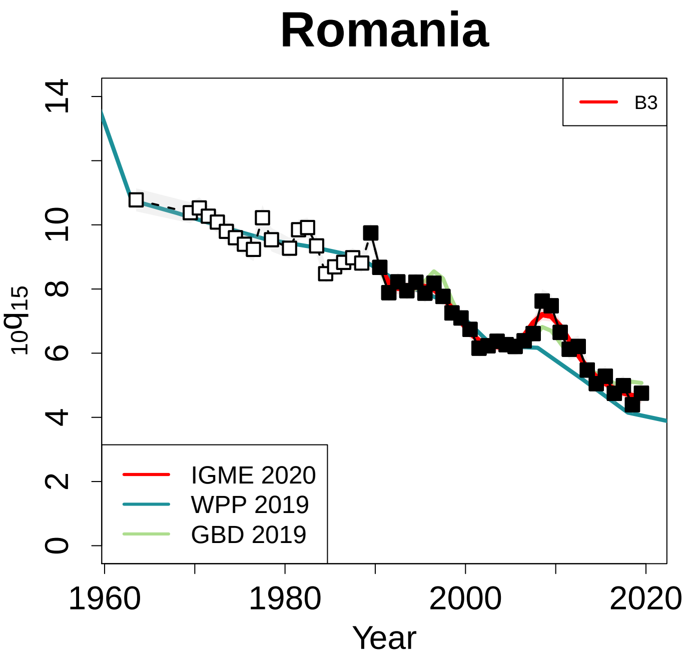
Romania
B3
IGME 2020
WPP 2019
GBD 2019
0
2
4
6
8
10
14
$_{10}q_{15}$
1960
1980
2000
2020
Year

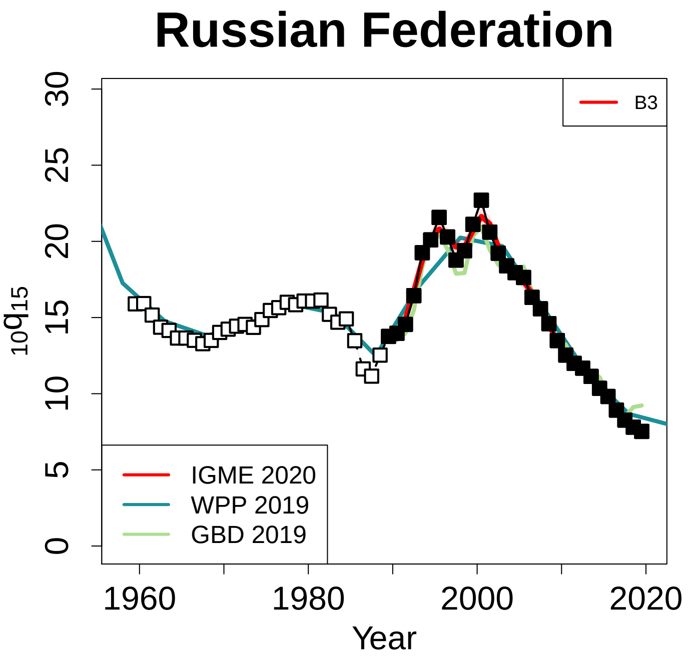
Russian Federation
B3
IGME 2020
WPP 2019
GBD 2019
0
5
10
15
20
25
30
$_{10}q_{15}$
1960
1980
2000
2020
Year

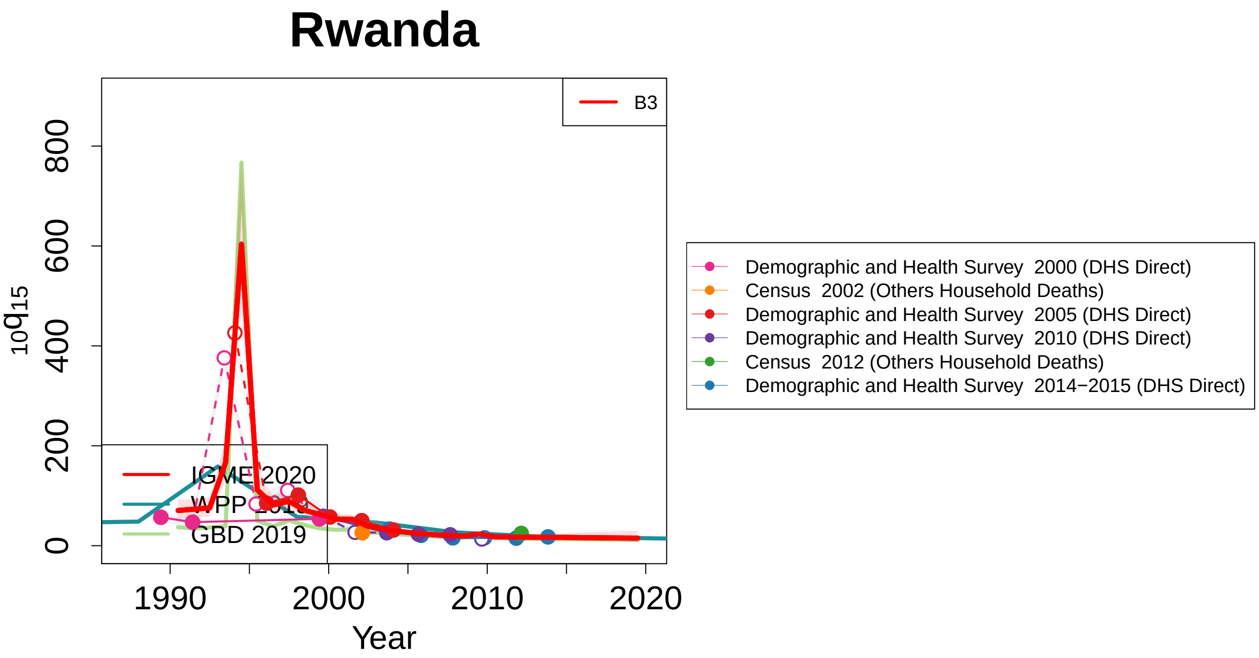
Rwanda
B3
800
600
400
200
0
$_{10}q_{15}$
IGME 2020
WPP 2019
GBD 2019
1990
2000
2010
2020
Year
Demographic and Health Survey 2000 (DHS Direct)
Census 2002 (Others Household Deaths)
Demographic and Health Survey 2005 (DHS Direct)
Demographic and Health Survey 2010 (DHS Direct)
Census 2012 (Others Household Deaths)
Demographic and Health Survey 2014−2015 (DHS Direct)

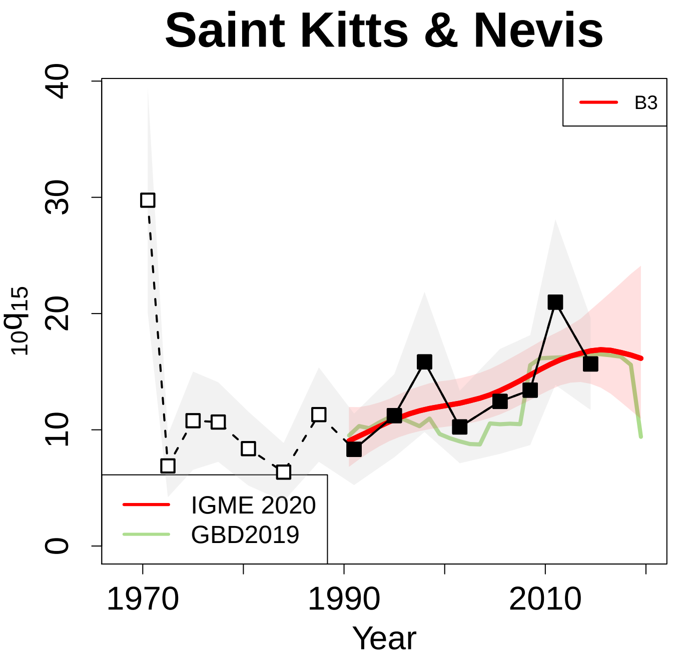
Saint Kitts & Nevis
B3
40
30
20
10
0
$_{10}q_{15}$
IGME 2020
GBD2019
1970
1990
2010
Year

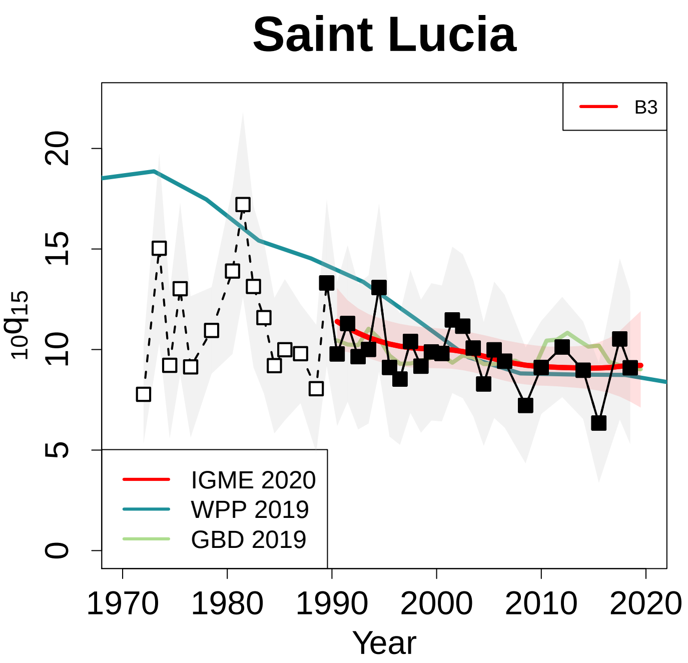
Saint Lucia
B3
IGME 2020
WPP 2019
GBD 2019
$_{10}q_{15}$
Year

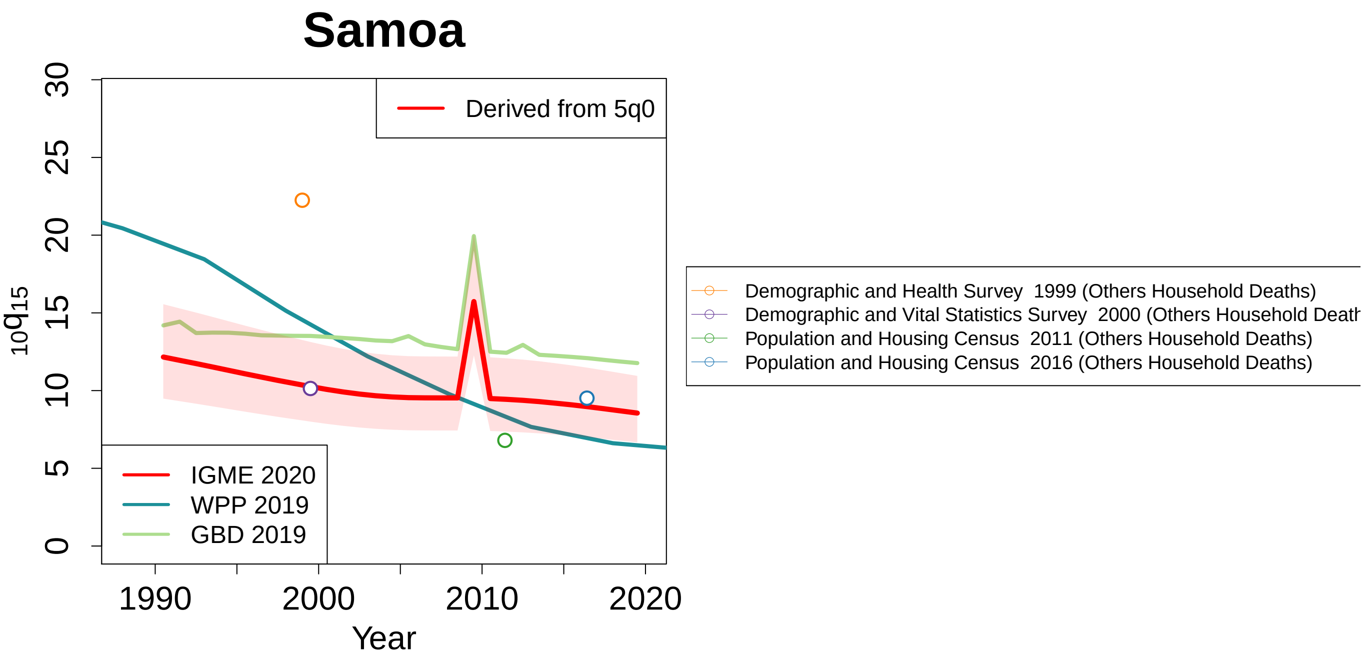
Samoa
Derived from 5q0
IGME 2020
WPP 2019
GBD 2019
Demographic and Health Survey 1999 (Others Household Deaths)
Demographic and Vital Statistics Survey 2000 (Others Household Death
Population and Housing Census 2011 (Others Household Deaths)
Population and Housing Census 2016 (Others Household Deaths)
$_{10}q_{15}$
Year

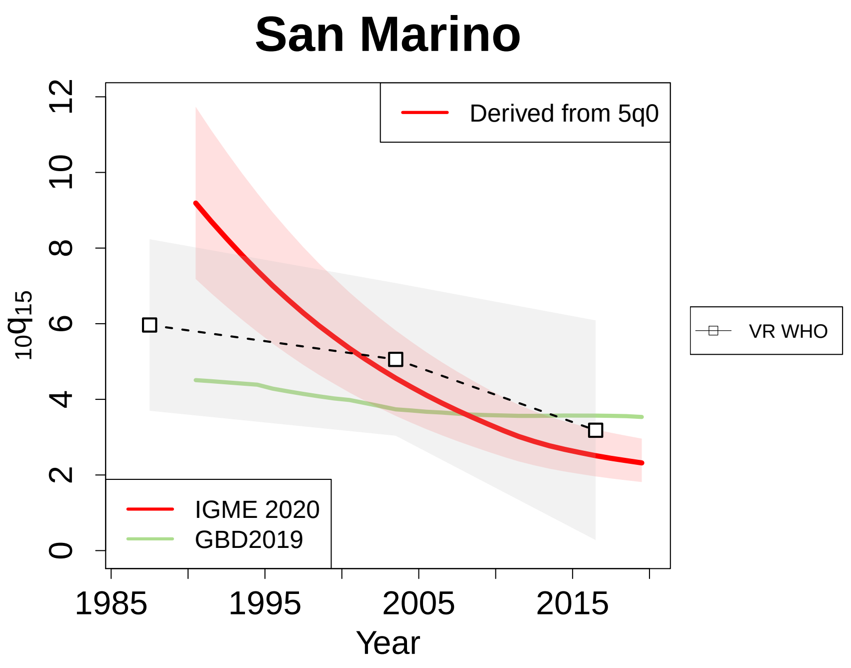
San Marino
Derived from 5q0
VR WHO
IGME 2020
GBD2019
$_{10}q_{15}$
0
2
4
6
8
10
12
1985
1995
2005
2015
Year

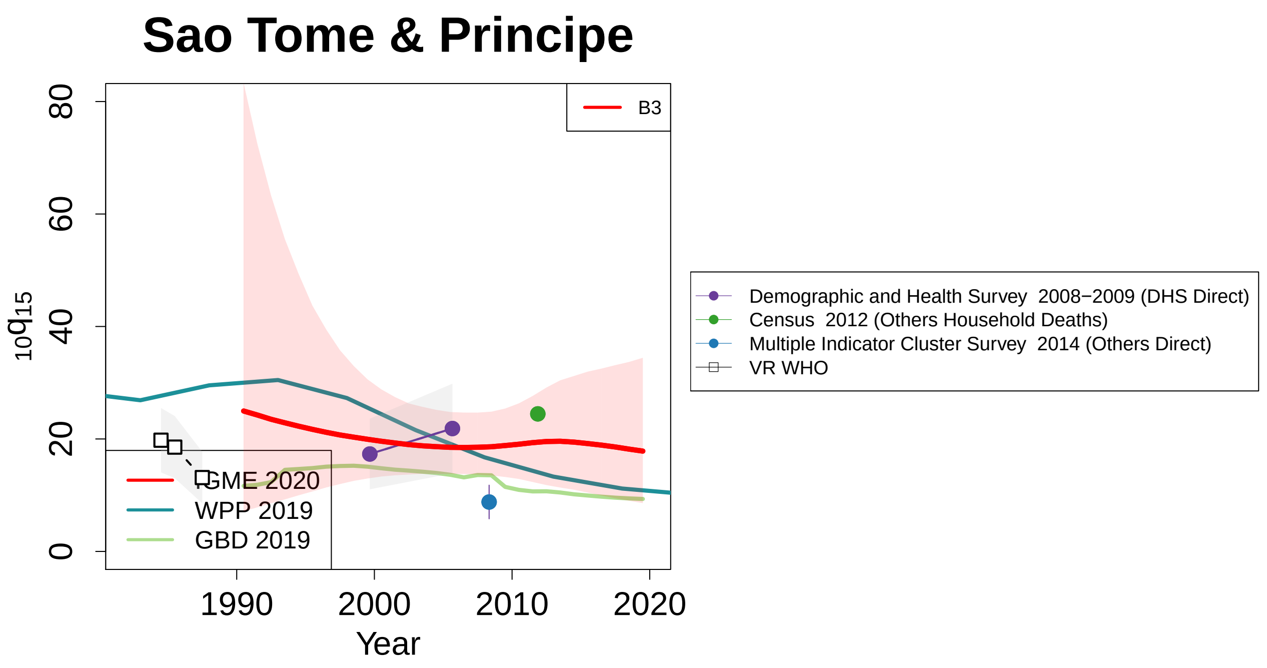
Sao Tome & Principe
B3
Demographic and Health Survey 2008−2009 (DHS Direct)
Census 2012 (Others Household Deaths)
Multiple Indicator Cluster Survey 2014 (Others Direct)
VR WHO
IGME 2020
WPP 2019
GBD 2019
$_{10}q_{15}$
0
20
40
60
80
1990
2000
2010
2020
Year

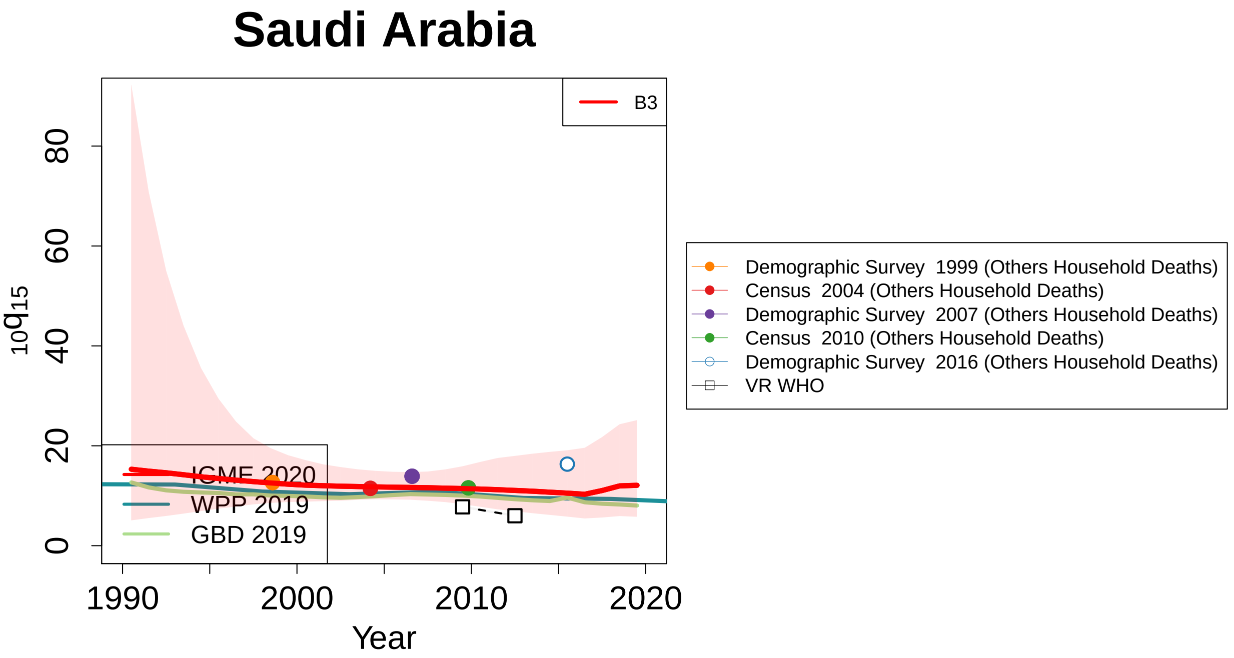
Saudi Arabia
B3
IGME 2020
WPP 2019
GBD 2019
10q15
Year
Demographic Survey 1999 (Others Household Deaths)
Census 2004 (Others Household Deaths)
Demographic Survey 2007 (Others Household Deaths)
Census 2010 (Others Household Deaths)
Demographic Survey 2016 (Others Household Deaths)
VR WHO

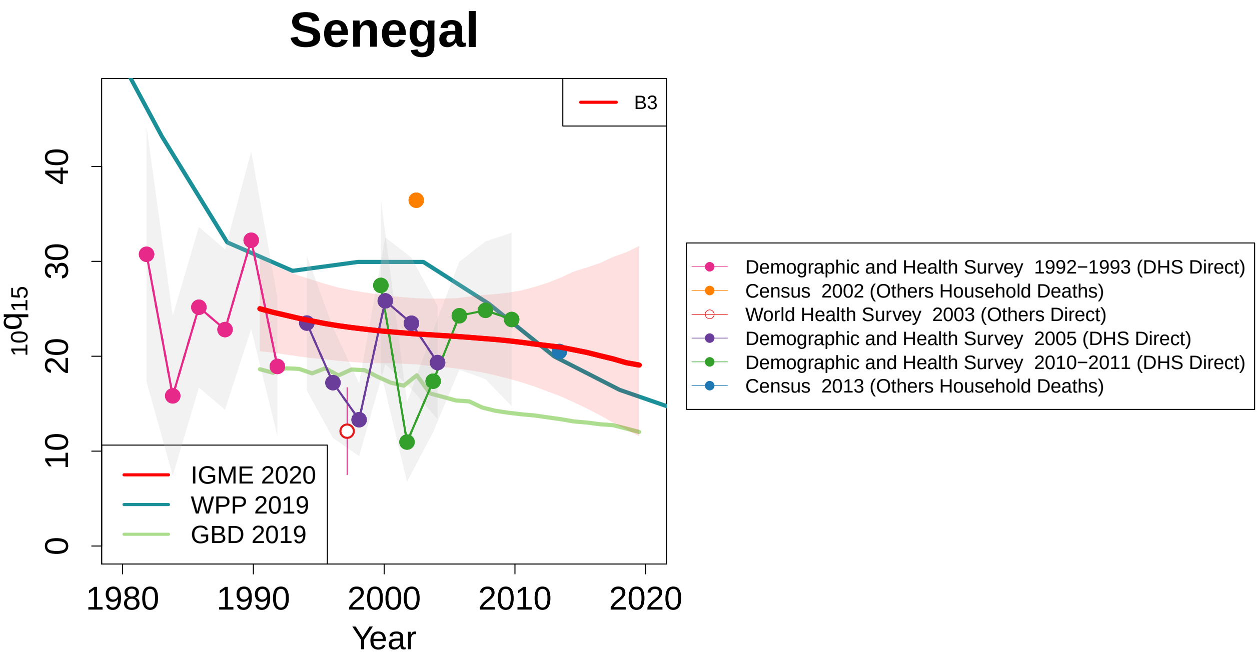
Senegal
B3
IGME 2020
WPP 2019
GBD 2019
10q15
Year
Demographic and Health Survey 1992−1993 (DHS Direct)
Census 2002 (Others Household Deaths)
World Health Survey 2003 (Others Direct)
Demographic and Health Survey 2005 (DHS Direct)
Demographic and Health Survey 2010−2011 (DHS Direct)
Census 2013 (Others Household Deaths)

Serbia

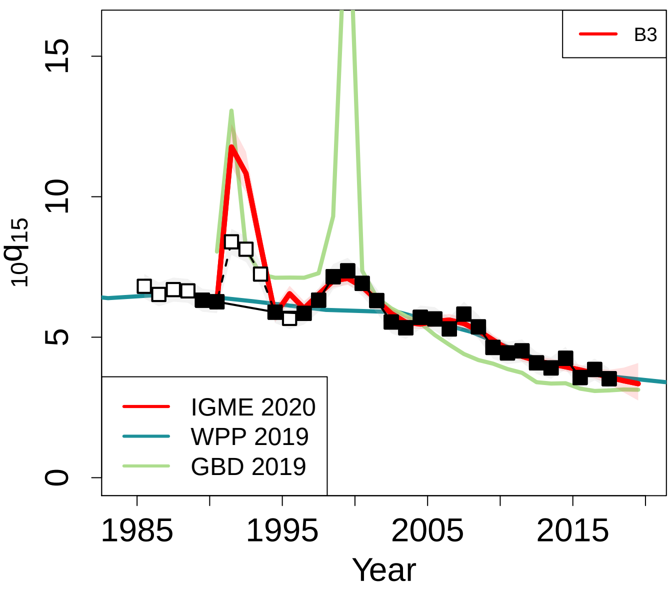

Seychelles

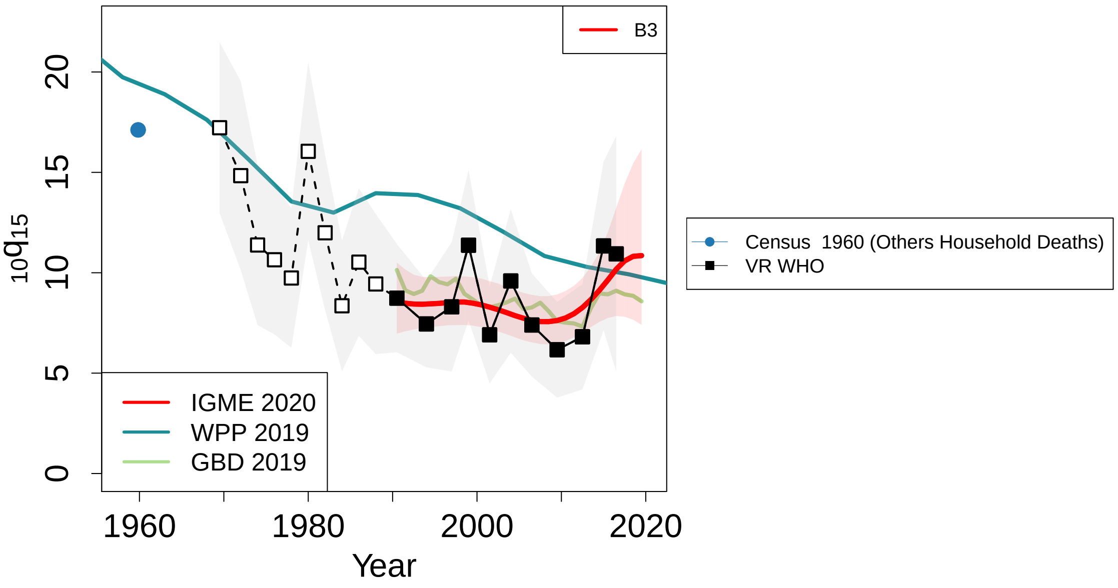

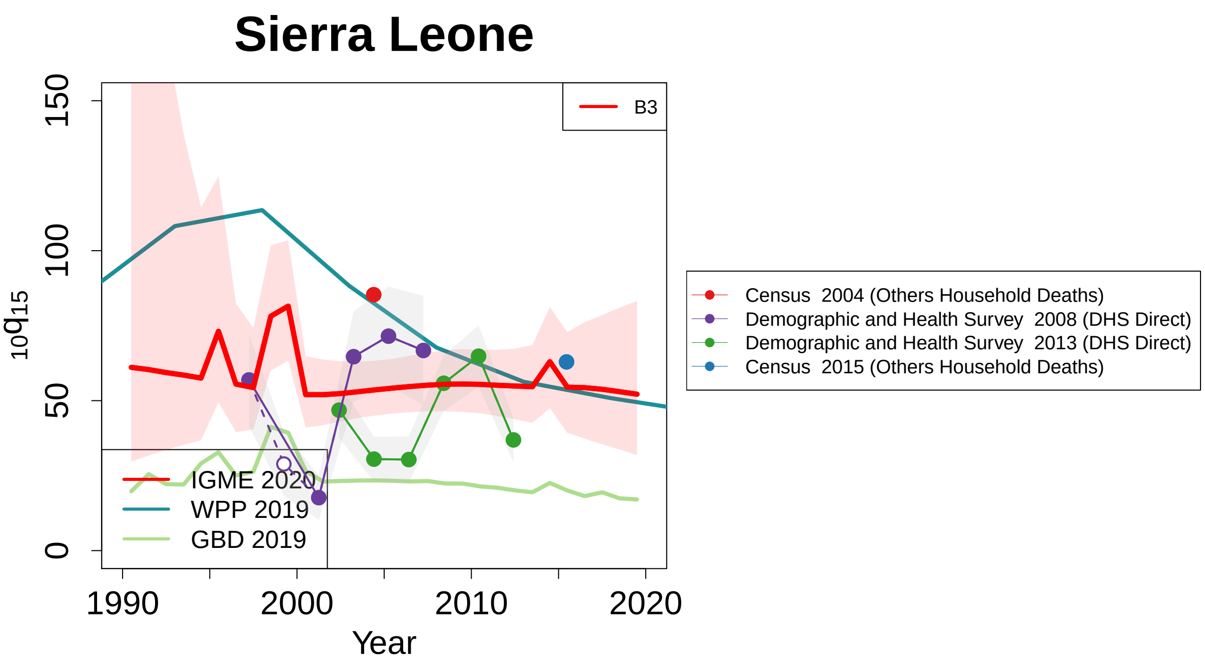
Sierra Leone
B3
Census 2004 (Others Household Deaths)
Demographic and Health Survey 2008 (DHS Direct)
Demographic and Health Survey 2013 (DHS Direct)
Census 2015 (Others Household Deaths)
IGME 2020
WPP 2019
GBD 2019
$_{10}q_{15}$
Year

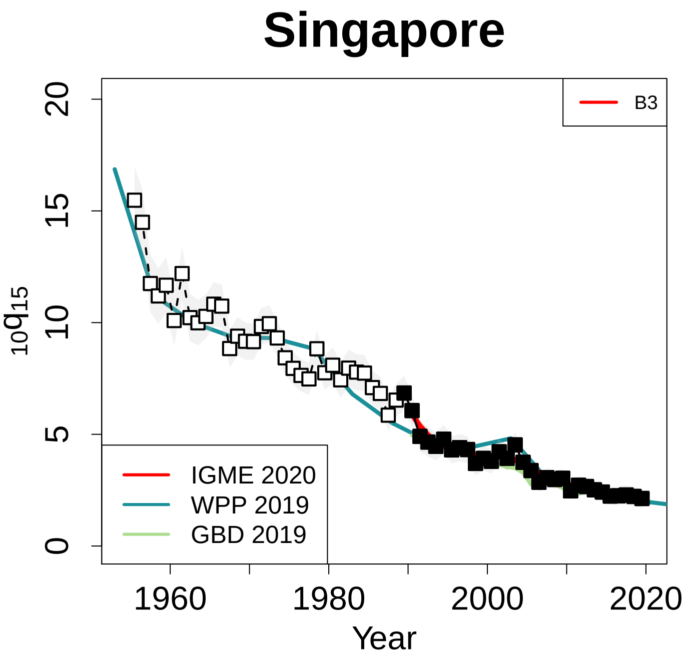
Singapore
B3
IGME 2020
WPP 2019
GBD 2019
$_{10}q_{15}$
Year

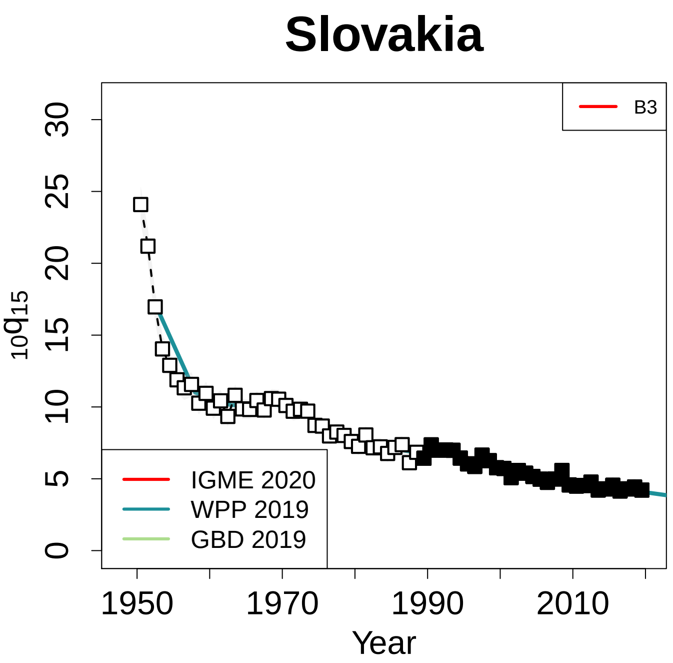
Slovakia
B3
IGME 2020
WPP 2019
GBD 2019
$_{10}q_{15}$
0
5
10
15
20
25
30
1950
1970
1990
2010
Year

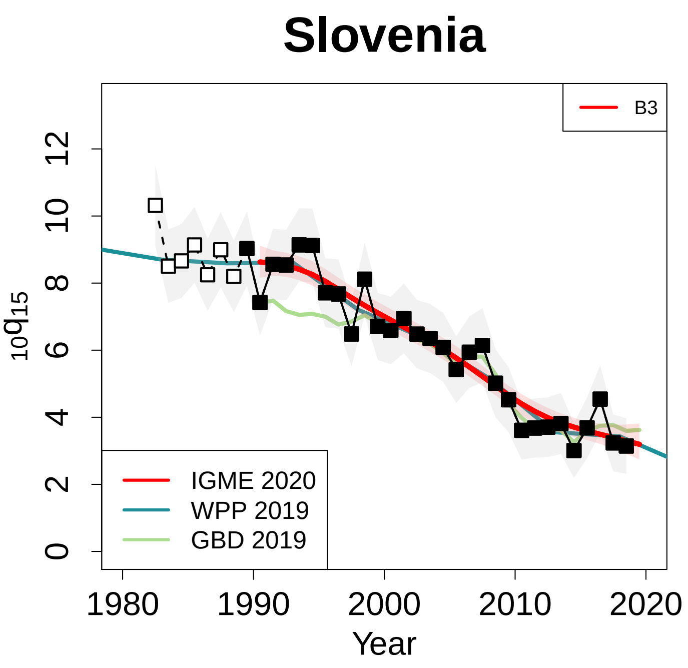
Slovenia
B3
IGME 2020
WPP 2019
GBD 2019
$_{10}q_{15}$
0
2
4
6
8
10
12
1980
1990
2000
2010
2020
Year

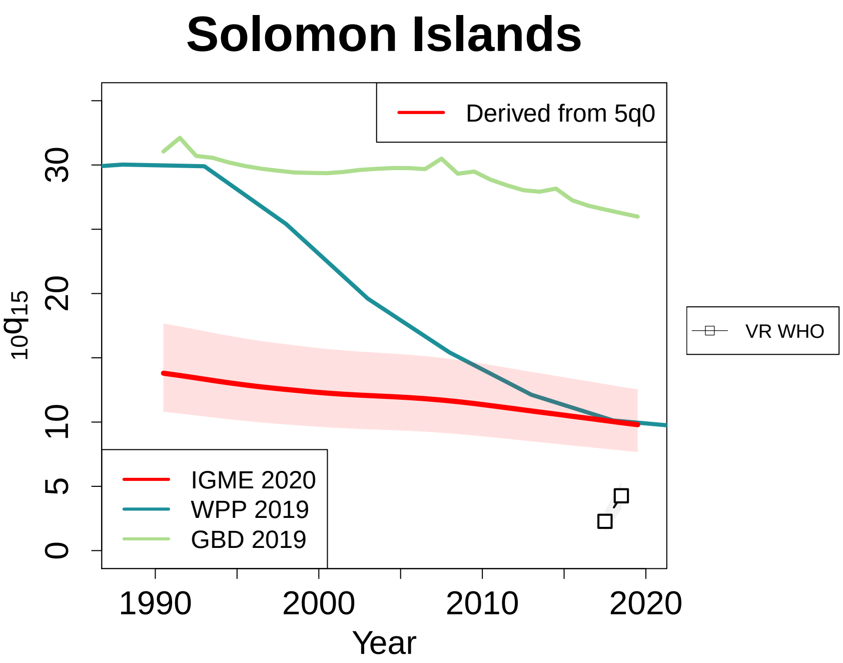
Solomon Islands
Derived from 5q0
VR WHO
$_{10}q_{15}$
0
5
10
20
30
IGME 2020
WPP 2019
GBD 2019
1990
2000
2010
2020
Year

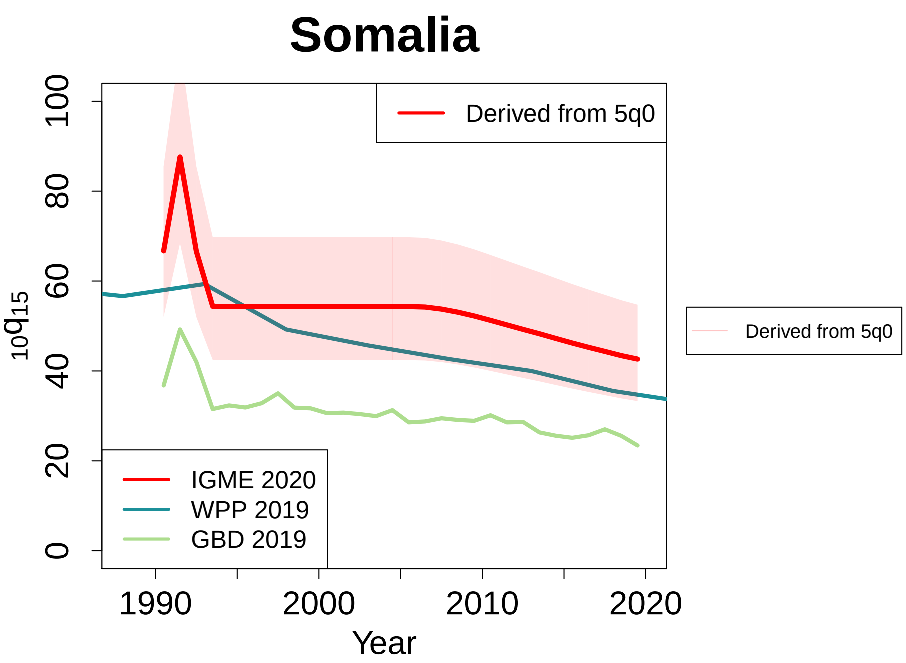
Somalia
Derived from 5q0
Derived from 5q0
$_{10}q_{15}$
0
20
40
60
80
100
IGME 2020
WPP 2019
GBD 2019
1990
2000
2010
2020
Year

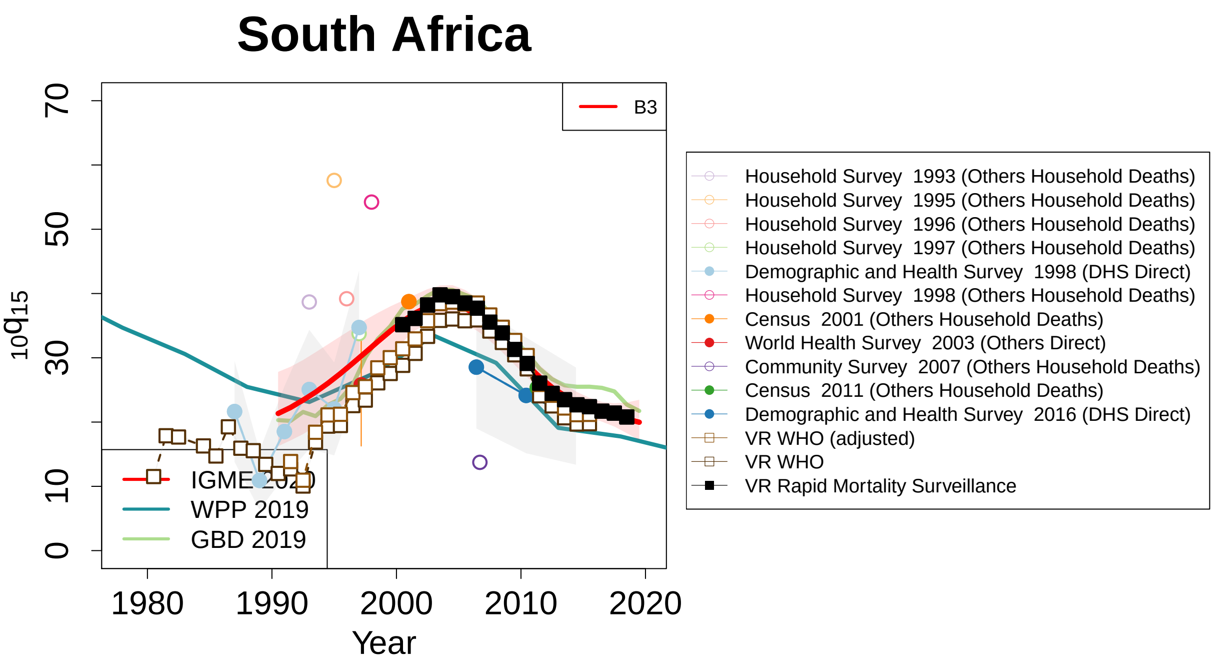

South Africa
B3
IGME 2020
WPP 2019
GBD 2019
70
50
30
10
0
$_{10}q_{15}$
1980
1990
2000
2010
2020
Year
Household Survey 1993 (Others Household Deaths)
Household Survey 1995 (Others Household Deaths)
Household Survey 1996 (Others Household Deaths)
Household Survey 1997 (Others Household Deaths)
Demographic and Health Survey 1998 (DHS Direct)
Household Survey 1998 (Others Household Deaths)
Census 2001 (Others Household Deaths)
World Health Survey 2003 (Others Direct)
Community Survey 2007 (Others Household Deaths)
Census 2011 (Others Household Deaths)
Demographic and Health Survey 2016 (DHS Direct)
VR WHO (adjusted)
VR WHO
VR Rapid Mortality Surveillance

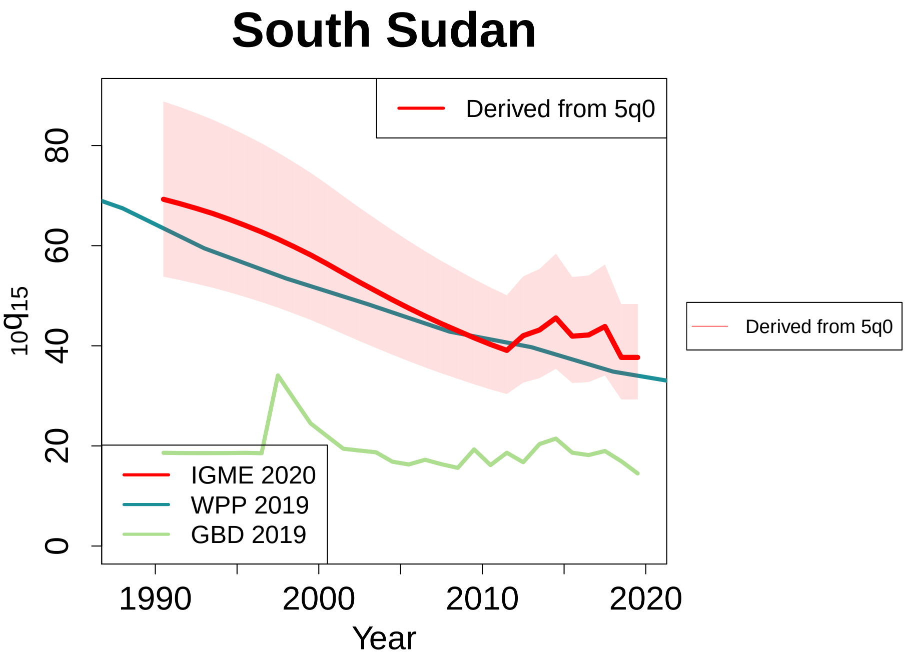

South Sudan
Derived from 5q0
IGME 2020
WPP 2019
GBD 2019
80
60
40
20
0
$_{10}q_{15}$
1990
2000
2010
2020
Year
Derived from 5q0

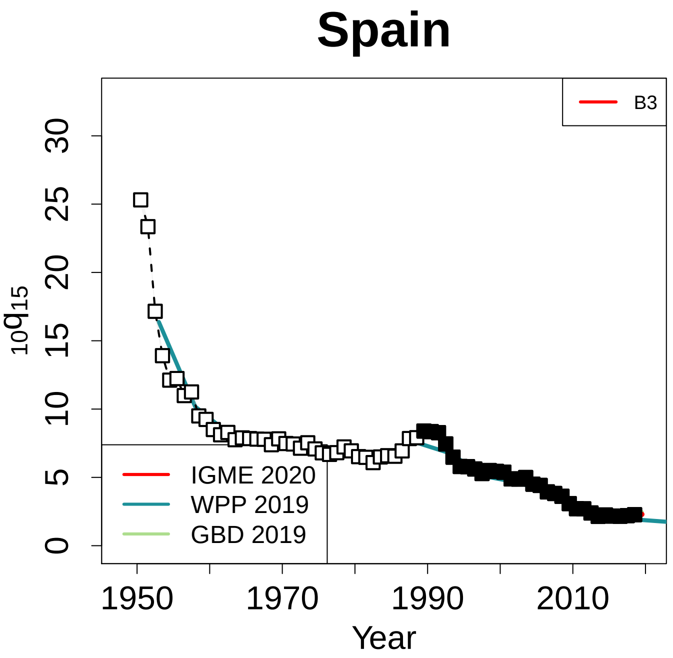
Spain
B3
IGME 2020
WPP 2019
GBD 2019
$_{10}q_{15}$
Year

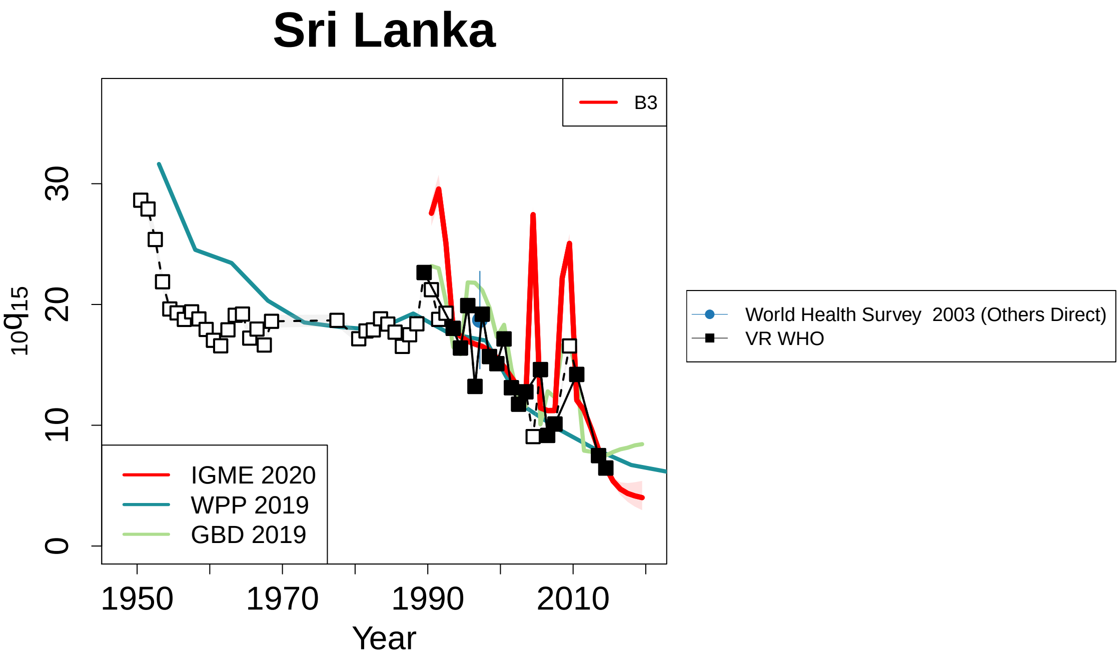
Sri Lanka
B3
IGME 2020
WPP 2019
GBD 2019
World Health Survey 2003 (Others Direct)
VR WHO
$_{10}q_{15}$
Year

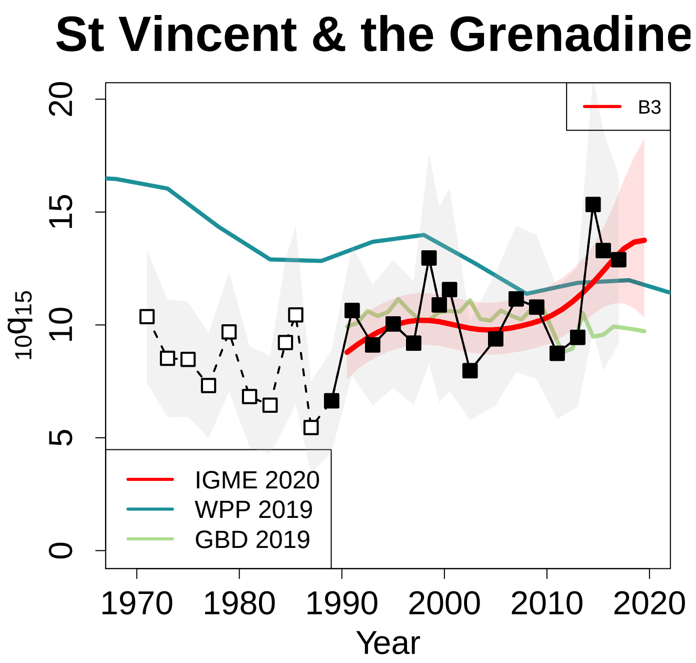
St Vincent & the Grenadine
B3
IGME 2020
WPP 2019
GBD 2019
$_{10}q_{15}$
Year

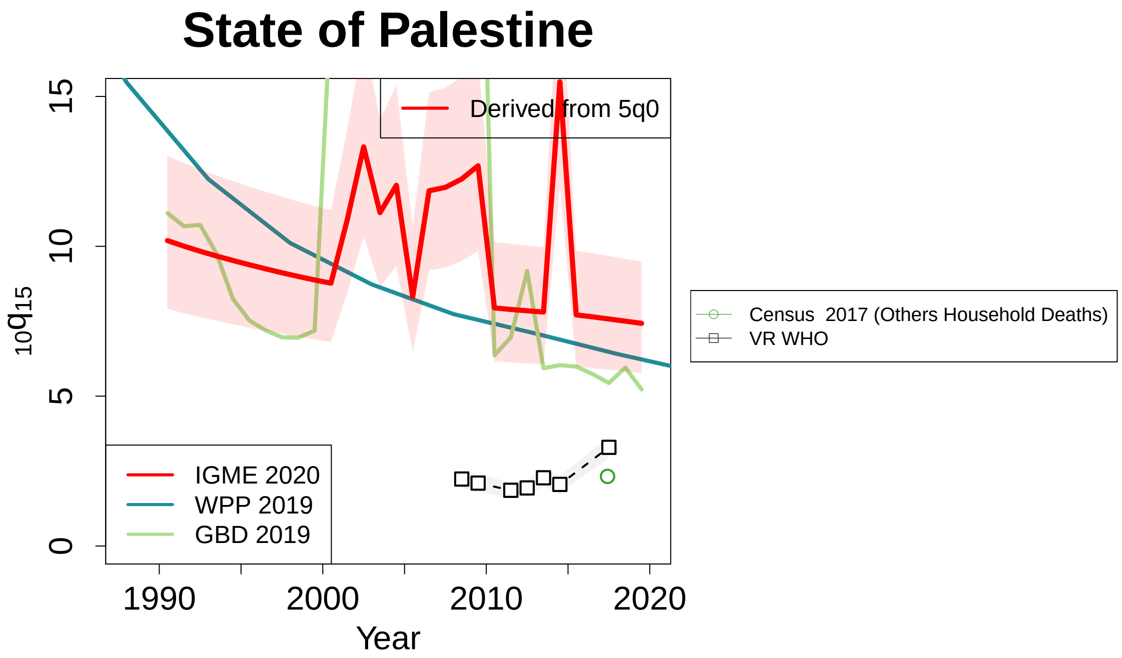
State of Palestine
Derived from 5q0
IGME 2020
WPP 2019
GBD 2019
Census 2017 (Others Household Deaths)
VR WHO
$_{10}q_{15}$
Year

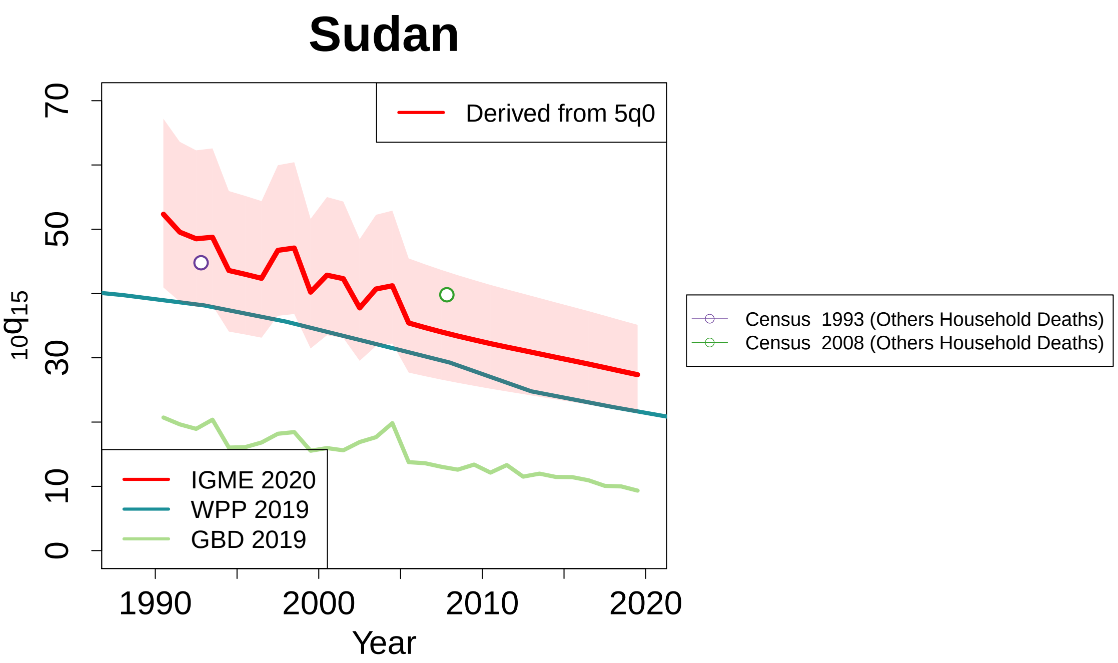
Sudan
Derived from 5q0
Census 1993 (Others Household Deaths)
Census 2008 (Others Household Deaths)
IGME 2020
WPP 2019
GBD 2019
$_{10}q_{15}$
Year

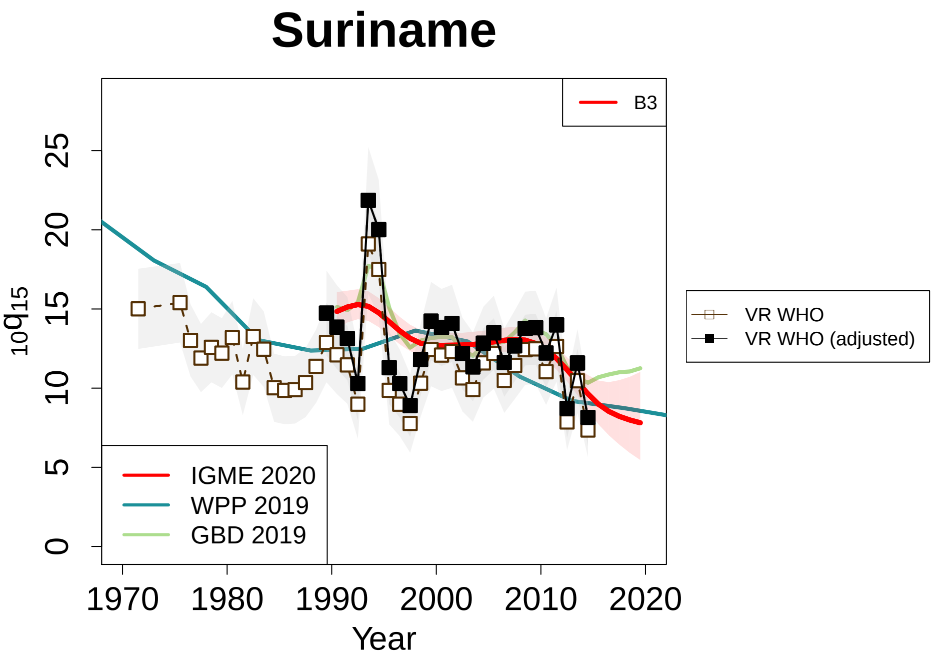
Suriname
B3
VR WHO
VR WHO (adjusted)
IGME 2020
WPP 2019
GBD 2019
$_{10}q_{15}$
Year

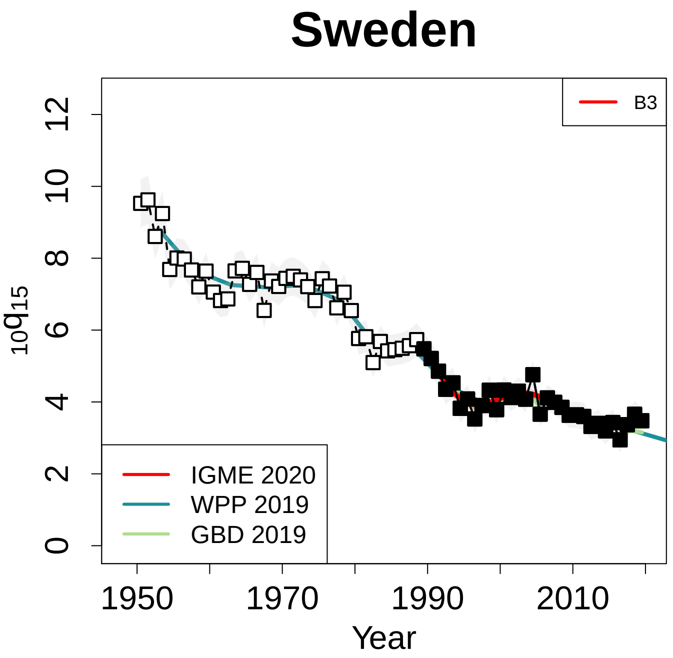

Sweden
B3
IGME 2020
WPP 2019
GBD 2019
0
2
4
6
8
10
12
$_{10}q_{15}$
1950
1970
1990
2010
Year

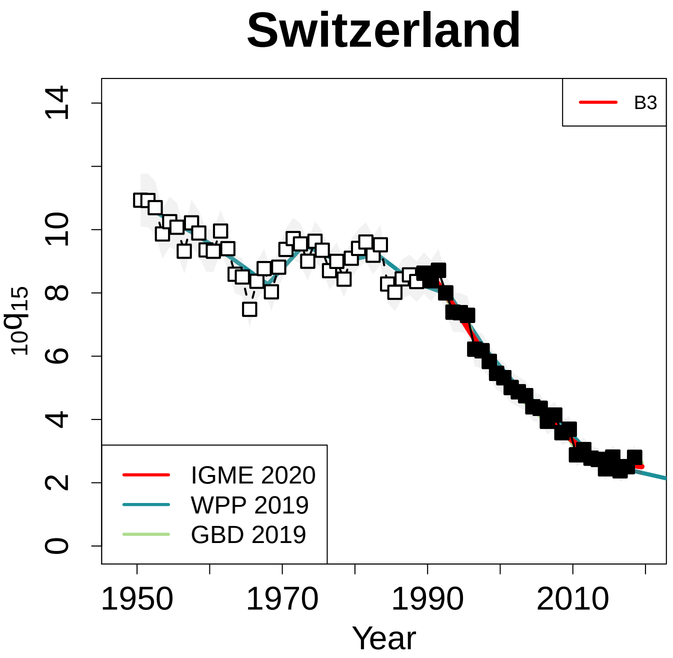

Switzerland
B3
IGME 2020
WPP 2019
GBD 2019
0
2
4
6
8
10
14
$_{10}q_{15}$
1950
1970
1990
2010
Year

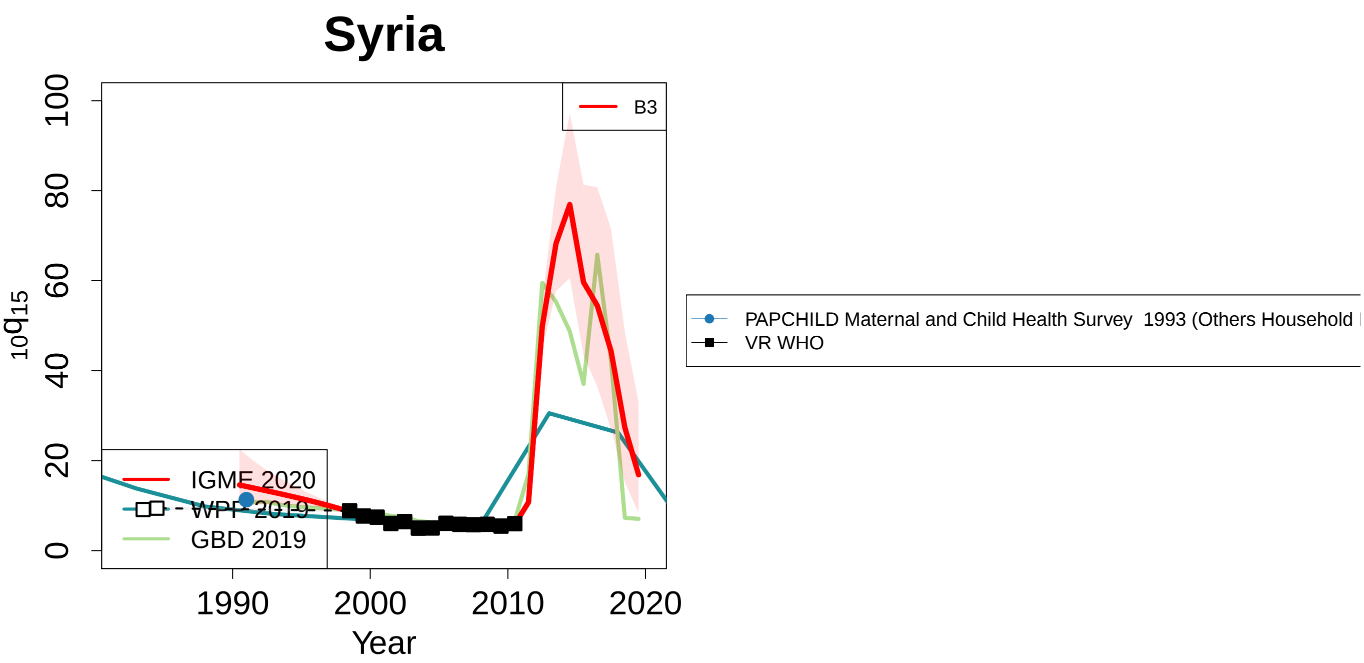
Syria
B3
IGME 2020
WPP 2019
GBD 2019
1990
2000
2010
2020
Year
PAPCHILD Maternal and Child Health Survey 1993 (Others Household
VR WHO

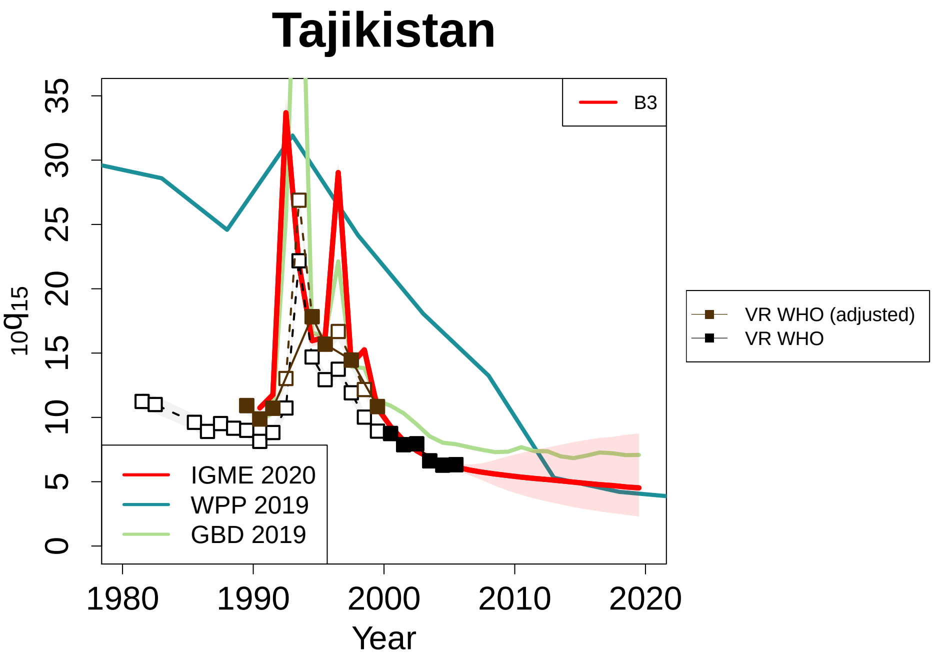
Tajikistan
B3
IGME 2020
WPP 2019
GBD 2019
1980
1990
2000
2010
2020
Year
VR WHO (adjusted)
VR WHO

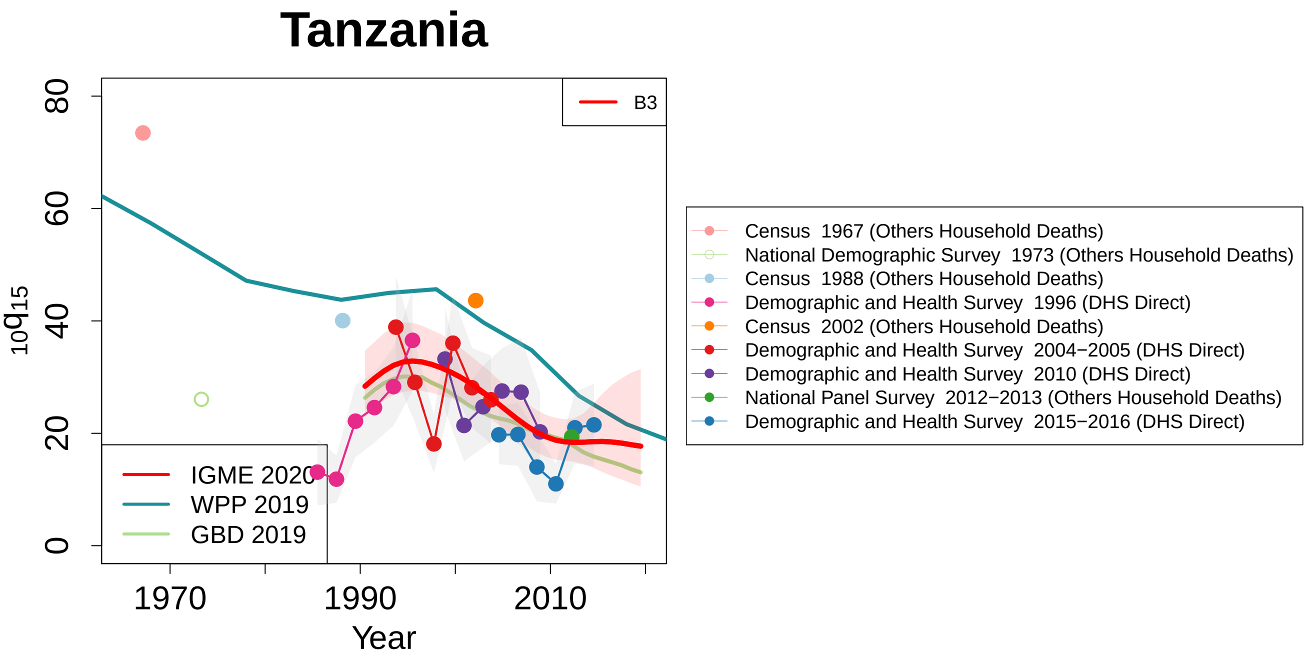
Tanzania
B3
IGME 2020
WPP 2019
GBD 2019
1970
1990
2010
Year
10q15
0
20
40
60
80
Census 1967 (Others Household Deaths)
National Demographic Survey 1973 (Others Household Deaths)
Census 1988 (Others Household Deaths)
Demographic and Health Survey 1996 (DHS Direct)
Census 2002 (Others Household Deaths)
Demographic and Health Survey 2004−2005 (DHS Direct)
Demographic and Health Survey 2010 (DHS Direct)
National Panel Survey 2012−2013 (Others Household Deaths)
Demographic and Health Survey 2015−2016 (DHS Direct)

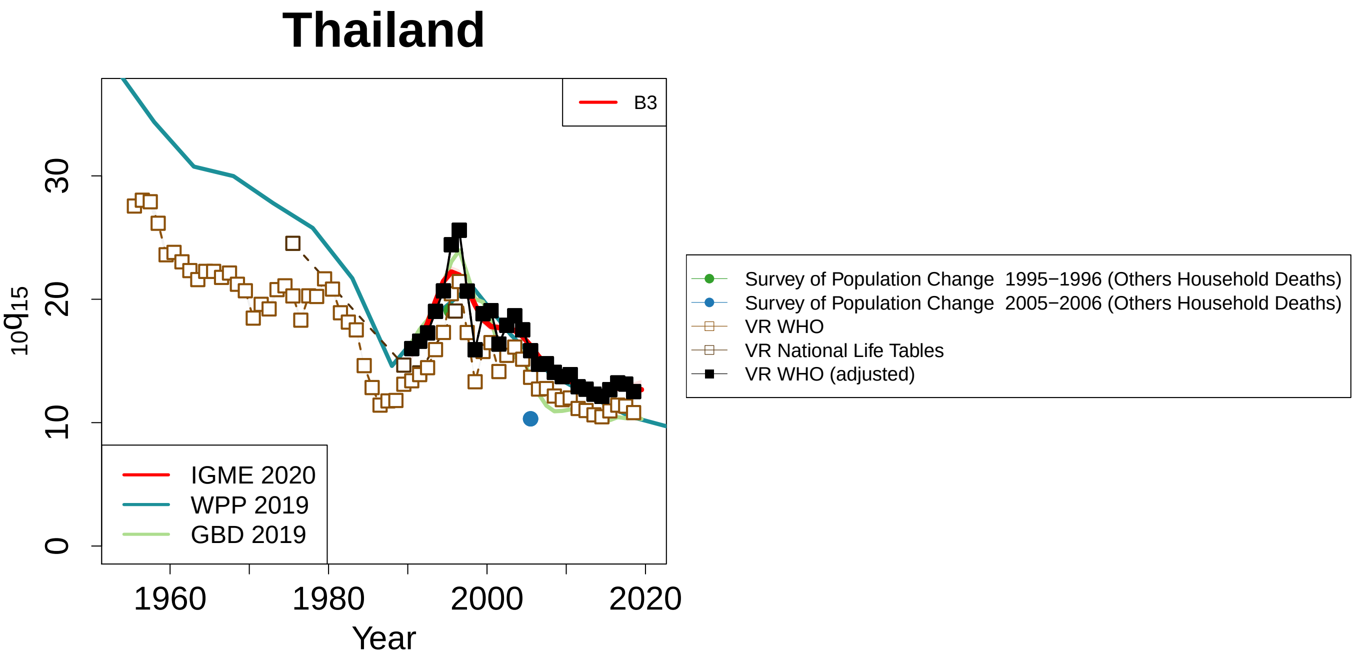
Thailand
B3
IGME 2020
WPP 2019
GBD 2019
1960
1980
2000
2020
Year
10q15
0
10
20
30
Survey of Population Change 1995−1996 (Others Household Deaths)
Survey of Population Change 2005−2006 (Others Household Deaths)
VR WHO
VR National Life Tables
VR WHO (adjusted)

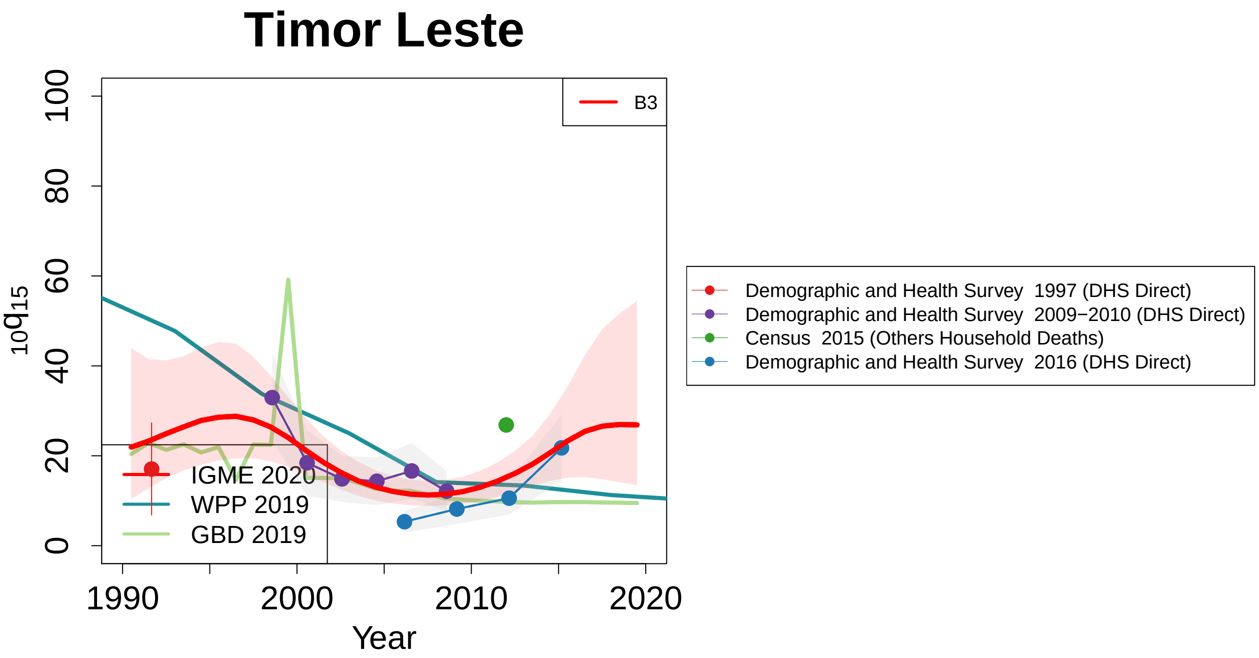
Timor Leste
B3
IGME 2020
WPP 2019
GBD 2019
Year
Demographic and Health Survey 1997 (DHS Direct)
Demographic and Health Survey 2009−2010 (DHS Direct)
Census 2015 (Others Household Deaths)
Demographic and Health Survey 2016 (DHS Direct)

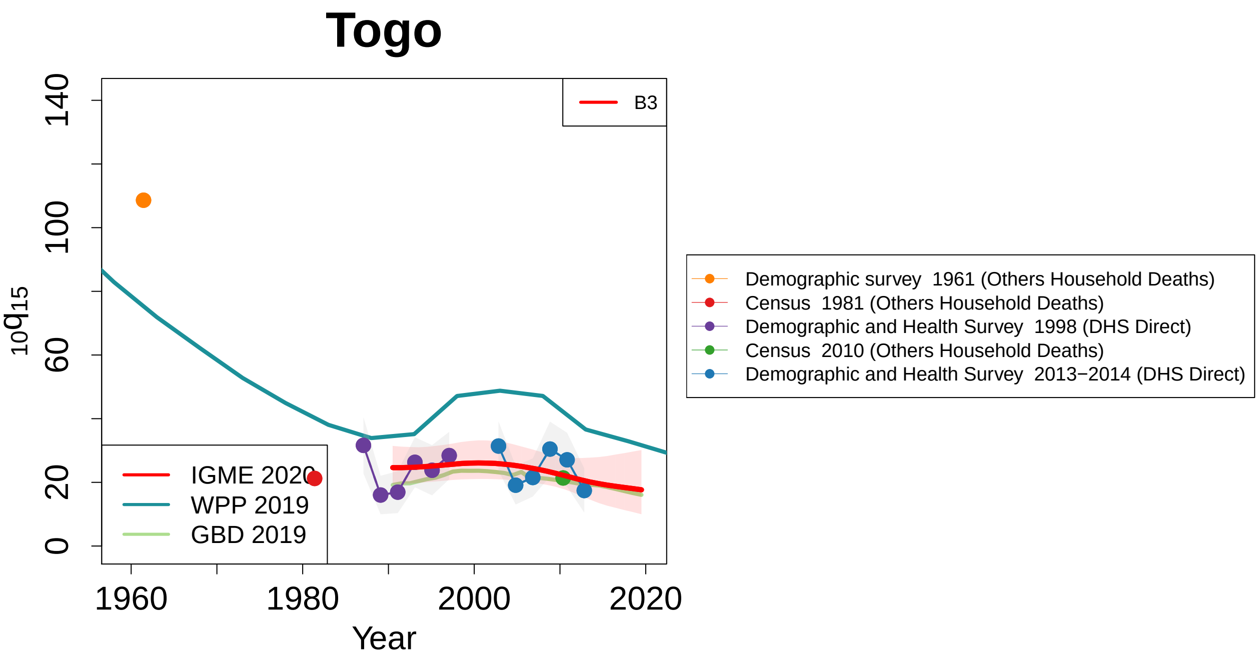
Togo
B3
IGME 2020
WPP 2019
GBD 2019
Year
Demographic survey 1961 (Others Household Deaths)
Census 1981 (Others Household Deaths)
Demographic and Health Survey 1998 (DHS Direct)
Census 2010 (Others Household Deaths)
Demographic and Health Survey 2013−2014 (DHS Direct)

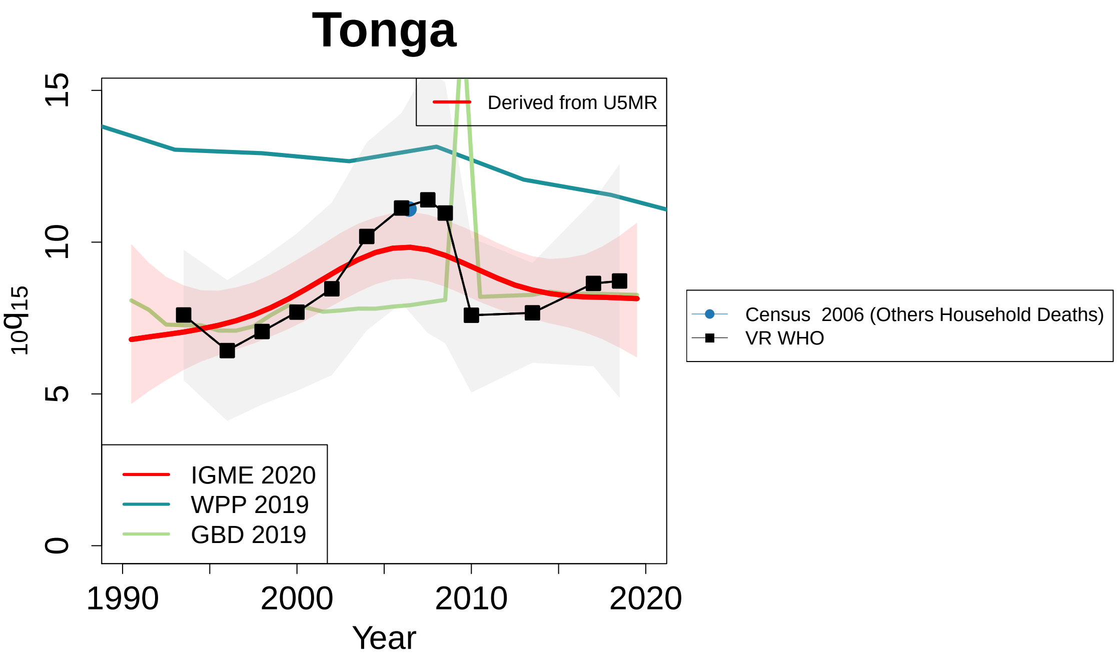
Tonga
Derived from U5MR
Census 2006 (Others Household Deaths)
VR WHO
IGME 2020
WPP 2019
GBD 2019
15
10
5
0
$_{10}q_{15}$
1990
2000
2010
2020
Year

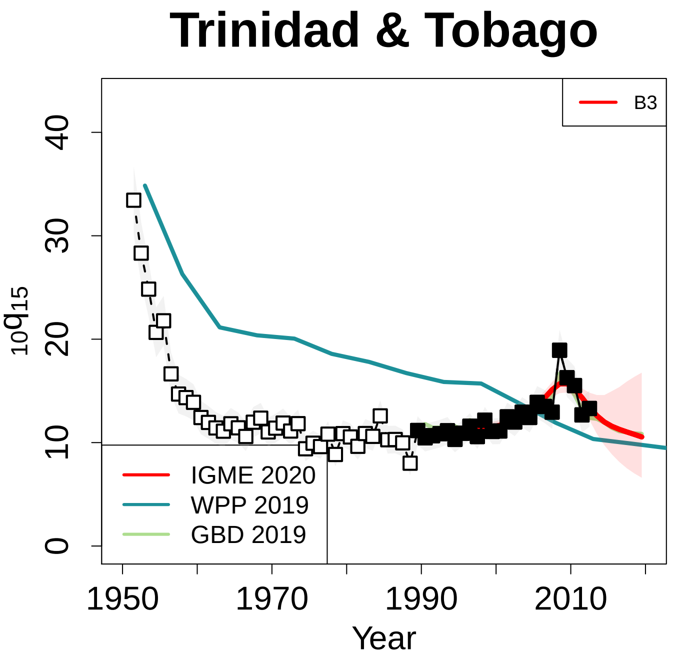
Trinidad & Tobago
B3
IGME 2020
WPP 2019
GBD 2019
40
30
20
10
0
$_{10}q_{15}$
1950
1970
1990
2010
Year

Tunisia

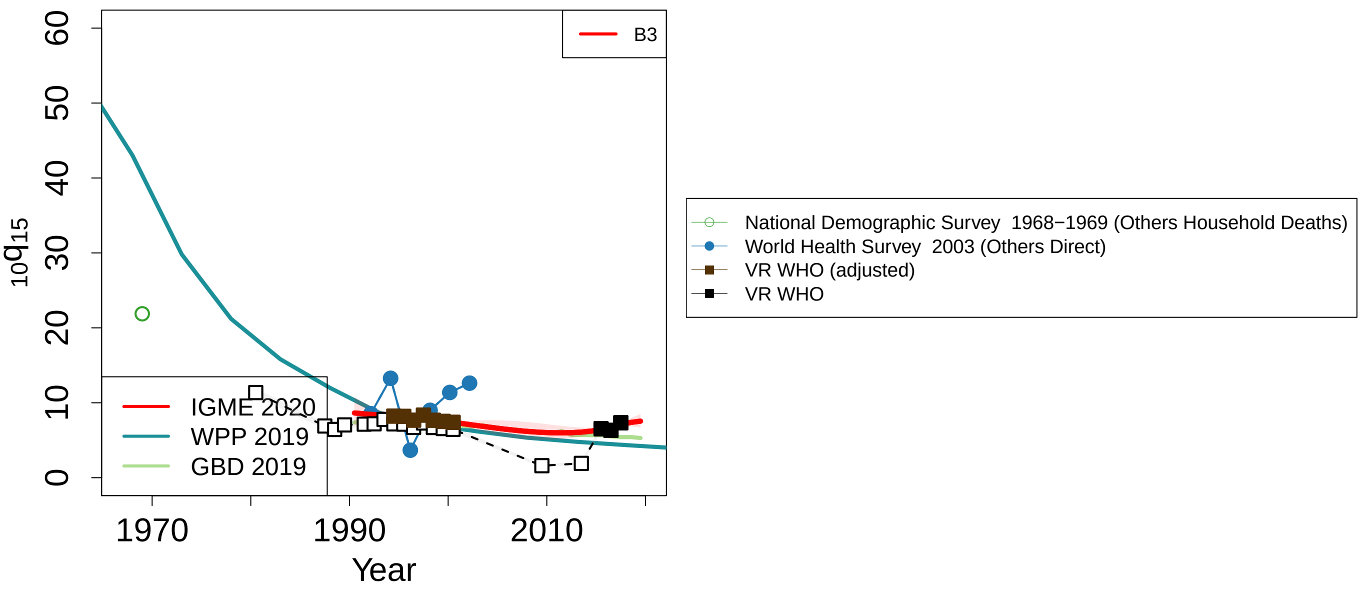

Turkey

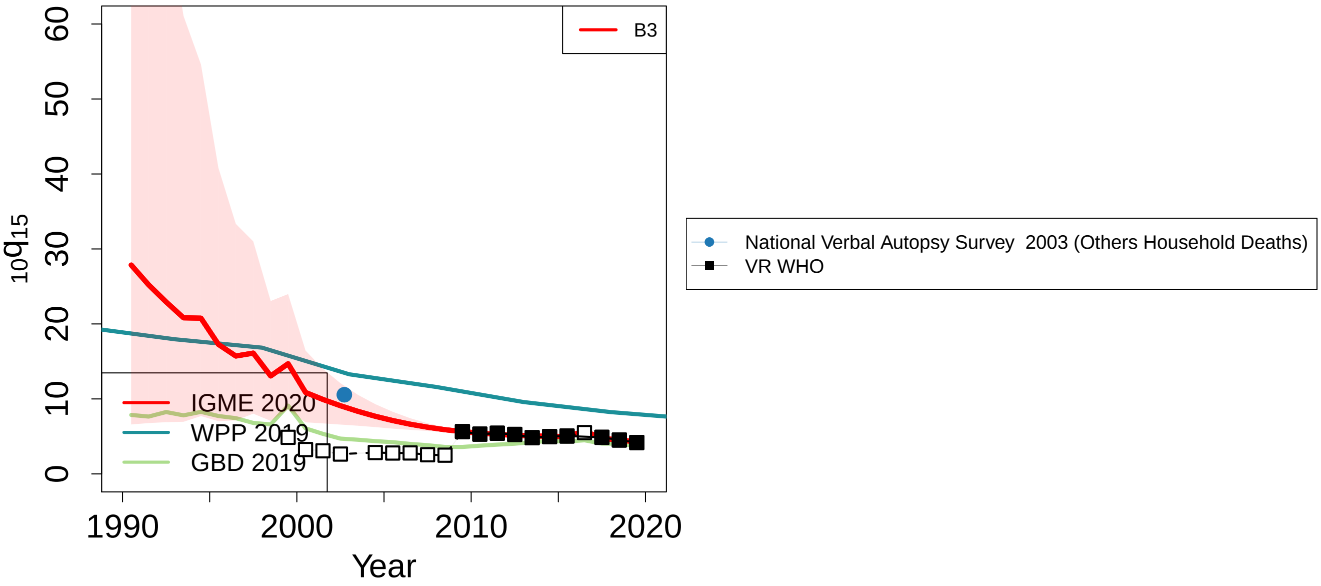

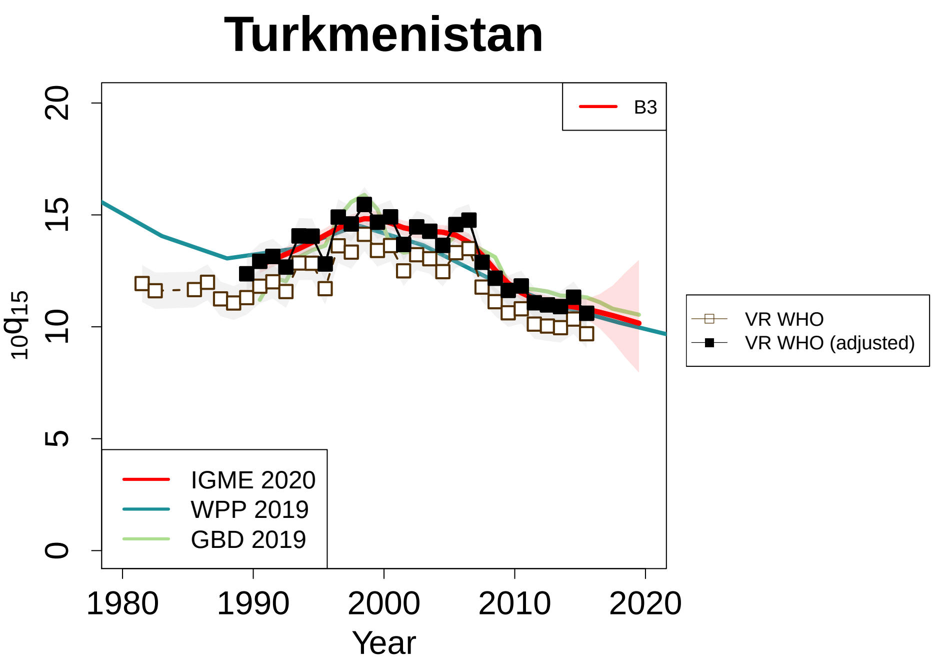
Turkmenistan
B3
VR WHO
VR WHO (adjusted)
IGME 2020
WPP 2019
GBD 2019
$_{10}q_{15}$
0
5
10
15
20
1980
1990
2000
2010
2020
Year

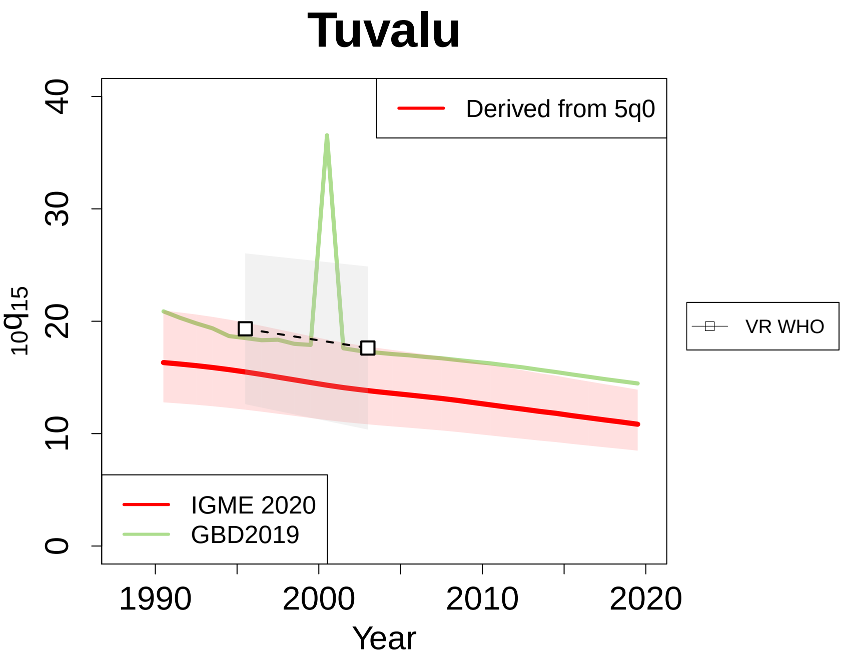
Tuvalu
Derived from 5q0
VR WHO
IGME 2020
GBD2019
$_{10}q_{15}$
0
10
20
30
40
1990
2000
2010
2020
Year

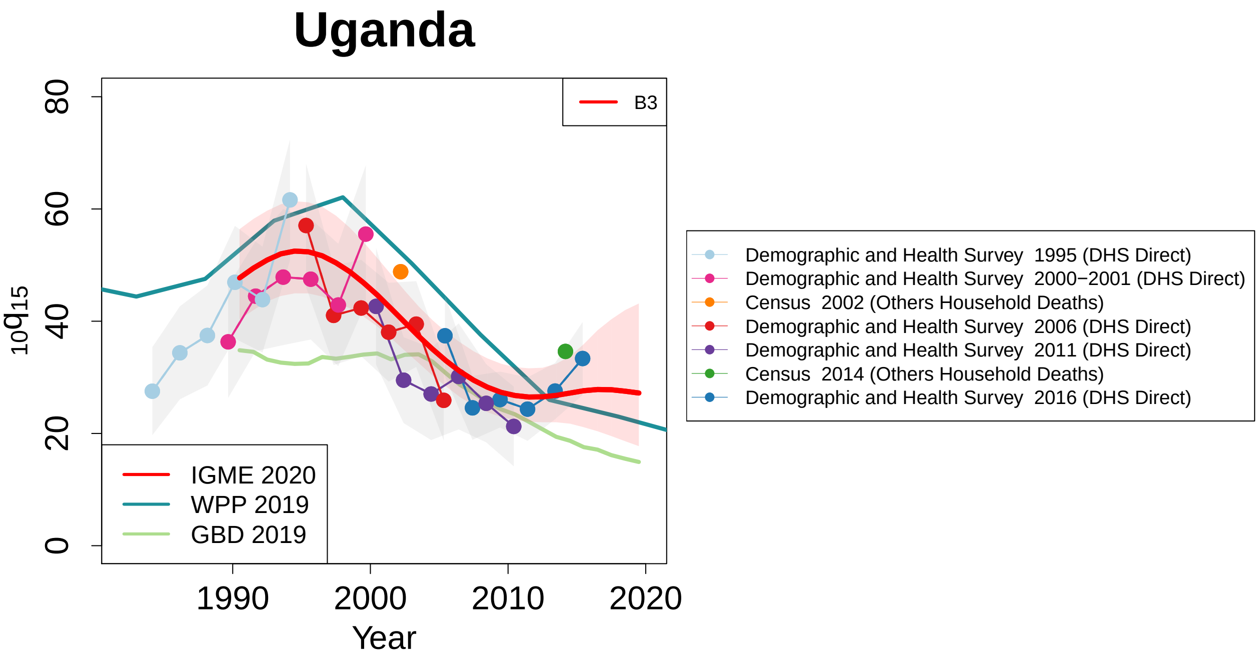
Uganda
B3
Demographic and Health Survey 1995 (DHS Direct)
Demographic and Health Survey 2000−2001 (DHS Direct)
Census 2002 (Others Household Deaths)
Demographic and Health Survey 2006 (DHS Direct)
Demographic and Health Survey 2011 (DHS Direct)
Census 2014 (Others Household Deaths)
Demographic and Health Survey 2016 (DHS Direct)
IGME 2020
WPP 2019
GBD 2019
0
20
40
60
80
$_{10}q_{15}$
1990
2000
2010
2020
Year

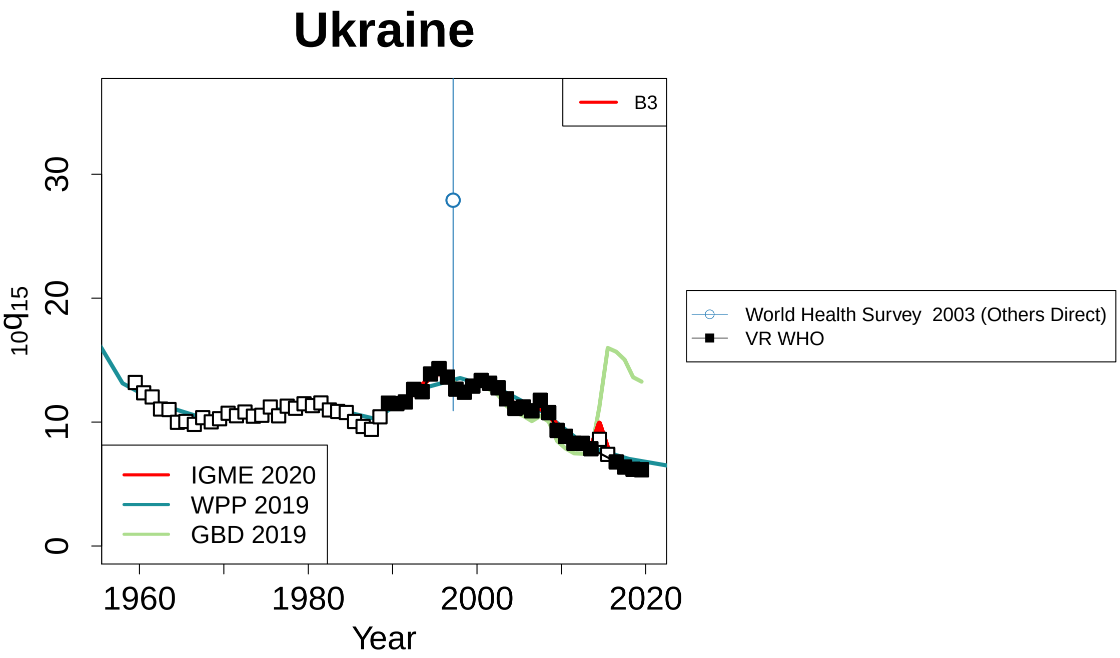
Ukraine
B3
World Health Survey 2003 (Others Direct)
VR WHO
IGME 2020
WPP 2019
GBD 2019
0
10
20
30
$_{10}q_{15}$
1960
1980
2000
2020
Year

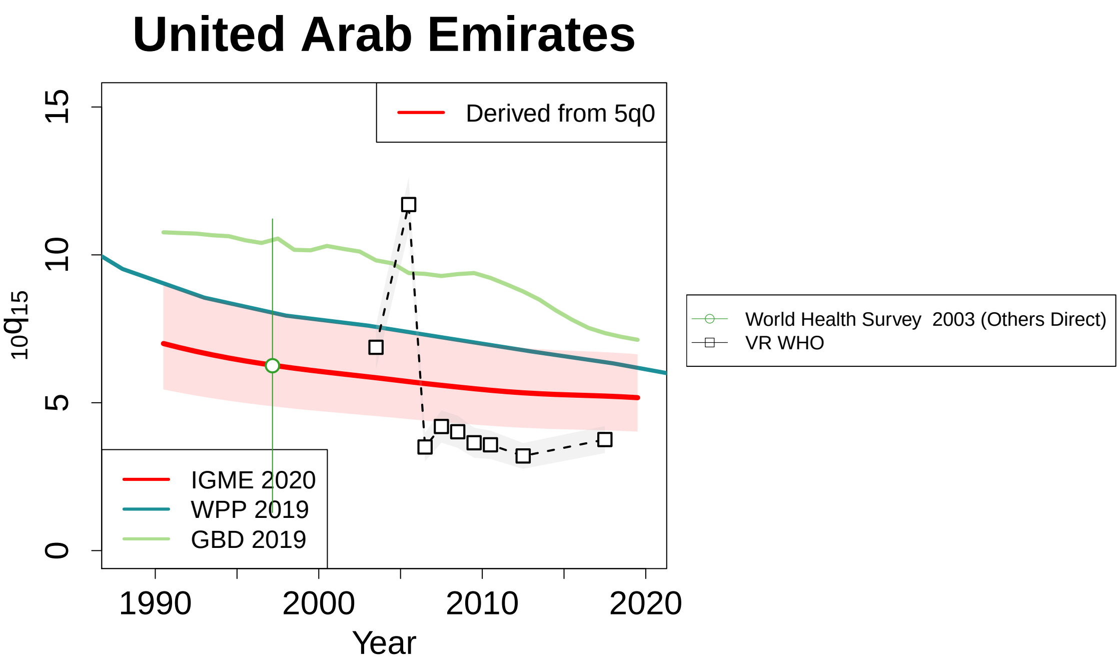
United Arab Emirates
Derived from 5q0
World Health Survey 2003 (Others Direct)
VR WHO
IGME 2020
WPP 2019
GBD 2019
$_{10}q_{15}$
0
5
10
15
1990
2000
2010
2020
Year

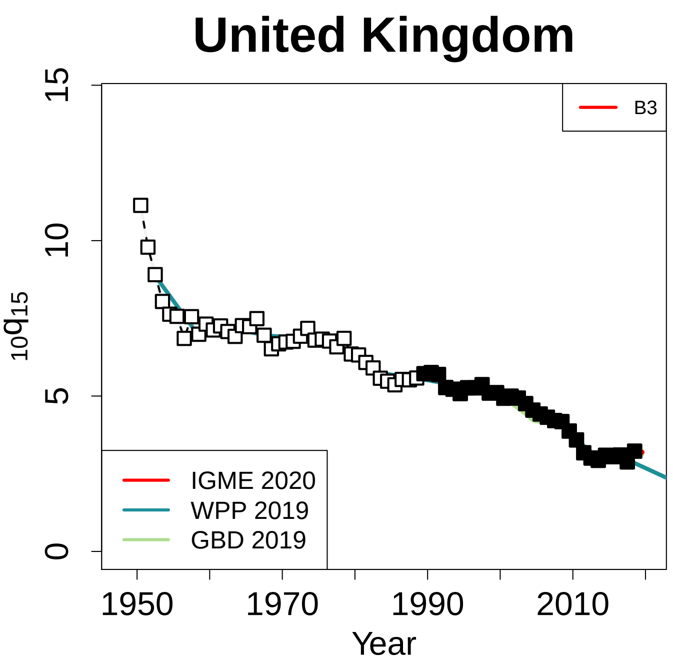
United Kingdom
B3
IGME 2020
WPP 2019
GBD 2019
$_{10}q_{15}$
0
5
10
15
1950
1970
1990
2010
Year

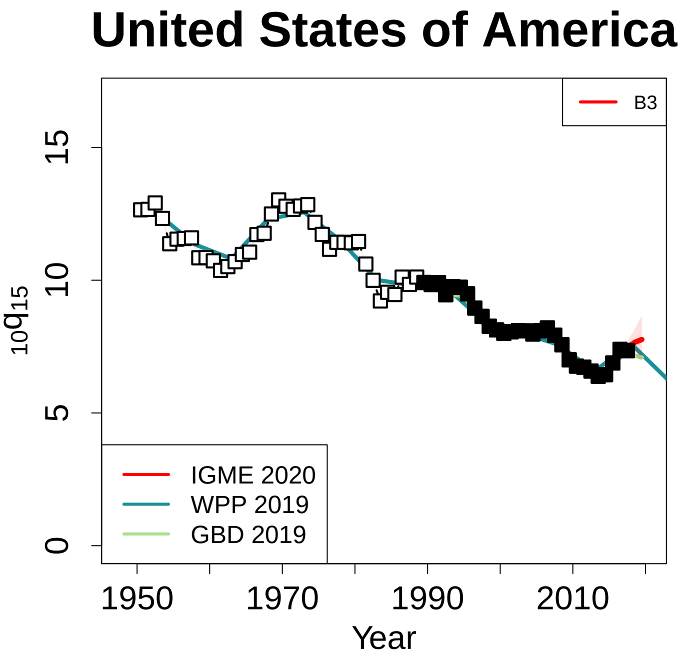
United States of America
B3
15
10
5
0
$_{10}q_{15}$
IGME 2020
WPP 2019
GBD 2019
1950
1970
1990
2010
Year

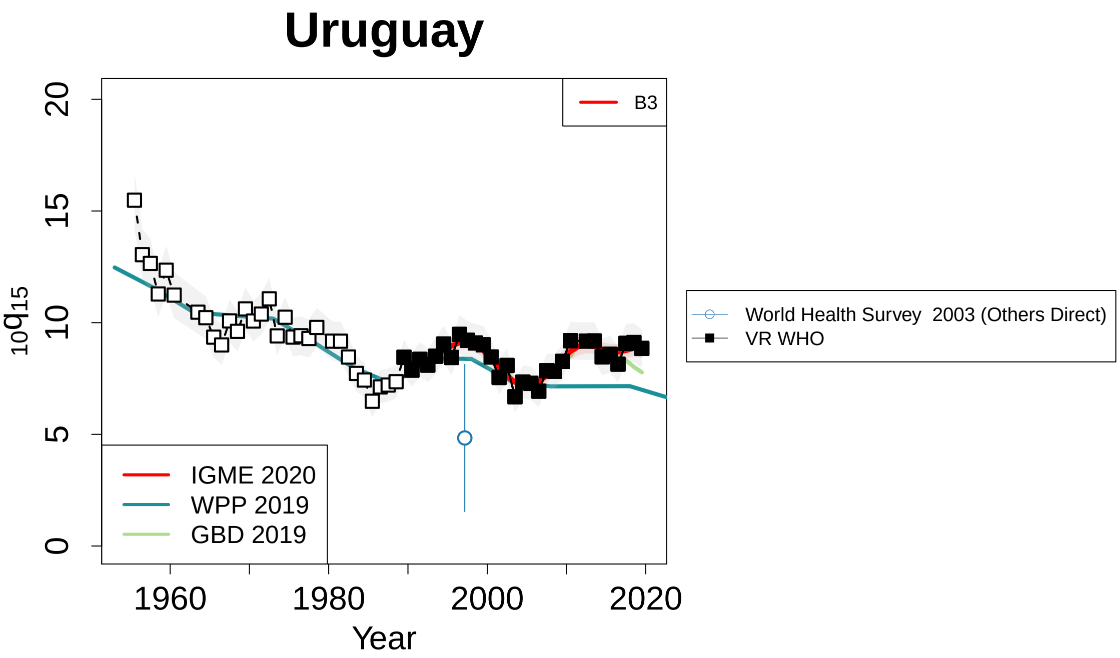
Uruguay
B3
20
15
10
5
0
$_{10}q_{15}$
IGME 2020
WPP 2019
GBD 2019
1960
1980
2000
2020
Year
World Health Survey 2003 (Others Direct)
VR WHO

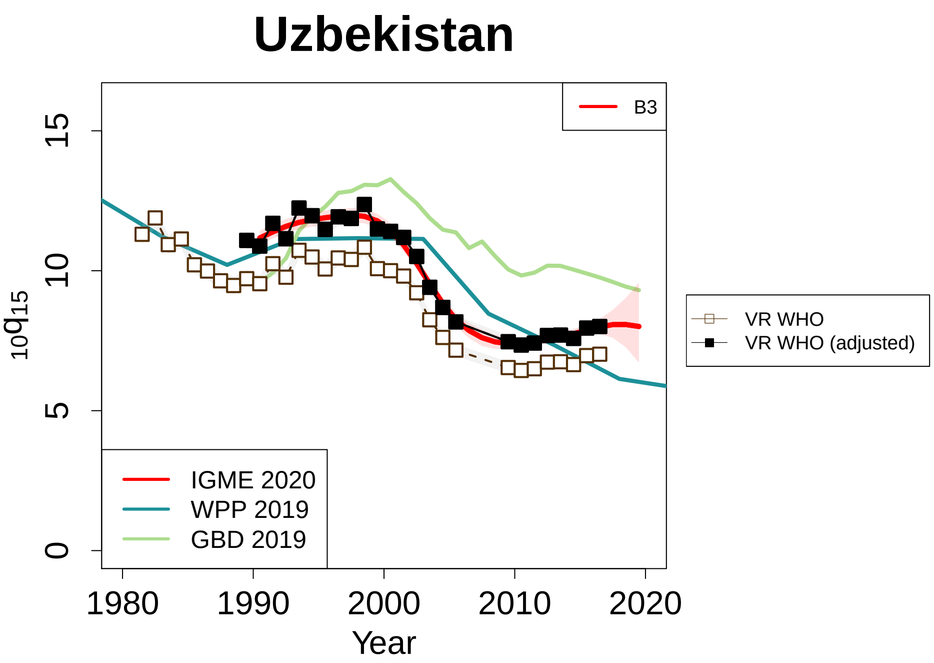
Uzbekistan
B3
VR WHO
VR WHO (adjusted)
IGME 2020
WPP 2019
GBD 2019
$_{10}q_{15}$
0
5
10
15
1980
1990
2000
2010
2020
Year

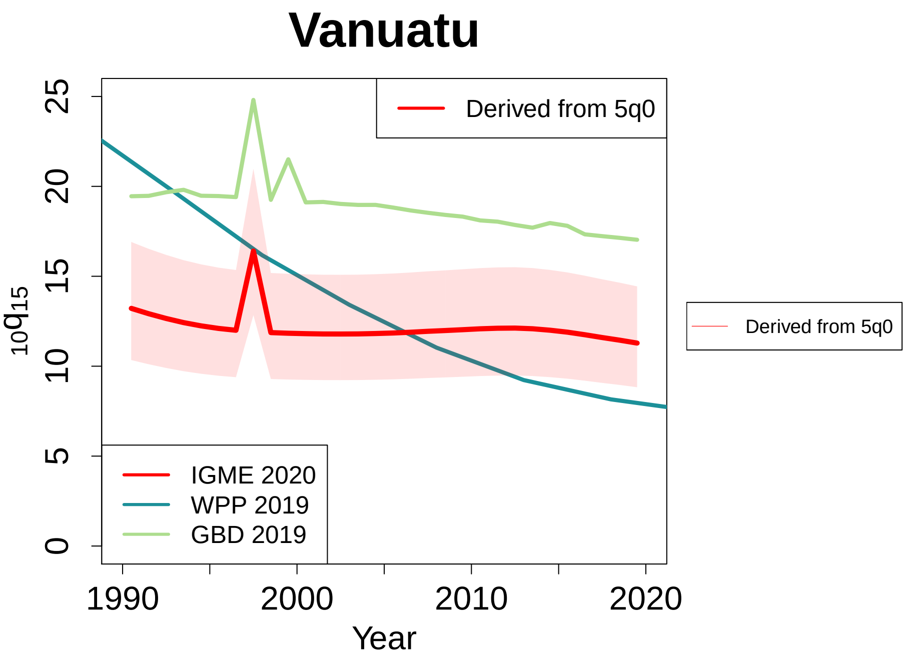
Vanuatu
Derived from 5q0
Derived from 5q0
IGME 2020
WPP 2019
GBD 2019
$_{10}q_{15}$
0
5
10
15
20
25
1990
2000
2010
2020
Year

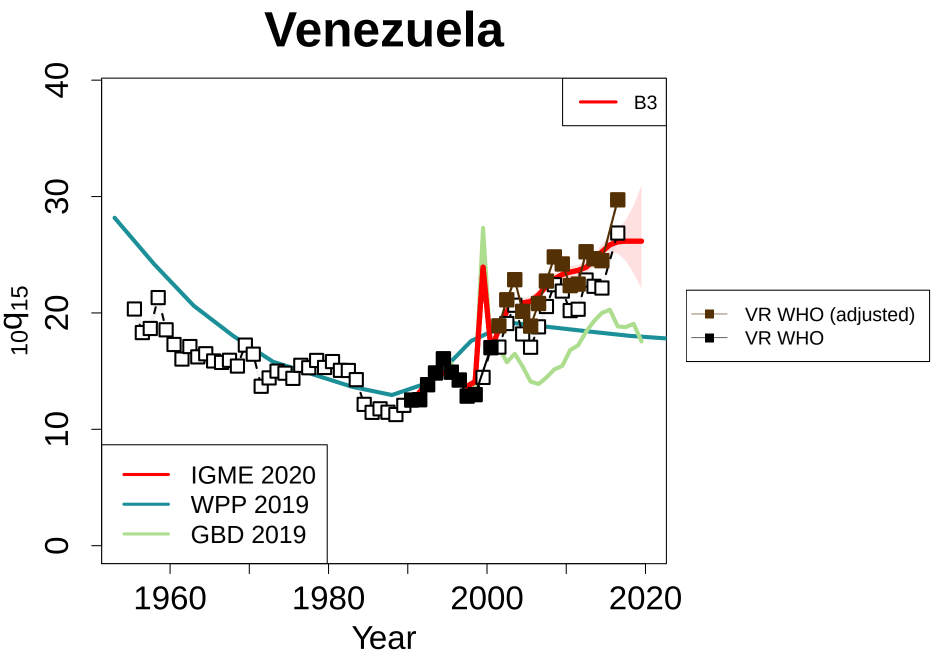
Venezuela
B3
VR WHO (adjusted)
VR WHO
IGME 2020
WPP 2019
GBD 2019
Year
10q15
0
10
20
30
40
1960
1980
2000
2020

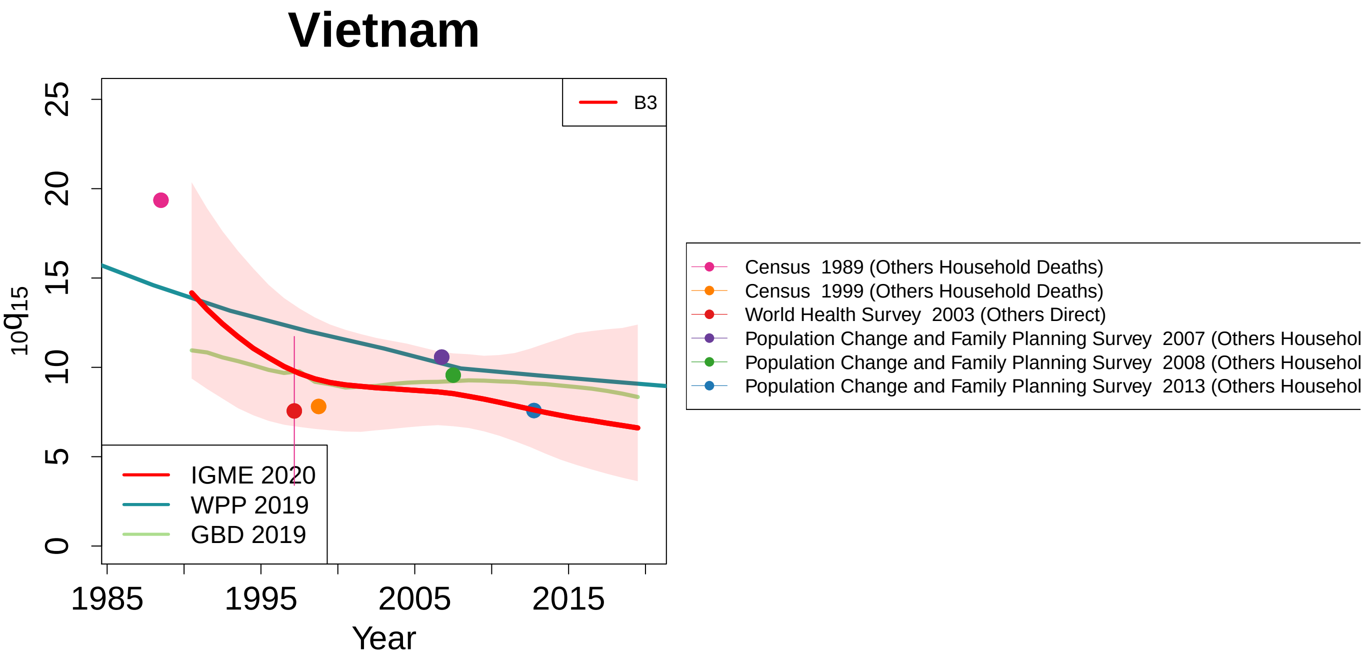
Vietnam
B3
Census 1989 (Others Household Deaths)
Census 1999 (Others Household Deaths)
World Health Survey 2003 (Others Direct)
Population Change and Family Planning Survey 2007 (Others Househol
Population Change and Family Planning Survey 2008 (Others Househol
Population Change and Family Planning Survey 2013 (Others Househol
IGME 2020
WPP 2019
GBD 2019
Year
10q15
0
5
10
15
20
25
1985
1995
2005
2015

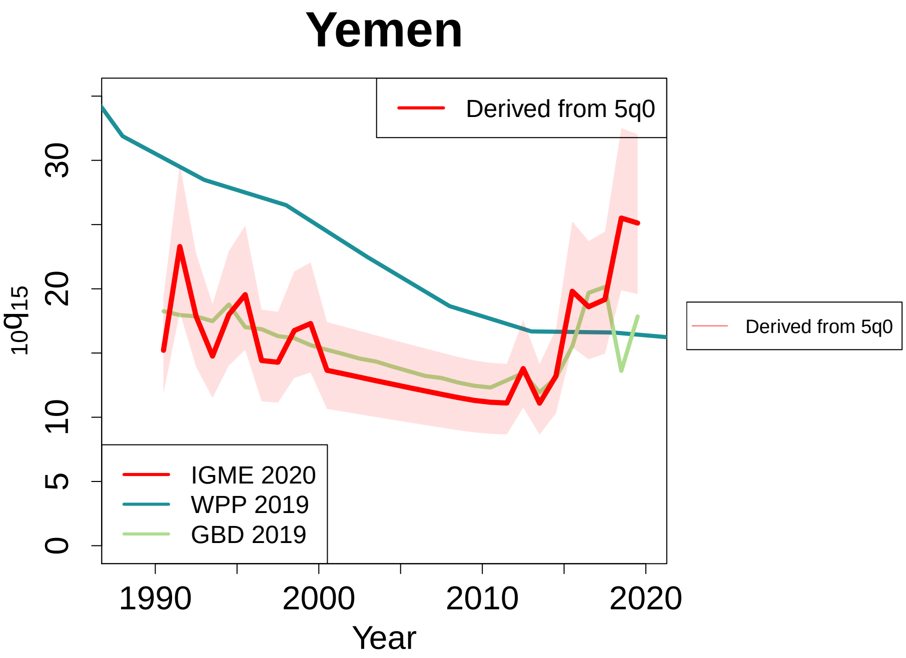
Yemen
Derived from 5q0
IGME 2020
WPP 2019
GBD 2019
0
5
10
20
30
$_{10}q_{15}$
1990
2000
2010
2020
Year
Derived from 5q0

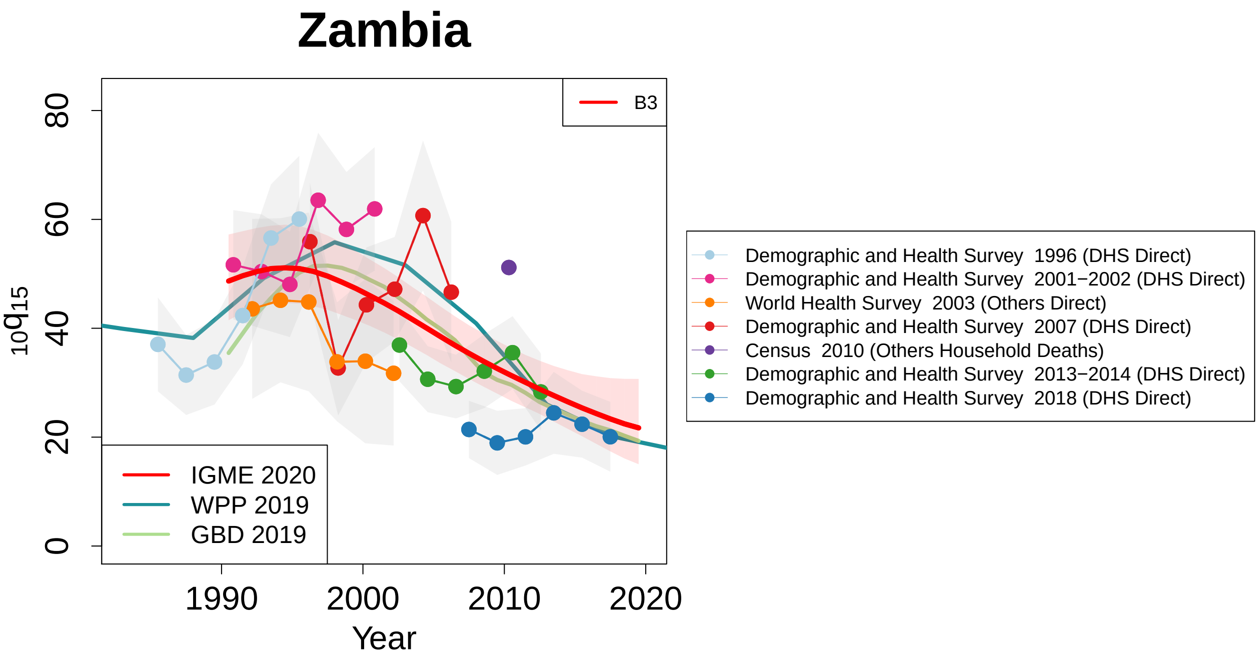
Zambia
B3
IGME 2020
WPP 2019
GBD 2019
0
20
40
60
80
$_{10}q_{15}$
1990
2000
2010
2020
Year
Demographic and Health Survey 1996 (DHS Direct)
Demographic and Health Survey 2001−2002 (DHS Direct)
World Health Survey 2003 (Others Direct)
Demographic and Health Survey 2007 (DHS Direct)
Census 2010 (Others Household Deaths)
Demographic and Health Survey 2013−2014 (DHS Direct)
Demographic and Health Survey 2018 (DHS Direct)

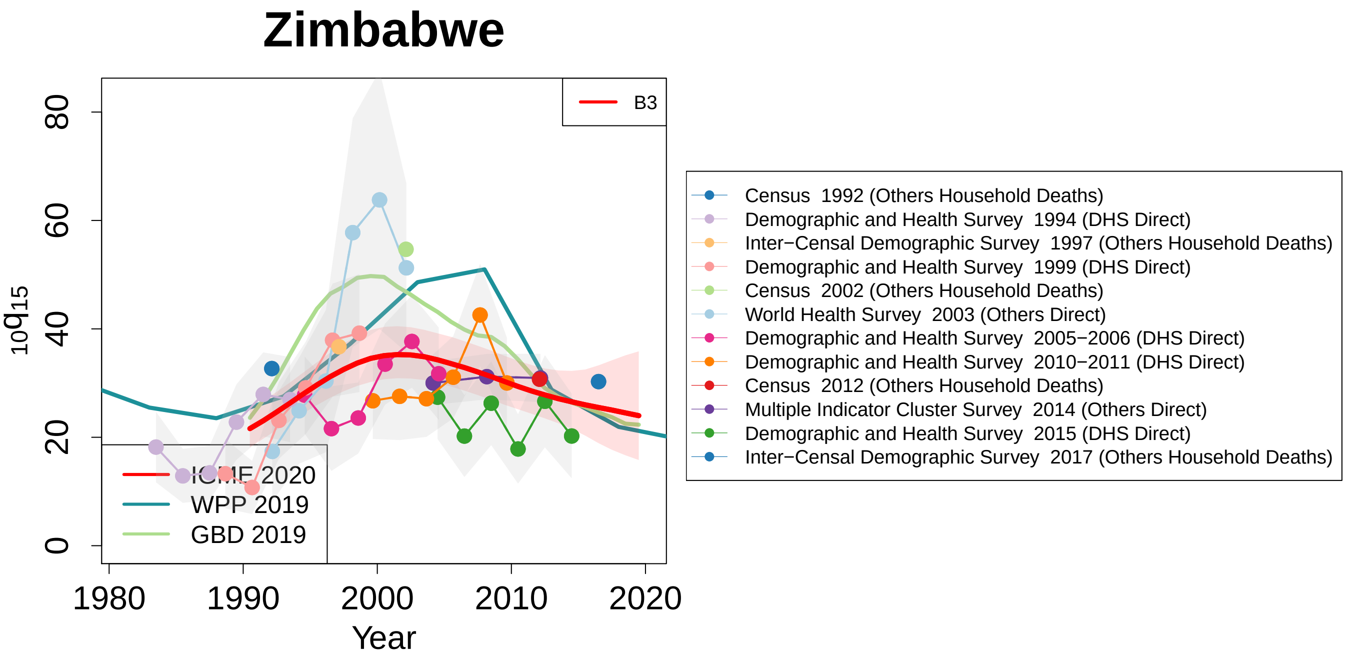
Zimbabwe
B3
IGME 2020
WPP 2019
GBD 2019
$_{10}q_{15}$
0
20
40
60
80
1980
1990
2000
2010
2020
Year
Census 1992 (Others Household Deaths)
Demographic and Health Survey 1994 (DHS Direct)
Inter−Censal Demographic Survey 1997 (Others Household Deaths)
Demographic and Health Survey 1999 (DHS Direct)
Census 2002 (Others Household Deaths)
World Health Survey 2003 (Others Direct)
Demographic and Health Survey 2005−2006 (DHS Direct)
Demographic and Health Survey 2010−2011 (DHS Direct)
Census 2012 (Others Household Deaths)
Multiple Indicator Cluster Survey 2014 (Others Direct)
Demographic and Health Survey 2015 (DHS Direct)
Inter−Censal Demographic Survey 2017 (Others Household Deaths)

6 Comparison with estimates from model life tables, the World Population Prospects 2019 and the Global Burden of Disease 2019

This section briefly compares the 2020 IGME estimates with those that could be obtained by indexing model life tables solely on U5MR, and those of the World Population Prospects 2019 and the Global Burden of Disease 2019. The country-specific plots presented in the previous section also display the estimates from the WPP and GBD.

In countries where the B3 model was used, the median ${}_{10}q_{15}$-to- ${}_{5}q_{0}$ ratio was 0.56 in 2019, increasing from 0.35 in 1990. In 25 countries, this ratio had already exceeded 1 in 2019 (7 in 1990). Figure S5 depicts the ${}_{10}q_{15}$-to- ${}_{5}q_{0}$ ratio estimated from the log-quadratic model based on high-quality vital registration and HDSS data and the ratio encompassed in two standard model life tables; the North and South models of Coale-Demeny life tables. While the B3 estimates conform well to the predictions of the log-quadratic model, they deviate substantially from the standard life tables. This suggests that youth mortality should be inferred from child mortality only as a last resort, and that caution should be exercised when using the traditional model life tables, especially in low-mortality countries.

As indicated in the main text, our estimates contrast with those of the World Population Prospects (WPP) 2019 or the Global Burden of Disease (GBD) 2019 Study. The difference between the latest GBD estimate of the probability ${}_{10}q_{15}$ and our estimate was more than 20% in 95 countries in 2019. The difference was more than 20% in 74 countries when comparing our study with the WPP, and more than 20% in 100 countries when comparing the GBD with the WPP.

To summarize these deviations by region, Table S12 presents the numbers of deaths for the year 2019 in this study against the GBD and WPP estimates. We use here the GBD regional classification to present the uncertainty intervals around GBD values (these are 95% uncertainty intervals). Globally, our estimates fall between the two other series: the number of deaths among 15-24-year-olds in the GBD survey is 12% lower than the estimate from this study, and the WPP estimate is 13% higher than ours. In 6 regions, the absolute differences are greater than 10,000 annual deaths between at least two series: North Africa and Middle East, Southeast Asia, South Asia, Western Sub-Saharan Africa, Eastern Sub-Saharan Africa and Central Sub-Saharan Africa. In North Africa and the Middle East, the total number of deaths in the GBD and WPP are respectively 31% and 18% lower than ours. In South-East Asia, the IGME and WPP estimates are consistent and lower than the WPP, while in South Asia, our estimates are lower than the other two series. But the most striking discrepancies are in sub-Saharan Africa. In West Africa, the WPP estimates of deaths among 15-24-year-olds are almost three times higher than those of the GBD survey, and in Central Africa, almost twice as high. Differences between estimates of adult mortality between the ages of 15 and 59 and maternal mortality in Africa have already been much debated [41], and this debate resonates here. Our estimates are consistently higher than the GBD values, while the consistency with WPP estimates varies

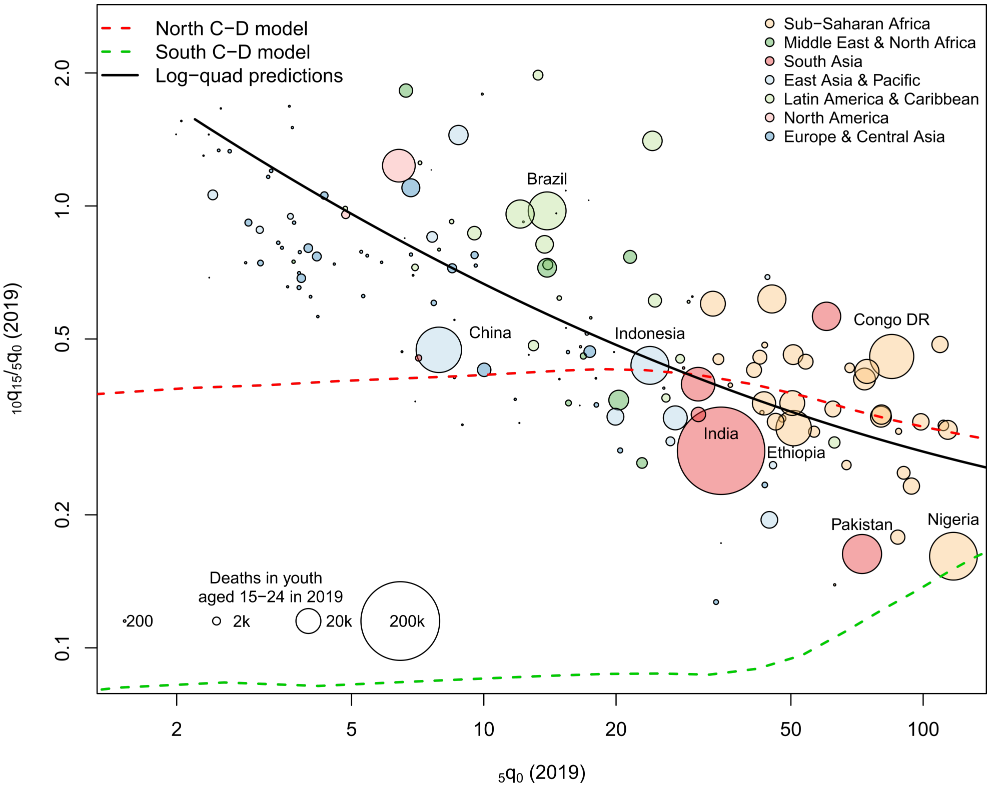

Figure S5 – *Ratio of the probability $_{10}q_{15}$ to the probability $_5q_0$ in 2019, according to the level of under-five mortality, in B3 estimates, the log-quadratic predictions and the North and South models of Coale-Demeny (C-D) life tables. Countries for which $_{10}q_{15}$ was inferred from $_5q_0$ are excluded.*

across sub-regions. It is difficult to make sense of these variations, which are due both to adjustments introduced to account for biases in sibling histories and to differences in the methods for reconstructing mortality trends. Deviations in the population at risk of dying do not play a large role here (the GBD estimate of the population aged 15-24 in Sub-Saharan Africa in 2019 was 3% higher than the WPP estimate). Given the increasing concentration of deaths in Sub-Saharan Africa, more research is warranted to disentangle the main drivers of deviations between mortality estimates.

	Adolescents aged 15-19			Young people aged 20-24		
	GBD 2019	WPP 2019	IGME 2020	GBD 2019	WPP 2019	IGME 2020
World	499	663	595	692	857	756
	(465-536)		(569-638)	(645-738)		(695-893)
High-income North America	12	11	12	21	23	24
	(12-12)		(11-13)	(21-21)		(21-28)
Australasia	1	1	1	1	1	1
	(1-1)		(1-1)	(1-1)		(1-1)
High-income Asia Pacific	2	2	2	3	3	3

	Adolescents aged 15-19			Young people aged 20-24		
	GBD 2019	WPP 2019	IGME 2020	GBD 2019	WPP 2019	IGME 2020
	(2-2)		(2-2)	(3-3)		(3-3)
Western Europe	5	5	5	9	8	8
	(5-6)		(5-5)	(9-9)		(8-9)
Southern Latin America	3	3	3	4	5	5
	(3-3)		(3-3)	(4-5)		(4-5)
Eastern Europe	7	6	5	13	11	9
	(6-8)		(5-5)	(12-15)		(8-9)
Central Europe	2	2	2	3	3	3
	(2-2)		(2-2)	(3-4)		(3-3)
Central Asia	5	4	4	8	6	6
	(4-5)		(4-4)	(7-9)		(5-7)
Central Latin America	20	21	25	31	35	36
	(17-23)		(24-25)	(27-36)		(33-39)
Andean Latin America	4	5	3	6	8	6
	(3-4)		(3-4)	(5-7)		(3-10)
Caribbean	3	5	3	5	6	5
	(3-4)		(2-4)	(4-6)		(3-8)
Tropical Latin America	18	18	20	27	29	28
	(17-19)		(18-21)	(26-28)		(24-33)
East Asia	29	30	24	49	45	46
	(25-33)		(17-31)	(42-56)		(24-84)
Southeast Asia	44	55	46	61	71	60
	(40-49)		(38-56)	(54-68)		(39-94)
Oceania	2	2	1	2	2	2
	(1-2)		(1-2)	(2-3)		(1-2)
North Africa and Middle East	38	46	58	51	60	71
	(34-44)		(53-64)	(45-59)		(58-91)
South Asia	155	153	149	219	206	194
	(142-168)		(129-172)	(198-242)		(132-270)
Southern Sub-Saharan Africa	11	10	12	20	17	19
	(10-13)		(11-13)	(18-23)		(15-24)
Western Sub-Saharan Africa	58	169	92	66	181	100
	(50-68)		(80-110)	(56-77)		(74-129)
Eastern Sub-Saharan Africa	62	81	84	70	97	103
	(53-72)		(74-99)	(61-80)		(76-136)
Central Sub-Saharan Africa	19	35	44	24	40	45
	(16-22)		(32-61)	(19-27)		(22-85)

Table S12 – *Comparison of numbers of deaths (in thousands) by GBD regions, GBD 2019, WPP 2019, and estimates derived from the B3 model for the year 2019.*
Note: the uncertainty intervals around the IGME estimates are 90% UIs, while the uncertainty intervals around the GBD estimates are 95% UIs.

7 UNICEF regional classification

Countries included in this study are classified in geographical regions according to the UNICEF classification (Figure S6). Aggregated estimates based on other regional classifications (SDG, WHO, etc.) are available at www.childmortality.org.

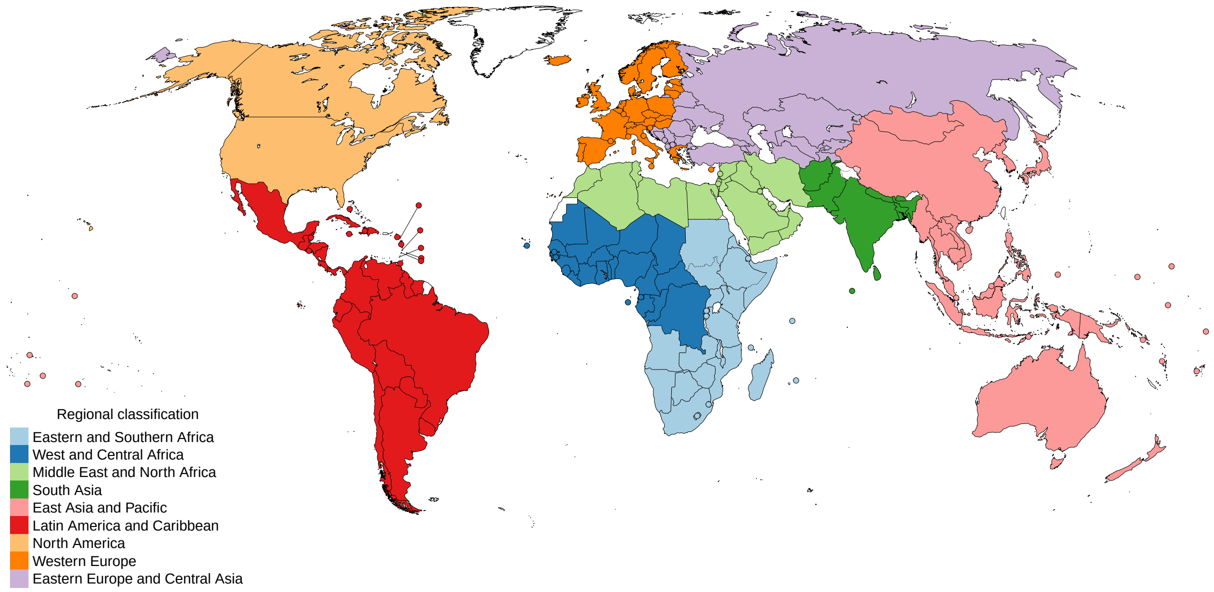

Figure S6 – *UNICEF Regional classification*

8 United Nations Inter-Agency Group for Child Mortality Estimation

The UN IGME is led and coordinated by the United Nations Children's Fund (UNICEF) and comprises the WHO, the UN Population Division and the World Bank Group. It was formed in 2004 to share data on child mortality, harmonize estimates within the UN system, improve methods for child mortality estimation, report on progress towards child survival goals and enhance country capacity to produce timely and properly assessed estimates of child mortality. The organizations and individuals involved in generating the 2020 estimates of mortality in children, adolescents and youth are the following:

- *United Nations Children's Fund*: Lucia Hug, Sinae Lee, Yang Liu, Anupam Mishra, David Sharrow, Danzhen You
- *World Health Organization*: Bochen Cao, Jessica Ho, Kathleen Louise Strong
- *World Bank Group*: Emi Suzuki
- *United Nations, Department of Economic and Social Affairs, Population Division*: Kirill Andreev, Lina Bassarsky, Victor Gaigbe-Togbe, Patrick Gerland, Danan Gu, Sara Hertog, Nan Li, Thomas Spoorenberg, Philipp Ueffing, Mark Wheldon
- *United Nations Economic Commission for Latin America and the Caribbean, Population Division*: Guiomar Bay, Helena Cruz Castanheira

The UN IGME's Technical Advisory Group provides guidance on estimation methods, technical issues and strategies for data analysis and data quality assessment. Its members are Leontine Alkema (University of Massachusetts, Amherst), Robert Black (Johns Hopkins University), Simon Cousens (London School of Hygiene and Tropical Medicine), Trevor Croft (The Demographic and Health Surveys Program, ICF), Michel Guillot (University of Pennsylvania and French Institute for Demographic Studies), Kenneth Hill (Stanton-Hill Research), Bruno Masquelier (University of Louvain), Colin Mathers (University of Edinburgh), Jon Pedersen (Mikro!), Jon Wakefield (University of Washington) and Neff Walker (Johns Hopkins University).

References

[1] T. Moultrie, R. Dorrington, A. Hill, K. Hill, I. Timæus, and B. Zaba, *Tools for demographic estimation.* Paris: International Union for the Scientific Study of Population, 2013.

[2] K. Hill, "Estimating Census and Death Registration Completeness," *Asian and Pacific Population Forum*, vol. 1(3), pp. 8–13, 1987.

[3] N. Bennett and S. Horiuchi, "Mortality estimation from registered deaths in less developed countries," *Demography*, vol. 21(2), pp. 217–234, 1984.

[4] K. Hill, D. You, and Y. Choi, "Death distribution methods for estimating adult mortality: Sensitivity analysis with simulated data error," *Demographic Research*, vol. 21, pp. 235–254, 2009.

[5] C. Murray, J. Rajaratnam, J. Marcus, T. Laakso, and A. Lopez, "What Can We Conclude from Death Registration? Improved Methods for Evaluating Completeness," *PLoS Med*, vol. 7, p. e1000262, 04 2010.

[6] T. Riffe, E. Lima, and B. Queiroz, *DDM: Death Registration Coverage Estimation*, 2017. R package version 1.0-0.

[7] World Health Organization, *Neonatal and Perinatal Mortality: Country, Regional and Global Estimates.* World Health Organization, 2006.

[8] D. Dicker *et al.*, "Global, regional, and national age-sex-specific mortality and life expectancy, 1950-2017: a systematic analysis for the global burden of disease study 2017," *The Lancet*, vol. 392, pp. 1684–1735, Nov. 2018.

[9] K. Hill, *Analytical methods to evaluate the completeness and quality of death registration: Current state of knowledge*, 2017.

[10] L. I. Lin, "A concordance correlation coefficient to evaluate reproducibility," *Biometrics*, vol. 45, pp. 255–268, Mar 1989.

[11] C. Rao and K. Matthew, "Overview of the principles and international experiences in implementing record linkage mechanisms to assess completeness of death registration." Population Division Technical Paper, No 2017/5, 2017.

[12] N. Li and P. Gerland, "Evaluating the completeness of death registration at old ages: A new method and its application to developed and developing countries." Population Division Technical Paper No. 2019/5, 2019.

[13] K. Hill, Y. Choi, and I. Timæus, "Unconventional approaches to mortality estimation," *Demographic research*, vol. 13(12), pp. 281–300, 2005.

[14] R. Özdemir, C. Rao, Z. Öcek, and G. D. Horasan, "Reliable mortality statistics for turkey: Are we there yet?," *BMC Public Health*, vol. 15, June 2015.

[15] United Nations, *World Population Prospects: The 2019 Revision.* UN, 2019.

[16] J. Pedersen and J. Liu, "Child mortality estimation: appropriate time periods for child mortality estimates from full birth histories," *PLoS Med.*, vol. 9, no. 8, p. e1001289, 2012.

[17] E. Gakidou and G. King, "Death by survey: estimating adult mortality without selection bias from sibling survival data," *Demography*, vol. 43 (3), pp. 569–585, 2006.

[18] B. Masquelier, "Adult Mortality from Sibling Survival Data: A Reappraisal of Selection Biases," *Demography*, vol. Volume 50, Issue 1, pp. 207–228, 2013.

[19] G. Rodriguez and J. Trussell, "Maximum likelihood estimation of the parameters of Coale's model nuptiality schedule from survey data." World Fertility Survey, Technical Bulletins, No 7, 1980.

[20] S. Helleringer, G. Pison, M. Kanté, G. Duthé, and A. Andro, "Reporting errors in survey data on adult mortality: results from a record linkage study in Senegal," *Demography*, vol. 51, no. 2, pp. 387–411, 2014.

[21] S. Helleringer, G. Pison, B. Masquelier, A. Kanté, L. Douillot, G. Duthé, C. Sokhna, and V. Delaunay, "Improving the quality of adult mortality data collected in demographic surveys: Validation study of a new siblings' survival questionnaire in niakhar, senegal," *PLoS Med*, vol. 11, p. e1001652, 05 2014.

[22] G. Reniers, B. Masquelier, and P. Gerland, "Adult Mortality in Africa," in *International Handbook of Adult Mortality* (R. Rogers and E. Crimmins, eds.), Springer, 2011.

[23] B. Masquelier, J. W. Eaton, P. Gerland, F. Pelletier, and K. K. Mutai, "Age patterns and sex ratios of adult mortality in countries with high hiv prevalence," *AIDS*, vol. 31 Suppl 1, pp. S77–S85, Apr 2017.

[24] Z. Obermeyer, J. Rajaratnam, C. Park, E. Gakidou, M. Hogan, A. Lopez, and C. Murray, "Measuring Adult Mortality Using Sibling Survival: A New Analytical Method and New Results for 44 Countries, 1974-2006," *PLoS Medicine*, vol. 7, p. e1000260, 04 2010.

[25] I. Timæus and M. Jasseh, "Adult mortality in Sub-Saharan Africa: evidence from demographic and health survey," *Demography*, vol. 41, 4, pp. 757–772, 2004.

[26] C. Stanton, A. Noureddine, and K. Hill, "DHS Maternal Mortality Indicators: an assessment of data quality and implications for Data Use," *Studies in Family planning*, vol. 31,2, pp. 111–123, 2000.

[27] B. Masquelier, "Sibship Sizes and Family Sizes in Survey Data Used to Estimate Mortality," *Population-E*, vol. 69, no. 2, pp. 249–268, 2014.

[28] M. Barbieri, J. R. Wilmoth, V. M. Shkolnikov, D. Glei, D. Jasilionis, D. Jdanov, C. Boe, T. Riffe, P. Grigoriev, and C. Winant, "Data resource profile: The human mortality database (HMD)," *International Journal of Epidemiology*, vol. 44, pp. 1549–1556, June 2015.

[29] J. Banister and K. Hill, "Mortality in China 1964-2000," *Popul Stud (Camb)*, vol. 58, no. 1, pp. 55–75, 2004.

[30] R. Dorrington, "The generalized growth balance method," in *Tools for demographic estimation* (T. Moultrie, R. Dorrington, A. Hill, K. Hill, I. Timæus, and B. Zaba, eds.), Paris: International Union for the Scientific Study of Population, 2013.

[31] L. Alkema and J. R. New, "Global estimation of child mortality using a bayesian b-spline bias-reduction model," *The Annals of Applied Statistics*, vol. 8, no. 4, pp. 2122–2149, 2014.

[32] P. H. C. Eilers and B. D. Marx, "Flexible smoothing with b -splines and penalties," *Statist. Sci.*, vol. 11, pp. 89–121, 05 1996.

[33] P. H. C. Eilers and B. D. Marx, "Splines, knots, and penalties," *Wiley Interdisciplinary Reviews: Computational Statistics*, vol. 2, no. 6, pp. 637–653, 2010.

[34] I. D. Currie and M. Durban, "Flexible smoothing with p-splines: a unified approach," *Statistical Modelling*, vol. 2, no. 4, pp. 333–349, 2002.

[35] Department of Peace and Conflict Research, Uppsala University, "Uppsala Conflict Data Program." Available at https://ucdp.uu.se/.

[36] WHO, "WHO methods and data sources for life tables 1990-2016." Global Health Estimates Technical Paper WHO/HIS/IER/GHE/2018.2, available at https://www.who.int/healthinfo/statistics/en/, 2018.

[37] C. Raleigh, A. Linke, H. Hegre, and J. Karlsen, "Introducing ACLED: An armed conflict location and event dataset," *Journal of Peace Research*, vol. 47, pp. 651–660, Sept. 2010.

[38] ACLED, "Armed conflict location and event data project." Available at https://acleddata.com.

[39] Centre for Research on the Epidemiology of Disasters, "EM-DAT: The International Disaster Database." Available at https://www.emdat.be/.

[40] A. Gelman and J. Hill, *Data analysis using regression and multilevel/Hierarchical models*. Cambridge University Press, 2007.

[41] N. J. Kassebaum, A. D. Lopez, C. J. L. Murray, and R. Lozano, "A comparison of maternal mortality estimates from GBD 2013 and WHO," *The Lancet*, vol. 384, pp. 2209–2210, Dec. 2014.
